# Mercury alters endogenous phosphorylation profiles of SYK in murine B cells

**DOI:** 10.1186/s12865-017-0221-0

**Published:** 2017-07-17

**Authors:** Joseph A. Caruso, Nicholas Carruthers, Namhee Shin, Randal Gill, Paul M. Stemmer, Allen Rosenspire

**Affiliations:** 1 Institute of Environmental Health Sciences, Center for Urban Responses to Environmental Stressors (CURES), Wayne State University, Detroit, MI 48201 USA; 2 Department of Immunology and Microbiology, Center for Urban Responses to Environmental Stressors (CURES), Wayne State University, Detroit, MI 48201 USA

**Keywords:** Mercury, Syk, B cell, Autoimmune disease, Proteomics, Phosphorylation

## Abstract

**Background:**

Epidemiological evidence and animal models suggest that exposure to low and non-neurotoxic concentrations of mercury may contribute to idiosyncratic autoimmune disease. Since defects in function and signaling in B cells are often associated with autoimmunity, we investigated whether mercury exposure might alter B cell responsiveness to self-antigens by interfering with B cell receptor (BCR) signal transduction. In this study we determined the effects of mercury on the protein tyrosine kinase SYK, a critical protein involved in regulation of the BCR signaling pathway.

**Methods:**

Phosphorylation sites of murine SYK were mapped before and after treatment of WEHI cell cultures with mercury, or with anti-IgM antibody (positive control) or pervanadate (a potent phosphatase inhibitor). Phosphopeptides were enriched by either titanium dioxide chromatography or anti-phosphotyrosine immunoaffinity, and analyzed by liquid chromatography-mass spectrometry. Select SYK phosphosite cluster regions were profiled for responsiveness to treatments using multiple reaction monitoring (MRM) methodology.

**Results:**

A total of 23 phosphosites were identified with high probability in endogenous SYK, including 19 tyrosine and 4 serine residues. For 10 of these sites phosphorylation levels were increased following BCR activation. Using MRM to profile changes in phosphorylation status we found that 4 cluster regions, encompassing 8 phosphosites, were activated by mercury and differentially responsive to all 3 treatments. Phosphorylation of tyrosine-342 and -346 residues were most sensitive to mercury exposure. This cluster is known to propagate normal BCR signal transduction by recruiting adaptor proteins such as PLC-γ and Vav-1 to SYK during formation of the BCR signalosome.

**Conclusions:**

Our data shows that mercury alters the phosphorylation status of SYK on tyrosine sites known to have a role in promoting BCR signals. Considering the importance of SYK in the BCR signaling pathway, these data suggest that mercury can alter BCR signaling in B cells, which might affect B cell responsiveness to self-antigen and have implications with respect to autoimmunity and autoimmune disease.

**Electronic supplementary material:**

The online version of this article (doi:10.1186/s12865-017-0221-0) contains supplementary material, which is available to authorized users.

## Background

Understanding immunological tolerance and the mechanisms that lead to activation of self-reactive lymphocytes and autoimmunity is a fundamental problem in immunology. Autoimmune diseases arise when an immune response is mounted against tissues or a specific molecule normally found in the body. B cells have roles in humoral immunity and the adaptive immune system through secretion of antibodies and inflammatory cytokines, presentation of antigens, and generation of ectopic germinal centers. Defects in B cell development, selection, and function can lead to autoimmunity [1, 2].

B cells originate in the bone marrow and must transit several checkpoints before reaching maturity. Between 50 and 75% of newly produced immature B cells are autoreactive, and many are removed by tolerance mechanisms in the bone marrow before migrating to the spleen where a different set autoantigens are presented [3]. B cells with the highest avidity to these autoantigens are deleted, whereas B cells with lower avidity survive for a period as anergic cells. Immature B cells with the lowest avidity to self-antigens mature and eventually migrate to germinal centers or marginal zones where they are activated by foreign antigen. These B cells eventually differentiate into antibody-producing plasma cells or memory cells.

A question remains as to why a pool of self-reactive anergic B cells are maintained since this silenced population could potentially be reactivated with self-destructive results. We have proposed that a critical function of anergic cells is to respond against pathogens which have evolved to mimic their host [4]. This would allow the host to neutralize an infection that initially evades the immune system through a cloaking stratagem, however, inappropriate activation of anergic cells would lead to recognition of self-antigens and unnecessary autoimmune responses.

A central issue in contemporary immunology is how the fate of B cells, particularly immature B cells and anergic B cells, are determined. It is now clear that B cell fate decisions are dependent upon BCR signaling, so that disruption of BCR signaling mechanisms in immature B cells can disrupt negative selection of self-reactive clones and lead to production of autoreactive B cells and autoimmune disease [5, 6]. Disruption of BCR signaling can occur as a consequence of genetic abnormalities, environmental factors, or a combination of both [7].

Mercury is a toxic heavy metal which is deposited in the environment from natural sources such as volcanic eruptions and forest fires; and anthropomorphic sources such as burning of coal, steel and cement plant emissions, and municipal/hospital waste incineration. Exposure to mercury can occur through occupation or diet, with the latter occurring predominantly through consumption of contaminated fish. Inorganic mercury accumulates in lakes and oceans where chemical and microbial activities convert it to organic mercury species which are more efficiently absorbed and transported within the body. Organic mercury accumulates in various tissues, predominantly in kidney, liver and muscle tissue, by binding to thiol moieties of proteins. Thus certain fish species near the top of the food chain maintain higher mercury burdens.

BCR activation leads to recruitment LYN and SYK proteins on the inner membrane. SYK is then activated through autophosphorylation and phosphorylation by LYN. Downstream targets of activated SYK include BLNK, Btk, Vav-1, HPK-1 and PLC-γ. Thus the functionality of both LYN and SYK are controlled by differential phosphorylation of multiple regulatory sites on both kinases. We have shown that mercury alters the phosphorylation status of LYN regulatory sites which are involved in BCR signaling [8]. These findings are extended here by profiling site specific phosphorylation of SYK in response to mercury in the WEHI-231 model of immature B cells.

## Methods

### Cell culture

Murine WEHI-231 B cell lymphoma cells were obtained from the American Type Culture Collection, Rockville MD. Cells were maintained in RPMI 1640 supplemented with 10% fetal bovine serum, 2 mM glutamine, 50 μM mercaptoethanol, 100 U/ml penicillin and 100 μg/ml streptomycin in a humidified 5% CO_2_ atmosphere. Cells were passaged three times a week and harvested for experiments while in logarithmic growth. On the day of the experiment, cells were washed, counted, and resuspended in serum-free medium.

### SYK Immunoprecipitation

7.5 × 10^7^ WEHI cells per 5 ml were mock treated or treated with activating antibody (100 μg of anti-immunoglobulin M per 10^6^ cells) for the indicated times, or the indicated concentrations of pervanadate or HgCl_2_ for 10 min at 37 °C. At the endpoint, cells were cooled in an ice bath, pelleted at 4 °C, washed with ice-cold wash buffer [150 mM NaCl, 50 mM Tris (pH 7.5), 1 mM NaF, 1 mM Na_3_VO_4_, 2 mM β-glycerophosphate], and re-pelleted. Cells were lysed with wash buffer containing 1% NP-40, 0.5% deoxycholate, 0.1% SDS, 2 mM MgCl_2_ and 100 U Benzonase (Novagen). After a 5 min digestion at 37 °C, 1X Protease Inhibitor Cocktail (Sigma) and 10 mM EDTA were added. Samples were centrifuged at high speed and supernatants transferred to new tubes, pre-cleared with Protein A/G-agarose, and then immunoprecipitated with 3 μg each of 2 anti-SYK antibodies (mouse monoclonal sc-73089 and rabbit polyclonal sc-1077, Santa Cruz Biotechnology) and 50 μl of Protein A/G-agarose overnight with end-over-end rotation at 4 °C. The following day, the beads were washed and proteins eluted by boiling with SDS sample buffer under reducing conditions. Proteins were separated by SDS-PAGE using 4-12% Bis-Tris gels (Life Technologies). For initial experiments proteins were transferred to nitrocellulose for western blotting to determine the migration of SYK. In subsequent experiments gels were stained with Coomassie Blue and gel slices were excised for mass spectrometric analysis.

### Phosphopeptide enrichment

WEHI cells were treated with anti-IgM (100 μg/10^6^ cells) for 0, 1, 2, 3, 5 and 15 min; or with HgCl_2_ for 10 min at 0, 1, 5, 10, 20, 50 and 100 μM concentrations; or with pervanadate for 10 min at 0, 1, 3, 10, 30 and 100 μM concentrations. At the endpoint, 9 ml of cold HANKS buffer was added, and cells were cooled in an ice bath. Cells were pelleted at low speed at 4 °C, and then transferred to 1.5 ml tubes in 1 ml of HANKS. Cells were pelleted and resuspended in 300 μl of 40 mM Tris, pH 8.0, 0.5% lithium dodecyl sulfate (LiDS) and the sample heated to 90 °C for 5 min. Protein concentration was estimated using the BCA assay. Samples were reduced using 5 mM TCEP, alkylated with 15 mM iodoacetamide, and digested overnight with trypsin at 37 °C. For phosphotyrosine enrichment, 1 mg of protein digest was passed through a YM-10 cartridge (Millipore) to remove excess trypsin, and a Detergent Removal Column (Pierce) to remove LiDS. Samples were brought up to a 1X IAP buffer solution (50 mM MOPS (pH 7.2), 10 mM Na_2_HPO_4_, 50 mM NaCl). 4G10 anti-phosphotyrosine-agarose conjugate (Millipore) was washed with IAP buffer and 50 μg was added to each sample followed by incubation overnight at 4 °C with end-over-end rotation. The 4G10 beads were set aside after centrifugation and the supernatants transferred to new tubes containing washed PTMScan beads (20 μl slurry of P-Tyr-100 and P-Tyr-1000, Cell Signaling Technology) followed by incubation for 4 h at 4 °C with end-over-end rotation. Both sets of beads were sequentially washed several times with IAP buffer and then water. Peptides were eluted from the beads using 2% acetonitrile (ACN), 0.1% formic acid, and then dried for storage. For phosphopeptide enrichment using titanium dioxide, 1 mg of digested protein was resuspended in a saturated glutamic acid solution containing 65% ACN, 2% trifluoroacetic acid (TFA). 10 mg of titanium dioxide beads was added (GL Sciences) followed by end-over-end rotation for 20 min at RT. After a series of washes, the phosphopeptides were eluted from the beads stepwise using a gradient of NH_4_OH from 300 mM to 1 M, and 60% ACN. Elutions were pooled and acidified using formic acid (1.3% final), and dried for storage.

### Liquid chromatography – Mass spectrometry

Peptides were resuspended in 5% ACN, 0.1% formic acid prior to reverse phase chromatographic separation using an EASY nLC-1000 UHPLC system (Thermo). For SYK immunoprecipitation experiments, peptides were analyzed with an Orbitrap Fusion mass spectrometer (Thermo). MS1 scan details include 1.2 × 10^5^ resolution, 4 × 10^4^ AGC target, 350–1600 m/z scan range; MS2 scan details include CID fragmentation and ion trap detection, an AGC target of 1 × 10^4^, and 35% collision energy. Samples derived from phosphopeptide enrichment experiments were analyzed on a Q Exactive mass spectrometer (Thermo). MS1 scan details include 7 × 10^4^ resolution, 3 × 10^6^ AGC target, 350–1800 m/z scan range; MS2 scan details include HCD fragmentation and Orbitrap detection, an AGC target of 2 × 10^5^, 1.75 × 10^4^ resolution and 30% normalized collision energy.

### Peptide assignments

For phosphopeptide enrichment, combined peak lists for each treatment group were generated using Proteome Discoverer (Thermo, version 1.4) and MS2 data were scored using Mascot (Matrix Science; version 2.4.0) and Sequest HT with the following parameters: static modification of C (carbamidomethylation, +57); variable modifications of NQ (deamidation, +1), M (oxidation, +16), STY (phosphorylation, +80); 10 ppm and 0.02 Da parent and fragment ion tolerances, respectively; up to 2 missed tryptic cleavages; uniprot *Mus musculus* protein database (16,670 entries, downloaded on 2014-06-24). Results were imported into Scaffold (Proteome Software, ver 4.1) where spectra were scored with X!Tandem (ver 2010.12.01.1) with additional variable modifications of peptide N-terminal dehydration (−18) and acetylation (+42). Peptide identification probabilities were estimated using the Peptide Prophet algorithm and were considered to be a positive hit if they scored ≤1% false discovery rate (FDR). A similar workflow was utilized for the anti-SYK immunoprecipitation experiments, except with a 0.6 Da fragment tolerance. Phosphorylation localization probabilities were scored with the Ascore algorithm using Scaffold PTM software (Proteome Software, ver 3.0). Site localization was confirmed above a 95% probability threshold.

### Multiple reaction monitoring

For each sample, the area of the gel lane where SYK was pre-determined to localize, between 60 and 90 kDa, was excised. Gel slices were reduced with 5 mM TCEP, alkylated with 15 mM iodoacetamide and in-gel digested with 0.04 μg trypsin (Promega). Eluted peptides were resuspended in 5% ACN, 0.1% formic acid, and 0.005% TFA and separated by reverse phase chromatography. Samples were analyzed on a TSQ Vantage triple quadrupole mass spectrometer (Thermo). Instrument settings included a FWHM of 0.7 and cycle time of 1.75 s. Method optimization was performed to determine i) the transitions which gave the highest signal, ii) the optimal collision energy for each transition, and iii) the peptide retention times. 27 transitions were monitored for 4 min each within a 35 min gradient. Data was analyzed using Skyline software (MacCoss lab, University of Washington, version 2.5). Peak areas for each transition were integrated, averaged for 3 biological replicates, and then normalized against an unphosphorylated control SYK peptide to control for changes in SYK protein abundance. The untreated samples were then normalized to a value of 1 and the sample groups were adjusted accordingly. Statistical analyses were performed using two-tailed T-tests assuming unequal variances.

## Results

### Phosphorylation profile of murine SYK

The two main goals of this work are to determine i) all the potential phosphosites of murine SYK, and ii) how the phosphorylation status of these sites are altered in response to mercury exposure. WEHI-231 is a B cell lymphoma cell line that is commonly used to study immature B lymphocytes since it readily undergoes apoptosis in response to BCR signaling [9]. Phosphosite determination was made for SYK in WEHI protein digests by utilizing LC-MS/MS on peptides after anti-SYK or -phosphotyrosine immunoprecipitations, or after phosphopeptide enrichment using titanium dioxide chromatography.

**Fig. 1:**
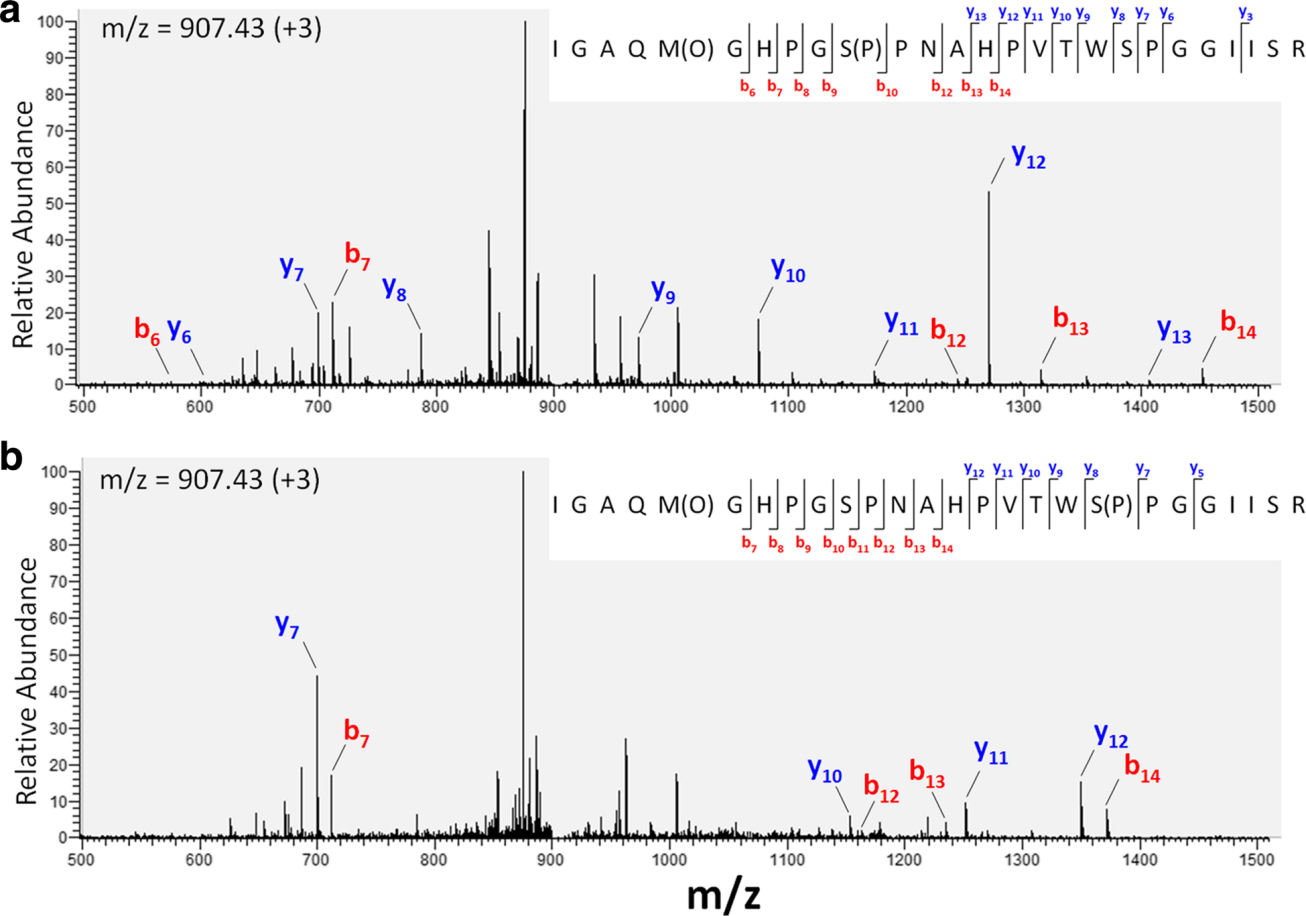
Representative spectral identification to unambiguously localize phosphosites. Phosphopeptides were enriched from WEHI protein digests and analyzed by LC-MS/MS. MS2 spectra are shown for phosphosite identifications of S270 (**a**) and S279 (**b**). Not all identified fragments are depicted. An additional oxidation modification of methionine at position 265 was also observed which may be an artifact of events occurring during peptide preparation. The *b*-ion series, in which the charge is localized to the N-terminal, is shown in red, and the *y*-ion series, where the charge is localized to the C-terminal, is shown in blue. Vertical lines in the inset represent cleavage sites after fragmentation within the mass spectrometer

Peptides can possess multiple serine, threonine and/or tyrosine residues, therefore localization of phosphorylated residues is predicated on the internal cleavage pattern after isolation and fragmentation within the mass spectrometer. For collision-induced dissociation (CID) or higher-energy collisional dissociation (HCD) cleavage reactions, this is determined by the overlapping *b*- and *y*-ion series. For example, spectra utilized to identify phosphorylated S270 and S279 are shown in Fig. 1 for the peptide 261-IGAQMGHPGSPNAHPVTWSPGGIISR-286. Note that *b*_12_, *b*_13_ and *b*_14_ are shifted to the right when S270 (Serine^270^) is phosphorylated in A, whereas *y*_10_, *y*_11_ and *y*_12_ are shifted to the right when S279 is phosphorylated in B. These fragmentation ions, or ‘transitions’, are of prime importance for accurate quantitation of phosphosites by MRM.

**Fig. 2:**
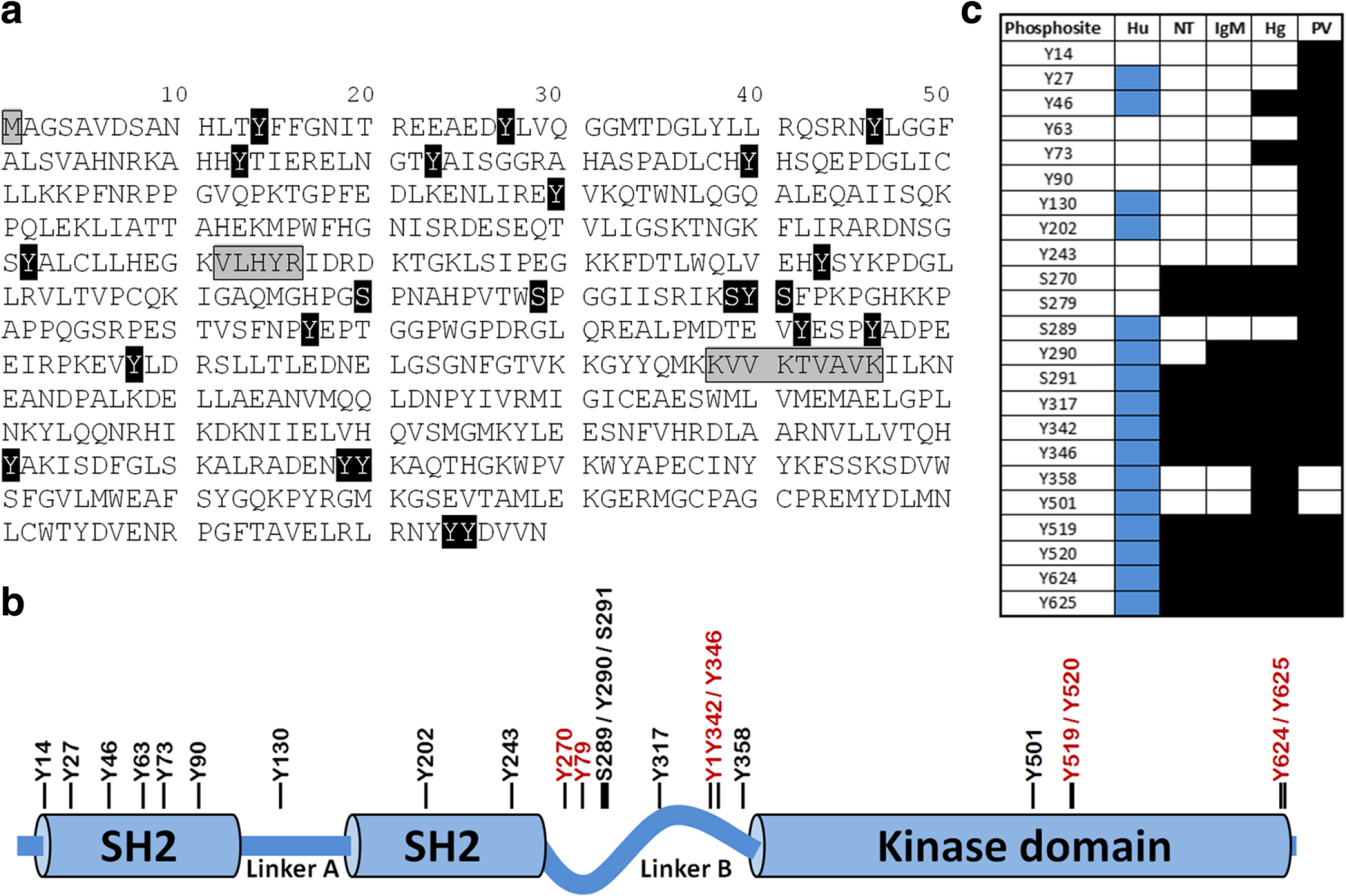
Phosphosite identification within tyrosine-protein kinase SYK. (**a**) Sequence coverage of murine SYK. Residues which were not sequenced are highlighted in *grey*, and phosphorylated residues are highlighted in *black*. MS2 spectral identification information can be found in the Additional files. The localization of phosphosites relative to protein tertiary structure are shown in (**b**). Phosphosites which were subsequently utilized for MRM quantitation are shown in red font. (**c**) Assignment of phosphosites to treatment classes. *Blue shaded boxes* represent phosphosites that have been identified in homologous sequences of human (Hu) recombinant SYK [34]. Black shaded boxes indicate that a spectral determination was positively identified for the indicated phosphosite and treatment regimen at any dosage tested. NT, not treated; IgM, activating anti-IgM antibody; Hg, mercury; PV, pervanadate

Based upon published genomic data, 96.7% of SYK amino acid residues were identified and sequenced in our experiments (Fig. 2a and Additional file 1). The only potential phosphosites not sequenced were Y215 and T392. In total, when results from all experimental conditions are included, 23 of the potential phosphosites were positively determined to be phosphorylated with high localization probability (Fig. 2 and Additional file 2). This included 4 phosphoserine residues in the central portion of SYK, and the remainder as phosphotyrosines along the length of the entire sequence. Phosphorylation of threonine residues were not identified by this unbiased approach in which site specific probability was estimated to be ≥95%.

Significantly, the profile of phosphorylated SYK residues depends upon the external cell environment. When the source of the phosphopeptides are considered according to cell treatment (Fig. 2c), it is evident that the distribution is not randomly dispersed for phosphosites that are observed before and after BCR activation. For example, after BCR activation with anti-IgM, we find several sites which are phosphorylated within the Linker B region and the C-terminal portion of the kinase domain, while in control cells we find no evidence that these sites are phosphorylated. On the other hand, mercury treatment led to identification of 4 phosphosites not observed in controls or the anti-IgM activated group, In this case Y46, Y358 and Y501 identifications were only observed at the highest and very toxic mercury concentration (100 μM), and with only 1 or 2 spectral identifications for each site (Additional file 3). In contrast, Y73 had dozens of spectral identifications at either 50 or 100 μM Hg^2+^, indicating it was more susceptible to modification following mercury exposure.

Pervanadate was included in these experiments since this compound effectively inhibits protein tyrosine phosphatases, particularly PTP1B, by irreversible oxidation of the active-site cysteine residues to cysteic acid [10]. Therefore, pervanadate would unmask weakly phosphorylated tyrosine residues in control cells by shifting the dynamic equilibrium of a phosphosite to the phosphorylated form. By spectral counting, pervanadate increased the number of phosphosites identified (Fig. 2c), and accounted for about 62% of all phosphopeptides identified (Additional file 3). The majority of the phosphotyrosine sites uncovered by pervanadate treatment (9 of 10) occur within SH2 domains which are critical for protein-protein interaction.

### Monitoring of the mercury dose response of select SYK phosphosites

**Fig. 3:**
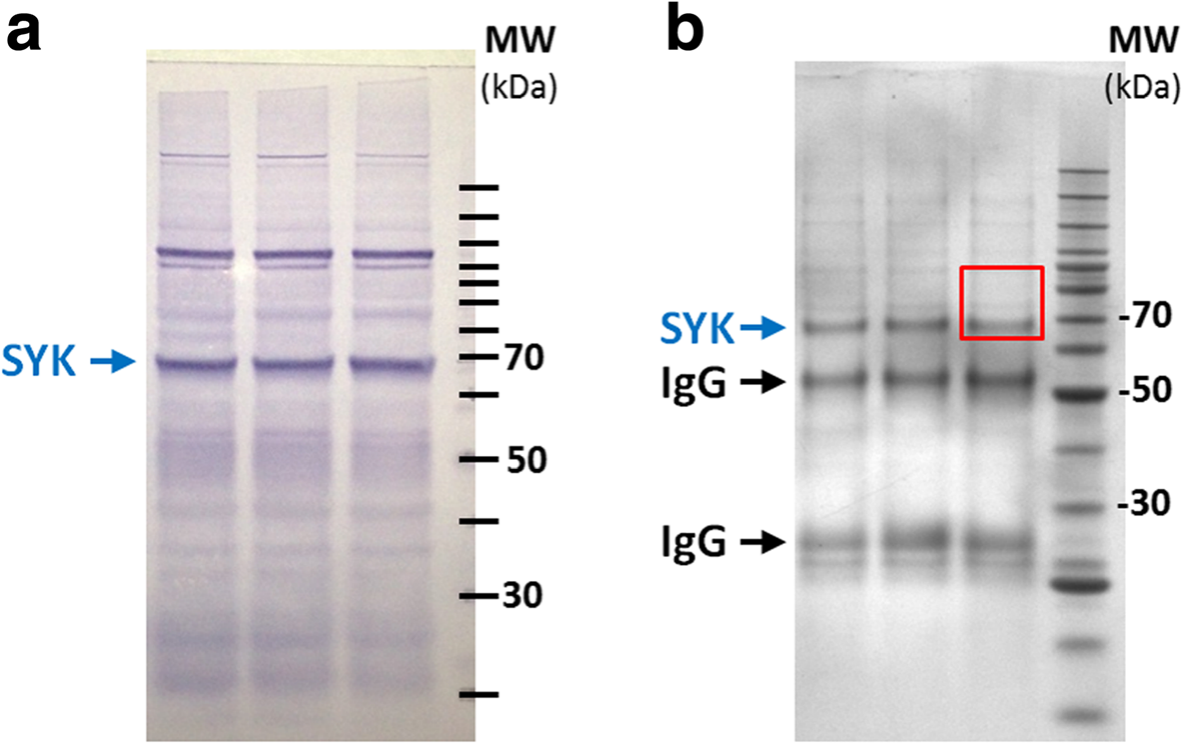
Enrichment of SYK for relative quantitative measurement of phosphorylation. (**a**), In triplicate samples, 25 μg of WEHI cell extract was separated by SDS-PAGE and SYK mobility was visualized by colorimetric western blotting. A major band close to the predicted molecular weight of SYK (71 kDa) is indicated by a *blue arrow*. Location of a molecular weight (MW) ladder is indicated on the right, which occurs in 10 kDa increments. (**b**), SYK was immunoprecipitated as described in the Material & Methods. Samples were separated by SDS-PAGE and the gel stained with Coomassie Blue. Major bands corresponding to SYK as well as heavy and light IgG chains are indicated. For multiple reaction monitoring (MRM) experiments, areas of the gel corresponding to the *red box* were excised

To achieve greater sensitivity for profiling changes in SYK phosphorylation status due to mercury exposure, an approach was undertaken which we previously utilized to characterize the tyrosine-protein kinase LYN [8]. Sensitivity of mass spectrometry is greatly enhanced by reducing interfering background ions. To this end, after mercury exposures, SYK was first immunoprecipitated from cell extracts, and then the samples were further purified by excising defined areas from SDS-PAGE gels (Fig. 3). Peptides were prepared from gel slices and analyzed by MRM. The advantage of this approach over antibody-based methods is the specificity that is attained for protein phosphosites through selection of unique transition ions.

**Fig. 4:**
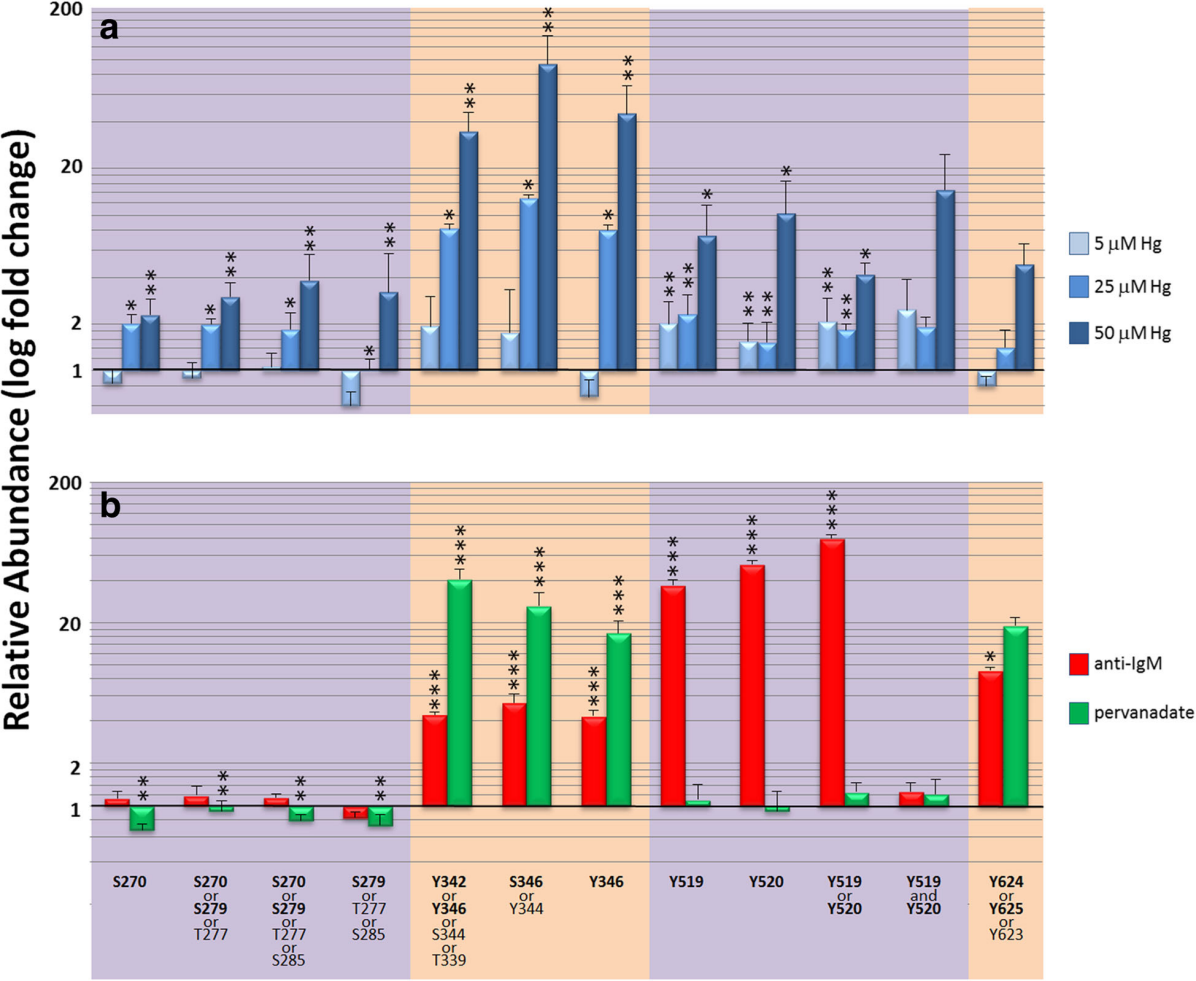
Quantitation of phosphorylation status within cluster regions of SYK. Phosphorylation of specific sites was quantified by MRM, and then standardized to a non-phosphorylated peptide (to control for SYK protein abundance) and normalized to untreated cells (to a value of 1.00). Results represent the average of 3 separate experiments, and each experiment was performed with biological triplicates. Error bars represent the Standard Error of the Mean (SEM). Phosphosites with localization probabilities ≥99% are in bold. (**a**) Monitoring of changes in phosphorylation status after exposure to 5, 25 or 50 μM Hg^2+^ for 10 min. (**b**) Monitoring of changes in phosphorylation status after exposure to either activating antibody (anti-IgM; 100 μg/10^6^ cells for 2 min; *red bars*) or pervanadate (10 μM for 10 min; *green bars*). Statistical analyses were performed on the following clusters: S270 or T277 or S279 or S285; T339 or Y342 or S344 or Y346; Y519 or Y520; Y519 and Y520; and Y623 or Y624 or Y625. * = *p* ≤ 0.1; ** = *p* ≤ 0.05; *** = *p* ≤ 0.005

Phosphopeptides which were associated with mercury exposure were tested to determine whether they were amenable to MRM analysis. For transitions to be successfully incorporated into MRM, the parent peptide should have a low charge state and good signal intensity, and fragmentation efficiency should be great enough to yield high signal intensity for the selected transition ions. Phosphosites from 4 clusters met these criteria: S270/S279 and Y342/Y346 clusters in the Linker B region; and Y519/Y520 and Y624/Y625 clusters in the kinase domain (Table 1 and Fig. 4). A probability-based localization algorithm (Ascore) indicated the unlikelihood of phosphorylation at T277, S285, T339, S344 and Y625 within these cluster regions. However it cannot be ruled out with 100% certainty that these sites are not phosphorylated based upon their location within the peptide sequence relative to the cleavage site so they are included in the results.

**Table 1:** Peptides and transition ions used for quantitative MRM analysis

Peptide	Phosphosite(s)	Parent m/z	Transition	Transition m/z	Collision Energy
VLTVPC[+57]QK	Control	472.765	b2	213.16	14
VLTVPC[+57]QK	Control	472.765	y4	532.255	17
VLTVPC[+57]QK	Control	472.765	y6	732.371	14
IGAQM[+16]GHPGS[+80]PNAHPVTWSPGGIISR	S270	907.43	y12	1269.695	37
IGAQM[+16]GHPGS[+80]PNAHPVTWSPGGIISR	S270 or T277 or S279	907.43	y7	699.415	38
IGAQM[+16]GHPGS[+80]PNAHPVTWSPGGIISR	S270 or T277 or S279 or S285	907.43	b7	711.324	36
IGAQM[+16]GHPGSPNAHPVTWS[+80]PGGIISR	T288 or S279 or S285	907.43	y10-98	1055.563	41
IGAQM[+16]GHPGSPNAHPVTWS[+80]PGGIISR	T277 or S279 or S285	907.43	y12-98	1251.684	37
EALPM[+16]DTEVY[+80]ESPYADPEEIRPK	T339 or Y342 or S344 or Y346	925.742	b2	201.087	38
EALPM[+16]DTEVY[+80]ESPYADPEEIRPK	T339 or Y342 or S344 or Y346	925.742	y4	513.351	38
EALPM[+16]DTEVY[+80]ESPYADPEEIRPK	T339 or Y342 or S344 or Y346	925.742	y7	868.489	42
EALPM[+16]DTEVY[+80]ESPYADPEEIRPK	T339 or Y342 or S344 or Y346	925.742	y20	1231.527	38
EALPM[+16]DTEVYESPY[+80]ADPEEIRPK	S344 or Y346	925.742	y12	741.337	38
EALPM[+16]DTEVYESPY[+80]ADPEEIRPK	Y346	925.742	y10	649.295	39
EALPM[+16]DTEVYESPY[+80]ADPEEIRPK	Y346	925.742	y11	697.821	38
ALRADENY[+80]YK	Y519	661.792	y2	310.176	28
ALRADENY[+80]YK	Y519 or Y520	661.792	b5	527.294	23
ALRADENY[+80]YK	Y519 or Y520	661.792	b6	656.336	23
ALRADENYY[+80]K	Y519 or Y520	661.792	y3	277.107	24
ALRADENYY[+80]K	Y519 or Y520	661.792	y3	553.206	20
ALRADENYY[+80.0]K	Y520	661.792	y2	390.142	26
ALRADENY[+80.0]Y[+80.0]K	Y519 + Y520	701.776	b3	341.23	24
ALRADENY[+80.0]Y[+80.0]K	Y519 + Y520	701.776	b5	527.294	20
ALRADENY[+80.0]Y[+80.0]K	Y519 + Y520	701.776	b6	656.336	20
LRNYY[+80.0]YDVVN	Y623 or Y624 or Y625	699.808	b7	1068.419	24
LRNYY[+80.0]YDVVN	Y623 or Y624 or Y625	699.808	b8	1167.487	21
LRNYY[+80.0]YDVVN	Y623 or Y624 or Y625	699.808	y2	232.129	24

Each transition was optimized for collision energy (Table 1) and chromatographic retention time and then a method was developed to monitor the signal intensity of all 27 transitions within a single LC-MS/MS run. Samples were treated in triplicate for each dosage within an experiment, and the final data represent the average of 3 separate experiments (Fig. 4). The results suggest that mercury can increase the phosphorylation of selected SYK phosphosites in a dose-dependent manner. This finding was most evident for phosphorylation of the Y342/Y346 cluster, which was induced to levels which were up to 13-fold higher with 25 μM mercury and 94-fold higher with 50 μM compared to mock-treated cell cultures. For all the sites, 50 μM mercury induced at least a 2-fold increase in phosphorylation, whereas 5 and 25 μM had no or more modest effects.

WEHI cell cultures were also exposed to either an anti-IgM antibody or pervanadate as a reference for SYK activation via BCR activation and phosphatase inhibition, respectively. Neither of the agents at the dosages and times tested activated phosphosites in the S270/S270 cluster, but both agents activated the Y342/Y346 and Y624/Y625 clusters (Fig. 4b). Antibody exposure led to increased phosphorylation of Y519 or Y520 (up to 79-fold higher relative to control), whereas pervanadate had little effect on this cluster. These results demonstrate that exogenous agents can impart unique phosphorylation profiles on SYK, and that these signatures can be monitored using sensitive and selective mass spectrometry-based techniques.

## Discussion

In addition to acting as a potent neurotoxin, mercury is known to have effects on autoimmunity. In autoimmune-prone murine models, exposure to sub-neurotoxic concentrations of mercury increases susceptibility to autoimmune disease [11–16]. Only a limited number of small-scale epidemiological studies have examined human subjects. For example, gold mining activity in Brazil was found to be associated with increased mercury exposure and higher production of proinflammatory cytokines and autoantibodies in workers [17, 18]. More recently it has been shown that blood mercury levels in women are correlated with circulating autoantibodies to double stranded DNA [19].

Genetic factors have a role in susceptibility to mercury-induced autoimmune disease in animal models, and are likely to be a factor in human susceptibility as well. However exposure to mercury alone is generally insufficient to induce autoimmune disease in humans. A more likely scenario is that mercury exacerbates a host response to a microbial or other environmental trigger. For instance, coxsackievirus B3 (CVB3) infection damages heart tissue and is thought to induce autoimmunity by exposure of cardiac myosin, an intracellular protein, to the immune system [20, 21]. When BALB/c mice are inoculated with extracts from heart tissue with infectious CVB3, mice develop autoimmune myocarditis similar to that observed in humans [22, 23], and pretreatment with low-dose mercury greatly increases the severity and frequency of disease [24].

In this report we wanted to focus on immature B cells, as it is negative selection driven by signaling through the BCR of immature B cells, rather than mature B cells, that is a major determinant that shapes the immune repertoire and establishes tolerance to self-antigens. However a complicating factor in the analysis of immature B cells is that they primarily reside in the spleen and so are not present in the peripheral circulation, making the collection of primary human immature B cells difficult. In order to overcome this challenge we have utilized the WEHI line because it is perhaps the most studied model of immature B cells. In WEHI cells, as in immature B cells, strong BCR signaling leads to cell death rather than proliferation as is found in mature B cells. Furthermore, although WEHI is of mouse origin, to date virtually all findings with respect to the WEHI BCR have been found to correlate well with human B cell BCRs [9].

We also wanted our findings to be relevant to actual environmental mercury exposures. Mercury levels have been reported for autopsy samples from Greenland [25], Korea [26], Norway [27] and Poland [28]. These data were calculated as μg Hg per g of tissue. If we make the assumption that the tissues have a density similar to water, then the concentration of Hg in these tissues ranged from 0.02 – 0.80 μM in spleen, 0.10 – 2.69 μM in liver, and 0.20 – 6.98 μM in kidney. We observed significant changes in SYK phosphorylation profiles with 10 min exposure to inorganic mercury in the range of 5 – 25 μM, which can conceivably be achieved in human spleen and other tissues during specific periods over a lifetime of dietary and environmental Hg exposure.

In vivo models suggest that pathogens can trigger an autoimmune response by either revealing novel self-antigens to the immune system [24, 29], or possibly by activating anergic B cells [4, 30], but the mechanism by which mercury amplifies this effect is not clear. We hypothesize that mercury recalibrates the threshold of B cell responsiveness to self-antigens by interfering with BCR signaling. Previously we have shown that mercury interferes with BCR signal transduction upstream of ERK [31]. Subsequent to the initiation of BCR signaling, and upstream of ERK, the tyrosine kinases LYN and SYK are the first proteins recruited to the developing B cell signalosome [32]. Importantly, by using flow cytometry methodology that specifically targeted pY346 of SYK, we have shown that pretreatment with Hg led to enhanced and prolonged SYK phosphorylation following BCR stimulation [33].

In the studies described here we have mapped the phosphorylation profile of SYK in mouse B cells, and demonstrated that mercury exposure can alter baseline phosphorylation levels of multiple cluster regions in a dose-dependent manner. It is likely that mercury induced phosphorylation of SYK interferes with SYK functionality and contributes to mercury immunotoxicity. 32 phosphosites have been reported for human recombinant SYK protein expressed in the chicken B cell line DT40 [34]. In this study we have identified 23 endogenous SYK phosphosites with high probability in immortalized murine B cell cultures, 14 of which mapped to homologous sequences of human SYK (Fig. 2c). Conservation of phosphorylation motifs across species would suggest a functional importance of these domains. Conversely a lack of conservation across species would suggest a lack of functional significance. This may explain why among the cluster regions we profiled in detail, only S270 and S279 residues were not conserved in human SYK, and only these two sites were unresponsive to either anti-IgM or pervanadate treatment (Fig. 4b).

Protein interaction studies and mutation analyses on SYK and the structurally similar homologue ZAP-70 have led to a better understanding of signal propagation by SYK in B cells. [35]. Activation of the BCR and Fc receptors leads to phosphorylation of ITAMs (immunoreceptor tyrosine-based activation motifs) within the cytoplasmic tails of receptor or coreceptor subunits. The two SH2 domains of SYK bind to phosphorylated ITAMs which results in an ‘open’ conformational change in SYK that exposes the linker regions to kinases. For instance, the Y342/Y346 cluster is phosphorylated by LYN leading to binding of Vav-1 and PLC-γ with an affinity that is dependent upon whether the sites are individually or dually phosphorylated [36–38]. Previously we have shown that 5 μM Hg does not affect PLC-γ phosphorylation [31], and its not until a very high concentration (250 μM) that we observe an increase [39]. However, low doses of Hg can affect the temporal dynamics of BCR signalosome formation and may alter signaling by outcomes that are yet to be determined. For example, we have shown that pretreatment with Hg led to a faster on/off interaction between SYK and PLC-γ following BCR stimulation [31]. Our results confirm that anti-IgM stimulation of the BCR leads to increased phosphorylation of either Y342 or Y346. We now report that the phosphorylation of these sites are highly sensitive to mercury exposure, suggesting that mercury interferes with BCR signal transduction at the level of SYK.

Binding of SYK to the activated BCR complex also reverses the closed, autoinhibitory conformation of the C-terminal kinase domain. SYK then trans-autophosphosphorylates the activation loop tyrosines at Y519 and Y520. This cluster region is not essential for kinase activity, but mutation of either site was found to abrogate SYK signaling in human or rat cells [40, 41]. We now have found that in murine B cell cultures, the highest fold changes in phosphorylation after anti-IgM stimulation occurred at singly phosphorylated Y519 or Y520, whereas mercury induced phosphorylation of this cluster in either single or double configurations.

There are 3 linked conserved tyrosine residues near the C-terminal of SYK (Y623, Y624 and Y625 in mouse). We found that Y624 and Y625 residues were endogenously singly or doubly phosphorylated in WEHI cell cultures. This data is in agreement with studies showing that substitution of tyrosine with phenylalanine affects SYK kinase activity and autophosphorylation when it occurs at positions 624 and 625 but not 623 [42]. Furthermore, ZAP-70 has only 2 terminally linked tyrosines in this region and therefore the third tyrosine is not highly conserved across protein family members. Our data show that the C-terminal tyrosine cluster was sensitive to anti-IgM and pervanadate treatment, but relatively insensitive to mercury, so that mercury does not affect all functional SYK phosphosites uniformly, but is selective.

## Conclusions

Our data on the effect of mercury exposure on SYK, taken together with our earlier work on LYN, suggests that exposure to moderate levels of inorganic mercury can disrupt BCR signaling by interfering with phosphorylation dependent kinase activation and localization. This work in particular implicates mercury interference with the BCR dependent phosphorylation of specific SYK phosphosites in the development of mercury dependent immunotoxicity.

## Additional files

Additional file 1: SYK protein sequencing coverage. These data represent results from a single anti-SYK immunoprecipitation experiment with WEHI cell extracts. The sample was digested with trypsin and analyzed by LC-MS/MS. MS2 spectral identifications were scored with Mascot and X!Tandem algorithms against forward and scrambled murine protein databases. Positive identifications were assigned below a 1% FDR threshold. (PDF 254 kb)

Additional file 2: Consensus SYK phosphorylation sites. Singly, doubly or triply phosphorylated peptides were scored for peptide identification probability and modification site localization probability. Cutoff for accepted phosphosites was a localization probability of ≥95%. Data represent top scoring peptides. (PDF 219 kb)

Additional file 3: Identification of SYK phosphopeptides. MS2 phosphopeptide identification information was collated from various studies on WEHI cell extracts, including anti-SYK and anti-pY immunoprecipitation experiments and titanium dioxide enrichments. Peptides were filtered to a ≤ 1% false discovery rate. (PDF 2499 kb)

## Abbreviations

ACN: Acetonitrile
BCR: B cell receptor
CID: Collision-induced dissociation
CVB3: Coxsackievirus B3
HCD: Higher-energy collisional dissociation
ITAM: Immunoreceptor tyrosine-based activation motif
LC-MS/MS: Liquid chromatography – tandem mass spectrometry
MRM: Multiple reaction monitoring
NT: Not treated
PV: Pervanadate
TFA: Trifluoroacetic acid
