# Supplementary material for: Mercury alters endogenous phosphorylation profiles of SYK in murine B cells
Source: BMC Immunol. 2017 Jul 17;18:37. doi: 10.1186/s12865-017-0221-0 (PMC5514489; doi:10.1186/s12865-017-0221-0)
Supplement: Supplementary file 1 — SYK protein sequencing coverage. These data represent results from a single anti-SYK immunoprecipitation experiment with WEHI cell extracts. The sample was digested with trypsin and analyzed by LC-MS/MS. MS2 spectral identifications were scored with Mascot and X!Tandem algorithms against forward and scrambled murine protein databases. Positive identifications were assigned below a 1% FDR threshold. (PDF 254 kb) [file 12865_2017_221_MOESM1_ESM.pdf]

Additional File 1. SYK protein sequencing coverage. These data represent results from a single anti-SYK immunoprecipitation experiment with WEHI cell extracts. The sample was digested with trypsin and analyzed by LC-MS/MS. MS2 spectral identifications were scored with Mascot and X!Tandem algorithms against forward and scrambled murine protein databases. Positive identifications were assigned below a 1% FDR threshold.

Peptide Sequence	Start	Stop	Best Peptide ID Prob.	Best Sequest XCORR	Best Mascot Ion Score	Best X! Tandem -log(e) Score	Total IDs	z = +2	z = +3	z = +4	Calculated Mass (AMU)
AGSAVDSANHLTYFFGNITR	2	21	100%	nd	nd	7.57	18	4	14	0	2184.04
AGSAVDSANHLTYFFGNITREEAEDYLVQGGMTDGLYLLR	2	41	100%	nd	nd	8.42	18	0	6	12	4453.09
EEAEDYLVQGGMTDGLYLLR	22	41	100%	5.66	85.70	8.17	14	6	8	0	2270.06
QSRNYLGGFALSVAHNR	42	58	100%	4.28	35.00	1.74	3	0	2	1	1889.97
NYLGGFALSVAHNR	45	58	100%	4.36	107.62	12.00	10	8	2	0	1519.77
NYLGGFALSVAHNRK	45	59	100%	3.96	74.20	8.59	9	3	1	5	1646.88
YLGGFALSVAHNR	46	58	99%	nd	nd	3.26	1	0	1	0	1,403.74
KAHHYTIERELNGTYAISGGR	59	79	100%	4.28	37.90	8.72	15	0	6	9	2374.19
AHHYTIERELNGTYAISGGR	60	79	100%	3.75	34.50	5.25	9	2	4	3	2245.11
ELNGTYAISGGR	68	79	100%	3.83	54.26	3.49	13	13	0	0	1220.59
AHASPADLCHYHSQEPDGLICLLK	80	103	100%	4.92	25.20	1.92	12	0	2	10	2732.29
KPFNRPPGVQPK	104	115	100%	4.32	37.90	3.74	21	6	15	0	1364.78
TGPFEDLKENLIR	116	128	100%	3.72	63.48	9.29	20	8	12	0	1531.81
TGPFEDLKENLIREYVK	116	132	100%	3.55	33.16	11.00	13	1	10	2	2051.08
TGPFEDLKENLIREYVKQTWNLQGQALEQAIISQKPQLEK	116	155	100%	nd	nd	2.72	3	0	0	3	4683.48
EYVKQTWNLQGQALEQAIISQKPQLEK	129	155	100%	6.82	67.60	4.14	11	0	6	5	3152.67
QTWNLQGQALEQAIISQKPQLEK	133	155	100%	4.47	74.80	10.40	35	9	18	8	2651.42
LIATTAHEK	156	164	100%	2.38	44.90	1.92	3	3	0	0	983.55
LIATTAHEKMPWFHGNISR	156	174	100%	3.11	42.19	4.46	4	0	0	4	2,224.12
LIATTAHEKMPWFHGNISRDESEQTVLIGSK	156	186	100%	4.98	20.70	2.64	3	0	0	3	3511.76
MPWFHGNISR	165	174	99%	2.44	37.45	5.19	3	0	3	0	1260.59
MPWFHGNISRDESEQTVLIGSK	165	186	100%	4.43	25.10	2.89	12	0	4	8	2547.23
DESEQTVLIGSK	175	186	100%	3.12	65.90	5.74	5	5	0	0	1305.65
TNGKFLIR	187	194	100%	2.05	62.71	6.21	12	12	0	0	949.55
ARDNSGSYALcLLHEGK	195	211	100%	5.08	38.10	3.64	4	0	4	0	1890.91
IDRDKTGKLSIPEGK	217	231	100%	nd	nd	3.96	1	0	0	1	1,655.92
DKTGKLSIPEGK	220	231	100%	2.98	47.59	4.42	2	1	1	0	1272.72
TGKLSIPEGK	222	231	100%	2.72	26.00	3.43	2	2	0	0	1029.59
LSIPEGKKFDTLWQLVEHYSYKPDGLLR	225	252	100%	3.43	nd	nd	1	0	0	1	3332.77
KFDTLWQLVEHYSYKPDGLLR	232	252	100%	6.39	48.60	6.38	23	0	8	15	2608.36
FDTLWQLVEHYSYKPDGLLR	233	252	100%	5.26	52.80	2.92	6	0	4	2	2480.26
VLTVPCQK	253	260	100%	1.82	25.12	4.04	1	1	0	0	944.5006
IGAQMGHPGSPNAHPVTWSPGGIISR	261	286	100%	5.32	60.70	8.48	50	4	19	27	2640.31
IKSYSFPKPGHK	287	298	100%	3.95	44.10	4.43	5	2	3	0	1388.77
SYSFPKPGHK	289	298	100%	2.44	24.90	2.27	3	3	0	0	1147.59
SYSFPKPGHKKPAPPQGSRPESTVSFNPYEPTGGPWGPDR	289	328	100%	5.55	16.90	nd	1	0	0	1	4339.08
KPAPPQGSRPESTVSFNPYEPTGGPWGPDR	299	328	100%	7.41	69.10	9.21	27	0	14	13	3288.51
KPAPPQGSRPESTVSFNPYEPTGGPWGPDRGLQR	299	332	99%	5.96	41.20	3.57	7	0	0	7	3662.81
EALPMDTEVYESPYADPEEIRPK	333	355	100%	3.97	43.40	12.38	22	9	13	0	2695.24
EALPMDTEVYESPYADPEEIRPKEVYLDR	333	361	100%	4.43	70.30	3.96	6	0	4	2	3470.63
EVYLDRSLLTLEDNELGSGNFGTVK	356	380	100%	5.07	69.10	6.34	6	1	5	0	2769.39
EVYLDRSLLTLEDNELGSGNFGTVKK	356	381	100%	5.63	41.20	nd	2	0	2	0	2898.47
SLLTLEDNELGSGNFGTVK	362	380	100%	6.06	97.20	11.50	21	15	6	0	1994.99
SLLTLEDNELGSGNFGTVKK	362	381	100%	5.58	91.60	7.92	8	4	4	0	2122.10
SLLTLEDNELGSGNFGTVKKGYYQMK	362	387	100%	nd	23.70	4.01	1	0	1	0	2908.44
ILKNEANDPALKDELLAEANVMQQLDNPYIVR	397	428	100%	7.42	93.70	9.41	21	0	9	12	3638.87
NEANDPALKDELLAEANVMQQLDNPYIVR	400	428	100%	6.56	75.70	6.42	11	0	11	0	3284.61
MIGICEAESWMLVMEMAELGPLNK	429	452	99%	nd	26.20	nd	1	0	1	0	2816.27

MIGICEAESWMLVMEMAELGPLNKYLQQNR	429	458	100%	nd	90.10	4.37	2	0	2	0	3618.68
HIKDKNIIELVHQVSMGMK	459	477	100%	5.07	43.10	2.60	20	0	8	12	2252.19
DKNIIELVHQVSMGMK	462	477	100%	2.73	8.69	2.42	1	0	1	0	1873.95
NIIELVHQVSMGMK	464	477	100%	2.96	28.50	nd	1	1	0	0	1630.83
YLEESNFVHR	478	487	100%	3.18	61.01	5.47	9	4	5	0	1293.62
YLEESNFVHRDLAAR	478	492	100%	3.06	70.84	7.80	5	1	3	1	1819.91
NVLLVTQHYAK	493	503	100%	2.60	48.05	5.22	8	5	3	0	1285.73
NVLLVTQHYAKISDFGLSK	493	511	100%	3.41	24.10	3.21	3	0	3	0	2133.17
ISDFGLSK	504	511	100%	1.73	54.03	2.72	1	1	0	0	865.4546
ALRADENYYK	512	521	100%	3.61	41.00	4.01	9	4	5	0	1242.61
ALRADENYYKAQTHGK	512	527	100%	2.82	10.98	1.05	1	0	0	1	1,863.93
ADENYYK	515	521	99%	2.82	38.70	2.66	3	3	0	0	902.39
AQTHGKWPVK	522	531	100%	3.19	43.20	4.82	4	4	0	0	1151.63
AQTHGKWPVKWYAPEcINYYK	522	542	100%	4.41	21.50	0.68	3	0	2	1	2639.29
WYAPECINYYK	532	542	100%	2.28	27.00	1.80	2	2	0	0	1506.67
FSSKSDVWSFGVLMWEAFSYGQKPYR	543	568	100%	5.83	39.00	1.05	4	0	0	4	3119.46
SDVWSFGVLMWEAFSYGQKPYR	547	568	100%	4.64	49.90	3.92	7	3	4	0	2670.23
GMKGSEVTAMLEKGER	569	584	100%	3.88	35.70	1.40	4	0	4	0	1754.84
GSEVTAMLEK	572	581	100%	2.96	35.30	3.89	8	8	0	0	1080.52
GSEVTAMLEKGER	572	584	100%	4.35	65.81	8.14	23	14	9	0	1422.69
MGCPAGCPR	585	593	100%	2.90	47.50	5.42	2	2	0	0	1021.40
EMYDLMNLCWTYDVENRPGFTAVELR	594	619	100%	4.72	83.90	4.23	7	0	7	0	3254.46
LRNYYYDVVN	620	629	100%	2.85	42.64	1.42	4	4	0	0	1318.64
NYYYDVVN	622	629	100%	2.36	47.00	1.80	4	4	0	0	1049.46
