# Supplementary material for: Mercury alters endogenous phosphorylation profiles of SYK in murine B cells
Source: BMC Immunol. 2017 Jul 17;18:37. doi: 10.1186/s12865-017-0221-0 (PMC5514489; doi:10.1186/s12865-017-0221-0)
Supplement: Supplementary file 2 — Consensus SYK phosphorylation sites. Singly, doubly or triply phosphorylated peptides were scored for peptide identification probability and modification site localization probability. Cutoff for accepted phosphosites was a localization probability of ≥95%. Data represent top scoring peptides. (PDF 219 kb) [file 12865_2017_221_MOESM2_ESM.pdf]

Additional File 2. Consensus SYK phosphorylation sites. Singly, doubly or triply phosphorylated peptides were scored for peptide identification probability and modification site loc alization probability. Cutoff for accepted phosphosites was a localization probability of ≥ 95%. Data represent top scoring peptides.

Phosphosite	Peptide Sequence[1]	Start	Stop	Variable Modifications	Fixed Modifications[2]	Localization Probability	Ascore	Peptide Probability[3]	Mascot Ion Score	Sequest XCORR	X!Tandem Expect	z	Actual Mass	Observed Mass	Delta AMU
Y14	aGSAVDSANHLTyFFGNITR	2	21	n-term Acetyl, Y13 Phospho		100%, 100%	1000, 27.96	100%	nd	nd	6.40E-12	3	2262.02	755.01	0.00596
Y27	EEAEDyLVQGGMTDGLYLLR	22	41	Y6 Phospho		100%	168.83	100%	55.79	2.996	1.70E-13	3	2351.05	784.69	0.00547
Y46	NyLGGFALSVAHNR	45	58	Y2 Phospho		100%	294.8	100%	94.26	3.784	8.30E-13	2	1597.74	799.88	0.00208
Y63	KAHHyTIER	59	67	Y5 Phospho		100%	27.96	100%	37.72	2.033	4.30E-04	3	1233.56	412.20	-0.00149
Y73	ELNGTyAISGGR	68	79	Y6 Phospho		100%	30.97	100%	74.69	1.809	4.00E-06	2	1316.58	659.30	0.00070
Y90	AHASPADLcHyHSQEPDGLIcLLK	80	103	Y11 Phospho	C9 IAA, C21 IAA	98%	17.21	100%	30.64	3.552	3.80E-06	4	2811.26	703.82	0.00594
Y130	TGPFEDLKENLIREyVK	116	132	Y15 Phospho		100%	314.35	100%	50.09	2.68	6.30E-13	3	2130.04	711.02	0.00106
Y202	ARDNSGSyALcLLHEGK	195	211	Y8 Phospho	C11 IAA	100%	24.95	99.99%	18.29	1.423	5.50E-04	3	1969.87	657.63	-0.00093
Y243	KFDTLWQLVEHySYKPDGLLR	232	252	Y12 Phospho		100%	26.2	100%	32.19	3.735	1.30E-08	4	2687.32	672.84	0.00848
S270	IGAQMGHPGsPNAHPVTWSPGGIISR	261	286	S10 Phospho		100%	231.99	100%	44.58	6.892	2.10E-09	3	2703.27	902.10	0.00022
S279	IGAQMGHPGSPNAHPVTWsPGGIISR	261	286	S19 Phospho		99%	21.95	99.83%	19.26	3.34	0.0012	3	2703.27	902.10	-0.00656
S270, S279	IGAQMGHPGsPNAHPVTWsPGGIISR	261	286	S10 Phospho, S19 Phospho		100%, 98%	28.35, 17.99	99.25%	25.84	3.157	0.0083	3	2783.24	928.7526	-0.00469
Y290	IKSySFPKPGHK	287	298	Y4 Phospho		100%	24.95	100%	45.32	3.081	4.40E-07	2	1467.73	734.87	-0.00104
S290, Y291	IKSysFPKPGHK	287	298	Y4 Phospho, S5 Phospho		100%, 99%	24.95, 21.95	100%	38.9	3.584	0.0017	2	1547.70	774.86	0.00297
S289, Y290, S291	IKsysFPKPGHK	287	298	S3 Phospho, Y4 Phospho, S5 Phospho		100%, 100%, 100%	1000, 1000, 1000	100%	18.71	2.178	0.019	3	1627.67	543.56	0.00513
Y317	KPAPPQGSRPESTVSFNPyEPTGGPWGPDR	299	328	Y19 Phospho		100%	96.95	100%	36.53	5.145	8.80E-16	3	3287.52	1096.85	0.01340
Y342	EALPMDTEVyESPYADPEEIRPK	333	355	Y10 Phospho		100%	77.91	100%	65.28	4.32	3.50E-11	3	2758.22	920.41	0.01010
Y346	EALPMDTEVYESPyADPEEIRPK	333	355	Y14 Phospho		100%	53.49	100%	50.86	nd	3.00E-11	3	2758.22	920.41	0.01430
Y342, Y346	EALPMDTEVyESPyADPEEIRPK	333	355	Y10 Phospho, Y14 Phospho		100%, 100%	23.20, 53.49	100%	41.14	3.265	7.90E-10	3	2838.21	947.08	0.03960
Y358	EVyLDR	356	361	Y3 Phospho		100%	1,000.00	90%	23.36	1.592	0.0091	2	873.38	437.70	0.01320
Y501	NVLLVTQHyAK	493	503	Y9 Phospho		99%	20.19	99.99%	25.17	2.971	0.0031	2	1364.69	683.35	0.00427
Y519	ALRADENyYK	512	521	Y8 Phospho		100%	58.42	100%	37.37	3.131	2.90E-10	2	1321.57	661.79	0.00245
Y520	ALRADENYyK	512	521	Y9 Phospho		100%	51.06	100%	40.77	2.897	4.40E-11	2	1321.57	661.79	0.00294
Y519, Y520	ALRADENyyK	512	521	Y8 Phospho, Y9 Phospho		100%, 100%	1000, 1000	100%	44.13	2.529	7.00E-11	2	1401.54	701.78	0.00377
Y624	LRNYyYDVVN	620	629	Y5 Phospho		100%	30.97	100%	39.56	2.619	1.40E-04	2	1397.60	699.81	0.00006
Y625	LRNYYyDVVN	620	629	Y6 Phospho		100%	27.96	100%	45.51	1.857	4.60E-04	2	1397.60	699.81	0.00018
Y624, Y625	LRNYyyDVVN	620	629	Y5 Phospho, Y6 Phospho		100%, 100%	30.97, 60.18	99.84%	13.74	nd	0.006	2	1477.59	739.80	0.02210

nd, not determined

[1]Lower case amino acid residues represent modified sites

[2]IAA = iodoacetamide-derived carbamidomethylation of cysteine residue

[3]Based on PeptideProphet algorithm
