# Supplementary material for: Mercury alters endogenous phosphorylation profiles of SYK in murine B cells
Source: BMC Immunol. 2017 Jul 17;18:37. doi: 10.1186/s12865-017-0221-0 (PMC5514489; doi:10.1186/s12865-017-0221-0)
Supplement: Supplementary file 3 — Identification of SYK phosphopeptides. MS2 phosphopeptide identification information was collated from various studies on WEHI cell extracts, including anti-SYK and anti-pY immunoprecipitation experiments and titanium dioxide enrichments. Peptides were filtered to a ≤ 1% false discovery rate. (PDF 2499 kb) [file 12865_2017_221_MOESM3_ESM.pdf]

Additional File 3. Identification of SYK phosphopeptides. MS2 phosphopeptide identification information was collated from various studies on WEHI cell extracts, including anti-SYK and anti-pY immunoprecipitation experiments and titanium dioxide enrichments. Peptides were filtered to a ≤ 1% false discovery rate.

lumbe	Experiment Type[1]	Cell Treatment[2]	Dosage	Peptide Start	Peptide Stop	Peptide sequence[3]	Variable modifications	Peptide probability	SEQUEST XCorr score	Mascot Ion score	X! Tandem -log(e) score	z	Observed m/z	Actual peptide mass (AMU)	delta mass (AMU)
1	PE	PV	30 μM	2	21	aGSAVDSANHLTyFFGNITR	n-term: Acetyl (+42.01), y13: Phospho (+79.97)	99.70%	0	0	10.5	3	755.01	2,262.01	0.00394
2	PE	PV	30 μM	2	21	aGSAVDSANHLTyFFGNITR	n-term: Acetyl (+42.01), y13: Phospho (+79.97)	99.70%	0	0	15.8	3	755.01	2,262.02	0.00577
3	PE	PV	30 μM	2	21	aGSAVDSANHLTyFFGNITR	n-term: Acetyl (+42.01), y13: Phospho (+79.97)	99.70%	0	0	6.49	3	755.36	2,263.05	1.04300
4	PE	PV	100 μM	2	21	aGSAVDSANHLTyFFGNITR	n-term: Acetyl (+42.01), y13: Phospho (+79.97)	99.70%	0	0	11.2	3	755.01	2,262.02	0.00596
5	PE	PV	100 μM	2	21	aGSAVDSANHLTyFFGNITR	n-term: Acetyl (+42.01), y13: Phospho (+79.97)	99.70%	0	0	9.77	2	1132.01	2,262.01	0.00164
6	PE	PV	100 μM	2	21	aGSAVDSANHLTyFFGNITR	n-term: Acetyl (+42.01), y13: Phospho (+79.97)	99.70%	0	0	12	3	755.01	2,262.02	0.00467
7	PE	PV	100 μM	2	21	aGSAVDSANHLTyFFGNITR	n-term: Acetyl (+42.01), y13: Phospho (+79.97)	99.70%	0	0	13.6	3	755.01	2,262.01	0.00248
8	PE	PV	100 μM	2	21	aGSAVDSANHLTyFFGNITR	n-term: Acetyl (+42.01), y13: Phospho (+79.97)	99.70%	0	0	8.32	2	1132.54	2,263.06	1.04800
9	PE	PV	100 μM	2	21	aGSAVDSANHLTyFFGNITR	n-term: Acetyl (+42.01), y13: Phospho (+79.97)	99.70%	0	0	8.4	2	1132.03	2,262.05	0.03850
10	PE	PV	30 μM	2	41	aGSAVDSANHLTYFFGNITREEAEDyLVQGGMTDGLYLLR	n-term: Acetyl (+42.01), y26: Phospho (+79.97)	99.70%	0	0	11.4	4	1129.78	4,515.10	0.02301
11	PE	PV	100 μM	2	41	aGSAVDSANHLTYFFGNItREEAEDYLVQGGMTDGLYLLR	n-term: Acetyl (+42.01), t19: Phospho (+79.97)	99.70%	0	0	11	4	1129.78	4,515.09	0.01764
12	PE	PV	100 μM	2	41	aGSAVDSANHLTYFFGNITREEAEDyLVQGGMTDGLYLLR	n-term: Acetyl (+42.01), y26: Phospho (+79.97)	99.70%	0	0	11.1	4	1129.78	4,515.09	0.01764
13	PE	PV	100 μM	2	41	aGSAVDSAnHLTYFFGNITREEAEDyLVQGGMTDGLYLLR	Deamidated (+0.98), y26: Phospho (+79.97)	99.60%	0	0	6.4	4	1130.28	4,517.09	1.02900
14	PE	PV	100 μM	2	41	aGSAVDSANHLtYFFGNITREEAEDYLVQGGMTDGLYLLR	n-term: Acetyl (+42.01), t12: Phospho (+79.97)	99.70%	0	0	11.1	4	1129.77	4,515.06	-0.01068
15	PE	PV	100 μM	2	41	aGSAVDSANHLTYFFGNITREEAEDyLVQGGMTDGLYLLR	n-term: Acetyl (+42.01), y26: Phospho (+79.97)	99.70%	0	0	12	4	1129.78	4,515.08	0.00436

16	PE	PV	100 μM	2	41	aGSAVDSANHLTYFFGNItREEAEDYLVQGGMTDGLYLLR	n-term: Acetyl (+42.01), t19: Phospho (+79.97)	99.70%	0	0	13.1	4	1129.79	4,515.13	0.05280
17	PE	PV	100 μM	2	41	aGSAVDSANHLTYFFGNItREEAEDYLVQGGMTDGLYLLR	n-term: Acetyl (+42.01), t19: Phospho (+79.97)	99.70%	0	0	14	3	1506.05	4,515.12	0.04616
18	PE	PV	30 μM	22	41	EEAEDyLVQGGMTDGLYLLR	y6: Phospho (+79.97)	99.70%	1.96	36.1	8.8	3	784.69	2,351.04	0.00363
19	PE	PV	100 μM	22	41	EEAEDyLVQGGMTDGLYLLR	y6: Phospho (+79.97)	99.70%	3	55.8	12.8	3	784.69	2,351.05	0.00546
20	PE	PV	100 μM	22	41	EEAEDyLVQGGMTDGLYLLR	y6: Phospho (+79.97)	99.70%	2.85	72.2	12.6	3	784.69	2,351.04	-0.00003
21	PE	Hg	100 μM	45	58	NyLGGFALSVAHNR	y2: Phospho (+79.97)	99.70%	2.8	19.7	1.04	2	799.88	1,597.75	0.00721
22	PE	PV	30 μM	45	58	NyLGGFALSVAHNR	y2: Phospho (+79.97)	99.70%	1.77	31.4	1.19	2	799.88	1,597.75	0.00562
23	PE	PV	30 μM	45	58	NyLGGFALSVAHNR	y2: Phospho (+79.97)	99.70%	1.92	27	0	2	799.88	1,597.74	0.00306
24	PE	PV	30 μM	45	58	NyLGGFALSVAHNR	y2: Phospho (+79.97)	98.10%	0.8	14.2	1.29	2	799.88	1,597.74	0.00281
25	PE	PV	30 μM	45	58	NyLGGFALSVAHNR	y2: Phospho (+79.97)	99.70%	3.29	73.8	11.5	2	799.88	1,597.74	0.00245
26	PE	PV	30 μM	45	58	NyLGGFALSVAHNR	y2: Phospho (+79.97)	99.70%	3.41	78	10.5	2	799.88	1,597.74	0.00391
27	PE	PV	30 μM	45	58	NyLGGFALSVAHNR	y2: Phospho (+79.97)	99.70%	1.87	82.9	4.07	2	799.88	1,597.74	-0.00195
28	PE	PV	30 μM	45	58	NyLGGFALSVAHNR	y2: Phospho (+79.97)	99.70%	2.34	103	8.37	2	799.88	1,597.74	0.00404
29	PE	PV	30 μM	45	58	NyLGGFALSVAHNR	y2: Phospho (+79.97)	99.70%	1.62	30.8	4.15	3	533.59	1,597.76	0.02215
30	PE	PV	30 μM	45	58	NyLGGFALSVAHNR	y2: Phospho (+79.97)	99.70%	1.99	74.3	9.52	2	799.89	1,597.76	0.02210
31	PE	PV	30 μM	45	58	NyLGGFALSVAHNR	y2: Phospho (+79.97)	99.70%	2.37	56.1	10.7	2	799.89	1,597.76	0.02125
32	PE	PV	30 μM	45	58	NyLGGFALSVAHNR	y2: Phospho (+79.97)	99.70%	1.65	36.9	5.48	3	533.59	1,597.76	0.02270
33	PE	PV	30 μM	45	58	NyLGGFALSVAHNR	y2: Phospho (+79.97)	99.70%	2.55	37.6	4.92	3	533.60	1,597.76	0.02325
34	PE	PV	30 μM	45	58	NyLGGFALSVAHNR	y2: Phospho (+79.97)	99.20%	0.78	12.8	0	2	799.88	1,597.74	0.00269
35	PE	PV	30 μM	45	58	NyLGGFALSVAHNR	y2: Phospho (+79.97)	99.70%	2.46	87.4	15.2	2	799.88	1,597.74	0.00037
36	PE	PV	30 μM	45	58	NyLGGFALSVAHNR	y2: Phospho (+79.97)	99.70%	2.35	39.8	6.35	3	533.59	1,597.75	0.00494
37	PE	PV	100 μM	45	58	NyLGGFALSVAHNR	y2: Phospho (+79.97)	81.60%	2.2	0	0	2	799.88	1,597.74	-0.00134
38	PE	PV	100 μM	45	58	NyLGGFALSVAHNR	y2: Phospho (+79.97)	99.70%	2.44	31.2	0	2	799.88	1,597.74	0.00330
39	PE	PV	100 μM	45	58	NyLGGFALSVAHNR	y2: Phospho (+79.97)	99.70%	2.23	38	1.09	2	799.88	1,597.74	0.00172
40	PE	PV	100 μM	45	58	NyLGGFALSVAHNR	y2: Phospho (+79.97)	99.70%	2.11	22.9	3.17	3	533.59	1,597.74	0.00311
41	PE	PV	100 μM	45	58	NyLGGFALSVAHNR	y2: Phospho (+79.97)	99.70%	2.13	84.5	14.9	2	799.88	1,597.74	0.00184
42	PE	PV	100 μM	45	58	NyLGGFALSVAHNR	y2: Phospho (+79.97)	99.70%	1.76	29.5	4.96	2	799.88	1,597.74	-0.00073
43	PE	PV	100 μM	45	58	NyLGGFALSVAHNR	y2: Phospho (+79.97)	99.70%	3.49	84.9	12.3	2	799.88	1,597.75	0.00501
44	PE	PV	100 μM	45	58	NyLGGFALSVAHNR	y2: Phospho (+79.97)	99.70%	3.78	94.3	12.1	2	799.88	1,597.74	0.00208
45	PE	PV	100 μM	45	58	NyLGGFALSVAHNR	y2: Phospho (+79.97)	99.70%	3.99	89.2	11.4	2	799.88	1,597.74	0.00343
46	PE	PV	100 μM	45	58	NyLGGFALSVAHNR	y2: Phospho (+79.97)	99.70%	3.5	59.6	7.7	3	533.59	1,597.74	0.00329
47	PE	PV	100 μM	45	58	NyLGGFALSVAHNR	y2: Phospho (+79.97)	99.70%	3.89	59.3	7.21	3	533.59	1,597.75	0.00530
48	PE	PV	100 μM	45	58	NyLGGFALSVAHNR	y2: Phospho (+79.97)	99.70%	2.57	113	8.27	2	799.88	1,597.74	-0.00341
49	PE	PV	100 μM	45	58	NyLGGFALSVAHNR	y2: Phospho (+79.97)	99.70%	1.2	21.9	1.15	3	533.59	1,597.74	0.00128
50	PE	PV	100 μM	45	58	NyLGGFALSVAHNR	y2: Phospho (+79.97)	99.70%	1.42	31.6	1.23	3	533.59	1,597.74	0.00182
51	PE	PV	100 μM	45	58	NyLGGFALSVAHNR	y2: Phospho (+79.97)	99.70%	2.74	99.1	15.4	2	799.88	1,597.74	0.00233
52	PE	PV	100 μM	45	58	NyLGGFALSVAHNR	y2: Phospho (+79.97)	99.70%	2.89	110	8.37	2	799.88	1,597.74	0.00233
53	PE	PV	100 μM	45	58	NyLGGFALSVAHNR	y2: Phospho (+79.97)	99.70%	3.04	99	16.2	2	799.88	1,597.74	0.00025
54	PE	PV	100 μM	45	58	NyLGGFALSVAHNR	y2: Phospho (+79.97)	99.70%	3.03	70	13.2	2	799.88	1,597.74	-0.00305
55	PE	PV	100 μM	45	58	NyLGGFALSVAHNR	y2: Phospho (+79.97)	99.70%	1.85	36.3	4.41	3	533.59	1,597.74	0.00128
56	PE	PV	100 μM	45	58	NyLGGFALSVAHNR	y2: Phospho (+79.97)	99.70%	2.51	89.1	8.25	2	799.88	1,597.74	0.00281
57	PE	PV	100 μM	45	58	NyLGGFALSVAHNR	y2: Phospho (+79.97)	99.70%	3.13	108	13.1	2	799.88	1,597.75	0.00574
58	PE	PV	100 μM	45	58	NyLGGFALSVAHNR	y2: Phospho (+79.97)	99.70%	2.27	31.1	3.74	3	533.60	1,597.76	0.02361

59	PE	PV	100 μM	45	58	NyLGGFALSVAHNR	y2: Phospho (+79.97)	99.70%	2.08	31	4.07	3	533.59	1,597.76	0.02288
60	PE	PV	100 μM	45	58	NyLGGFALSVAHNR	y2: Phospho (+79.97)	99.70%	2.56	31.6	5.74	3	533.59	1,597.76	0.02142
61	PE	PV	100 μM	45	58	NyLGGFALSVAHNR	y2: Phospho (+79.97)	99.70%	2.62	40.6	6.37	3	533.59	1,597.76	0.02197
62	PE	PV	100 μM	45	58	NyLGGFALSVAHNR	y2: Phospho (+79.97)	99.70%	2.24	54.7	11.2	2	799.89	1,597.76	0.02076
63	PE	PV	100 μM	45	58	NyLGGFALSVAHNR	y2: Phospho (+79.97)	99.70%	2.22	76.7	10.1	2	799.89	1,597.76	0.02296
64	PE	PV	100 μM	45	58	NyLGGFALSVAHNR	y2: Phospho (+79.97)	99.70%	2.33	67.8	10.1	2	799.89	1,597.76	0.02430
65	PE	PV	100 μM	45	58	NyLGGFALSVAHNR	y2: Phospho (+79.97)	99.70%	2.56	70.4	8.7	2	799.89	1,597.76	0.02393
66	PE	PV	100 μM	45	58	NyLGGFALSVAHNR	y2: Phospho (+79.97)	99.70%	3.03	65.4	9	2	799.89	1,597.76	0.02296
67	PE	PV	100 μM	45	58	NyLGGFALSVAHNR	y2: Phospho (+79.97)	99.70%	2.56	47	6.3	3	533.59	1,597.76	0.02288
68	PE	PV	100 μM	45	58	NyLGGFALSVAHNR	y2: Phospho (+79.97)	99.70%	2.72	50.9	5.54	3	533.59	1,597.76	0.02233
69	PE	PV	100 μM	45	58	NyLGGFALSVAHNR	y2: Phospho (+79.97)	99.50%	0.74	17.4	1.11	2	799.88	1,597.74	-0.00085
70	PE	PV	100 μM	45	58	NyLGGFALSVAHNR	y2: Phospho (+79.97)	99.70%	1.34	50.5	3.49	2	799.88	1,597.75	0.00672
71	PE	PV	100 μM	45	58	NyLGGFALSVAHNR	y2: Phospho (+79.97)	99.70%	2.66	77.2	12.7	2	799.88	1,597.74	0.00013
72	PE	PV	100 μM	45	58	NyLGGFALSVAHNR	y2: Phospho (+79.97)	99.70%	2.64	37	8.26	3	533.59	1,597.74	0.00054
73	PE	PV	100 μM	45	59	NyLGGFALSVAHNRK	y2: Phospho (+79.97)	99.70%	3.33	26.5	5.31	3	576.29	1,725.84	0.00485
74	PE	PV	100 μM	45	59	NyLGGFALSVAHNRK	y2: Phospho (+79.97)	99.70%	3.68	33.2	6.37	3	576.29	1,725.84	0.00393
75	PE	PV	100 μM	45	59	NyLGGFALSVAHNRK	y2: Phospho (+79.97)	99.70%	2.33	28.2	5.47	3	576.29	1,725.83	-0.00028
76	PE	PV	30 μM	59	67	KAHHyTIER	y5: Phospho (+79.97)	99.20%	0.93	16.6	0	3	412.20	1,233.56	-0.00085
77	PE	PV	30 μM	59	67	KAHHyTIER	y5: Phospho (+79.97)	99.70%	2.6	22	2.77	3	412.20	1,233.56	-0.00122
78	PE	PV	100 μM	59	67	KAHHyTIER	y5: Phospho (+79.97)	99.70%	2.15	28.7	2.82	3	412.20	1,233.57	0.00180
79	PE	PV	100 μM	59	67	KAHHyTIER	y5: Phospho (+79.97)	99.70%	2.06	18.8	3	3	412.20	1,233.57	0.00061
80	PE	PV	100 μM	59	67	KAHHyTIER	y5: Phospho (+79.97)	99.70%	2.03	37.7	3.37	3	412.20	1,233.56	-0.00149
81	PE	PV	100 μM	59	67	KAHHyTIER	y5: Phospho (+79.97)	99.70%	2.18	22.2	2.96	3	412.20	1,233.56	-0.00140
82	PE	PV	100 μM	59	67	KAHHyTIER	y5: Phospho (+79.97)	99.70%	3.08	16.1	3.14	3	412.20	1,233.56	-0.00122
83	PE	PV	100 μM	59	67	KAHHyTIER	y5: Phospho (+79.97)	99.70%	2.13	30.7	2.34	3	412.20	1,233.56	-0.00113
84	PE	Hg	50 μM	68	79	ELNGTyAISGGR	y6: Phospho (+79.97)	99.70%	2.71	33.2	4	2	659.30	1,316.58	0.00241
85	PE	Hg	50 μM	68	79	ELNGTyAISGGR	y6: Phospho (+79.97)	99.70%	1.35	52.4	2.14	2	659.29	1,316.58	-0.00101
86	PE	Hg	50 μM	68	79	ELNGTyAISGGR	y6: Phospho (+79.97)	99.70%	1.81	74.7	5.4	2	659.30	1,316.58	0.00070
87	PE	Hg	50 μM	68	79	ELNGTyAISGGR	y6: Phospho (+79.97)	99.70%	2.84	51.1	7.4	2	659.30	1,316.58	0.00082
88	PE	Hg	50 μM	68	79	ELNGTyAISGGR	y6: Phospho (+79.97)	99.70%	3.14	51.2	7.39	2	659.30	1,316.58	0.00009
89	PE	Hg	50 μM	68	79	ELnGtYAISGGR	n3: Deamidated (+0.98), t5: Phospho (+79.97)	99.60%	0.98	27.7	0	2	659.79	1,317.56	0.00374
90	PE	Hg	50 μM	68	79	ELNGTyAISGGR	y6: Phospho (+79.97)	99.70%	1.51	57.2	3.06	2	659.30	1,316.58	0.00631
91	PE	Hg	50 μM	68	79	ELNGTyAISGGR	y6: Phospho (+79.97)	99.70%	2.96	66.7	5.33	2	659.30	1,316.58	0.00436
92	PE	Hg	50 μM	68	79	ELNGtYAISGGR	t5: Phospho (+79.97)	99.70%	2.57	37.7	5.82	2	659.31	1,316.60	0.01938
93	PE	Hg	50 μM	68	79	ELNGTyAISGGR	y6: Phospho (+79.97)	99.70%	2.45	42	5.64	2	659.30	1,316.59	0.01571
94	PE	Hg	50 μM	68	79	ELNGtYAISGGR	t5: Phospho (+79.97)	99.70%	2.45	58.1	6.21	2	659.30	1,316.59	0.01803
95	PE	Hg	100 μM	68	79	ELnGTyAISGGR	n3: Deamidated (+0.98), y6: Phospho (+79.97)	98.90%	2.3	28.3	0	2	659.79	1,317.57	0.00631
96	PE	Hg	100 μM	68	79	ELNGtYAISGGR	t5: Phospho (+79.97)	99.50%	2.15	0	1.03	2	659.30	1,316.58	0.00448
97	PE	Hg	100 μM	68	79	ELNGTyAISGGR	y6: Phospho (+79.97)	99.70%	3.47	26.3	1.46	2	659.30	1,316.58	0.00216
98	PE	Hg	100 μM	68	79	ELNGTyAISGGR	y6: Phospho (+79.97)	99.70%	3.06	54.3	4.92	2	659.30	1,316.58	0.00473
99	PE	Hg	100 μM	68	79	ELNGtYAISGGR	t5: Phospho (+79.97)	95.00%	0.95	34.1	1.23	2	659.30	1,316.58	0.00058
100	PE	Hg	100 μM	68	79	ELNGTyAISGGR	y6: Phospho (+79.97)	97.80%	1.27	63.1	0.77	2	659.30	1,316.58	0.00058

101	PE	Hg	100 µM	68	79	ELnGTyAISGGR	n3: Deamidated (+0.98), y6: Phospho (+79.97)	98.70%	1.06	63.5	1.54	2	659.79	1,317.56	-0.00102
102	PE	Hg	100 µM	68	79	ELnGTyAISGGR	n3: Deamidated (+0.98), y6: Phospho (+79.97)	99.10%	1.38	65.4	2.17	2	659.79	1,317.56	0.00118
103	PE	Hg	100 µM	68	79	ELNGTyAISGGR	y6: Phospho (+79.97)	99.70%	1.78	78.4	2.57	2	659.30	1,316.58	0.00168
104	PE	Hg	100 µM	68	79	ELNGTyAISGGR	y6: Phospho (+79.97)	99.70%	2.27	60.4	4.55	2	659.30	1,316.58	-0.00016
105	PE	Hg	100 µM	68	79	ELNGTyAISGGR	y6: Phospho (+79.97)	99.70%	1.9	67.3	6.11	2	659.30	1,316.58	0.00229
106	PE	Hg	100 µM	68	79	ELNGTyAISGGR	y6: Phospho (+79.97)	99.70%	2.93	66.7	5.72	2	659.30	1,316.58	0.00216
107	PE	Hg	100 µM	68	79	ELNGTyAISGGR	y6: Phospho (+79.97)	99.70%	0	49.5	6.47	2	659.30	1,316.58	0.00436
108	PE	Hg	100 µM	68	79	ELNGTyAISGGR	y6: Phospho (+79.97)	99.70%	0	50	7.15	2	659.30	1,316.58	0.00546
109	PE	Hg	100 µM	68	79	ELNGTyAISGGR	y6: Phospho (+79.97)	99.70%	0	49.6	7.28	2	659.30	1,316.58	0.00326
110	PE	Hg	100 µM	68	79	ELnGTyAISGGR	n3: Deamidated (+0.98), y6: Phospho (+79.97)	99.70%	1.61	62.4	2.92	2	659.79	1,317.56	-0.00248
111	PE	Hg	100 µM	68	79	ELNGTyAISGGR	y6: Phospho (+79.97)	99.70%	2.4	56.9	4.08	2	659.29	1,316.57	-0.00333
112	PE	Hg	100 µM	68	79	ELNGTyAISGGR	y6: Phospho (+79.97)	99.70%	3.26	42.7	5.74	2	659.30	1,316.58	-0.00016
113	PE	Hg	100 µM	68	79	ELNGTyAISGGR	y6: Phospho (+79.97)	99.70%	3.25	41.7	6.33	2	659.29	1,316.57	-0.00138
114	PE	Hg	100 µM	68	79	ELNGTyAISGGR	y6: Phospho (+79.97)	99.70%	3.19	50	7.96	2	659.30	1,316.58	0.00094
115	PE	Hg	100 µM	68	79	ELnGtYAISGGR	n3: Deamidated (+0.98), t5: Phospho (+79.97)	97.10%	0.97	18.5	0	2	659.79	1,317.56	0.00155
116	PE	Hg	100 µM	68	79	ELNGTyAISGGR	y6: Phospho (+79.97)	99.70%	1.46	56	2.08	2	659.30	1,316.58	0.00448
117	PE	Hg	100 µM	68	79	ELNGTyAISGGR	y6: Phospho (+79.97)	99.70%	3.33	53	4.82	2	659.30	1,316.58	0.00595
118	PE	Hg	100 µM	68	79	ELNGtYAISGGR	t5: Phospho (+79.97)	99.70%	2.26	26.6	5.89	2	659.30	1,316.59	0.01779
119	PE	PV	1 µM	68	79	ELNGtYAISGGR	t5: Phospho (+79.97)	83.00%	0.25	0	0	2	659.29	1,316.57	-0.00443
120	PE	PV	10 µM	68	79	ELNGTyAISGGR	y6: Phospho (+79.97)	94.70%	1.8	12.7	0	2	659.30	1,316.58	0.00387
121	PE	PV	10 µM	68	79	ELNGTyAISGGR	y6: Phospho (+79.97)	99.70%	1.19	44.2	2.05	2	659.30	1,316.58	0.00168
122	PE	PV	30 µM	68	79	ELNGTyAISGGR	y6: Phospho (+79.97)	97.60%	1.27	15.6	0	2	659.30	1,316.58	0.00851
123	PE	PV	30 µM	68	79	ELNGTyAISGGR	y6: Phospho (+79.97)	99.30%	2.81	26.2	1.48	2	659.30	1,316.58	0.00680
124	PE	PV	30 µM	68	79	ELNGTyAISGGR	y6: Phospho (+79.97)	99.70%	2.59	38.4	2.01	2	659.30	1,316.58	0.00546
125	PE	PV	30 µM	68	79	ELNGTyAISGGR	y6: Phospho (+79.97)	99.70%	2.51	33.1	1.52	2	659.29	1,316.58	-0.00089
126	PE	PV	30 µM	68	79	ELNGTyAISGGR	y6: Phospho (+79.97)	99.50%	1.7	61.7	1.37	2	659.30	1,316.58	0.00290
127	PE	PV	30 µM	68	79	ELNGTyAISGGR	y6: Phospho (+79.97)	99.70%	2.21	62.4	2.62	2	659.30	1,316.58	0.00497
128	PE	PV	30 µM	68	79	ELnGTyAISGGR	n3: Deamidated (+0.98), y6: Phospho (+79.97)	99.70%	1.44	58.3	3.28	2	659.79	1,317.56	-0.00114
129	PE	PV	30 µM	68	79	ELnGTyAISGGR	n3: Deamidated (+0.98), y6: Phospho (+79.97)	99.70%	2.51	66.1	5.89	2	659.79	1,317.56	-0.00297
130	PE	PV	30 µM	68	79	ELNGTyAISGGR	y6: Phospho (+79.97)	99.70%	2.83	71.7	5.72	2	659.30	1,316.58	0.00277
131	PE	PV	30 µM	68	79	ELNGTyAISGGR	y6: Phospho (+79.97)	99.70%	1.78	31.9	2.7	2	659.30	1,316.58	0.00314
132	PE	PV	30 µM	68	79	ELnGTyAISGGR	n3: Deamidated (+0.98), y6: Phospho (+79.97)	99.70%	2.94	44.7	4.92	2	659.79	1,317.57	0.00472
133	PE	PV	30 µM	68	79	ELNGTyAISGGR	y6: Phospho (+79.97)	99.70%	3	34.7	6.02	2	659.30	1,316.58	0.00338
134	PE	PV	30 µM	68	79	ELNGTyAISGGR	y6: Phospho (+79.97)	99.70%	3.16	49.6	7.74	2	659.30	1,316.58	0.00387
135	PE	PV	30 µM	68	79	ELNGTyAISGGR	y6: Phospho (+79.97)	99.70%	3.43	48.3	8.18	2	659.30	1,316.58	0.00326
136	PE	PV	30 µM	68	79	ELNGTyAISGGR	y6: Phospho (+79.97)	99.70%	3.4	50.3	7.13	2	659.30	1,316.58	0.00363
137	PE	PV	30 µM	68	79	ELnGTyAISGGR	n3: Deamidated (+0.98), y6: Phospho (+79.97)	99.70%	1.3	69.4	3.48	2	659.79	1,317.56	0.00094

138	PE	PV	30 μM	68	79	ELNGTyAISGGR	y6: Phospho (+79.97)	99.70%	2.73	57	5.08	2	659.29	1,316.58	-0.00101
139	PE	PV	30 μM	68	79	ELNGTyAISGGR	y6: Phospho (+79.97)	99.70%	2.74	52.3	4.06	2	659.29	1,316.58	-0.00126
140	PE	PV	30 μM	68	79	ELNGTyAISGGR	y6: Phospho (+79.97)	99.70%	2.95	51.3	4.25	2	659.30	1,316.58	0.00351
141	PE	PV	30 μM	68	79	ELNGTyAISGGR	y6: Phospho (+79.97)	99.70%	3.42	49.7	7.64	2	659.30	1,316.58	0.00058
142	PE	PV	30 μM	68	79	ELNGTyAISGGR	y6: Phospho (+79.97)	99.70%	3.41	49.9	5.44	2	659.29	1,316.58	-0.00101
143	PE	PV	30 μM	68	79	ELNGTyAISGGR	y6: Phospho (+79.97)	99.70%	3.06	66.6	5.64	2	659.30	1,316.58	0.00424
144	PE	PV	30 μM	68	79	ELNGTyAISGGR	y6: Phospho (+79.97)	99.70%	2.67	36.9	4.89	2	659.30	1,316.60	0.01901
145	PE	PV	30 μM	68	79	ELNGTyAISGGR	y6: Phospho (+79.97)	99.70%	2.39	39.1	5.55	2	659.30	1,316.59	0.01791
146	PE	PV	30 μM	68	79	ELNGTyAISGGR	y6: Phospho (+79.97)	99.70%	2.24	39.6	6.27	2	659.30	1,316.59	0.01779
147	PE	PV	30 μM	68	79	ELNGTyAISGGR	y6: Phospho (+79.97)	99.70%	2.12	28.2	3.72	2	659.30	1,316.58	0.00619
148	PE	PV	30 μM	68	79	ELNGTyAISGGR	y6: Phospho (+79.97)	99.70%	2.64	57.6	5.52	2	659.30	1,316.58	0.00107
149	PE	PV	100 μM	68	79	ELNGTyAISGGR	y6: Phospho (+79.97)	89.60%	2.79	0	0	2	659.30	1,316.58	0.00314
150	PE	PV	100 μM	68	79	ELNGTyAISGGR	y6: Phospho (+79.97)	98.70%	1.85	13.4	0.89	2	659.30	1,316.58	0.00326
151	PE	PV	100 μM	68	79	ELnGTyAISGGR	n3: Deamidated (+0.98), y6: Phospho (+79.97)	99.70%	2.15	34	1.05	2	659.79	1,317.56	0.00216
152	PE	PV	100 μM	68	79	ELNGTyAISGGR	y6: Phospho (+79.97)	99.70%	2.79	55	3.4	2	659.30	1,316.58	0.00119
153	PE	PV	100 μM	68	79	ELNGTyAISGGR	y6: Phospho (+79.97)	99.70%	2.97	38.2	3.35	2	659.30	1,316.58	0.00204
154	PE	PV	100 μM	68	79	ELNGTyAISGGR	y6: Phospho (+79.97)	99.70%	2.41	62.8	6.24	2	659.30	1,316.58	0.00631
155	PE	PV	100 μM	68	79	ELnGTyAISGGR	n3: Deamidated (+0.98), y6: Phospho (+79.97)	99.70%	2.58	74.8	4.96	2	659.79	1,317.56	-0.00138
156	PE	PV	100 μM	68	79	ELNGTyAISGGR	y6: Phospho (+79.97)	99.70%	2.73	59.7	5.6	2	659.30	1,316.58	0.00277
157	PE	PV	100 μM	68	79	ELNGTyAISGGR	y6: Phospho (+79.97)	99.70%	2.97	66.5	4.77	2	659.30	1,316.58	0.00094
158	PE	PV	100 μM	68	79	ELnGTyAISGGR	n3: Deamidated (+0.98), y6: Phospho (+79.97)	99.70%	2.65	56	4.08	2	659.79	1,317.56	0.00008
159	PE	PV	100 μM	68	79	ELNGTyAISGGR	y6: Phospho (+79.97)	99.70%	3.35	53.2	4.66	2	659.30	1,316.58	-0.00028
160	PE	PV	100 μM	68	79	ELNGTyAISGGR	y6: Phospho (+79.97)	99.70%	3.35	51.3	5.05	2	659.30	1,316.58	0.00082
161	PE	PV	100 μM	68	79	ELNGTyAISGGR	y6: Phospho (+79.97)	99.70%	2.56	33.9	4.89	2	659.30	1,316.58	0.00412
162	PE	PV	100 μM	68	79	ELnGTyAISGGR	n3: Deamidated (+0.98), y6: Phospho (+79.97)	99.70%	2.89	47.9	4.41	2	659.79	1,317.57	0.00557
163	PE	PV	100 μM	68	79	ELnGTyAISGGR	n3: Deamidated (+0.98), y6: Phospho (+79.97)	99.70%	3.05	53.5	5.85	2	659.79	1,317.57	0.00545
164	PE	PV	100 μM	68	79	ELnGTyAISGGR	n3: Deamidated (+0.98), y6: Phospho (+79.97)	99.70%	2.76	52.5	6.7	2	659.79	1,317.57	0.00509
165	PE	PV	100 μM	68	79	ELNGTyAISGGR	y6: Phospho (+79.97)	99.70%	3.23	50	7.14	2	659.30	1,316.58	0.00656
166	PE	PV	100 μM	68	79	ELNGTyAISGGR	y6: Phospho (+79.97)	99.70%	3.41	50	6.26	2	659.30	1,316.58	0.00400
167	PE	PV	100 μM	68	79	ELNGTyAISGGR	y6: Phospho (+79.97)	99.70%	3.43	51.3	5.85	2	659.30	1,316.58	0.00424
168	PE	PV	100 μM	68	79	ELnGtYAISGGR	n3: Deamidated (+0.98), t5: Phospho (+79.97)	99.70%	2.84	38	6.35	2	659.79	1,317.57	0.00594
169	PE	PV	100 μM	68	79	ELNGTyAISGGR	y6: Phospho (+79.97)	99.70%	3.41	51.4	6.17	2	659.30	1,316.58	0.00412
170	PE	PV	100 μM	68	79	ELnGtYAISGGR	n3: Deamidated (+0.98), t5: Phospho (+79.97)	99.70%	2.83	60.9	4.25	2	659.79	1,317.56	0.00020
171	PE	PV	100 μM	68	79	ELnGTyAISGGR	n3: Deamidated (+0.98), y6: Phospho (+79.97)	99.60%	2.49	54	4.32	2	659.79	1,317.57	0.00655
172	PE	PV	100 μM	68	79	ELNGTyAISGGR	y6: Phospho (+79.97)	99.70%	3.13	49.3	5.66	2	659.30	1,316.58	0.00326
173	PE	PV	100 μM	68	79	ELNGTyAISGGR	y6: Phospho (+79.97)	99.70%	3.3	52.9	5.77	2	659.30	1,316.58	0.00326

174	PE	PV	100 µM	68	79	ELnGTyAISGGR	n3: Deamidated (+0.98), y6: Phospho (+79.97)	99.70%	2.99	46.7	7.72	2	659.79	1,317.56	0.00350
175	PE	PV	100 µM	68	79	ELnGtYAISGGR	n3: Deamidated (+0.98), t5: Phospho (+79.97)	99.70%	3.11	39.7	7.44	2	659.79	1,317.56	0.00118
176	PE	PV	100 µM	68	79	ELNGTyAISGGR	y6: Phospho (+79.97)	99.70%	3.51	52.9	5.33	2	659.30	1,316.58	0.00168
177	PE	PV	100 µM	68	79	ELNGTyAISGGR	y6: Phospho (+79.97)	99.70%	3.36	49.9	7.62	2	659.30	1,316.58	-0.00077
178	PE	PV	100 µM	68	79	ELnGTyAISGGR	n3: Deamidated (+0.98), y6: Phospho (+79.97)	99.70%	3.16	53.6	6.12	2	659.79	1,317.56	0.00094
179	PE	PV	100 µM	68	79	ELnGTyAISGGR	n3: Deamidated (+0.98), y6: Phospho (+79.97)	99.70%	3.24	53.8	6.42	2	659.79	1,317.56	0.00094
180	PE	PV	100 µM	68	79	ELNGTyAISGGR	y6: Phospho (+79.97)	99.70%	1.4	37.7	2.12	2	659.30	1,316.58	0.00532
181	PE	PV	100 µM	68	79	ELnGTyAISGGR	n3: Deamidated (+0.98), y6: Phospho (+79.97)	99.70%	2.7	62.9	5.3	2	659.79	1,317.56	0.00179
182	PE	PV	100 µM	68	79	ELNGTyAISGGR	y6: Phospho (+79.97)	98.40%	1.8	21.1	0	1	1317.60	1,316.59	0.01603
183	PE	PV	100 µM	68	79	ELnGtYAISGGR	n3: Deamidated (+0.98), t5: Phospho (+79.97)	99.70%	2.09	36.5	5.09	2	659.80	1,317.58	0.02059
184	PE	PV	100 µM	68	79	ELNGTyAISGGR	y6: Phospho (+79.97)	99.70%	2.99	49.3	4.55	2	659.30	1,316.59	0.01754
185	PE	PV	100 µM	68	79	ELnGtYAISGGR	n3: Deamidated (+0.98), t5: Phospho (+79.97)	99.70%	2.32	38.3	5.22	2	659.80	1,317.58	0.01998
186	PE	PV	100 µM	68	79	ELnGTyAISGGR	n3: Deamidated (+0.98), y6: Phospho (+79.97)	99.70%	2.24	44	5.89	2	659.80	1,317.58	0.02218
187	PE	PV	100 µM	68	79	ELNGTyAISGGR	y6: Phospho (+79.97)	99.70%	2.45	52.7	5.22	2	659.30	1,316.59	0.01779
188	PE	PV	100 µM	68	79	ELNGTyAISGGR	y6: Phospho (+79.97)	99.70%	2.93	36.1	5.68	2	659.31	1,316.60	0.01925
189	PE	PV	100 µM	68	79	ELNGTyAISGGR	y6: Phospho (+79.97)	99.70%	2.18	71.5	7.05	2	659.30	1,316.59	0.01559
190	PE	PV	100 µM	68	79	eLnGTyAISGGR	n-term: Dehydrated (-18.01), n3: Deamidated (+0.98), y6: Phospho (+79.97)	97.60%	0	0	2.09	2	650.78	1,299.55	-0.00034
191	PE	PV	100 µM	68	79	ELNGTyAISGGR	y6: Phospho (+79.97)	99.70%	1.51	46.5	1.89	2	659.30	1,316.58	0.00375
192	PE	PV	30 µM	80	103	AHASPADLcHYHsQEPDGLIcLLK	c9: Carbamidomethyl (+57.02), s13: Phospho (+79.97), c21: Carbamidomethyl (+57.02)	99.70%	2.56	14.1	4.03	4	703.83	2,811.27	0.01961
193	PE	PV	30 µM	80	103	AHASPADLcHyHSQEPDGLIcLLK	c9: Carbamidomethyl (+57.02), y11: Phospho (+79.97), c21: Carbamidomethyl (+57.02)	99.70%	3.55	30.6	5.42	4	703.82	2,811.26	0.00594
194	PE	PV	30 µM	80	103	AHASPADLcHYHsQEPDGLIcLLK	c9: Carbamidomethyl (+57.02), s13: Phospho (+79.97), c21: Carbamidomethyl (+57.02)	99.70%	3.85	26	4.19	4	703.82	2,811.26	0.00643
195	PE	PV	100 µM	80	103	AHASPADLcHyHSQEPDGLIcLLK	c9: Carbamidomethyl (+57.02), y11: Phospho (+79.97), c21: Carbamidomethyl (+57.02)	99.70%	3.93	30.5	6.37	4	703.82	2,811.25	0.00325
196	PE	PV	100 µM	80	103	AHASPADLcHYHsQEPDGLIcLLK	c9: Carbamidomethyl (+57.02), s13: Phospho (+79.97), c21: Carbamidomethyl (+57.02)	99.70%	2.57	27.1	3.38	4	703.82	2,811.26	0.00863
197	PE	PV	10 µM	116	132	TGPFEDLKENLIREyVK	y15: Phospho (+79.97)	99.70%	2.68	50.1	12.2	3	711.02	2,130.04	0.00106
198	PE	PV	10 µM	116	132	TGPFEDLKENLIREyVK	y15: Phospho (+79.97)	99.70%	3.37	29.8	6.04	3	711.02	2,130.04	-0.00077

199	PE	PV	10 μM	116	132	TGPFEDLKENLIREyVK	y15: Phospho (+79.97)	99.70%	2.94	37.5	4.14	3	711.02	2,130.04	0.00289
200	PE	PV	30 μM	116	132	TGPFEDLKENLIREyVK	y15: Phospho (+79.97)	99.70%	3.11	23.2	6.1	3	711.02	2,130.04	-0.00169
201	PE	PV	30 μM	116	132	TGPFEDLKENLIREyVK	y15: Phospho (+79.97)	99.70%	3.35	39.4	4.89	3	711.02	2,130.04	-0.00370
202	PE	PV	100 μM	116	132	TGPFEDLKENLIREyVK	y15: Phospho (+79.97)	99.70%	3.65	38.1	8.07	3	711.02	2,130.04	-0.00041
203	PE	PV	30 μM	129	155	EyVKQTWNLQGQALEQAIISQKPQLEK	y2: Phospho (+79.97)	99.00%	1.42	0	0.43	4	813.42	3,249.65	0.00180
204	PE	PV	30 μM	129	155	EyVKQTWNLQGQALEQAIISQKPQLEK	y2: Phospho (+79.97)	99.30%	1.09	0	0.48	4	813.42	3,249.64	-0.00357
205	PE	PV	30 μM	129	155	EyVKQTWNLQGQALEQAIISQKPQLEK	y2: Phospho (+79.97)	99.60%	1.25	0	0.52	4	813.42	3,249.64	-0.00016
206	PE	PV	30 μM	129	155	EyVKQTWNLQGQALEQAIISQKPQLEK	y2: Phospho (+79.97)	99.70%	1.63	27.2	2.41	3	1084.22	3,249.65	0.00737
207	PE	PV	30 μM	129	155	EyVKQTWNLQGQALEQAIISQKPQLEK	y2: Phospho (+79.97)	99.70%	2.21	26.9	6.96	4	813.42	3,249.65	0.01034
208	PE	PV	100 μM	129	155	EyVKQTWNLQGQALEQAIISQKPQLEK	y2: Phospho (+79.97)	93.70%	0.91	0	2.17	4	813.42	3,249.65	0.00400
209	PE	PV	100 μM	129	155	EyVKQTWNLQGQALEQAIISQKPQLEK	y2: Phospho (+79.97)	99.30%	1.86	0	1.36	4	813.42	3,249.65	0.00888
210	PE	PV	100 μM	129	155	EyVKQTWNLQGQALEQAIISQKPQLEK	y2: Phospho (+79.97)	99.70%	1.43	0	1.66	4	813.42	3,249.65	0.00204
211	PE	PV	100 μM	129	155	EyVKQTWNLQGQALEQAIISQKPQLEK	y2: Phospho (+79.97)	99.70%	1.31	16.3	2.52	4	813.42	3,249.65	0.00131
212	PE	PV	100 μM	129	155	EyVKQTWNLQGQALEQAIISQKPQLEK	y2: Phospho (+79.97)	99.70%	1.55	29.3	3.72	4	813.42	3,249.65	0.01107
213	PE	PV	100 μM	129	155	EyVKQTWNLQGQALEQAIISQKPQLEK	y2: Phospho (+79.97)	99.70%	2.46	31.8	4.85	4	813.42	3,249.64	-0.00211
214	PE	PV	100 μM	129	155	EyVKQTWNLQGQALEQAIISQKPQLEK	y2: Phospho (+79.97)	99.70%	3.18	24.6	1.8	4	813.42	3,249.64	-0.00504
215	PE	PV	30 μM	195	211	ARDNSGSyALcLLHEGK	y8: Phospho (+79.97), c11: Carbamidomethyl (+57.02)	99.60%	1.27	18.8	2.31	3	657.63	1,969.88	0.00328
216	PE	PV	100 μM	195	211	ARDNSGSyALcLLHEGK	y8: Phospho (+79.97), c11: Carbamidomethyl (+57.02)	99.70%	1.42	18.3	3.26	3	657.63	1,969.87	-0.00093
217	PE	PV	30 μM	197	211	DNSGSyALcLLHEGK	y6: Phospho (+79.97), c9: Carbamidomethyl (+57.02)	99.70%	3.38	34	6.43	3	581.92	1,742.74	0.00628
218	PE	PV	100 μM	197	211	DNsGSYALcLLHEGK	s3: Phospho (+79.97), c9: Carbamidomethyl (+57.02)	96.40%	0.74	13.4	0	3	581.92	1,742.74	0.00317
219	PE	PV	100 μM	197	211	DNsGSYALcLLHEGK	s3: Phospho (+79.97), c9: Carbamidomethyl (+57.02)	99.70%	0.94	29.3	3.38	3	581.92	1,742.74	0.00885
220	PE	PV	30 μM	232	252	KFDTLWQLVEHYSyKPDGLLR	y14: Phospho (+79.97)	99.70%	2.07	31.5	6.54	4	672.84	2,687.32	0.00921
221	PE	PV	30 μM	232	252	KFDTLWQLVEHySYKPDGLLR	y12: Phospho (+79.97)	99.70%	2.99	35	8.72	4	672.84	2,687.33	0.01189
222	PE	PV	30 μM	232	252	KFDTLWQLVEHYsYKPDGLLR	s13: Phospho (+79.97)	99.70%	2.75	22.6	7.89	4	672.84	2,687.32	0.00408
223	PE	PV	100 μM	232	252	KFDTLWQLVEHySYKPDGLLR	y12: Phospho (+79.97)	99.70%	3.73	32.2	7.89	4	672.84	2,687.32	0.00848
224	IP	Ig	2 min	261	286	IGAQmGHPGsPNAHPVTWSPGGIISR	m5: Oxidation (+15.99), s10: Phospho (+79.97)	99.70%	4.22	37.5	3.34	3	907.44	2,719.29	0.01879
225	IP	Ig	2 min	261	286	IGAQmGHPGsPNAHPVTWSPGGIISR	m5: Oxidation (+15.99), s10: Phospho (+79.97)	98.20%	4.01	34.3	1.55	3	907.44	2,719.29	0.02062
226	IP	Ig	2 min	261	286	IGAQmGHPGsPNAHPVTWSPGGIISR	m5: Oxidation (+15.99), s10: Phospho (+79.97)	98.70%	3.66	29.4	1.96	3	907.44	2,719.29	0.02428
227	IP	Ig	2 min	261	286	IGAQmGHPGsPNAHPVTWSPGGIISR	m5: Oxidation (+15.99), s10: Phospho (+79.97)	98.80%	4.47	50.3	2.18	3	907.44	2,719.29	0.02044
228	IP	Ig	2 min	261	286	IGAQmGHPGsPNAHPVTWSPGGIISR	m5: Oxidation (+15.99), s10: Phospho (+79.97)	97.70%	2.38	31	3	3	907.43	2,719.27	0.00158
229	IP	Ig	2 min	261	286	IGAQmGHPGsPNAHPVTWSPGGIISR	m5: Oxidation (+15.99), s10: Phospho (+79.97)	98.90%	2.01	41	4.29	4	680.82	2,719.27	0.00114
230	IP	Ig	2 min	261	286	IGAQmGHPGsPNAHPVTWSPGGIISR	m5: Oxidation (+15.99), s10: Phospho (+79.97)	99.50%	4.22	27.2	2	3	907.43	2,719.27	0.00103

231	IP	Ig	2 min	261	286	IGAQmGHPGsPNAHPVTWSPGGIISR	m5: Oxidation (+15.99), s10: Phospho (+79.97)	99.70%	3.69	24.1	2.36	3	907.43	2,719.27	0.00121
232	IP	Ig	2 min	261	286	IGAQmGHPGsPNAHPVTWSPGGIISR	m5: Oxidation (+15.99), s10: Phospho (+79.97)	99.70%	4.11	48.4	4.14	3	907.43	2,719.27	0.00121
233	IP	NT	0	261	286	IGAQmGHPGsPNAHPVTWSPGGIISR	m5: Oxidation (+15.99), s10: Phospho (+79.97)	97.80%	1.08	20.6	1.47	3	907.43	2,719.28	0.01037
234	IP	NT	0	261	286	IGAQMGHPGsPNAHPVTWSPGGIISR	s10: Phospho (+79.97)	99.70%	2.28	28	4.05	3	902.10	2,703.28	0.00132
235	IP	NT	0	261	286	IGAQmGHPGsPNAHPVTWSPGGIISR	m5: Oxidation (+15.99), s10: Phospho (+79.97)	99.70%	2.47	32.8	7.92	4	680.83	2,719.27	0.00334
236	IP	NT	0	261	286	IGAQmGHPGsPNAHPVTWSPGGIISR	m5: Oxidation (+15.99), s10: Phospho (+79.97)	99.70%	2.37	35.3	3.42	3	907.43	2,719.28	0.00597
237	IP	NT	0	261	286	IGAQMGHPGsPNAHPVTWSPGGIISR	s10: Phospho (+79.97)	94.00%	1.44	28.1	1.77	3	902.10	2,703.27	0.00040
238	IP	NT	0	261	286	IGAQmGHPGsPNAHPVTWSPGGIISR	m5: Oxidation (+15.99), s10: Phospho (+79.97)	99.50%	3.95	45.9	8.48	4	680.82	2,719.27	-0.00057
239	IP	NT	0	261	286	IGAQmGHPGsPNAHPVTWSPGGIISR	m5: Oxidation (+15.99), s10: Phospho (+79.97)	99.70%	4.52	30	4.04	3	907.43	2,719.28	0.00817
240	IP	NT	0	261	286	IGAQmGHPGsPNAHPVTWSPGGIISR	m5: Oxidation (+15.99), s10: Phospho (+79.97)	99.60%	3.61	26.4	0	3	907.43	2,719.27	0.00103
241	IP	Hg	50 µM	261	286	IGAQmGHPGsPNAHPVTWSPGGIISR	m5: Oxidation (+15.99), s10: Phospho (+79.97)	96.50%	3.46	28.4	0	3	907.44	2,719.29	0.02062
242	IP	Hg	50 µM	261	286	IGAQmGHPGsPNAHPVTWSPGGIISR	m5: Oxidation (+15.99), s10: Phospho (+79.97)	99.70%	3.9	35.7	0	3	907.44	2,719.29	0.01751
243	IP	Hg	50 µM	261	286	IGAQmGHPGsPNAHPVTWSPGGIISR	m5: Oxidation (+15.99), s10: Phospho (+79.97)	99.70%	5.39	45.9	9.7	4	680.82	2,719.27	0.00089
244	IP	Hg	50 µM	261	286	IGAQmGHPGsPNAHPVTWSPGGIISR	m5: Oxidation (+15.99), s10: Phospho (+79.97)	99.70%	4.87	30.8	7.66	3	907.43	2,719.27	-0.00208
245	IP	Hg	50 µM	261	286	IGAQmGHPGsPNAHPVTWsPGGIISR	m5: Oxidation (+15.99), s10: Phospho (+79.97), s19: Phospho (+79.97)	99.60%	4.29	3.55	0	3	934.09	2,799.24	0.00436
246	IP	Hg	50 µM	261	286	IGAQmGHPGSPNAHPVTWsPGGIISR	m5: Oxidation (+15.99), s19: Phospho (+79.97)	99.70%	4.66	4.73	2.89	3	907.43	2,719.28	0.00597
247	IP	Hg	50 µM	261	286	IGAQmGHPGsPNAHPVTWSPGGIISR	m5: Oxidation (+15.99), s10: Phospho (+79.97)	99.70%	4.26	46.9	3.39	3	907.43	2,719.27	-0.00044
248	IP	Hg	50 µM	261	286	IGAQmGHPGsPNAHPVTWSPGGIISR	m5: Oxidation (+15.99), s10: Phospho (+79.97)	99.70%	5.1	38.4	3.21	3	907.43	2,719.27	0.00231
249	IP	Hg	50 µM	261	286	IGAQmGHPGsPNAHPVTWSPGGIISR	m5: Oxidation (+15.99), s10: Phospho (+79.97)	99.70%	4.59	33.7	4.57	3	907.43	2,719.27	0.00378
250	IP	PV	0	261	286	IGAQmGHPGSPNAHPVTWsPGGIISR	m5: Oxidation (+15.99), s19: Phospho (+79.97)	97.00%	3.88	6.15	1.38	3	907.43	2,719.27	0.00488
251	IP	PV	0	261	286	IGAQmGHPGsPNAHPVTWSPGGIISR	m5: Oxidation (+15.99), s10: Phospho (+79.97)	99.20%	4.05	30.9	2.17	3	907.43	2,719.27	0.00359
252	IP	PV	0	261	286	IGAQmGHPGsPNAHPVTWSPGGIISR	m5: Oxidation (+15.99), s10: Phospho (+79.97)	99.70%	4.49	28.9	3.52	3	907.43	2,719.27	0.00359
253	IP	PV	0	261	286	IGAQmGHPGsPNAHPVTWSPGGIISR	m5: Oxidation (+15.99), s10: Phospho (+79.97)	99.70%	4.34	45.9	3.64	3	907.43	2,719.27	0.00304

254	IP	PV	0	261	286	IGAQmGHPGsPNAHPVTWSPGGIISR	m5: Oxidation (+15.99), s10: Phospho (+79.97)	99.70%	4.95	37.4	3.92	3	907.43	2,719.27	0.00085
255	PE	Ig	NT	261	286	IGAQMGHPGSPNAHPVTWsPGGIISR	s19: Phospho (+79.97)	99.70%	3.03	30.8	3.08	4	676.83	2,703.28	0.00948
256	PE	Ig	NT	261	286	IGAQMGHPGsPNAHPVTWSPGGIISR	s10: Phospho (+79.97)	99.70%	2.3	26.9	3.96	3	902.10	2,703.28	0.00754
257	PE	Ig	NT	261	286	IGAQMGHPGsPNAHPVTWSPGGIISR	s10: Phospho (+79.97)	99.70%	3.32	22	4.22	3	902.10	2,703.28	0.00480
258	PE	Ig	NT	261	286	IGAQMGHPGsPNAHPVTWSPGGIISR	s10: Phospho (+79.97)	99.70%	3.81	34.2	9.68	4	676.83	2,703.28	0.00362
259	PE	Ig	NT	261	286	IGAQMGHPGSPNAHPVTWsPGGIISR	s19: Phospho (+79.97)	99.50%	3.11	12.8	0	4	676.83	2,703.28	0.00533
260	PE	Ig	NT	261	286	IGAQMGHPGSPNAHPVTWsPGGIISR	s19: Phospho (+79.97)	99.70%	2.69	23.2	3.1	4	676.83	2,703.28	0.00948
261	PE	Ig	NT	261	286	IGAQmGHPGsPNAHPVTWSPGGIISR	m5: Oxidation (+15.99), s10: Phospho (+79.97)	99.70%	2.85	23	2.8	4	680.83	2,719.27	0.00431
262	PE	Ig	NT	261	286	IGAQmGHPGsPNAHPVTWSPGGIISR	m5: Oxidation (+15.99), s10: Phospho (+79.97)	99.70%	3.36	24.5	5.8	3	907.43	2,719.28	0.01037
263	PE	Ig	NT	261	286	IGAQMGHPGsPNAHPVTWSPGGIISR	s10: Phospho (+79.97)	99.70%	4	38.2	7.37	4	676.82	2,703.27	-0.00370
264	PE	Ig	NT	261	286	IGAQMGHPGsPNAHPVTWSPGGIISR	s10: Phospho (+79.97)	99.70%	6.56	33.8	9.13	3	902.10	2,703.27	-0.00033
265	PE	Ig	NT	261	286	IGAQMGHPGsPNAHPVTWSPGGIISR	s10: Phospho (+79.97)	99.70%	5.3	47.6	8.96	4	676.83	2,703.27	0.00045
266	PE	Ig	NT	261	286	IGAQMGHPGsPNAHPVTWSPGGIISR	s10: Phospho (+79.97)	99.70%	2.71	29.6	4.03	3	902.11	2,703.31	0.03757
267	PE	Ig	NT	261	286	IGAQMGHPGsPNAHPVTWSPGGIISR	s10: Phospho (+79.97)	99.70%	2.88	45.3	6.42	4	676.84	2,703.31	0.03731
268	PE	Ig	NT	261	286	IGAQMGHPGsPNAHPVTWSPGGIISR	s10: Phospho (+79.97)	99.70%	3.02	30.7	7.8	4	676.84	2,703.32	0.04244
269	PE	Ig	NT	261	286	IGAQMGHPGsPNAHPVTWSPGGIISR	s10: Phospho (+79.97)	99.70%	3.15	38.6	4.68	3	902.11	2,703.31	0.03647
270	PE	Ig	1 min	261	286	IGAQMGHPGsPNAHPVTWSPGGIISR	s10: Phospho (+79.97)	99.70%	3.17	29.2	7.27	4	676.83	2,703.27	-0.00004
271	PE	Ig	1 min	261	286	IGAQMGHPGsPNAHPVTWSPGGIISR	s10: Phospho (+79.97)	99.70%	3.5	54.3	10.2	4	676.83	2,703.27	-0.00297
272	PE	Ig	1 min	261	286	IGAQMGHPGSPNAHPVTWsPGGIISR	s19: Phospho (+79.97)	99.60%	2.86	16.9	1.52	4	676.82	2,703.27	-0.00541
273	PE	Ig	1 min	261	286	IGAQMGHPGsPNAHPVTWSPGGIISR	s10: Phospho (+79.97)	99.70%	3.63	24	6.7	3	902.10	2,703.28	0.00150
274	PE	Ig	1 min	261	286	IGAQMGHPGSPNAHPVtWSPGGIISR	t17: Phospho (+79.97)	99.70%	3.29	22.5	7.77	4	676.83	2,703.28	0.00460
275	PE	Ig	1 min	261	286	IGAQMGHPGsPNAHPVTWSPGGIISR	s10: Phospho (+79.97)	99.70%	4.51	33.5	8.28	4	676.83	2,703.27	-0.00004
276	PE	Ig	1 min	261	286	IGAQMGHPGsPNAHPVTWSPGGIISR	s10: Phospho (+79.97)	99.70%	6.89	44.6	8.68	3	902.10	2,703.27	0.00022
277	PE	Ig	1 min	261	286	IGAQMGHPGsPNAHPVTWSPGGIISR	s10: Phospho (+79.97)	99.70%	5.27	49.2	11.3	4	676.83	2,703.28	0.00631
278	PE	Ig	1 min	261	286	IGAQMGHPGsPNAHPVTWSPGGIISR	s10: Phospho (+79.97)	99.70%	5.69	35.9	7.51	3	902.10	2,703.27	0.00058
279	PE	Ig	1 min	261	286	IGAQMGHPGsPNAHPVTWSPGGIISR	s10: Phospho (+79.97)	99.70%	2.74	26.6	2.27	3	902.11	2,703.31	0.03336
280	PE	Ig	1 min	261	286	IGAQMGHPGsPNAHPVTWSPGGIISR	s10: Phospho (+79.97)	99.70%	2.63	17.7	3.8	3	902.11	2,703.30	0.02219
281	PE	Ig	1 min	261	286	IGAQmGHPGsPNAHPVTWSPGGIISR	m5: Oxidation (+15.99), s10: Phospho (+79.97)	99.70%	3.29	29.5	2.51	3	907.44	2,719.31	0.03838
282	PE	Ig	1 min	261	286	IGAQMGHPGsPNAHPVTWSPGGIISR	s10: Phospho (+79.97)	99.70%	2.92	26.2	3.8	3	902.11	2,703.32	0.04087
283	PE	Ig	1 min	261	286	IGAQMGHPGsPNAHPVTWSPGGIISR	s10: Phospho (+79.97)	99.70%	2.48	40.3	7.82	4	676.83	2,703.31	0.03414
284	PE	Ig	1 min	261	286	IGAQMGHPGsPNAHPVTWSPGGIISR	s10: Phospho (+79.97)	99.70%	3.1	23.8	8.59	4	676.83	2,703.31	0.03097
285	PE	Ig	1 min	261	286	IGAQMGHPGsPNAHPVTWSPGGIISR	s10: Phospho (+79.97)	99.70%	3.43	35.8	5.28	3	902.11	2,703.31	0.03373
286	PE	Ig	2 min	261	286	IGAQMGHPGSPNAHPVTWsPGGIISR	s19: Phospho (+79.97)	99.70%	2.78	24.8	2.52	4	676.83	2,703.28	0.00924
287	PE	Ig	2 min	261	286	IGAQMGHPGsPNAHPVTWSPGGIISR	s10: Phospho (+79.97)	99.70%	2.86	17.1	4.96	3	902.10	2,703.28	0.00296
288	PE	Ig	2 min	261	286	IGAQMGHPGsPNAHPVTWSPGGIISR	s10: Phospho (+79.97)	99.70%	3	27	5.24	3	902.10	2,703.28	0.00223
289	PE	Ig	2 min	261	286	IGAQMGHPGsPNAHPVTWSPGGIISR	s10: Phospho (+79.97)	99.70%	3.56	35.5	7.82	4	676.83	2,703.28	0.00362
290	PE	Ig	2 min	261	286	IGAQMGHPGsPNAHPVTWSPGGIISR	s10: Phospho (+79.97)	99.70%	4.3	39.1	9.14	4	676.83	2,703.27	0.00020
291	PE	Ig	2 min	261	286	IGAQMGHPGsPNAHPVTWSPGGIISR	s10: Phospho (+79.97)	99.70%	4.31	45.4	10.4	4	676.83	2,703.28	0.00265
292	PE	Ig	2 min	261	286	IGAQMGHPGSPNAHPVTWsPGGIISR	s19: Phospho (+79.97)	99.70%	3.34	19.3	2.92	3	902.10	2,703.27	-0.00656
293	PE	Ig	2 min	261	286	IGAQMGHPGSPNAHPVTWsPGGIISR	s19: Phospho (+79.97)	99.70%	2.75	21.5	0	4	676.83	2,703.28	0.00435
294	PE	Ig	2 min	261	286	IGAQMGHPGsPNAHPVTWSPGGIISR	s10: Phospho (+79.97)	99.70%	2.48	23.6	6.28	4	676.83	2,703.28	0.00362

295	PE	Ig	2 min	261	286	IGAQMGHPGsPNAHPVTWSPGGIISR	s10: Phospho (+79.97)	99.70%	5.14	37.1	9.85	4	676.83	2,703.28	0.00142
296	PE	Ig	2 min	261	286	IGAQMGHPGsPNAHPVTWSPGGIISR	s10: Phospho (+79.97)	99.70%	6.2	43.1	9.77	3	902.10	2,703.28	0.00095
297	PE	Ig	2 min	261	286	IGAQMGHPGsPNAHPVTWSPGGIISR	s10: Phospho (+79.97)	99.70%	5.78	38.3	7.44	3	902.10	2,703.27	0.00003
298	PE	Ig	2 min	261	286	IGAQMGHPGsPNAHPVTWSPGGIISR	s10: Phospho (+79.97)	99.70%	1.55	11.6	0	3	902.10	2,703.28	0.00461
299	PE	Ig	2 min	261	286	IGAQmGHPGsPnAHPVTWSPGGIISR	m5: Oxidation (+15.99), s10: Phospho (+79.97), n12: Deamidated (+0.98)	89.40%	0	0	3.74	3	907.78	2,720.31	0.05760
300	PE	Ig	2 min	261	286	IGAQMGHPGsPNAHPVTWSPGGIISR	s10: Phospho (+79.97)	99.70%	2.81	17.6	3.17	3	902.11	2,703.30	0.02256
301	PE	Ig	2 min	261	286	IGAQMGHPGsPNAHPVTWSPGGIISR	s10: Phospho (+79.97)	99.70%	2.71	16.6	2.89	3	902.11	2,703.30	0.02915
302	PE	Ig	2 min	261	286	IGAQMGHPGsPNAHPVTWSPGGIISR	s10: Phospho (+79.97)	99.70%	2.88	30	3.89	3	902.11	2,703.31	0.03281
303	PE	Ig	2 min	261	286	IGAQmGHPGsPNAHPVTWSPGGIISR	m5: Oxidation (+15.99), s10: Phospho (+79.97)	99.70%	3.11	21.1	5.52	4	680.83	2,719.31	0.03996
304	PE	Ig	2 min	261	286	IGAQMGHPGsPNAHPVTWSPGGIISR	s10: Phospho (+79.97)	99.70%	2.43	31.4	6.08	4	676.83	2,703.31	0.03634
305	PE	Ig	2 min	261	286	IGAQMGHPGsPNAHPVTWSPGGIISR	s10: Phospho (+79.97)	99.70%	3.09	45.3	6.8	4	676.84	2,703.31	0.03829
306	PE	Ig	2 min	261	286	IGAQMGHPGsPNAHPVTWSPGGIISR	s10: Phospho (+79.97)	99.70%	2.91	40.6	9.34	4	676.84	2,703.31	0.03853
307	PE	Ig	3 min	261	286	IGAQMGHPGsPNAHPVTWSPGGIISR	s10: Phospho (+79.97)	99.70%	3.71	0	4.34	3	902.10	2,703.28	0.00571
308	PE	Ig	3 min	261	286	IGAQMGHPGsPNAHPVTWSPGGIISR	s10: Phospho (+79.97)	99.70%	3.88	24	6.89	3	902.10	2,703.27	-0.00619
309	PE	Ig	3 min	261	286	IGAQMGHPGsPNAHPVTWSPGGIISR	s10: Phospho (+79.97)	99.70%	3.11	19.3	7.49	4	676.83	2,703.28	0.00265
310	PE	Ig	3 min	261	286	IGAQMGHPGsPNAHPVTWSPGGIISR	s10: Phospho (+79.97)	99.70%	3.14	30	7.59	4	676.83	2,703.28	0.00387
311	PE	Ig	3 min	261	286	IGAQMGHPGsPNAHPVTWSPGGIISR	s10: Phospho (+79.97)	99.70%	4.16	35.8	8.92	4	676.83	2,703.28	0.00362
312	PE	Ig	3 min	261	286	IGAQMGHPGSPNAHPVTWsPGGIISR	s19: Phospho (+79.97)	99.50%	2.49	0	2.55	3	902.10	2,703.27	-0.00729
313	PE	Ig	3 min	261	286	IGAQMGHPGSPNAHPVTWsPGGIISR	s19: Phospho (+79.97)	99.70%	3.16	28	1.68	4	676.83	2,703.27	-0.00297
314	PE	Ig	3 min	261	286	IGAQMGHPGsPNAHPVTWSPGGIISR	s10: Phospho (+79.97)	99.70%	3.21	18.4	4.11	3	902.10	2,703.27	-0.00235
315	PE	Ig	3 min	261	286	IGAQMGHPGsPNAHPVTWSPGGIISR	s10: Phospho (+79.97)	99.70%	1.98	19.6	6.85	4	676.83	2,703.28	0.00313
316	PE	Ig	3 min	261	286	IGAQMGHPGsPNAHPVTWSPGGIISR	s10: Phospho (+79.97)	99.70%	3.86	27.2	7.49	3	902.10	2,703.27	0.00003
317	PE	Ig	3 min	261	286	IGAQMGHPGsPNAHPVTWSPGGIISR	s10: Phospho (+79.97)	99.70%	4.05	38	7.11	4	676.83	2,703.28	0.00094
318	PE	Ig	3 min	261	286	IGAQMGHPGsPNAHPVTWSPGGIISR	s10: Phospho (+79.97)	99.70%	4.54	30.3	7.96	4	676.83	2,703.28	0.00338
319	PE	Ig	3 min	261	286	IGAQMGHPGsPNAHPVTWSPGGIISR	s10: Phospho (+79.97)	99.70%	5.6	41.6	8.29	3	902.10	2,703.27	-0.00326
320	PE	Ig	3 min	261	286	IGAQMGHPGsPNAHPVTWSPGGIISR	s10: Phospho (+79.97)	99.70%	5.13	30.6	6.64	3	902.10	2,703.28	0.00351
321	PE	Ig	3 min	261	286	IGAQMGHPGsPNAHPVTWSPGGIISR	s10: Phospho (+79.97)	99.70%	2.76	18	3.03	3	902.11	2,703.32	0.04123
322	PE	Ig	3 min	261	286	IGAQMGHPGsPNAHPVTWSPGGIISR	s10: Phospho (+79.97)	99.70%	3.72	28	4.2	3	902.11	2,703.31	0.03501
323	PE	Ig	3 min	261	286	IGAQMGHPGsPNAHPVTWSPGGIISR	s10: Phospho (+79.97)	99.70%	3.18	42	8.77	4	676.84	2,703.31	0.03951
324	PE	Ig	3 min	261	286	IGAQMGHPGsPNAHPVTWSPGGIISR	s10: Phospho (+79.97)	99.70%	3.75	39	5.51	3	902.11	2,703.31	0.03885
325	PE	Ig	5 min	261	286	IGAQMGHPGsPNAHPVTWSPGGIISR	s10: Phospho (+79.97)	99.70%	2.98	10.8	4.11	3	902.10	2,703.28	0.00699
326	PE	Ig	5 min	261	286	IGAQMGHPGsPNAHPVTWSPGGIISR	s10: Phospho (+79.97)	99.70%	3.58	20.6	6.8	3	902.10	2,703.28	0.00937
327	PE	Ig	5 min	261	286	IGAQMGHPGsPNAHPVTWSPGGIISR	s10: Phospho (+79.97)	99.70%	3.29	28.1	7.54	4	676.83	2,703.27	-0.00273
328	PE	Ig	5 min	261	286	IGAQMGHPGsPNAHPVTWSPGGIISR	s10: Phospho (+79.97)	99.70%	3.21	28.7	9.41	4	676.83	2,703.27	-0.00151
329	PE	Ig	5 min	261	286	IGAQMGHPGSPNAHPVTWsPGGIISR	s19: Phospho (+79.97)	88.10%	2.73	0	0	3	902.10	2,703.27	-0.00052
330	PE	Ig	5 min	261	286	IGAQMGHPGSPNAHPVTWsPGGIISR	s19: Phospho (+79.97)	99.70%	3.8	33	3.42	4	676.83	2,703.28	0.00167
331	PE	Ig	5 min	261	286	IGAQMGHPGsPNAHPVTWSPGGIISR	s10: Phospho (+79.97)	99.70%	2.52	26	4.6	4	676.83	2,703.28	0.00435
332	PE	Ig	5 min	261	286	IGAQMGHPGsPNAHPVTWSPGGIISR	s10: Phospho (+79.97)	99.70%	2.87	42	8.42	4	676.82	2,703.27	-0.00566
333	PE	Ig	5 min	261	286	IGAQMGHPGsPNAHPVTWSPGGIISR	s10: Phospho (+79.97)	99.70%	3.99	20.5	8.92	3	902.10	2,703.27	-0.00271
334	PE	Ig	5 min	261	286	IGAQMGHPGsPNAHPVTWSPGGIISR	s10: Phospho (+79.97)	99.70%	4.19	38.6	9.89	4	676.83	2,703.28	0.00435
335	PE	Ig	5 min	261	286	IGAQMGHPGsPNAHPVTWSPGGIISR	s10: Phospho (+79.97)	99.70%	4.46	38.7	7.11	3	902.10	2,703.28	0.00388
336	PE	Ig	5 min	261	286	IGAQMGHPGsPNAHPVTWSPGGIISR	s10: Phospho (+79.97)	99.30%	1.66	44.1	0	4	676.83	2,703.28	0.00899

337	PE	Ig	5 min	261	286	IGAQMGHPGsPNAHPVTWSPGGIISR	s10: Phospho (+79.97)	99.70%	1.83	31.4	0	3	902.10	2,703.28	0.00718
338	PE	Ig	5 min	261	286	IGAQmGHPGsPNAHPVTWSPGGIISR	m5: Oxidation (+15.99), s10: Phospho (+79.97)	93.70%	2.66	22	0	3	907.44	2,719.31	0.04333
339	PE	Ig	5 min	261	286	IGAQMGHPGSPNAHPVTWsPGGIISR	s19: Phospho (+79.97)	99.40%	2.36	17	0	4	676.84	2,703.32	0.04677
340	PE	Ig	5 min	261	286	IGAQMGHPGsPNAHPVTWSPGGIISR	s10: Phospho (+79.97)	99.70%	2.59	30.8	3.72	3	902.11	2,703.31	0.03757
341	PE	Ig	5 min	261	286	IGAQMGHPGsPNAHPVTWSPGGIISR	s10: Phospho (+79.97)	99.70%	3.16	26	3.92	3	902.11	2,703.31	0.03977
342	PE	Ig	5 min	261	286	IGAQMGHPGsPNAHPVTWSPGGIISR	s10: Phospho (+79.97)	99.70%	2.84	20.6	4.7	3	902.11	2,703.32	0.04801
343	PE	Ig	5 min	261	286	IGAQMGHPGsPNAHPVTWSPGGIISR	s10: Phospho (+79.97)	99.70%	2.16	26.3	4.89	3	902.11	2,703.32	0.04544
344	PE	Ig	5 min	261	286	IGAQMGHPGsPNAHPVTWSPGGIISR	s10: Phospho (+79.97)	99.70%	2.77	26	7.06	3	902.11	2,703.31	0.03336
345	PE	Ig	15 min	261	286	IGAQMGHPGsPNAHPVTWSPGGIISR	s10: Phospho (+79.97)	99.30%	3.21	0	2.49	3	902.10	2,703.28	0.00919
346	PE	Ig	15 min	261	286	IGAQMGHPGsPNAHPVTWSPGGIISR	s10: Phospho (+79.97)	99.70%	3.05	28.1	7.34	4	676.83	2,703.28	0.00069
347	PE	Ig	15 min	261	286	IGAQMGHPGsPNAHPVTWSPGGIISR	s10: Phospho (+79.97)	99.70%	4.19	16.7	7.28	3	902.09	2,703.25	-0.02121
348	PE	Ig	15 min	261	286	IGAQMGHPGsPNAHPVTWSPGGIISR	s10: Phospho (+79.97)	99.70%	3.73	54.1	7.03	4	676.83	2,703.29	0.01436
349	PE	Ig	15 min	261	286	IGAQMGHPGsPNAHPVTWSPGGIISR	s10: Phospho (+79.97)	99.70%	5.08	50.9	10.5	4	676.83	2,703.28	0.00387
350	PE	Ig	15 min	261	286	IGAQMGHPGsPNAHPVTWSPGGIISR	s10: Phospho (+79.97)	99.70%	3.39	29.1	10.8	4	676.83	2,703.27	-0.00151
351	PE	Ig	15 min	261	286	IGAQMGHPGsPNAHPVTWSPGGIISR	s10: Phospho (+79.97)	99.70%	5.84	38.2	7.85	3	902.10	2,703.27	0.00022
352	PE	Ig	15 min	261	286	IGAQMGHPGsPNAHPVTWSPGGIISR	s10: Phospho (+79.97)	99.10%	0.74	32.8	0	4	676.83	2,703.28	0.00533
353	PE	Ig	15 min	261	286	IGAQmGHPGsPNAHPVTWSPGGIISR	m5: Oxidation (+15.99), s10: Phospho (+79.97)	99.70%	2.41	15.3	3.04	3	907.45	2,719.32	0.04717
354	PE	Ig	15 min	261	286	IGAQMGHPGsPNAHPVTWSPGGIISR	s10: Phospho (+79.97)	99.70%	2.92	17.5	2.66	3	902.11	2,703.32	0.04087
355	PE	Ig	15 min	261	286	IGAQmGHPGsPNAHPVTWSPGGIISR	m5: Oxidation (+15.99), s10: Phospho (+79.97)	99.70%	2.72	16	3.77	4	680.84	2,719.32	0.04777
356	PE	Ig	15 min	261	286	IGAQMGHPGsPNAHPVTWSPGGIISR	s10: Phospho (+79.97)	99.70%	1.82	15.3	3.6	4	676.83	2,703.31	0.03463
357	PE	Ig	15 min	261	286	IGAQMGHPGsPNAHPVTWSPGGIISR	s10: Phospho (+79.97)	99.70%	3.93	33.3	5.31	3	902.11	2,703.31	0.03757
358	PE	Ig	15 min	261	286	IGAQMGHPGsPNAHPVTWSPGGIISR	s10: Phospho (+79.97)	99.70%	2.46	32.7	5.52	4	676.84	2,703.31	0.04049
359	PE	Ig	15 min	261	286	IGAQMGHPGsPNAHPVTWSPGGIISR	s10: Phospho (+79.97)	99.70%	1.98	27	6.35	4	676.83	2,703.31	0.03609
360	PE	Ig	15 min	261	286	IGAQMGHPGsPNAHPVTWSPGGIISR	s10: Phospho (+79.97)	99.70%	2.65	38.6	4.7	3	902.11	2,703.32	0.04416
361	PE	Ig	15 min	261	286	IGAQMGHPGsPNAHPVTWSPGGIISR	s10: Phospho (+79.97)	99.70%	3.18	59.3	6.59	3	902.11	2,703.31	0.03464
362	PE	Hg	0	261	286	IGAQMGHPGsPNAHPVTWSPGGIISR	s10: Phospho (+79.97)	99.40%	2.87	0	4.82	3	902.10	2,703.28	0.00718
363	PE	Hg	0	261	286	IGAQMGHPGsPNAHPVTWSPGGIISR	s10: Phospho (+79.97)	99.70%	3.6	37.2	8.15	4	676.83	2,703.29	0.01143
364	PE	Hg	0	261	286	IGAQMGHPGsPNAHPVTWSPGGIISR	s10: Phospho (+79.97)	96.10%	2.06	0	3.54	3	902.10	2,703.28	0.00068
365	PE	Hg	0	261	286	IGAQmGHPGsPNAHPVTWSPGGIISR	m5: Oxidation (+15.99), s10: Phospho (+79.97)	99.70%	3.1	36.6	7.24	4	680.83	2,719.27	0.00212
366	PE	Hg	0	261	286	IGAQMGHPGsPNAHPVTWSPGGIISR	s10: Phospho (+79.97)	99.70%	5.57	43.1	7.44	3	902.10	2,703.27	-0.00125
367	PE	Hg	0	261	286	IGAQMGHPGsPNAHPVTWSPGGIISR	s10: Phospho (+79.97)	99.70%	3.31	37.8	9.09	4	676.83	2,703.28	0.00265
368	PE	Hg	0	261	286	IGAQMGHPGsPNAHPVTWSPGGIISR	s10: Phospho (+79.97)	99.70%	3.23	39.1	8.92	4	676.83	2,703.27	-0.00175
369	PE	Hg	0	261	286	IGAQMGHPGsPNAHPVTWSPGGIISR	s10: Phospho (+79.97)	84.20%	0	10.3	0	3	902.10	2,703.28	0.00718
370	PE	Hg	0	261	286	IGAQMGHPGsPNAHPVTWSPGGIISR	s10: Phospho (+79.97)	99.30%	0.5	19.6	0	4	676.83	2,703.28	0.00997
371	PE	Hg	1 μM	261	286	IGAQMGHPGsPNAHPVTWSPGGIISR	s10: Phospho (+79.97)	99.70%	3.3	11.8	4.23	3	902.10	2,703.28	0.00809
372	PE	Hg	1 μM	261	286	IGAQMGHPGsPNAHPVTWSPGGIISR	s10: Phospho (+79.97)	99.70%	2.91	52.2	10.8	4	676.83	2,703.27	-0.00053
373	PE	Hg	1 μM	261	286	IGAQMGHPGsPNAHPVTWSPGGIISR	s10: Phospho (+79.97)	99.70%	4.66	40.8	11.2	4	676.83	2,703.28	0.00728
374	PE	Hg	1 μM	261	286	IGAQMGHPGsPNAHPVTWSPGGIISR	s10: Phospho (+79.97)	97.00%	3.41	0	3.7	3	902.10	2,703.27	-0.00747
375	PE	Hg	1 μM	261	286	IGAQMGHPGsPNAHPVTWSPGGIISR	s10: Phospho (+79.97)	99.70%	2.49	25.4	4.89	3	902.10	2,703.27	0.00003
376	PE	Hg	1 μM	261	286	IGAQMGHPGsPNAHPVTWSPGGIISR	s10: Phospho (+79.97)	99.70%	4.65	31.1	6.89	3	902.10	2,703.27	-0.00638
377	PE	Hg	1 μM	261	286	IGAQMGHPGsPNAHPVTWSPGGIISR	s10: Phospho (+79.97)	99.70%	2.98	36.5	9.22	4	676.83	2,703.27	-0.00199

378	PE	Hg	1 µM	261	286	IGAQMGHPGsPNAHPVTWSPGGIISR	s10: Phospho (+79.97)	99.70%	3.5	43.8	10.9	4	676.83	2,703.27	-0.00004
379	PE	Hg	5 µM	261	286	IGAQMGHPGsPNAHPVTWSPGGIISR	s10: Phospho (+79.97)	99.70%	3.72	20	6.14	3	902.10	2,703.28	0.00406
380	PE	Hg	5 µM	261	286	IGAQMGHPGsPNAHPVTWSPGGIISR	s10: Phospho (+79.97)	99.70%	4	47.2	10.5	4	676.83	2,703.27	0.00020
381	PE	Hg	5 µM	261	286	IGAQMGHPGsPNAHPVTWSPGGIISR	s10: Phospho (+79.97)	99.70%	3.92	52	10.3	4	676.83	2,703.28	0.01021
382	PE	Hg	5 µM	261	286	IGAQmGHPGsPNAHPVTWSPGGIISR	m5: Oxidation (+15.99), s10: Phospho (+79.97)	99.30%	1.65	19.7	2.89	3	907.43	2,719.28	0.01366
383	PE	Hg	5 µM	261	286	IGAQMGHPGsPNAHPVTWSPGGIISR	s10: Phospho (+79.97)	99.70%	3.78	34.8	8.12	4	676.82	2,703.27	-0.00614
384	PE	Hg	5 µM	261	286	IGAQMGHPGsPNAHPVTWSPGGIISR	s10: Phospho (+79.97)	99.70%	2.92	36.9	9.32	4	676.82	2,703.27	-0.00737
385	PE	Hg	5 µM	261	286	IGAQMGHPGsPNAHPVTWSPGGIISR	s10: Phospho (+79.97)	92.70%	0.98	11.9	0	3	902.10	2,703.29	0.01175
386	PE	Hg	10 µM	261	286	IGAQMGHPGsPNAHPVTWSPGGIISR	s10: Phospho (+79.97)	99.70%	3.75	18.8	4.17	3	902.10	2,703.28	0.00754
387	PE	Hg	10 µM	261	286	IGAQMGHPGsPNAHPVTWSPGGIISR	s10: Phospho (+79.97)	99.70%	3.3	28.3	8.44	4	676.83	2,703.28	0.00924
388	PE	Hg	10 µM	261	286	IGAQMGHPGsPNAHPVTWSPGGIISR	s10: Phospho (+79.97)	99.70%	4.2	39.8	8.28	4	676.83	2,703.28	0.00240
389	PE	Hg	10 µM	261	286	IGAQMGHPGsPNAHPVTWSPGGIISR	s10: Phospho (+79.97)	99.70%	4.62	50.2	11.6	4	676.83	2,703.27	-0.00028
390	PE	Hg	10 µM	261	286	IGAQMGHPGsPNAHPVTWSPGGIISR	s10: Phospho (+79.97)	99.70%	3.42	35.2	7.66	3	902.10	2,703.28	0.00370
391	PE	Hg	10 µM	261	286	IGAQMGHPGsPNAHPVTWSPGGIISR	s10: Phospho (+79.97)	99.50%	0	0	4.29	3	902.77	2,705.28	2.00200
392	PE	Hg	10 µM	261	286	IGAQMGHPGsPNAHPVTWSPGGIISR	s10: Phospho (+79.97)	99.70%	3.44	43.2	7.33	4	676.82	2,703.27	-0.00444
393	PE	Hg	10 µM	261	286	IGAQMGHPGsPNAHPVTWSPGGIISR	s10: Phospho (+79.97)	99.70%	2.99	39.8	9.15	4	676.83	2,703.27	-0.00346
394	PE	Hg	20 µM	261	286	IGAQMGHPGsPNAHPVTWSPGGIISR	s10: Phospho (+79.97)	99.70%	3.37	20.7	4.23	3	902.10	2,703.28	0.00516
395	PE	Hg	20 µM	261	286	IGAQMGHPGsPNAHPVTWSPGGIISR	s10: Phospho (+79.97)	99.70%	4.08	53.7	8.85	4	676.83	2,703.29	0.01388
396	PE	Hg	20 µM	261	286	IGAQMGHPGsPNAHPVTWSPGGIISR	s10: Phospho (+79.97)	99.70%	3.68	42.8	9.04	4	676.83	2,703.28	0.00850
397	PE	Hg	20 µM	261	286	IGAQMGHPGsPNAHPVTWSPGGIISR	s10: Phospho (+79.97)	99.70%	3.25	17.2	2.42	3	902.10	2,703.27	-0.00180
398	PE	Hg	20 µM	261	286	IGAQMGHPGsPNAHPVTWSPGGIISR	s10: Phospho (+79.97)	99.70%	3.72	34.9	8.92	4	676.82	2,703.26	-0.01054
399	PE	Hg	20 µM	261	286	IGAQMGHPGsPNAHPVTWSPGGIISR	s10: Phospho (+79.97)	99.70%	3.26	27.2	9.38	4	676.83	2,703.27	0.00045
400	PE	Hg	20 µM	261	286	IGAQMGHPGsPNAHPVTWSPGGIISR	s10: Phospho (+79.97)	99.70%	4.62	38.3	5.89	3	902.10	2,703.27	-0.00143
401	PE	Hg	20 µM	261	286	IGAQMGHPGsPNAHPVTWSPGGIISR	s10: Phospho (+79.97)	99.70%	1.26	36.4	3.1	4	676.83	2,703.28	0.01046
402	PE	Hg	20 µM	261	286	IGAQMGHPGsPNAHPVTWSPGGIISR	s10: Phospho (+79.97)	99.70%	1.31	31.4	0.96	3	902.10	2,703.29	0.01596
403	PE	Hg	50 µM	261	286	IGAQMGHPGsPNAHPVTWSPGGIISR	s10: Phospho (+79.97)	99.70%	4.11	36.2	7.64	4	676.83	2,703.28	0.00631
404	PE	Hg	50 µM	261	286	IGAQMGHPGsPNAHPVTWSPGGIISR	s10: Phospho (+79.97)	99.70%	3.96	29.6	9.26	4	676.83	2,703.28	0.00094
405	PE	Hg	50 µM	261	286	IGAQMGHPGsPNAHPVTWSPGGIISR	s10: Phospho (+79.97)	99.70%	3.84	16.3	5.21	3	902.10	2,703.27	-0.00015
406	PE	Hg	50 µM	261	286	IGAQMGHPGsPNAHPVTWSPGGIISR	s10: Phospho (+79.97)	99.70%	4.4	26.2	5.64	3	902.10	2,703.27	-0.00125
407	PE	Hg	50 µM	261	286	IGAQMGHPGsPNAHPVTWSPGGIISR	s10: Phospho (+79.97)	99.70%	3.26	45.3	11.9	4	676.83	2,703.27	-0.00077
408	PE	Hg	50 µM	261	286	IGAQMGHPGsPNAHPVTWSPGGIISR	s10: Phospho (+79.97)	99.70%	0.77	25.1	1.15	3	902.10	2,703.29	0.01120
409	PE	Hg	100 µM	261	286	IGAQMGHPGsPNAHPVTWSPGGIISR	s10: Phospho (+79.97)	99.70%	0	33.3	7.34	4	676.83	2,703.28	0.00631
410	PE	Hg	100 µM	261	286	IGAQMGHPGsPNAHPVTWSPGGIISR	s10: Phospho (+79.97)	99.70%	3.31	15.9	5.96	3	902.10	2,703.27	-0.00052
411	PE	Hg	100 µM	261	286	IGAQMGHPGsPNAHPVTWSPGGIISR	s10: Phospho (+79.97)	99.70%	1.99	28.1	8.32	4	676.82	2,703.27	-0.00395
412	PE	Hg	100 µM	261	286	IGAQMGHPGsPNAHPVTWSPGGIISR	s10: Phospho (+79.97)	99.70%	2.71	39.3	10.6	4	676.82	2,703.27	-0.00932
413	PE	PV	0	261	286	IGAQMGHPGsPNAHPVTWsPGGIISR	s10: Phospho (+79.97), s19: Phospho (+79.97)	99.20%	3.16	25.8	2.08	3	928.75	2,783.24	-0.00469
414	PE	PV	0	261	286	IGAQMGHPGsPNAHPVTWSPGGIISR	s10: Phospho (+79.97)	99.70%	4.61	0	3.41	3	902.10	2,703.27	-0.00161
415	PE	PV	0	261	286	IGAQMGHPGsPNAHPVTWSPGGIISR	s10: Phospho (+79.97)	99.70%	3.36	20.8	7.68	4	676.82	2,703.27	-0.00590
416	PE	PV	0	261	286	IGAQMGHPGsPNAHPVTWSPGGIISR	s10: Phospho (+79.97)	99.70%	4.99	41.2	10.5	4	676.83	2,703.27	-0.00077
417	PE	PV	0	261	286	IGAQMGHPGSPNAHPVTWsPGGIISR	s19: Phospho (+79.97)	99.70%	3.13	16.9	1.42	3	902.10	2,703.27	0.00003
418	PE	PV	0	261	286	IGAQMGHPGsPNAHPVTWSPGGIISR	s10: Phospho (+79.97)	99.70%	5.75	31.8	6.39	3	902.10	2,703.28	0.00150
419	PE	PV	1 µM	261	286	IGAQMGHPGsPNAHPVTWSPGGIISR	s10: Phospho (+79.97)	99.70%	2.7	22.2	7.13	4	676.83	2,703.28	0.00435
420	PE	PV	1 µM	261	286	IGAQMGHPGSPNAHPVTWsPGGIISR	s19: Phospho (+79.97)	99.40%	1.86	10.4	0	4	676.83	2,703.28	0.00435

421	PE	PV	1 μM	261	286	IGAQMGHPGsPNAHPVTWSPGGIISR	s10: Phospho (+79.97)	99.70%	4.48	50.3	10.8	4	676.83	2,703.28	0.00655
422	PE	PV	3 μM	261	286	IGAQMGHPGsPNAHPVTWSPGGIISR	s10: Phospho (+79.97)	99.70%	2.39	12.2	5.12	3	902.10	2,703.28	0.00296
423	PE	PV	3 μM	261	286	IGAQMGHPGsPNAHPVTWSPGGIISR	s10: Phospho (+79.97)	99.70%	2.79	25.5	6.77	4	676.83	2,703.28	0.00655
424	PE	PV	3 μM	261	286	IGAQMGHPGsPNAHPVTWSPGGIISR	s10: Phospho (+79.97)	99.70%	3.82	32.7	10.2	4	676.83	2,703.28	0.00460
425	PE	PV	10 μM	261	286	IGAQMGHPGsPNAHPVTWSPGGIISR	s10: Phospho (+79.97)	99.70%	3.69	25.2	6.8	3	902.10	2,703.28	0.00919
426	PE	PV	10 μM	261	286	IGAQMGHPGsPNAHPVTWSPGGIISR	s10: Phospho (+79.97)	99.70%	4.09	26	6.92	4	676.83	2,703.28	0.00411
427	PE	PV	10 μM	261	286	IGAQMGHPGsPNAHPVTWSPGGIISR	s10: Phospho (+79.97)	99.70%	2.35	26	8.48	4	676.83	2,703.28	0.00094
428	PE	PV	10 μM	261	286	IGAQMGHPGsPNAHPVTWSPGGIISR	s10: Phospho (+79.97)	99.70%	2.97	26.4	9.31	4	676.83	2,703.28	0.00094
429	PE	PV	30 μM	261	286	IGAQMGHPGsPNAHPVTWSPGGIISR	s10: Phospho (+79.97)	99.70%	2.82	16.2	5.77	3	902.10	2,703.28	0.00480
430	PE	PV	30 μM	261	286	IGAQMGHPGsPNAHPVTWSPGGIISR	s10: Phospho (+79.97)	99.70%	3.76	29.8	5.77	3	902.10	2,703.28	0.00498
431	PE	PV	30 μM	261	286	IGAQMGHPGsPNAHPVTWSPGGIISR	s10: Phospho (+79.97)	99.70%	2.4	17.4	5.96	4	676.83	2,703.28	0.00704
432	PE	PV	30 μM	261	286	IGAQMGHPGsPNAHPVTWSPGGIISR	s10: Phospho (+79.97)	99.70%	2.76	0	7.06	3	902.10	2,703.28	0.00461
433	PE	PV	30 μM	261	286	IGAQMGHPGsPNAHPVTWSPGGIISR	s10: Phospho (+79.97)	99.70%	4.09	24.3	6.89	4	676.83	2,703.28	0.00728
434	PE	PV	30 μM	261	286	IGAQMGHPGsPNAHPVTWSPGGIISR	s10: Phospho (+79.97)	99.70%	3.61	31.8	7.7	4	676.83	2,703.27	-0.00151
435	PE	PV	30 μM	261	286	IGAQMGHPGsPNAHPVTWSPGGIISR	s10: Phospho (+79.97)	99.70%	5.06	53.8	10.2	4	676.83	2,703.27	-0.00151
436	PE	PV	30 μM	261	286	IGAQMGHPGsPNAHPVTWSPGGIISR	s10: Phospho (+79.97)	99.70%	3.77	38.8	11.1	4	676.83	2,703.27	-0.00004
437	PE	PV	30 μM	261	286	IGAQMGHPGsPNAHPVTWSPGGIISR	s10: Phospho (+79.97)	99.70%	0.94	24.2	1.33	3	902.10	2,703.28	0.00205
438	PE	PV	100 μM	261	286	IGAQMGHPGsPNAHPVTWSPGGIISR	s10: Phospho (+79.97)	99.70%	4.39	35.4	5.82	3	902.10	2,703.28	0.00406
439	PE	PV	100 μM	261	286	IGAQMGHPGsPNAHPVTWSPGGIISR	s10: Phospho (+79.97)	99.70%	4.1	52.1	9.35	4	676.83	2,703.27	-0.00126
440	PE	PV	100 μM	261	286	IGAQmGHPGsPNAHPVTWSPGGIISR	m5: Oxidation (+15.99), s10: Phospho (+79.97)	99.70%	4.63	25.2	9.47	3	907.43	2,719.26	-0.00758
441	PE	PV	100 μM	261	286	IGAQmGHPGsPNAHPVTWSPGGIISR	m5: Oxidation (+15.99), s10: Phospho (+79.97)	99.70%	3.6	31.6	9.47	4	680.82	2,719.27	-0.00228
442	IP	PV	0	287	298	IKSySFPKPGHK	y4: Phospho (+79.97)	99.70%	1.45	21.4	2.02	3	490.25	1,467.73	-0.00127
443	PE	Ig	NT	287	298	IKSYsFPKPGHK	s5: Phospho (+79.97)	96.90%	1.68	17	0	3	490.25	1,467.72	-0.00457
444	PE	Ig	2 min	287	298	IKSySFPKPGHK	y4: Phospho (+79.97)	99.70%	2.4	29.5	0	3	490.25	1,467.73	-0.00191
445	PE	Ig	2 min	287	298	IKSysFPKPGHK	y4: Phospho (+79.97), s5: Phospho (+79.97)	99.70%	1.7	19.7	0	3	516.91	1,547.70	0.00307
446	PE	Ig	3 min	287	298	IKSysFPKPGHK	y4: Phospho (+79.97), s5: Phospho (+79.97)	97.90%	1.46	13.4	0	3	516.91	1,547.70	0.00124
447	PE	Ig	3 min	287	298	IKSySFPKPGHK	y4: Phospho (+79.97)	99.70%	2.56	22	0	3	490.25	1,467.72	-0.00502
448	PE	Ig	5 min	287	298	IKSySFPKPGHK	y4: Phospho (+79.97)	85.30%	1.57	14.5	0	3	490.25	1,467.72	-0.00292
449	PE	Hg	10 μM	287	298	IKSySFPKPGHK	y4: Phospho (+79.97)	99.70%	1.82	19.6	2	3	490.25	1,467.72	-0.00457
450	PE	Hg	50 μM	287	298	IKSysFPKPGHK	y4: Phospho (+79.97), s5: Phospho (+79.97)	99.70%	1.81	16.3	0	3	516.91	1,547.70	0.00289
451	PE	PV	10 μM	287	298	IKSysFPKPGHK	y4: Phospho (+79.97), s5: Phospho (+79.97)	99.70%	3	22.2	1.2	3	516.91	1,547.70	0.00380
452	PE	PV	10 μM	287	298	IKsYsFPKPGHK	s3: Phospho (+79.97), s5: Phospho (+79.97)	99.70%	2.68	11.2	1.55	3	516.91	1,547.70	0.00399
453	PE	PV	10 μM	287	298	IKSysFPKPGHK	y4: Phospho (+79.97), s5: Phospho (+79.97)	99.70%	3.13	23	0	3	516.91	1,547.70	0.00435
454	PE	PV	10 μM	287	298	IKsYsFPKPGHK	s3: Phospho (+79.97), s5: Phospho (+79.97)	99.70%	2.87	17.7	0	3	516.91	1,547.70	0.00271
455	PE	PV	10 μM	287	298	IKSysFPKPGHK	y4: Phospho (+79.97), s5: Phospho (+79.97)	99.70%	3.17	15.2	0	3	516.91	1,547.70	0.00380

456	PE	PV	10 μM	287	298	IKSysFPKPGHK	y4: Phospho (+79.97), s5: Phospho (+79.97)	99.70%	3.8	25.5	0	3	516.91	1,547.70	0.00325
457	PE	PV	10 μM	287	298	IKsYsFPKPGHK	s3: Phospho (+79.97), s5: Phospho (+79.97)	87.20%	2.73	0	0	3	516.91	1,547.70	0.00142
458	PE	PV	10 μM	287	298	IKsYsFPKPGHK	s3: Phospho (+79.97), s5: Phospho (+79.97)	95.90%	3.16	0	0	3	516.91	1,547.70	0.00124
459	PE	PV	10 μM	287	298	IKSysFPKPGHK	y4: Phospho (+79.97), s5: Phospho (+79.97)	99.50%	1.98	11.5	0	3	516.91	1,547.69	-0.00023
460	PE	PV	10 μM	287	298	IKSysFPKPGHK	y4: Phospho (+79.97), s5: Phospho (+79.97)	99.70%	2.02	14.4	0	3	516.91	1,547.69	-0.00004
461	PE	PV	10 μM	287	298	IKsYSFPKPGHK	s3: Phospho (+79.97)	97.00%	0.5	19.3	0	2	734.87	1,467.73	0.00323
462	PE	PV	10 μM	287	298	IKsysFPKPGHK	s3: Phospho (+79.97), y4: Phospho (+79.97), s5: Phospho (+79.97)	98.60%	0.92	13.8	0	2	814.84	1,627.66	0.00440
463	PE	PV	10 μM	287	298	IKsYsFPKPGHK	s3: Phospho (+79.97), s5: Phospho (+79.97)	99.70%	1.22	0	0.96	3	516.91	1,547.70	0.00563
464	PE	PV	10 μM	287	298	IKsysFPKPGHK	s3: Phospho (+79.97), y4: Phospho (+79.97), s5: Phospho (+79.97)	99.70%	1.73	11.7	0	3	543.56	1,627.67	0.00695
465	PE	PV	10 μM	287	298	IKSysFPKPGHK	y4: Phospho (+79.97), s5: Phospho (+79.97)	99.70%	1.35	0	1.2	3	516.91	1,547.70	0.00344
466	PE	PV	10 μM	287	298	IKsysFPKPGHK	s3: Phospho (+79.97), y4: Phospho (+79.97), s5: Phospho (+79.97)	99.70%	1.85	11.8	1.54	3	543.56	1,627.67	0.00622
467	PE	PV	10 μM	287	298	IKSySFPKPGHK	y4: Phospho (+79.97)	99.70%	2.55	59.7	6.17	2	734.87	1,467.73	0.00006
468	PE	PV	10 μM	287	298	IKsYSFPKPGHK	s3: Phospho (+79.97)	99.70%	2.35	17.2	2.1	3	490.25	1,467.73	0.00230
469	PE	PV	10 μM	287	298	IKSysFPKPGHK	y4: Phospho (+79.97), s5: Phospho (+79.97)	99.70%	2.53	60.4	2.66	2	774.86	1,547.70	0.00296
470	PE	PV	10 μM	287	298	IKsYsFPKPGHK	s3: Phospho (+79.97), s5: Phospho (+79.97)	99.70%	2.89	19	1.64	3	516.91	1,547.70	0.00948
471	PE	PV	10 μM	287	298	IKSysFPKPGHK	y4: Phospho (+79.97), s5: Phospho (+79.97)	99.70%	3.4	32	1.85	3	516.91	1,547.70	0.00692
472	PE	PV	10 μM	287	298	IKSySFPKPGHK	y4: Phospho (+79.97)	99.70%	3.02	32.4	3.27	3	490.25	1,467.73	0.00111
473	PE	PV	10 μM	287	298	IKSysFPKPGHK	y4: Phospho (+79.97), s5: Phospho (+79.97)	98.60%	1.61	13.2	0	2	774.87	1,547.72	0.02542
474	PE	PV	10 μM	287	298	IKSysFPKPGHK	y4: Phospho (+79.97), s5: Phospho (+79.97)	99.50%	2.52	16.9	0	3	516.91	1,547.72	0.02431
475	PE	PV	10 μM	287	298	IKsYsFPKPGHK	s3: Phospho (+79.97), s5: Phospho (+79.97)	99.70%	2.79	23.7	0	3	516.91	1,547.72	0.02578
476	PE	PV	10 μM	287	298	IKsysFPKPGHK	s3: Phospho (+79.97), y4: Phospho (+79.97), s5: Phospho (+79.97)	84.80%	1.12	19.9	0	3	543.56	1,627.66	0.00219
477	PE	PV	10 μM	287	298	IKSySFPKPGHK	y4: Phospho (+79.97)	99.70%	0.76	20.3	2.46	2	734.87	1,467.73	0.00115
478	PE	PV	10 μM	287	298	IKSysFPKPGHK	y4: Phospho (+79.97), s5: Phospho (+79.97)	99.70%	2.5	43.9	1.42	2	774.86	1,547.70	0.00223

479	PE	PV	30 µM	287	298	IKsYsFPKPGHK	s3: Phospho (+79.97), s5: Phospho (+79.97)	82.80%	1.48	13.2	0	3	516.91	1,547.70	0.00234
480	PE	PV	30 µM	287	298	IKsYsFPKPGHK	s3: Phospho (+79.97), s5: Phospho (+79.97)	91.00%	2.23	0	0	3	516.91	1,547.70	0.00289
481	PE	PV	30 µM	287	298	IKSysFPKPGHK	y4: Phospho (+79.97), s5: Phospho (+79.97)	95.00%	1.29	17	0.59	3	516.91	1,547.70	0.00179
482	PE	PV	30 µM	287	298	IKsYsFPKPGHK	s3: Phospho (+79.97), s5: Phospho (+79.97)	99.70%	2.44	14.9	1.55	3	516.91	1,547.70	0.00289
483	PE	PV	30 µM	287	298	IKSysFPKPGHK	y4: Phospho (+79.97), s5: Phospho (+79.97)	99.70%	2.35	17.5	1.57	3	516.91	1,547.70	0.00179
484	PE	PV	30 µM	287	298	IKSYsFPKPGHK	s5: Phospho (+79.97)	97.90%	1.72	14.8	1.35	3	490.25	1,467.73	0.00615
485	PE	PV	30 µM	287	298	IKSySFPKPGHK	y4: Phospho (+79.97)	99.30%	1.88	19.9	2.77	3	490.25	1,467.73	0.00560
486	PE	PV	30 µM	287	298	IKSysFPKPGHK	y4: Phospho (+79.97), s5: Phospho (+79.97)	99.70%	2.71	14.8	0	3	516.91	1,547.70	0.00325
487	PE	PV	30 µM	287	298	IKSysFPKPGHK	y4: Phospho (+79.97), s5: Phospho (+79.97)	99.70%	3.23	21.8	0	3	516.91	1,547.70	0.00472
488	PE	PV	30 µM	287	298	IKSysFPKPGHK	y4: Phospho (+79.97), s5: Phospho (+79.97)	99.70%	3.24	21.9	1.41	3	516.91	1,547.70	0.00362
489	PE	PV	30 µM	287	298	IKsYsFPKPGHK	s3: Phospho (+79.97), s5: Phospho (+79.97)	99.70%	3.04	11.2	0	3	516.91	1,547.70	0.00234
490	PE	PV	30 µM	287	298	IKsYsFPKPGHK	s3: Phospho (+79.97), s5: Phospho (+79.97)	99.70%	3.34	20	0	3	516.91	1,547.70	0.00527
491	PE	PV	30 µM	287	298	IKSYsFPKPGHK	s5: Phospho (+79.97)	99.60%	2.02	16.4	0	3	490.25	1,467.73	0.00157
492	PE	PV	30 µM	287	298	IKSYsFPKPGHK	s5: Phospho (+79.97)	99.70%	2.2	17.6	0	3	490.25	1,467.73	-0.00017
493	PE	PV	30 µM	287	298	IKsYsFPKPGHK	s3: Phospho (+79.97), s5: Phospho (+79.97)	99.70%	3.38	14.8	0	3	516.91	1,547.69	0.00014
494	PE	PV	30 µM	287	298	IKSysFPKPGHK	y4: Phospho (+79.97), s5: Phospho (+79.97)	99.70%	3.26	26.5	0	3	516.91	1,547.70	0.00124
495	PE	PV	30 µM	287	298	IKSysFPKPGHK	y4: Phospho (+79.97), s5: Phospho (+79.97)	99.70%	3.81	27.5	1.32	3	516.91	1,547.69	-0.00041
496	PE	PV	30 µM	287	298	IKSysFPKPGHK	y4: Phospho (+79.97), s5: Phospho (+79.97)	96.60%	0.92	25.7	0	2	774.85	1,547.69	-0.00058
497	PE	PV	30 µM	287	298	IKsYsFPKPGHK	s3: Phospho (+79.97), s5: Phospho (+79.97)	97.70%	1.29	16.8	0	3	516.91	1,547.70	0.00673
498	PE	PV	30 µM	287	298	IKSYsFPKPGHK	s5: Phospho (+79.97)	99.70%	0.9	26.6	3.19	2	734.87	1,467.73	-0.00104
499	PE	PV	30 µM	287	298	IKSysFPKPGHK	y4: Phospho (+79.97), s5: Phospho (+79.97)	99.70%	1.89	27.2	0.55	2	774.86	1,547.70	0.00186
500	PE	PV	30 µM	287	298	IKSySFPKPGHK	y4: Phospho (+79.97)	99.70%	3.08	45.3	6.36	2	734.87	1,467.73	-0.00104
501	PE	PV	30 µM	287	298	IKSySFPKPGHK	y4: Phospho (+79.97)	99.70%	2.75	19.6	3.42	3	490.25	1,467.73	-0.00063
502	PE	PV	30 µM	287	298	IKsYsFPKPGHK	s3: Phospho (+79.97), s5: Phospho (+79.97)	99.70%	2.6	11.7	2	3	516.91	1,547.70	0.00563
503	PE	PV	30 µM	287	298	IKSysFPKPGHK	y4: Phospho (+79.97), s5: Phospho (+79.97)	99.70%	3.58	38.9	2.77	2	774.86	1,547.70	0.00296
504	PE	PV	30 µM	287	298	IKSySFPKPGHK	y4: Phospho (+79.97)	99.70%	2.68	34.8	3.32	3	490.25	1,467.73	0.00111

505	PE	PV	30 µM	287	298	IKSysFPKPGHK	y4: Phospho (+79.97), s5: Phospho (+79.97)	99.70%	3.09	24.2	1.52	3	516.91	1,547.71	0.01223
506	PE	PV	30 µM	287	298	IKsYsFPKPGHK	s3: Phospho (+79.97), s5: Phospho (+79.97)	82.00%	2.78	10.2	0	3	516.91	1,547.72	0.02340
507	PE	PV	30 µM	287	298	IKsYsFPKPGHK	s3: Phospho (+79.97), s5: Phospho (+79.97)	97.30%	2.21	14.4	0	3	516.91	1,547.72	0.02523
508	PE	PV	30 µM	287	298	IKsYsFPKPGHK	s3: Phospho (+79.97), s5: Phospho (+79.97)	99.10%	2.64	19.9	0	3	516.91	1,547.72	0.02431
509	PE	PV	30 µM	287	298	IKSYsFPKPGHK	s5: Phospho (+79.97)	99.30%	1.9	19.7	0	3	490.26	1,467.75	0.02290
510	PE	PV	30 µM	287	298	IKsYsFPKPGHK	s3: Phospho (+79.97), s5: Phospho (+79.97)	99.50%	1.84	11.1	0	2	774.87	1,547.72	0.02188
511	PE	PV	30 µM	287	298	IKSysFPKPGHK	y4: Phospho (+79.97), s5: Phospho (+79.97)	99.60%	1.98	37.4	0	2	774.87	1,547.72	0.02249
512	PE	PV	30 µM	287	298	IKSYsFPKPGHK	s5: Phospho (+79.97)	99.70%	2.23	34.8	0	3	490.26	1,467.75	0.02253
513	PE	PV	30 µM	287	298	IKSysFPKPGHK	y4: Phospho (+79.97), s5: Phospho (+79.97)	99.70%	3.02	18.2	0	3	516.91	1,547.72	0.02468
514	PE	PV	30 µM	287	298	IKSysFPKPGHK	y4: Phospho (+79.97), s5: Phospho (+79.97)	99.70%	2.41	34	0	2	774.86	1,547.72	0.02127
515	PE	PV	30 µM	287	298	IKSySFPKPGHK	y4: Phospho (+79.97)	86.50%	1.39	0	1.36	4	367.94	1,467.73	0.00491
516	PE	PV	30 µM	287	298	IKsYsFPKPGHK	s3: Phospho (+79.97), s5: Phospho (+79.97)	97.80%	1.7	0	0	3	516.91	1,547.70	0.00106
517	PE	PV	30 µM	287	298	IKsYsFPKPGHK	s3: Phospho (+79.97), s5: Phospho (+79.97)	98.20%	1.07	17.2	0	2	774.85	1,547.69	-0.00046
518	PE	PV	30 µM	287	298	IKsYSFPKPGHK	s3: Phospho (+79.97)	99.00%	1.48	0	0	3	490.25	1,467.73	0.00111
519	PE	PV	30 µM	287	298	IKsYsFPKPGHK	s3: Phospho (+79.97), s5: Phospho (+79.97)	99.10%	1.8	10.2	0	3	516.91	1,547.70	0.00197
520	PE	PV	30 µM	287	298	IKSySFPKPGHK	y4: Phospho (+79.97)	99.70%	1.84	30.9	2.15	2	734.87	1,467.73	0.00299
521	PE	PV	30 µM	287	298	IKsYsFPKPGHK	s3: Phospho (+79.97), s5: Phospho (+79.97)	99.70%	1.79	10.9	1.66	3	516.91	1,547.70	0.00234
522	PE	PV	30 µM	287	298	IKsYsFPKPGHK	s3: Phospho (+79.97), s5: Phospho (+79.97)	99.70%	1.96	0	1.96	3	516.91	1,547.70	0.00454
523	PE	PV	30 µM	287	298	IKsysFPKPGHK	s3: Phospho (+79.97), y4: Phospho (+79.97), s5: Phospho (+79.97)	99.70%	1.95	10.2	1.13	3	543.56	1,627.66	-0.00147
524	PE	PV	30 µM	287	298	IKsYsFPKPGHK	s3: Phospho (+79.97), s5: Phospho (+79.97)	99.70%	1.91	14	1.6	3	516.91	1,547.70	0.00216
525	PE	PV	30 µM	287	298	IKsysFPKPGHK	s3: Phospho (+79.97), y4: Phospho (+79.97), s5: Phospho (+79.97)	99.70%	1.82	13.3	0	3	543.56	1,627.66	0.00110
526	PE	PV	30 µM	287	298	IKSysFPKPGHK	y4: Phospho (+79.97), s5: Phospho (+79.97)	99.70%	1.89	15.4	2.24	3	516.91	1,547.70	0.00161
527	PE	PV	30 µM	287	298	IKsYsFPKPGHK	s3: Phospho (+79.97), s5: Phospho (+79.97)	99.70%	1.93	10.5	0.89	3	516.91	1,547.70	0.00179
528	PE	PV	30 µM	287	298	IKsYsFPKPGHK	s3: Phospho (+79.97), s5: Phospho (+79.97)	99.70%	2.14	0	0	3	516.91	1,547.70	0.00161

529	PE	PV	30 μM	287	298	IKSysFPKPGHK	y4: Phospho (+79.97), s5: Phospho (+79.97)	99.70%	2.4	26.8	2.31	3	516.91	1,547.70	0.00124
530	PE	PV	30 μM	287	298	IKsYsFPKPGHK	s3: Phospho (+79.97), s5: Phospho (+79.97)	99.70%	1.95	15.2	1.55	3	516.91	1,547.70	0.00271
531	PE	PV	30 μM	287	298	IKSysFPKPGHK	y4: Phospho (+79.97), s5: Phospho (+79.97)	99.70%	2.35	17	1.92	3	516.91	1,547.70	0.00454
532	PE	PV	30 μM	287	298	IKsYsFPKPGHK	s3: Phospho (+79.97), s5: Phospho (+79.97)	99.70%	2.2	10.6	1.64	3	516.91	1,547.70	0.00344
533	PE	PV	30 μM	287	298	IKsYsFPKPGHK	s3: Phospho (+79.97), s5: Phospho (+79.97)	99.70%	2.13	10	2.72	3	516.91	1,547.70	0.00271
534	PE	PV	30 μM	287	298	IKsYsFPKPGHK	s3: Phospho (+79.97), s5: Phospho (+79.97)	99.70%	2.63	18.6	1.48	3	516.91	1,547.70	0.00289
535	PE	PV	30 μM	287	298	IKSysFPKPGHK	y4: Phospho (+79.97), s5: Phospho (+79.97)	99.70%	2.31	19.4	1.43	3	516.91	1,547.70	0.00216
536	PE	PV	30 μM	287	298	IKSySFPKPGHK	y4: Phospho (+79.97)	99.70%	2.48	39.8	2.82	3	490.25	1,467.73	0.00129
537	PE	PV	30 μM	287	298	IKSySFPKPGHK	y4: Phospho (+79.97)	99.70%	2.4	32.2	3.32	3	490.25	1,467.73	0.00367
538	PE	PV	30 μM	287	298	IKSysFPKPGHK	y4: Phospho (+79.97), s5: Phospho (+79.97)	99.70%	2.7	15.4	1.59	3	516.91	1,547.70	0.00271
539	PE	PV	30 μM	287	298	IKSySFPKPGHK	y4: Phospho (+79.97)	99.70%	2.65	24.9	2.09	3	490.25	1,467.73	0.00377
540	PE	PV	100 μM	287	298	IKSysFPKPGHK	y4: Phospho (+79.97), s5: Phospho (+79.97)	98.20%	1.35	10.5	0.5	3	516.91	1,547.70	0.00179
541	PE	PV	100 μM	287	298	IKSysFPKPGHK	y4: Phospho (+79.97), s5: Phospho (+79.97)	98.50%	1.45	12.7	0.8	3	516.91	1,547.70	0.00124
542	PE	PV	100 μM	287	298	IKsYsFPKPGHK	s3: Phospho (+79.97), s5: Phospho (+79.97)	93.30%	2.53	0	0	3	516.91	1,547.70	0.00252
543	PE	PV	100 μM	287	298	IKSySFPKPGHK	y4: Phospho (+79.97)	99.20%	1.93	15.5	0	3	490.25	1,467.73	0.00468
544	PE	PV	100 μM	287	298	IKsYsFPKPGHK	s3: Phospho (+79.97), s5: Phospho (+79.97)	99.70%	2.92	0	0	3	516.91	1,547.70	0.00417
545	PE	PV	100 μM	287	298	IKsYsFPKPGHK	s3: Phospho (+79.97), s5: Phospho (+79.97)	99.70%	2.66	15.8	0	3	516.91	1,547.70	0.00271
546	PE	PV	100 μM	287	298	IKSysFPKPGHK	y4: Phospho (+79.97), s5: Phospho (+79.97)	99.70%	2.86	13.6	0	3	516.91	1,547.70	0.00362
547	PE	PV	100 μM	287	298	IKSysFPKPGHK	y4: Phospho (+79.97), s5: Phospho (+79.97)	99.70%	3.07	14.4	0	3	516.91	1,547.70	0.00490
548	PE	PV	100 μM	287	298	IKSysFPKPGHK	y4: Phospho (+79.97), s5: Phospho (+79.97)	99.70%	2.92	14.7	0	3	516.91	1,547.70	0.00563
549	PE	PV	100 μM	287	298	IKSysFPKPGHK	y4: Phospho (+79.97), s5: Phospho (+79.97)	98.60%	3.02	0	0	3	516.91	1,547.70	0.00307
550	PE	PV	100 μM	287	298	IKSysFPKPGHK	y4: Phospho (+79.97), s5: Phospho (+79.97)	99.20%	1.93	13.1	0	3	516.91	1,547.69	-0.00059
551	PE	PV	100 μM	287	298	IKsYSFPKPGHK	s3: Phospho (+79.97)	99.70%	2.23	13.1	0	3	490.25	1,467.73	0.00148
552	PE	PV	100 μM	287	298	IKsYsFPKPGHK	s3: Phospho (+79.97), s5: Phospho (+79.97)	99.70%	2.83	11.6	0	3	516.91	1,547.69	0.00051
553	PE	PV	100 μM	287	298	IKSysFPKPGHK	y4: Phospho (+79.97), s5: Phospho (+79.97)	99.70%	3.39	10.9	0	3	516.91	1,547.69	0.00014

554	PE	PV	100 µM	287	298	IKSysFPKPGHK	y4: Phospho (+79.97), s5: Phospho (+79.97)	97.90%	1.2	12.5	1.03	2	774.86	1,547.70	0.01090
555	PE	PV	100 µM	287	298	IKsysFPKPGHK	s3: Phospho (+79.97), y4: Phospho (+79.97), s5: Phospho (+79.97)	98.40%	0.93	12.4	0	2	814.84	1,627.66	0.00245
556	PE	PV	100 µM	287	298	IKsYsFPKPGHK	s3: Phospho (+79.97), s5: Phospho (+79.97)	98.70%	1.72	16.3	0	3	516.91	1,547.70	0.00600
557	PE	PV	100 µM	287	298	IKsYSFPKPGHK	s3: Phospho (+79.97)	99.70%	2.06	0	0	3	490.25	1,467.73	-0.00072
558	PE	PV	100 µM	287	298	IKSySFPKPGHK	y4: Phospho (+79.97)	99.70%	1.82	24.8	2.37	3	490.25	1,467.73	0.00230
559	PE	PV	100 µM	287	298	IKSySFPKPGHK	y4: Phospho (+79.97)	99.70%	2.91	30.8	3.16	3	490.25	1,467.73	0.00111
560	PE	PV	100 µM	287	298	IKSySFPKPGHK	y4: Phospho (+79.97)	99.70%	2.54	53.2	5.57	2	734.87	1,467.73	0.00152
561	PE	PV	100 µM	287	298	IKsysFPKPGHK	s3: Phospho (+79.97), y4: Phospho (+79.97), s5: Phospho (+79.97)	99.70%	2.29	14.9	2.41	3	543.56	1,627.67	0.00494
562	PE	PV	100 µM	287	298	IKSysFPKPGHK	y4: Phospho (+79.97), s5: Phospho (+79.97)	99.70%	2.53	49	1.54	2	774.86	1,547.70	0.00272
563	PE	PV	100 µM	287	298	IKSySFPKPGHK	y4: Phospho (+79.97)	99.70%	2.62	49.2	4.32	2	734.87	1,467.73	-0.00031
564	PE	PV	100 µM	287	298	IKsYsFPKPGHK	s3: Phospho (+79.97), s5: Phospho (+79.97)	99.70%	2.5	24	2.49	3	516.91	1,547.70	0.00637
565	PE	PV	100 µM	287	298	IKsYsFPKPGHK	s3: Phospho (+79.97), s5: Phospho (+79.97)	99.70%	2.68	22.5	2.28	3	516.91	1,547.70	0.00728
566	PE	PV	100 µM	287	298	IKSysFPKPGHK	y4: Phospho (+79.97), s5: Phospho (+79.97)	99.70%	3.2	23.9	2.44	3	516.91	1,547.70	0.00655
567	PE	PV	100 µM	287	298	IKSysFPKPGHK	y4: Phospho (+79.97), s5: Phospho (+79.97)	98.80%	1.99	12.8	0	2	774.87	1,547.72	0.02555
568	PE	PV	100 µM	287	298	IKsysFPKPGHK	s3: Phospho (+79.97), y4: Phospho (+79.97), s5: Phospho (+79.97)	99.10%	2.44	14.6	0	3	543.57	1,627.69	0.02581
569	PE	PV	100 µM	287	298	IKSysFPKPGHK	y4: Phospho (+79.97), s5: Phospho (+79.97)	99.20%	2.76	14.7	0	3	516.91	1,547.72	0.02541
570	PE	PV	100 µM	287	298	IKSysFPKPGHK	y4: Phospho (+79.97), s5: Phospho (+79.97)	99.60%	2.17	25.8	0	2	774.87	1,547.72	0.02225
571	PE	PV	100 µM	287	298	IKsysFPKPGHK	s3: Phospho (+79.97), y4: Phospho (+79.97), s5: Phospho (+79.97)	99.70%	2.38	17.2	0	3	543.57	1,627.69	0.02728
572	PE	PV	100 µM	287	298	IKsYsFPKPGHK	s3: Phospho (+79.97), s5: Phospho (+79.97)	99.70%	3.42	14.6	0	3	516.91	1,547.72	0.02669
573	PE	PV	100 µM	287	298	IKSysFPKPGHK	y4: Phospho (+79.97), s5: Phospho (+79.97)	85.00%	1.97	0	0	3	516.91	1,547.70	0.00399
574	PE	PV	100 µM	287	298	IKsySFPKPGHK	s3: Phospho (+79.97), y4: Phospho (+79.97)	89.10%	0	0	1.39	3	517.24	1,548.70	1.00600
575	PE	PV	100 µM	287	298	IKsYsFPKPGHK	s3: Phospho (+79.97), s5: Phospho (+79.97)	93.00%	1.88	0	0	3	516.91	1,547.70	0.00271
576	PE	PV	100 µM	287	298	IKsYSFPKPGHK	s3: Phospho (+79.97)	93.60%	1.17	0	0.51	3	490.25	1,467.73	0.00038

577	PE	PV	100 µM	287	298	IKsYsFPKPGHK	s3: Phospho (+79.97), s5: Phospho (+79.97)	95.10%	1.96	0	0	3	516.91	1,547.70	0.00289
578	PE	PV	100 µM	287	298	IKsySFPKPGHK	s3: Phospho (+79.97), y4: Phospho (+79.97)	97.20%	0	0	1.96	3	517.24	1,548.70	1.00500
579	PE	PV	100 µM	287	298	IKSySFPKPGHK	y4: Phospho (+79.97)	99.70%	2	0	0.68	4	367.94	1,467.73	0.00125
580	PE	PV	100 µM	287	298	IKsysFPKPGHK	s3: Phospho (+79.97), y4: Phospho (+79.97), s5: Phospho (+79.97)	99.70%	1.51	14.5	1.21	2	814.84	1,627.66	0.00050
581	PE	PV	100 µM	287	298	IKsysFPKPGHK	s3: Phospho (+79.97), y4: Phospho (+79.97), s5: Phospho (+79.97)	99.70%	1.55	11.5	0.77	3	543.56	1,627.66	0.00091
582	PE	PV	100 µM	287	298	IKsysFPKPGHK	s3: Phospho (+79.97), y4: Phospho (+79.97), s5: Phospho (+79.97)	99.70%	1.01	21	0	2	814.84	1,627.66	-0.00231
583	PE	PV	100 µM	287	298	IKSySFPKPGHK	y4: Phospho (+79.97)	99.70%	1.8	0	0.82	4	367.94	1,467.73	0.00259
584	PE	PV	100 µM	287	298	IKsysFPKPGHK	s3: Phospho (+79.97), y4: Phospho (+79.97), s5: Phospho (+79.97)	99.70%	1.97	10	1.35	3	543.56	1,627.66	0.00018
585	PE	PV	100 µM	287	298	IKsysFPKPGHK	s3: Phospho (+79.97), y4: Phospho (+79.97), s5: Phospho (+79.97)	99.70%	1.66	20.2	1.07	3	543.56	1,627.66	-0.00147
586	PE	PV	100 µM	287	298	IKSysFPKPGHK	y4: Phospho (+79.97), s5: Phospho (+79.97)	99.70%	2.01	60.5	1.72	2	774.85	1,547.69	-0.00094
587	PE	PV	100 µM	287	298	IKsYsFPKPGHK	s3: Phospho (+79.97), s5: Phospho (+79.97)	99.70%	2.27	0	0.42	3	516.91	1,547.70	0.00216
588	PE	PV	100 µM	287	298	IKsYSFPKPGHK	s3: Phospho (+79.97)	99.70%	2.06	26.2	0.89	3	490.25	1,467.73	0.00285
589	PE	PV	100 µM	287	298	IKSYsFPKPGHK	s5: Phospho (+79.97)	99.70%	1.78	40.5	4.21	2	734.87	1,467.73	0.00176
590	PE	PV	100 µM	287	298	IKsYSFPKPGHK	s3: Phospho (+79.97)	99.70%	2.23	24.1	1.74	3	490.25	1,467.73	0.00001
591	PE	PV	100 µM	287	298	IKsysFPKPGHK	s3: Phospho (+79.97), y4: Phospho (+79.97), s5: Phospho (+79.97)	99.70%	2.18	18.7	1.72	3	543.56	1,627.67	0.00512
592	PE	PV	100 µM	287	298	IKsysFPKPGHK	s3: Phospho (+79.97), y4: Phospho (+79.97), s5: Phospho (+79.97)	99.70%	2.56	20.3	0	3	543.56	1,627.66	0.00146
593	PE	PV	100 µM	287	298	IKSysFPKPGHK	y4: Phospho (+79.97), s5: Phospho (+79.97)	99.70%	2.57	18.8	0.68	3	516.91	1,547.70	0.00472
594	PE	PV	100 µM	287	298	IKSysFPKPGHK	y4: Phospho (+79.97), s5: Phospho (+79.97)	99.70%	2.97	18.8	1.89	3	516.91	1,547.70	0.00289
595	PE	PV	100 µM	287	298	IKSySFPKPGHK	y4: Phospho (+79.97)	99.70%	2.49	33	2.92	3	490.25	1,467.73	0.00120
596	PE	PV	100 µM	287	298	IKsYsFPKPGHK	s3: Phospho (+79.97), s5: Phospho (+79.97)	99.70%	2.56	21.1	1.05	3	516.91	1,547.70	0.00344
597	PE	PV	100 µM	287	298	IKsYsFPKPGHK	s3: Phospho (+79.97), s5: Phospho (+79.97)	99.70%	2.87	0	2.15	3	516.91	1,547.70	0.00271
598	PE	Ig	2 min	289	298	SYsFPKPGHK	s3: Phospho (+79.97)	83.40%	1.43	0	0	3	409.86	1,226.55	0.00412
599	PE	Hg	1 µM	289	298	SYsFPKPGHK	s3: Phospho (+79.97)	88.10%	1.13	0	0.52	3	409.86	1,226.55	0.00495

600	PE	Hg	5 µM	289	298	SYsFPKPGHK	s3: Phospho (+79.97)	99.70%	1.52	0	0.62	3	409.86	1,226.55	0.00330
601	PE	PV	3 µM	289	298	SYsFPKPGHK	s3: Phospho (+79.97)	97.50%	2.04	12.1	0	3	409.86	1,226.57	0.01822
602	PE	PV	3 µM	289	298	SYsFPKPGHK	s3: Phospho (+79.97)	99.30%	2.2	12.8	0	3	409.86	1,226.57	0.01841
603	PE	PV	10 µM	289	298	sYSFPKPGHK	s1: Phospho (+79.97)	98.50%	1.09	11	0	3	409.86	1,226.55	0.00339
604	PE	PV	10 µM	289	298	sYSFPKPGHK	s1: Phospho (+79.97)	99.70%	2.41	14.7	1.23	3	409.86	1,226.55	0.00339
605	PE	PV	10 µM	289	298	sYSFPKPGHK	s1: Phospho (+79.97)	99.70%	2.03	40.7	9.62	2	614.28	1,226.55	0.00167
606	PE	PV	10 µM	289	298	sYsFPKPGHK	s1: Phospho (+79.97), s3: Phospho (+79.97)	99.20%	0.74	11.1	1.46	2	654.26	1,306.51	-0.00031
607	PE	PV	10 µM	289	298	SySFPKPGHK	y2: Phospho (+79.97)	99.70%	1.23	13.6	1.77	3	409.86	1,226.55	-0.00055
608	PE	PV	10 µM	289	298	SySFPKPGHK	y2: Phospho (+79.97)	99.70%	1.88	15.8	2.43	3	409.86	1,226.55	0.00156
609	PE	PV	30 µM	289	298	SySFPKPGHK	y2: Phospho (+79.97)	99.70%	1.48	11.4	1.15	3	409.86	1,226.55	0.00477
610	PE	PV	30 µM	289	298	sYSFPKPGHK	s1: Phospho (+79.97)	99.70%	1.11	11.1	1.02	3	409.86	1,226.55	0.00422
611	PE	PV	30 µM	289	298	SySFPKPGHK	y2: Phospho (+79.97)	99.70%	2.16	17.7	1.49	3	409.86	1,226.55	0.00330
612	PE	PV	30 µM	289	298	SySFPKPGHK	y2: Phospho (+79.97)	99.70%	1.71	36.5	4.89	2	614.28	1,226.55	0.00167
613	PE	PV	30 µM	289	298	sYSFPKPGHK	s1: Phospho (+79.97)	99.70%	1.53	39.1	3.8	2	614.28	1,226.55	0.00106
614	PE	PV	30 µM	289	298	sYsFPKPGHK	s1: Phospho (+79.97), s3: Phospho (+79.97)	99.60%	0.7	0	1.23	2	654.26	1,306.51	-0.00031
615	PE	PV	30 µM	289	298	sYSFPKPGHK	s1: Phospho (+79.97)	99.70%	1.17	14.2	1.57	2	614.28	1,226.55	-0.00053
616	PE	PV	30 µM	289	298	SySFPKPGHK	y2: Phospho (+79.97)	99.70%	1.14	11.1	1.68	2	614.28	1,226.55	-0.00224
617	PE	PV	30 µM	289	298	SySFPKPGHK	y2: Phospho (+79.97)	99.70%	1.8	21.5	2.6	3	409.86	1,226.55	0.00229
618	PE	PV	30 µM	289	298	SysFPKPGHK	y2: Phospho (+79.97), s3: Phospho (+79.97)	99.70%	1.63	24.7	4.51	2	654.26	1,306.51	-0.00129
619	PE	PV	30 µM	289	298	SySFPKPGHK	y2: Phospho (+79.97)	99.70%	2.43	15.2	2.39	3	409.86	1,226.55	0.00119
620	PE	PV	30 µM	289	298	SySFPKPGHK	y2: Phospho (+79.97)	99.70%	2.32	12.8	2.4	3	409.86	1,226.55	0.00000
621	PE	PV	30 µM	289	298	SySFPKPGHK	y2: Phospho (+79.97)	99.70%	2.05	15.1	2.51	3	409.86	1,226.55	0.00129
622	PE	PV	100 µM	289	298	SySFPKPGHK	y2: Phospho (+79.97)	99.70%	1.78	11.6	0.89	3	409.86	1,226.55	0.00577
623	PE	PV	100 µM	289	298	sYSFPKPGHK	s1: Phospho (+79.97)	99.70%	1.47	17.2	2.24	2	614.28	1,226.55	-0.00139
624	PE	PV	100 µM	289	298	SySFPKPGHK	y2: Phospho (+79.97)	99.70%	2.57	34.9	8.41	2	614.28	1,226.55	0.00215
625	PE	PV	100 µM	289	298	SysFPKPGHK	y2: Phospho (+79.97), s3: Phospho (+79.97)	99.60%	1.56	13.9	0.85	3	436.51	1,306.52	0.00050
626	PE	PV	100 µM	289	298	SySFPKPGHK	y2: Phospho (+79.97)	99.70%	1.67	0	1.27	3	409.86	1,226.55	0.00174
627	PE	PV	100 µM	289	298	SySFPKPGHK	y2: Phospho (+79.97)	99.70%	2.18	19.2	3.06	3	409.86	1,226.55	-0.00045
628	PE	PV	100 µM	289	298	SySFPKPGHK	y2: Phospho (+79.97)	99.70%	2.1	13.9	3.09	3	409.86	1,226.55	0.00202
629	IP	NT	0	299	328	KPAPPQGSRPESTVSFNPyEPTGGPWGPDR	y19: Phospho (+79.97)	98.20%	2.7	24.8	3.66	4	822.89	3,287.52	0.01562
630	IP	NT	0	299	328	KPAPPQGSRPESTVSFNPyEPTGGPWGPDR	y19: Phospho (+79.97)	99.10%	2.12	37.7	8.41	3	1096.84	3,287.51	0.00569
631	IP	NT	0	299	328	KPAPPQGSRPESTVSFNPyEPTGGPWGPDR	y19: Phospho (+79.97)	99.70%	5.11	35.9	11.4	4	822.88	3,287.50	-0.00782
632	IP	NT	0	299	328	KPAPPQGSRPESTVSFNPyEPTGGPWGPDR	y19: Phospho (+79.97)	99.10%	5.29	48.7	2.96	3	1096.84	3,287.50	0.00019
633	IP	NT	0	299	328	KPAPPQGSRPESTVSFNPyEPTGGPWGPDR	y19: Phospho (+79.97)	99.40%	5.27	52	2.96	3	1096.84	3,287.51	0.00276
634	IP	NT	0	299	328	KPAPPQGSRPESTVSFNPyEPTGGPWGPDR	y19: Phospho (+79.97)	99.70%	6.24	50.6	3.19	3	1096.84	3,287.50	0.00056
635	IP	Hg	50 µM	299	328	KPAPPQGSRPESTVSFnPyEPTGGPWGPDR	n17: Deamidated (+0.98), y19: Phospho (+79.97)	99.10%	3.64	17.8	10.6	3	1097.18	3,288.51	0.02399
636	IP	Hg	50 µM	299	328	KPAPPQGSRPESTVSFNPyEPTGGPWGPDR	y19: Phospho (+79.97)	99.60%	3.07	42.6	10	4	822.89	3,287.51	0.00732
637	IP	Hg	50 µM	299	328	KPAPPQGSRPESTVSFNPyEPTGGPWGPDR	y19: Phospho (+79.97)	99.10%	4.97	35.2	2.57	3	1096.84	3,287.50	-0.00677
638	IP	Hg	50 µM	299	328	KPAPPQGSRPESTVSFNPyEPTGGPWGPDR	y19: Phospho (+79.97)	99.50%	5.29	30.5	3.74	3	1096.84	3,287.50	-0.00054
639	IP	Hg	50 µM	299	328	KPAPPQGSRPESTVSFNPyEPTGGPWGPDR	y19: Phospho (+79.97)	99.70%	5.33	35.6	5.66	3	1096.84	3,287.50	-0.00017

640	IP	PV	0	299	328	KPAPPQGSRPESTVSFNPYEPtGGPWGPDR	t22: Phospho (+79.97)	99.70%	5.13	25.4	13.6	3	1096.84	3,287.51	0.00239
641	IP	PV	0	299	328	KPAPPQGSRPESTVSFNPyEPTGGPWGPDR	y19: Phospho (+79.97)	99.70%	4.19	36.6	13.3	4	822.89	3,287.51	0.00732
642	IP	PV	0	299	328	KPAPPQGSRPESTVSFNPyEPTGGPWGPDR	y19: Phospho (+79.97)	98.60%	3.69	24.4	1.32	4	822.88	3,287.50	-0.00196
643	IP	PV	0	299	328	KPAPPQGSRPESTVSFNPyEPTGGPWGPDR	y19: Phospho (+79.97)	98.90%	3.66	25.9	1.6	4	822.88	3,287.51	0.00268
644	IP	PV	0	299	328	KPAPPQGSRPESTVSFNPyEPTGGPWGPDR	y19: Phospho (+79.97)	99.60%	5.86	37.4	1.66	3	1096.84	3,287.51	0.00349
645	IP	PV	0	299	328	KPAPPQGSRPESTVSFNPyEPTGGPWGPDR	y19: Phospho (+79.97)	99.70%	3.59	28.6	2.11	4	822.88	3,287.51	0.00268
646	IP	PV	0	299	328	KPAPPQGSRPESTVSFNPyEPTGGPWGPDR	y19: Phospho (+79.97)	99.70%	5.63	32.5	2.82	3	1096.84	3,287.51	0.00495
647	IP	PV	0	299	328	KPAPPQGSRPESTVSFNPyEPTGGPWGPDR	y19: Phospho (+79.97)	99.70%	4.19	30.1	1.92	4	822.88	3,287.51	0.00341
648	IP	PV	0	299	328	KPAPPQGSRPESTVSFNPyEPTGGPWGPDR	y19: Phospho (+79.97)	99.70%	5.92	40.7	2.44	3	1096.84	3,287.51	0.00459
649	IP	PV	0	299	328	KPAPPQGSRPESTVSFNPyEPTGGPWGPDR	y19: Phospho (+79.97)	99.70%	5.8	48.8	4.64	3	1096.84	3,287.51	0.00569
650	IP	PV	0	299	328	KPAPPQGSRPESTVSFNPyEPTGGPWGPDR	y19: Phospho (+79.97)	99.70%	3.95	33.5	2.8	4	822.88	3,287.51	0.00586
651	IP	PV	0	299	328	KPAPPQGSRPESTVSFnPyEPTGGPWGPDR	n17: Deamidated (+0.98), y19: Phospho (+79.97)	99.70%	5.97	60.1	4.8	3	1097.18	3,288.51	0.02655
652	PE	Ig	NT	299	328	KPAPPQGSRPESTVSFNPyEPTGGPWGPDR	y19: Phospho (+79.97)	99.70%	3.89	0	5.55	4	822.89	3,287.51	0.00683
653	PE	Ig	NT	299	328	KPAPPQGSRPESTVSFNPyEPTGGPWGPDR	y19: Phospho (+79.97)	99.70%	4.72	20.7	8.89	4	822.89	3,287.53	0.02563
654	PE	Ig	1 min	299	328	KPAPPQGSRPESTVSFNPyEPTGGPWGPDR	y19: Phospho (+79.97)	99.70%	5.12	11.1	5.08	4	822.89	3,287.51	0.00732
655	PE	Ig	1 min	299	328	KPAPPQGSRPESTVSFNPyEPTGGPWGPDR	y19: Phospho (+79.97)	99.70%	4.87	22.3	8.59	4	822.89	3,287.53	0.02221
656	PE	Ig	1 min	299	328	KPAPPQGSRPESTVSFNPyEPTGGPWGPDR	y19: Phospho (+79.97)	99.70%	4.5	26.2	8.34	4	822.89	3,287.52	0.01611
657	PE	Ig	1 min	299	328	KPAPPQGSRPESTVSFNPyEPTGGPWGPDR	y19: Phospho (+79.97)	99.70%	5.22	21.7	11	4	822.89	3,287.53	0.02465
658	PE	Ig	1 min	299	328	KPAPPQGSRPESTVSFNPyEPTGGPWGPDR	y19: Phospho (+79.97)	99.70%	4.99	18.1	11.1	4	822.89	3,287.52	0.01831
659	PE	Ig	1 min	299	328	KPAPPQGSRPESTVSFNPyEPTGGPWGPDR	y19: Phospho (+79.97)	99.70%	5.58	26.1	8.6	4	822.89	3,287.53	0.02490
660	PE	Ig	1 min	299	328	KPAPPQGSRPESTVSFNPyEPTGGPWGPDR	y19: Phospho (+79.97)	99.70%	4.78	24	10.7	4	822.88	3,287.51	0.00122
661	PE	Ig	1 min	299	328	KPAPPQGSRPESTVSFNPyEPTGGPWGPDR	y19: Phospho (+79.97)	99.70%	3.45	23.9	6.42	4	822.90	3,287.55	0.04834
662	PE	Ig	1 min	299	328	KPAPPQGSRPESTVSFNPyEPTGGPWGPDR	y19: Phospho (+79.97)	99.70%	2.52	17.4	6.49	3	1096.86	3,287.55	0.04727
663	PE	Ig	1 min	299	328	KPAPPQGSRPESTVSFNPyEPTGGPWGPDR	y19: Phospho (+79.97)	99.70%	2.78	23.2	7.12	3	1096.86	3,287.55	0.04487
664	PE	Ig	1 min	299	328	KPAPPQGSRPESTVSFNPyEPTGGPWGPDR	y19: Phospho (+79.97)	99.70%	3.59	33.1	9.6	4	822.89	3,287.53	0.02587
665	PE	Ig	1 min	299	328	KPAPPQGSRPESTVSFnPyEPTGGPWGPDR	n17: Deamidated (+0.98), y19: Phospho (+79.97)	99.70%	3.65	20	11.9	4	823.15	3,288.55	0.06359
666	PE	Ig	1 min	299	328	KPAPPQGSRPESTVSFNPyEPTGGPWGPDR	y19: Phospho (+79.97)	99.70%	3.63	19.6	11.5	3	1096.86	3,287.55	0.04633
667	PE	Ig	1 min	299	328	KPAPPQGSRPESTVSFNPyEPTGGPWGPDR	y19: Phospho (+79.97)	99.70%	3.73	37.9	10.4	4	822.89	3,287.54	0.03881
668	PE	Ig	1 min	299	328	KPAPPQGSRPESTVSFNPyEPTGGPWGPDR	y19: Phospho (+79.97)	99.70%	3.67	25.8	11.9	4	822.89	3,287.54	0.03320
669	PE	Ig	2 min	299	328	KPAPPQGSRPESTVSFNPyEPTGGPWGPDR	y19: Phospho (+79.97)	99.70%	3.33	26.3	10.5	4	822.89	3,287.52	0.01489
670	PE	Ig	2 min	299	328	KPAPPQGSRPESTVSFNPyEPTGGPWGPDR	y19: Phospho (+79.97)	99.60%	2.59	11.5	3.74	3	1096.85	3,287.52	0.01814
671	PE	Ig	2 min	299	328	KPAPPqGSRPESTVSFNPyEPTGGPWGPDR	q6: Deamidated (+0.98), y19: Phospho (+79.97)	99.70%	2.96	15.1	8.24	4	823.14	3,288.51	0.02648
672	PE	Ig	2 min	299	328	KPAPPQGSRPESTVSFNPyEPTGGPWGPDR	y19: Phospho (+79.97)	99.70%	4.86	11.7	6.55	4	822.89	3,287.51	0.00854
673	PE	Ig	2 min	299	328	KPAPPQGSRPESTVSFNPyEPTGGPWGPDR	y19: Phospho (+79.97)	99.70%	5.03	26.3	9.37	4	822.89	3,287.53	0.02734
674	PE	Ig	2 min	299	328	KPAPPQGSRPESTVSFNPyEPTGGPWGPDR	y19: Phospho (+79.97)	99.70%	0	0	12.6	4	823.39	3,289.52	2.01900
675	PE	Ig	2 min	299	328	KPAPPqGSRPESTVSFNPyEPTGGPWGPDR	q6: Deamidated (+0.98), y19: Phospho (+79.97)	99.70%	3.38	13.5	8.32	4	823.13	3,288.50	0.01403
676	PE	Ig	2 min	299	328	KPAPPQGSRPESTVSFNPyEPTGGPWGPDR	y19: Phospho (+79.97)	99.70%	5.05	30	9.77	4	822.89	3,287.53	0.02710
677	PE	Ig	2 min	299	328	KPAPPQGSRPESTVSFNPyEPTGGPWGPDR	y19: Phospho (+79.97)	99.70%	5.36	12.3	5.41	4	822.88	3,287.51	0.00537
678	PE	Ig	2 min	299	328	KPAPPQGSRPESTVSFNPyEPTGGPWGPDR	y19: Phospho (+79.97)	99.70%	3.85	33.6	11.6	3	1096.85	3,287.52	0.01484
679	PE	Ig	2 min	299	328	KPAPPQGSRPESTVSFNPyEPTGGPWGPDR	y19: Phospho (+79.97)	99.70%	5.16	23.3	12.6	4	822.89	3,287.52	0.01855
680	PE	Ig	2 min	299	328	KPAPPQGSRPESTVSFNPyEPTGGPWGPDR	y19: Phospho (+79.97)	99.70%	5.95	29.1	12	4	822.88	3,287.51	0.00244

681	PE	Ig	2 min	299	328	KPAPPQGSRPESTVSFNPyEPTGGPWGPDR	y19: Phospho (+79.97)	99.70%	2.99	37.9	0	4	822.88	3,287.51	0.00586
682	PE	Ig	2 min	299	328	KPAPPQGSRPESTVSFNPyEPTGGPWGPDR	y19: Phospho (+79.97)	99.70%	2.44	23.9	6	3	1096.86	3,287.55	0.04597
683	PE	Ig	2 min	299	328	KPAPPQGSRPESTVSFNPyEPTGGPWGPDR	y19: Phospho (+79.97)	99.70%	2.35	19.6	7.28	3	1096.86	3,287.55	0.04926
684	PE	Ig	2 min	299	328	KPAPPQGSRPESTVSFNPyEPTGGPWGPDR	y19: Phospho (+79.97)	99.70%	2.63	15.3	8	3	1096.86	3,287.56	0.05146
685	PE	Ig	2 min	299	328	KPAPPQGSRPESTVSFNPyEPTGGPWGPDR	y19: Phospho (+79.97)	99.70%	3.07	23.5	8.82	4	822.90	3,287.56	0.05273
686	PE	Ig	2 min	299	328	KPAPPQGSRPESTVSFNPyEPTGGPWGPDR	y19: Phospho (+79.97)	99.70%	3.43	27.5	8.66	4	822.90	3,287.55	0.05029
687	PE	Ig	2 min	299	328	KPAPPQGSRPESTVSFNPyEPTGGPWGPDR	y19: Phospho (+79.97)	99.70%	3.04	17	8.85	4	822.89	3,287.54	0.04077
688	PE	Ig	2 min	299	328	KPAPPQGSRPESTVSFNPyEPTGGPWGPDR	y19: Phospho (+79.97)	99.70%	2.47	27.2	11.7	3	1096.86	3,287.56	0.05256
689	PE	Ig	2 min	299	328	KPAPPQGSRPESTVSFNPyEPTGGPWGPDR	y19: Phospho (+79.97)	99.70%	2.7	28.8	11.3	4	822.90	3,287.56	0.05493
690	PE	Ig	2 min	299	328	KPAPPQGsRPESTVSFNPYEPTGGPWGPDR	s8: Phospho (+79.97)	99.70%	2.75	22.4	11.5	3	1096.86	3,287.57	0.06135
691	PE	Ig	2 min	299	328	KPAPPQGSRPESTVSFNPyEPTGGPWGPDR	y19: Phospho (+79.97)	99.70%	3.21	25.5	10	4	822.89	3,287.55	0.04467
692	PE	Ig	2 min	299	328	KPAPPQGSRPESTVSFNPyEPTGGPWGPDR	y19: Phospho (+79.97)	99.70%	3.25	21.8	10.7	4	822.89	3,287.54	0.04052
693	PE	Ig	2 min	299	328	KPAPPQGSRPESTVSFNPyEPTGGPWGPDR	y19: Phospho (+79.97)	99.70%	3.19	16.5	10.1	4	822.89	3,287.55	0.04541
694	PE	Ig	3 min	299	328	KPAPPQGSRPESTVSFNPyEPTGGPWGPDR	y19: Phospho (+79.97)	99.70%	1.98	37.2	5.74	4	822.88	3,287.51	0.00586
695	PE	Ig	3 min	299	328	KPAPPQGSRPESTVSFNPyEPTGGPWGPDR	y19: Phospho (+79.97)	93.80%	1.66	0	1.77	3	1096.85	3,287.52	0.01740
696	PE	Ig	3 min	299	328	KPAPPQGSRPESTVSFNPyEPTGGPWGPDR	y19: Phospho (+79.97)	99.70%	3.53	12.7	4.51	4	822.89	3,287.53	0.02929
697	PE	Ig	3 min	299	328	KPAPPQGSRPESTVSFNPyEPTGGPWGPDR	y19: Phospho (+79.97)	99.70%	2.19	20.2	7.04	3	1096.85	3,287.52	0.01924
698	PE	Ig	3 min	299	328	KPAPPQGSRPESTVSFNPyEPTGGPWGPDR	y19: Phospho (+79.97)	99.70%	4.45	25.8	8.42	4	822.89	3,287.53	0.02685
699	PE	Ig	3 min	299	328	KPAPPQGSRPESTVSFNPyEPTGGPWGPDR	y19: Phospho (+79.97)	99.70%	4.67	19.7	8.24	4	822.89	3,287.53	0.02661
700	PE	Ig	3 min	299	328	KPAPPQGSRPESTVSFNPyEPTGGPWGPDR	y19: Phospho (+79.97)	99.70%	5.42	32.7	10	4	822.89	3,287.53	0.02685
701	PE	Ig	3 min	299	328	KPAPPQGSRPESTVSFNPyEPTGGPWGPDR	y19: Phospho (+79.97)	99.70%	5.42	28.7	11.2	4	822.89	3,287.53	0.02197
702	PE	Ig	3 min	299	328	KPAPPQGSRPESTVSFNPyEPTGGPWGPDR	y19: Phospho (+79.97)	99.70%	4.99	19.5	9.72	4	822.89	3,287.51	0.00903
703	PE	Ig	3 min	299	328	KPAPPqGSRPESTVSFNPyEPTGGPWGPDR	q6: Deamidated (+0.98), y19: Phospho (+79.97)	99.70%	4.13	13	6.7	4	823.13	3,288.50	0.01647
704	PE	Ig	3 min	299	328	KPAPPQGSRPESTVSFNPyEPTGGPWGPDR	y19: Phospho (+79.97)	99.70%	3.18	21.4	9.21	3	1096.85	3,287.52	0.01777
705	PE	Ig	3 min	299	328	KPAPPQGSRPESTVSFNPyEPTGGPWGPDR	y19: Phospho (+79.97)	99.70%	5.21	27.1	12.8	4	822.89	3,287.51	0.00830
706	PE	Ig	3 min	299	328	KPAPPQGSRPESTVSFNPyEPTGGPWGPDR	y19: Phospho (+79.97)	99.70%	2.74	0	7.59	3	1096.85	3,287.54	0.03755
707	PE	Ig	3 min	299	328	KPAPPQGSRPESTVSFNPyEPTGGPWGPDR	y19: Phospho (+79.97)	99.70%	3.37	10	5.92	3	1096.86	3,287.55	0.04926
708	PE	Ig	3 min	299	328	KPAPPQGSRPESTVSFNPyEPTGGPWGPDR	y19: Phospho (+79.97)	99.70%	3.57	27.8	9.35	4	822.89	3,287.55	0.04345
709	PE	Ig	3 min	299	328	KPAPPQGSRPESTVSFNPyEPTGGPWGPDR	y19: Phospho (+79.97)	99.70%	2.62	15.5	9.92	3	1096.85	3,287.54	0.03864
710	PE	Ig	3 min	299	328	KPAPPQGSRPESTVSFNPyEPTGGPWGPDR	y19: Phospho (+79.97)	99.70%	2.56	18.2	9.82	3	1096.86	3,287.55	0.04597
711	PE	Ig	3 min	299	328	KPAPPQGSRPESTVSFNPyEPTGGPWGPDR	y19: Phospho (+79.97)	99.70%	3.13	33.8	11.8	4	822.90	3,287.56	0.05151
712	PE	Ig	3 min	299	328	KPAPPQGSRPESTVSFNPyEPTGGPWGPDR	y19: Phospho (+79.97)	99.70%	3.58	26.5	10.7	4	822.90	3,287.55	0.04809
713	PE	Ig	5 min	299	328	KPAPPQGSRPESTVSFNPyEPTGGPWGPDR	y19: Phospho (+79.97)	99.70%	2.14	24.5	6.23	4	822.89	3,287.52	0.01269
714	PE	Ig	5 min	299	328	KPAPPQGSRPESTVSFNPyEPTGGPWGPDR	y19: Phospho (+79.97)	96.70%	0	0	3.21	4	823.39	3,289.52	2.01100
715	PE	Ig	5 min	299	328	KPAPPQGSRPESTVSFNPyEPTGGPWGPDR	y19: Phospho (+79.97)	99.70%	0	0	8.17	4	822.89	3,287.54	0.03295
716	PE	Ig	5 min	299	328	KPAPPQGSRPESTVSFNPyEPTGGPWGPDR	y19: Phospho (+79.97)	99.70%	2.68	17.4	4.17	3	1096.85	3,287.52	0.01264
717	PE	Ig	5 min	299	328	KPAPPQGSRPESTVSFNPyEPTGGPWGPDR	y19: Phospho (+79.97)	99.70%	3.28	17	5.39	3	1096.85	3,287.52	0.01264
718	PE	Ig	5 min	299	328	KPAPPQGSRPESTVSFNPyEPTGGPWGPDR	y19: Phospho (+79.97)	99.70%	3.81	24	9.21	4	822.89	3,287.53	0.02978
719	PE	Ig	5 min	299	328	KPAPPQGSRPESTVSFNPyEPTGGPWGPDR	y19: Phospho (+79.97)	99.70%	5.21	23	13.6	4	822.89	3,287.52	0.02002
720	PE	Ig	5 min	299	328	KPAPPQGSRPESTVSFNPyEPTGGPWGPDR	y19: Phospho (+79.97)	99.70%	5.69	45.1	13.7	4	822.89	3,287.53	0.03003
721	PE	Ig	5 min	299	328	KPAPPqGSRPESTVSFNPyEPTGGPWGPDR	q6: Deamidated (+0.98), y19: Phospho (+79.97)	99.70%	3.51	0	7.85	4	823.13	3,288.50	0.00914
722	PE	Ig	5 min	299	328	KPAPPQGSRPESTVSFNPyEPTGGPWGPDR	y19: Phospho (+79.97)	99.70%	3.66	40.7	0	4	822.89	3,287.51	0.01074
723	PE	Ig	5 min	299	328	KPAPPQGSRPESTVSFNPyEPTGGPWGPDR	y19: Phospho (+79.97)	99.70%	0	0	7.06	4	822.90	3,287.57	0.06689

724	PE	Ig	5 min	299	328	KPAPPQGSRPESTVSFNPyEPTGGPWGPDR	y19: Phospho (+79.97)	99.70%	2.93	13.3	5.92	4	822.89	3,287.55	0.04321
725	PE	Ig	5 min	299	328	KPAPPQGSRPESTVSFNPyEPTGGPWGPDR	y19: Phospho (+79.97)	99.70%	2.05	23.7	7.82	3	1096.86	3,287.55	0.04633
726	PE	Ig	15 min	299	328	KPAPPQGSRPESTVSFNPyEPTGGPWGPDR	y19: Phospho (+79.97)	99.70%	3.42	0	5.85	4	822.89	3,287.53	0.03076
727	PE	Ig	15 min	299	328	KPAPPQGSRPESTVSFNPyEPTGGPWGPDR	y19: Phospho (+79.97)	99.70%	5.51	29.2	10.4	4	822.89	3,287.53	0.02270
728	PE	Ig	15 min	299	328	KPAPPqGSRPESTVSFnPyEPTGGPWGPDR	q6: Deamidated (+0.98), n17: Deamidated (+0.98), y19: Phospho (+79.97)	99.70%	4.53	17.6	8.8	4	823.38	3,289.49	0.01854
729	PE	Ig	15 min	299	328	KPAPPQGSRPESTVSFNPyEPTGGPWGPDR	y19: Phospho (+79.97)	99.70%	3.11	41.9	0	4	822.89	3,287.51	0.01049
730	PE	Ig	15 min	299	328	KPAPPQGSRPESTVSFNPyEPTGGPWGPDR	y19: Phospho (+79.97)	99.70%	3.05	24	5.48	4	822.89	3,287.53	0.03027
731	PE	Ig	15 min	299	328	KPAPPQGSRPESTVSFNPyEPTGGPWGPDR	y19: Phospho (+79.97)	99.70%	1.67	16.4	4.48	3	1096.86	3,287.55	0.04157
732	PE	Hg	0	299	328	KPAPPQGSRPESTVSFNPyEPTGGPWGPDR	y19: Phospho (+79.97)	99.70%	3.81	0	6.89	4	822.89	3,287.52	0.01757
733	PE	Hg	0	299	328	KPAPPQGSRPESTVSFNPYEPtGGPWGPDR	t22: Phospho (+79.97)	99.40%	0.85	16.1	0	4	822.88	3,287.50	0.00048
734	PE	Hg	0	299	328	KPAPPQGSRPESTVSFNPyEPTGGPWGPDR	y19: Phospho (+79.97)	99.70%	2.41	21.8	7.62	4	822.89	3,287.55	0.04394
735	PE	Hg	5 μM	299	328	KPAPPQGSRPESTVSFNPyEPTGGPWGPDR	y19: Phospho (+79.97)	99.70%	1.32	24.7	2.27	4	822.89	3,287.52	0.01367
736	PE	Hg	5 μM	299	328	KPAPPQGSRPESTVSFNPyEPTGGPWGPDR	y19: Phospho (+79.97)	99.70%	2.97	16.5	6.62	4	822.89	3,287.54	0.03247
737	PE	Hg	10 μM	299	328	KPAPPQGSRPESTVSFNPyEPTGGPWGPDR	y19: Phospho (+79.97)	99.70%	4.54	0	5.43	4	822.89	3,287.52	0.01196
738	PE	Hg	10 μM	299	328	KPAPPQGSRPESTVSFNPyEPTGGPWGPDR	y19: Phospho (+79.97)	99.70%	4.2	10.5	6.43	4	822.89	3,287.52	0.01098
739	PE	Hg	10 μM	299	328	KPAPPQGSRPESTVSFNPyEPTGGPWGPDR	y19: Phospho (+79.97)	99.70%	0	14	4.62	4	822.89	3,287.55	0.04150
740	PE	Hg	20 μM	299	328	KPAPPQGSRPESTVSFNPyEPTGGPWGPDR	y19: Phospho (+79.97)	99.70%	3.86	0	4.74	4	822.89	3,287.54	0.03100
741	PE	Hg	50 μM	299	328	KPAPPQGSRPESTVSFNPyEPTGGPWGPDR	y19: Phospho (+79.97)	99.70%	4.44	14.9	4.4	4	822.89	3,287.52	0.01586
742	PE	Hg	50 μM	299	328	KPAPPQGSRPESTVSFnPyEPTGGPWGPDR	n17: Deamidated (+0.98), y19: Phospho (+79.97)	99.70%	3.19	0	4.92	4	823.13	3,288.50	0.01622
743	PE	Hg	50 μM	299	328	KPAPPQGSRPESTVSFNPyEPTGGPWGPDR	y19: Phospho (+79.97)	99.70%	0	19	7.7	4	822.89	3,287.54	0.03247
744	PE	Hg	50 μM	299	328	KPAPPQGSRPESTVSFNPyEPTGGPWGPDR	y19: Phospho (+79.97)	99.70%	5.62	12.9	7.22	4	822.89	3,287.52	0.01635
745	PE	Hg	50 μM	299	328	KPAPPQGSRPESTVSFNPyEPTGGPWGPDR	y19: Phospho (+79.97)	99.70%	5.84	26.3	9.25	4	822.89	3,287.53	0.02807
746	PE	Hg	50 μM	299	328	KPAPPQGSRPESTVSFNPyEPTGGPWGPDR	y19: Phospho (+79.97)	99.70%	5.26	22.8	9.16	4	822.89	3,287.53	0.02246
747	PE	Hg	50 μM	299	328	KPAPPQGSRPESTVSFNPyEPTGGPWGPDR	y19: Phospho (+79.97)	99.70%	5.63	21.9	10.2	4	822.89	3,287.51	0.01049
748	PE	Hg	50 μM	299	328	KPAPPQGSRPESTVSFNPyEPTGGPWGPDR	y19: Phospho (+79.97)	99.70%	4.91	0	7.32	3	1096.84	3,287.50	-0.00091
749	PE	Hg	50 μM	299	328	KPAPPQGSRPESTVSFNPyEPTGGPWGPDR	y19: Phospho (+79.97)	99.70%	5.03	24.8	9.82	4	822.89	3,287.51	0.00854
750	PE	Hg	50 μM	299	328	KPAPPQGSRPESTVSFNPyEPTGGPWGPDR	y19: Phospho (+79.97)	99.70%	5.72	16.9	9.82	4	822.88	3,287.50	-0.00147
751	PE	Hg	50 μM	299	328	KPAPPQGSRPESTVSFNPyEPTGGPWGPDR	y19: Phospho (+79.97)	99.70%	1.91	16.1	3.37	4	822.89	3,287.52	0.01367
752	PE	Hg	50 μM	299	328	KPAPPQGSRPESTVSFNPyEPTGGPWGPDR	y19: Phospho (+79.97)	99.70%	3.06	0	3.55	3	1096.86	3,287.57	0.06172
753	PE	Hg	50 μM	299	328	KPAPPQGSRPESTVSFnPyEPTGGPWGPDR	n17: Deamidated (+0.98), y19: Phospho (+79.97)	99.70%	3.02	25.2	6.01	4	823.14	3,288.55	0.05944
754	PE	Hg	50 μM	299	328	KPAPPQGSRPESTVSFNPyEPTGGPWGPDR	y19: Phospho (+79.97)	99.70%	3.44	0	6.59	4	822.90	3,287.56	0.05200
755	PE	Hg	50 μM	299	328	KPAPPQGSRPESTVSFNPyEPTGGPWGPDR	y19: Phospho (+79.97)	99.70%	2.99	13.9	7.54	4	822.90	3,287.56	0.05835
756	PE	Hg	50 μM	299	328	KPAPPQGSRPESTVSFNPyEPTGGPWGPDR	y19: Phospho (+79.97)	99.70%	3.35	19	8.62	3	1096.86	3,287.55	0.04185
757	PE	Hg	50 μM	299	328	KPAPPQGSRPESTVSFNPyEPTGGPWGPDR	y19: Phospho (+79.97)	99.70%	2.82	19	9.64	3	1096.86	3,287.56	0.05293
758	PE	Hg	50 μM	299	328	KPAPPQGSRPESTVSFNPyEPTGGPWGPDR	y19: Phospho (+79.97)	99.70%	3.37	14.9	8.96	4	822.89	3,287.55	0.04296
759	PE	Hg	50 μM	299	328	KPAPPQGSRPESTVSFNPyEPTGGPWGPDR	y19: Phospho (+79.97)	99.70%	2.55	18.9	9.96	3	1096.86	3,287.56	0.05732
760	PE	Hg	50 μM	299	328	KPAPPQGSRPESTVSFNPyEPTGGPWGPDR	y19: Phospho (+79.97)	99.70%	3.73	20.4	8.92	4	822.89	3,287.55	0.04296
761	PE	Hg	50 μM	299	328	KPAPPQGSRPESTVSFNPyEPTGGPWGPDR	y19: Phospho (+79.97)	99.70%	3.38	26.9	10.6	4	822.89	3,287.55	0.04394
762	PE	Hg	50 μM	299	328	KPAPPQGSRPESTVSFNPyEPTGGPWGPDR	y19: Phospho (+79.97)	99.70%	3.41	26.7	10.6	4	822.89	3,287.55	0.04638
763	PE	Hg	50 μM	299	328	KPAPPQGSRPESTVSFNPyEPTGGPWGPDR	y19: Phospho (+79.97)	99.70%	3.65	28.4	11	4	822.89	3,287.55	0.04614
764	PE	Hg	50 μM	299	328	KPAPPQGSRPESTVSFNPyEPTGGPWGPDR	y19: Phospho (+79.97)	99.70%	3.4	20	10.9	3	1096.86	3,287.54	0.03938

765	PE	Hg	50 µM	299	328	KPAPPQGSRPESTVSFNPyEPTGGPWGPDR	y19: Phospho (+79.97)	99.70%	3.91	30	12	4	822.90	3,287.55	0.04956
766	PE	Hg	50 µM	299	328	KPAPPQGSRPESTVSFNPyEPTGGPWGPDR	y19: Phospho (+79.97)	99.70%	3.78	23.3	10.2	4	822.89	3,287.55	0.04126
767	PE	Hg	100 µM	299	328	KPAPPQGSRPESTVSFNPyEPTGGPWGPDR	y19: Phospho (+79.97)	99.70%	2.62	20.7	6.89	3	1096.85	3,287.51	0.00971
768	PE	Hg	100 µM	299	328	KPAPPQGSRPESTVSFNPyEPTGGPWGPDR	y19: Phospho (+79.97)	99.70%	3.86	22.7	11.1	4	822.88	3,287.49	-0.01099
769	PE	Hg	100 µM	299	328	KPAPPqGSRPESTVSFNPyEPTGGPWGPDR	q6: Deamidated (+0.98), y19: Phospho (+79.97)	99.20%	0	28.1	9.32	4	823.14	3,288.52	0.02819
770	PE	Hg	100 µM	299	328	KPAPPQGSRPESTVSFNPyEPTGGPWGPDR	y19: Phospho (+79.97)	99.50%	0	27	11	4	822.89	3,287.53	0.02563
771	PE	Hg	100 µM	299	328	KPAPPQGSRPESTVSFNPyEPTGGPWGPDR	y19: Phospho (+79.97)	99.60%	0	23.1	11.1	4	822.89	3,287.51	0.00854
772	PE	Hg	100 µM	299	328	KPAPPQGSRPESTVSFNPyEPTGGPWGPDR	y19: Phospho (+79.97)	99.70%	0	21.9	11.1	4	822.89	3,287.52	0.01220
773	PE	Hg	100 µM	299	328	KPAPPQGSRPESTVSFNPyEPTGGPWGPDR	y19: Phospho (+79.97)	99.70%	0	30.2	13.3	4	822.89	3,287.53	0.02661
774	PE	Hg	100 µM	299	328	KPAPPqGSRPESTVSFNPyEPTGGPWGPDR	q6: Deamidated (+0.98), y19: Phospho (+79.97)	99.70%	0	19.4	6.09	4	823.14	3,288.51	0.02355
775	PE	Hg	100 µM	299	328	KPAPPQGSRPESTVSFNPyEPTGGPWGPDR	y19: Phospho (+79.97)	99.70%	0	16.4	6.6	3	1096.85	3,287.53	0.02143
776	PE	Hg	100 µM	299	328	KPAPPQGSRPESTVSFNPyEPTGGPWGPDR	y19: Phospho (+79.97)	99.70%	0	11.3	7.12	4	822.89	3,287.52	0.01562
777	PE	Hg	100 µM	299	328	KPAPPQGSRPESTVSFNPyEPTGGPWGPDR	y19: Phospho (+79.97)	99.70%	0	12	8	3	1096.85	3,287.52	0.01740
778	PE	Hg	100 µM	299	328	KPAPPQGSRPESTVSFNPyEPTGGPWGPDR	y19: Phospho (+79.97)	99.70%	0	20.8	14.9	3	1096.85	3,287.52	0.01740
779	PE	Hg	100 µM	299	328	KPAPPqGSRPESTVSFNPyEPTGGPWGPDR	q6: Deamidated (+0.98), y19: Phospho (+79.97)	99.70%	0	0	10.1	4	823.14	3,288.52	0.03331
780	PE	Hg	100 µM	299	328	KPAPPQGSRPESTVSFNPyEPTGGPWGPDR	y19: Phospho (+79.97)	99.70%	4.2	16.5	6.47	4	822.89	3,287.51	0.00708
781	PE	Hg	100 µM	299	328	KPAPPQGSRPESTVSFNPyEPTGGPWGPDR	y19: Phospho (+79.97)	99.70%	4.83	11.1	7.27	4	822.88	3,287.51	0.00268
782	PE	Hg	100 µM	299	328	KPAPPqGSRPESTVSFnPyEPTGGPWGPDR	q6: Deamidated (+0.98), n17: Deamidated (+0.98), y19: Phospho (+79.97)	99.70%	4.99	24.7	9.89	4	823.38	3,289.50	0.02806
783	PE	Hg	100 µM	299	328	KPAPPQGSRPESTVSFNPyEPTGGPWGPDR	y19: Phospho (+79.97)	99.70%	0	0	7.05	4	823.40	3,289.55	2.04800
784	PE	Hg	100 µM	299	328	KPAPPQGSRPESTVSFNPyEPTGGPWGPDR	y19: Phospho (+79.97)	99.70%	2.83	13.1	5.43	3	1096.86	3,287.55	0.04487
785	PE	Hg	100 µM	299	328	KPAPPQGSRPESTVSFNPyEPTGGPWGPDR	y19: Phospho (+79.97)	99.70%	2.56	16	6.52	3	1096.86	3,287.55	0.04414
786	PE	Hg	100 µM	299	328	KPAPPQGSRPESTVSFNPyEPTGGPWGPDR	y19: Phospho (+79.97)	99.70%	2.96	10.6	7.03	4	822.90	3,287.55	0.04809
787	PE	Hg	100 µM	299	328	KPAPPQGSRPESTVSFNPyEPTGGPWGPDR	y19: Phospho (+79.97)	99.70%	3.49	19.4	11.4	4	822.90	3,287.56	0.05639
788	PE	Hg	100 µM	299	328	KPAPPQGSRPESTVSFNPyEPTGGPWGPDR	y19: Phospho (+79.97)	99.70%	3.23	22.6	11.2	3	1096.86	3,287.57	0.06245
789	PE	Hg	100 µM	299	328	KPAPPQGSRPESTVSFNPyEPTGGPWGPDR	y19: Phospho (+79.97)	99.70%	3.49	19.8	12	4	822.89	3,287.55	0.04492
790	PE	Hg	100 µM	299	328	KPAPPQGSRPESTVSFNPyEPTGGPWGPDR	y19: Phospho (+79.97)	99.70%	2.9	31.5	11.7	4	822.90	3,287.55	0.04956
791	PE	Hg	100 µM	299	328	KPAPPQGSRPESTVSFNPyEPTGGPWGPDR	y19: Phospho (+79.97)	99.70%	3.48	20.5	11	4	822.89	3,287.55	0.04663
792	PE	PV	0	299	328	KPAPPQGSRPESTVSFNPyEPTGGPWGPDR	y19: Phospho (+79.97)	99.70%	4.63	11.9	5.92	4	822.89	3,287.53	0.02417
793	PE	PV	0	299	328	KPAPPQGSRPESTVSFNPyEPTGGPWGPDR	y19: Phospho (+79.97)	99.70%	2.41	21.8	7.62	4	822.89	3,287.55	0.04394
794	PE	PV	1 µM	299	328	KPAPPQGSRPESTVSFNPyEPTGGPWGPDR	y19: Phospho (+79.97)	93.00%	2.4	0	3.8	4	822.89	3,287.52	0.01904
795	PE	PV	3 µM	299	328	KPAPPQGSRPESTVSFNPyEPTGGPWGPDR	y19: Phospho (+79.97)	99.70%	3.95	14.5	6.42	4	822.89	3,287.52	0.01586
796	PE	PV	10 µM	299	328	KPAPPQGSRPESTVSFNPyEPTGGPWGPDR	y19: Phospho (+79.97)	99.60%	1.88	12.9	2.27	4	822.89	3,287.51	0.00781
797	PE	PV	10 µM	299	328	KPAPPQGSRPESTVSFNPyEPTGGPWGPDR	y19: Phospho (+79.97)	99.70%	3.2	22.2	8.92	3	1096.85	3,287.51	0.00971
798	PE	PV	10 µM	299	328	KPAPPQGSRPESTVSFNPyEPTGGPWGPDR	y19: Phospho (+79.97)	99.70%	5.17	12.5	8.04	3	1096.84	3,287.51	0.00202
799	PE	PV	10 µM	299	328	KPAPPQGSRPESTVSFNPyEPTGGPWGPDR	y19: Phospho (+79.97)	99.70%	5.23	31.4	11.7	4	822.88	3,287.50	-0.00025
800	PE	PV	10 µM	299	328	KPAPPqGSRPESTVSFNPyEPTGGPWGPDR	q6: Deamidated (+0.98), y19: Phospho (+79.97)	99.70%	2.65	22.5	5.7	3	1097.18	3,288.50	0.01520
801	PE	PV	10 µM	299	328	KPAPPQGSRPESTVSFNPyEPTGGPWGPDR	y19: Phospho (+79.97)	99.70%	4.8	0	5.66	4	822.89	3,287.52	0.01440
802	PE	PV	10 µM	299	328	KPAPPQGSRPESTVSFNPyEPTGGPWGPDR	y19: Phospho (+79.97)	99.70%	3.01	22.6	8.18	3	1096.85	3,287.52	0.01631
803	PE	PV	10 µM	299	328	KPAPPQGSRPESTVSFNPyEPTGGPWGPDR	y19: Phospho (+79.97)	99.70%	4.43	24.6	10.5	3	1096.85	3,287.52	0.01667

804	PE	PV	10 μM	299	328	KPAPPQGSRPESTVSFNPyEPTGGPWGPDR	y19: Phospho (+79.97)	99.70%	5.39	35.5	8.96	4	822.89	3,287.52	0.01269
805	PE	PV	10 μM	299	328	KPAPPQGSRPESTVSFNPyEPTGGPWGPDR	y19: Phospho (+79.97)	99.70%	3.59	25.6	12.1	3	1096.85	3,287.52	0.02033
806	PE	PV	10 μM	299	328	KPAPPQGSRPESTVSFNPyEPTGGPWGPDR	y19: Phospho (+79.97)	99.70%	5.26	32.8	10.3	4	822.89	3,287.52	0.01611
807	PE	PV	10 μM	299	328	KPAPPQGSRPESTVSFNPyEPTGGPWGPDR	y19: Phospho (+79.97)	99.70%	6.14	25	9.85	4	822.89	3,287.51	0.00756
808	PE	PV	10 μM	299	328	KPAPPQGSRPESTVSFNPyEPTGGPWGPDR	y19: Phospho (+79.97)	99.70%	6.26	30.2	11.8	4	822.89	3,287.51	0.01049
809	PE	PV	10 μM	299	328	KPAPPQGSRPESTVSFNPyEPTGGPWGPDR	y19: Phospho (+79.97)	99.70%	7.35	32.4	12.4	4	822.89	3,287.52	0.01294
810	PE	PV	10 μM	299	328	KPAPPqGSRPESTVSFNPyEPTGGPWGPDR	q6: Deamidated (+0.98), y19: Phospho (+79.97)	99.70%	4.29	48.5	9.92	4	823.14	3,288.52	0.03112
811	PE	PV	10 μM	299	328	KPAPPqGSRPESTVSFNPyEPTGGPWGPDR	q6: Deamidated (+0.98), y19: Phospho (+79.97)	99.70%	5.31	36	13.5	4	823.13	3,288.51	0.01867
812	PE	PV	10 μM	299	328	KPAPPqGSRPESTVSFNPyEPTGGPWGPDR	q6: Deamidated (+0.98), y19: Phospho (+79.97)	99.70%	4.95	42.8	11.2	4	823.13	3,288.51	0.02111
813	PE	PV	10 μM	299	328	KPAPPQGSRPESTVSFNPyEPTGGPWGPDR	y19: Phospho (+79.97)	99.70%	6.39	37.8	14.5	4	822.89	3,287.51	0.01025
814	PE	PV	10 μM	299	328	KPAPPqGSRPESTVSFNPyEPTGGPWGPDR	q6: Deamidated (+0.98), y19: Phospho (+79.97)	99.70%	5.51	37.6	13.1	4	823.13	3,288.50	0.01598
815	PE	PV	10 μM	299	328	KPAPPQGSRPESTVSFNPyEPTGGPWGPDR	y19: Phospho (+79.97)	99.70%	6.35	36.2	12.8	4	822.89	3,287.52	0.01171
816	PE	PV	10 μM	299	328	KPAPPqGSRPESTVSFNPyEPTGGPWGPDR	q6: Deamidated (+0.98), y19: Phospho (+79.97)	99.70%	5.31	44.6	12.1	4	823.13	3,288.51	0.01818
817	PE	PV	10 μM	299	328	KPAPPQGSRPESTVSFNPyEPTGGPWGPDR	y19: Phospho (+79.97)	99.70%	6.64	37.5	14.8	4	822.89	3,287.52	0.01171
818	PE	PV	10 μM	299	328	KPAPPQGSRPESTVSFNPyEPTGGPWGPDR	y19: Phospho (+79.97)	99.70%	6.07	25	11.4	3	1096.85	3,287.51	0.01081
819	PE	PV	10 μM	299	328	KPAPPQGSRPESTVSFNPyEPTGGPWGPDR	y19: Phospho (+79.97)	99.70%	5.95	21.5	10.5	3	1096.85	3,287.52	0.01557
820	PE	PV	10 μM	299	328	KPAPPQGSRPESTVSFNPyEPTGGPWGPDR	y19: Phospho (+79.97)	99.70%	6.03	21.6	15.3	3	1096.85	3,287.52	0.01704
821	PE	PV	10 μM	299	328	KPAPPQGSRPESTVSFNPyEPTGGPWGPDR	y19: Phospho (+79.97)	99.70%	5.14	36.5	15.1	3	1096.85	3,287.52	0.01338
822	PE	PV	10 μM	299	328	KPAPPqGSRPESTVSFNPyEPTGGPWGPDR	q6: Deamidated (+0.98), y19: Phospho (+79.97)	99.70%	2.77	14.2	4.08	3	1097.17	3,288.49	0.00019
823	PE	PV	10 μM	299	328	KPAPPqGSRPESTVSFnPyEPTGGPWGPDR	q6: Deamidated (+0.98), n17: Deamidated (+0.98), y19: Phospho (+79.97)	99.70%	0	0	11.1	3	1097.84	3,290.49	1.02200
824	PE	PV	10 μM	299	328	KPAPPqGSRPESTVSFNPyEPTGGPWGPDR	q6: Deamidated (+0.98), y19: Phospho (+79.97)	99.70%	3.44	24	6.92	3	1097.17	3,288.48	-0.00824
825	PE	PV	10 μM	299	328	KPAPPQGSRPESTVSFNPyEPTGGPWGPDR	y19: Phospho (+79.97)	99.70%	5.36	31.7	9.55	4	822.89	3,287.51	0.00854
826	PE	PV	10 μM	299	328	KPAPPQGSRPESTVSFNPyEPTGGPWGPDR	y19: Phospho (+79.97)	99.70%	5.97	35.7	11.7	4	822.88	3,287.51	0.00561
827	PE	PV	10 μM	299	328	KPAPPQGSRPESTVSFNPyEPTGGPWGPDR	y19: Phospho (+79.97)	99.70%	3.62	26	9.85	4	822.88	3,287.49	-0.01123
828	PE	PV	10 μM	299	328	KPAPPQGSRPESTVSFNPyEPTGGPWGPDR	y19: Phospho (+79.97)	99.70%	3.06	20.3	14.8	3	1096.85	3,287.53	0.03022
829	PE	PV	10 μM	299	328	KPAPPqGSRPESTVSFNPyEPTGGPWGPDR	q6: Deamidated (+0.98), y19: Phospho (+79.97)	99.70%	3.61	12.8	7.2	3	1097.18	3,288.51	0.01959
830	PE	PV	10 μM	299	328	KPAPPqGSRPESTVSFNPyEPTGGPWGPDR	q6: Deamidated (+0.98), y19: Phospho (+79.97)	99.70%	4.18	27.3	11.9	4	823.13	3,288.50	0.01256
831	PE	PV	10 μM	299	328	KPAPPqGSRPESTVSFNPyEPTGGPWGPDR	q6: Deamidated (+0.98), y19: Phospho (+79.97)	99.70%	5.21	32.1	11.5	4	823.13	3,288.50	0.01574
832	PE	PV	10 μM	299	328	KPAPPqGSRPESTVSFNPyEPTGGPWGPDR	q6: Deamidated (+0.98), y19: Phospho (+79.97)	99.70%	4.68	35	9.34	4	823.13	3,288.50	0.01525
833	PE	PV	10 μM	299	328	KPAPPqGSRPESTVSFNPyEPTGGPWGPDR	q6: Deamidated (+0.98), y19: Phospho (+79.97)	99.70%	4.77	20.1	10	3	1097.18	3,288.51	0.02509

834	PE	PV	10 µM	299	328	KPAPPqGSRPESTVSFNPyEPTGGPWGPDR	q6: Deamidated (+0.98), y19: Phospho (+79.97)	99.70%	5	31.6	10.2	4	823.13	3,288.50	0.00841
835	PE	PV	10 µM	299	328	KPAPPQGSRPESTVSFNPyEPTGGPWGPDR	y19: Phospho (+79.97)	99.70%	5.22	36.9	11.3	4	822.88	3,287.51	0.00390
836	PE	PV	10 µM	299	328	KPAPPQGSRPESTVSFNPyEPTGGPWGPDR	y19: Phospho (+79.97)	99.70%	5.61	26.8	9.7	4	822.88	3,287.50	-0.00196
837	PE	PV	10 µM	299	328	KPAPPqGSRPESTVSFNPyEPTGGPWGPDR	q6: Deamidated (+0.98), y19: Phospho (+79.97)	99.70%	5.64	39.4	12.7	4	823.13	3,288.50	0.00939
838	PE	PV	10 µM	299	328	KPAPPQGSRPESTVSFNPyEPTGGPWGPDR	y19: Phospho (+79.97)	99.70%	5.83	35	10.6	4	822.88	3,287.51	0.00366
839	PE	PV	10 µM	299	328	KPAPPQGSRPESTVSFNPyEPTGGPWGPDR	y19: Phospho (+79.97)	99.70%	4.98	21.9	13.9	3	1096.85	3,287.53	0.02509
840	PE	PV	10 µM	299	328	KPAPPQGSRPESTVSFNPyEPTGGPWGPDR	y19: Phospho (+79.97)	99.70%	5.82	14.7	13	3	1096.84	3,287.50	-0.00164
841	PE	PV	10 µM	299	328	KPAPPQGSRPESTVSFNPyEPTGGPWGPDR	y19: Phospho (+79.97)	99.70%	5.61	22.1	7.66	3	1096.85	3,287.52	0.01887
842	PE	PV	10 µM	299	328	KPAPPQGSRPESTVSFNPyEPTGGPWGPDR	y19: Phospho (+79.97)	99.70%	6.45	36.2	13.3	4	822.88	3,287.51	0.00390
843	PE	PV	10 µM	299	328	KPAPPQGSRPESTVSFNPyEPTGGPWGPDR	y19: Phospho (+79.97)	99.70%	6.57	26.6	12.5	3	1096.84	3,287.51	0.00276
844	PE	PV	10 µM	299	328	KPAPPQGSRPESTVSFNPyEPTGGPWGPDR	y19: Phospho (+79.97)	99.70%	6.62	38.5	12.7	3	1096.85	3,287.53	0.02143
845	PE	PV	10 µM	299	328	KPAPPQGSRPESTVSFNPyEPTGGPWGPDR	y19: Phospho (+79.97)	99.70%	3.39	27.6	6.51	4	822.89	3,287.51	0.00732
846	PE	PV	10 µM	299	328	KPAPPQGSRPESTVSFnPyEPTGGPWGPDR	n17: Deamidated (+0.98), y19: Phospho (+79.97)	99.70%	0	0	10.2	3	1097.53	3,289.56	1.07000
847	PE	PV	10 µM	299	328	KPAPPQGSRPESTVSFNPyEPTGGPWGPDR	y19: Phospho (+79.97)	99.70%	0	15.9	5.74	3	1096.86	3,287.55	0.04341
848	PE	PV	10 µM	299	328	KPAPPQGSRPESTVSFNPyEPTGGPWGPDR	y19: Phospho (+79.97)	99.70%	2.13	50.5	6.85	4	822.89	3,287.54	0.03881
849	PE	PV	10 µM	299	328	KPAPPQGSRPESTVSFNPyEPTGGPWGPDR	y19: Phospho (+79.97)	99.70%	4.47	22.3	6.43	3	1096.86	3,287.56	0.05183
850	PE	PV	10 µM	299	328	KPAPPQGSRPESTVSFNPyEPTGGPWGPDR	y19: Phospho (+79.97)	99.70%	3.14	0	6.74	3	1096.86	3,287.55	0.05073
851	PE	PV	10 µM	299	328	KPAPPQGSRPESTVSFNPyEPTGGPWGPDR	y19: Phospho (+79.97)	99.70%	2.64	18.6	8.54	3	1096.86	3,287.56	0.05403
852	PE	PV	10 µM	299	328	KPAPPQGSRPESTVSFNPyEPTGGPWGPDR	y19: Phospho (+79.97)	99.70%	3.82	14.9	9.06	3	1096.86	3,287.56	0.05403
853	PE	PV	10 µM	299	328	KPAPPQGSRPESTVSFNPyEPTGGPWGPDR	y19: Phospho (+79.97)	99.70%	3.38	12.9	10.7	3	1096.86	3,287.56	0.05622
854	PE	PV	10 µM	299	328	KPAPPQGSRPESTVSFNPyEPTGGPWGPDR	y19: Phospho (+79.97)	99.70%	3.15	26.5	12.3	3	1096.86	3,287.56	0.05219
855	PE	PV	10 µM	299	328	KPAPPQGSRPESTVSFNPyEPTGGPWGPDR	y19: Phospho (+79.97)	99.70%	3.34	20.4	12.1	3	1096.86	3,287.55	0.05073
856	PE	PV	10 µM	299	328	KPAPPQGSRPESTVSFNPyEPTGGPWGPDR	y19: Phospho (+79.97)	99.70%	3.5	25.6	11.1	4	822.90	3,287.55	0.04858
857	PE	PV	10 µM	299	328	KPAPPQGSRPESTVSFNPyEPTGGPWGPDR	y19: Phospho (+79.97)	99.70%	3.62	28.2	11.7	4	822.90	3,287.56	0.05175
858	PE	PV	10 µM	299	328	KPAPPQGSRPESTVSFNPyEPTGGPWGPDR	y19: Phospho (+79.97)	99.70%	4.03	17.8	11	3	1096.86	3,287.55	0.04817
859	PE	PV	10 µM	299	328	KPAPPQGSRPESTVSFNPyEPTGGPWGPDR	y19: Phospho (+79.97)	99.70%	3.53	26.3	11	4	822.90	3,287.55	0.05004
860	PE	PV	10 µM	299	328	KPAPPQGSRPESTVSFNPyEPTGGPWGPDR	y19: Phospho (+79.97)	99.70%	3.82	34.1	11.6	4	822.90	3,287.55	0.04834
861	PE	PV	10 µM	299	328	KPAPPQGSRPESTVSFNPyEPTGGPWGPDR	y19: Phospho (+79.97)	99.70%	3.39	30.4	11.2	4	822.90	3,287.55	0.04956
862	PE	PV	10 µM	299	328	KPAPPQGSRPESTVSFNPyEPTGGPWGPDR	y19: Phospho (+79.97)	99.70%	3.66	32	11.6	4	822.90	3,287.56	0.05444
863	PE	PV	10 µM	299	328	KPAPPQGSRPESTVSFNPyEPTGGPWGPDR	y19: Phospho (+79.97)	99.70%	3.62	26.2	11.2	4	822.89	3,287.55	0.04419
864	PE	PV	10 µM	299	328	KPAPPQGSRPESTVSFNPyEPTGGPWGPDR	y19: Phospho (+79.97)	99.70%	3.78	28.9	10.1	4	822.90	3,287.55	0.04711
865	PE	PV	10 µM	299	328	KPAPPQGSRPESTVSFNPyEPTGGPWGPDR	y19: Phospho (+79.97)	99.70%	3.66	24.2	12.6	4	822.90	3,287.55	0.04711
866	PE	PV	30 µM	299	328	KPAPPQGSRPESTVSFNPyEPTGGPWGPDR	y19: Phospho (+79.97)	99.70%	3.59	17.8	7.7	4	822.89	3,287.52	0.01977
867	PE	PV	30 µM	299	328	KPAPPQGSRPESTVSFNPyEPTGGPWGPDR	y19: Phospho (+79.97)	99.70%	3.82	23.2	13	3	1096.84	3,287.51	0.00678
868	PE	PV	30 µM	299	328	KPAPPQGSRPESTVSFNPyEPTGGPWGPDR	y19: Phospho (+79.97)	99.70%	4.41	37.8	7.54	4	822.88	3,287.51	0.00537
869	PE	PV	30 µM	299	328	KPAPPQGSRPESTVSFNPyEPTGGPWGPDR	y19: Phospho (+79.97)	99.70%	5.67	34.8	12.1	4	822.88	3,287.51	0.00659
870	PE	PV	30 µM	299	328	KPAPPQGSRPESTVSFNPyEPTGGPWGPDR	y19: Phospho (+79.97)	99.70%	4.91	22.3	12	3	1096.85	3,287.52	0.01594
871	PE	PV	30 µM	299	328	KPAPPQGSRPESTVSFNPyEPTGGPWGPDR	y19: Phospho (+79.97)	99.70%	5.25	17	6.62	4	822.89	3,287.52	0.01489
872	PE	PV	30 µM	299	328	KPAPPQGSRPESTVSFNPyEPTGGPWGPDR	y19: Phospho (+79.97)	99.70%	3.78	21.8	11.7	3	1096.85	3,287.53	0.02143
873	PE	PV	30 µM	299	328	KPAPPQGSRPESTVSFNPyEPTGGPWGPDR	y19: Phospho (+79.97)	99.70%	5.22	22.1	8.74	4	822.89	3,287.51	0.00854
874	PE	PV	30 µM	299	328	KPAPPQGSRPESTVSFNPyEPTGGPWGPDR	y19: Phospho (+79.97)	99.70%	5.43	34	10.1	4	822.89	3,287.51	0.01025
875	PE	PV	30 µM	299	328	KPAPPQGSRPESTVSFNPyEPTGGPWGPDR	y19: Phospho (+79.97)	99.70%	6.75	31.5	12	4	822.89	3,287.52	0.01489

876	PE	PV	30 μM	299	328	KPAPPqGSRPESTVSFNPyEPTGGPWGPDR	q6: Deamidated (+0.98), y19: Phospho (+79.97)	99.70%	5.04	30.5	13.3	4	823.13	3,288.50	0.01622
877	PE	PV	30 μM	299	328	KPAPPQGSRPESTVSFnPyEPTGGPWGPDR	n17: Deamidated (+0.98), y19: Phospho (+79.97)	99.70%	3.42	16.7	4.43	3	1097.18	3,288.52	0.02948
878	PE	PV	30 μM	299	328	KPAPPQGSRPESTVSFnPyEPTGGPWGPDR	n17: Deamidated (+0.98), y19: Phospho (+79.97)	99.70%	3.65	25.5	9.09	3	1097.18	3,288.51	0.02399
879	PE	PV	30 μM	299	328	KPAPPQGSRPESTVSFnPyEPTGGPWGPDR	n17: Deamidated (+0.98), y19: Phospho (+79.97)	99.70%	5.11	34.8	13.3	4	823.13	3,288.51	0.01915
880	PE	PV	30 μM	299	328	KPAPPQGSRPESTVSFNPyEPTGGPWGPDR	y19: Phospho (+79.97)	99.70%	6.45	28.5	12.9	4	822.89	3,287.52	0.01171
881	PE	PV	30 μM	299	328	KPAPPqGSRPESTVSFNPyEPTGGPWGPDR	q6: Deamidated (+0.98), y19: Phospho (+79.97)	99.70%	4.56	25.6	13.1	4	823.14	3,288.51	0.02282
882	PE	PV	30 μM	299	328	KPAPPQGSRPESTVSFNPyEPTGGPWGPDR	y19: Phospho (+79.97)	99.70%	6.88	37.3	14.3	4	822.89	3,287.52	0.01220
883	PE	PV	30 μM	299	328	KPAPPQGSRPESTVSFnPyEPTGGPWGPDR	n17: Deamidated (+0.98), y19: Phospho (+79.97)	99.70%	3.87	22.8	6.7	3	1097.18	3,288.51	0.01996
884	PE	PV	30 μM	299	328	KPAPPQGSRPESTVSFNPyEPTGGPWGPDR	y19: Phospho (+79.97)	99.70%	5.76	29.4	12.2	4	822.89	3,287.51	0.00756
885	PE	PV	30 μM	299	328	KPAPPQGSRPESTVSFNPyEPTGGPWGPDR	y19: Phospho (+79.97)	99.70%	4.25	15.2	11.6	3	1096.85	3,287.52	0.01924
886	PE	PV	30 μM	299	328	KPAPPQGSRPESTVSFNPyEPTGGPWGPDR	y19: Phospho (+79.97)	99.70%	6.35	36.9	13.3	4	822.89	3,287.52	0.01220
887	PE	PV	30 μM	299	328	KPAPPqGSRPESTVSFNPyEPTGGPWGPDR	q6: Deamidated (+0.98), y19: Phospho (+79.97)	99.70%	5.2	46.8	14.4	4	823.13	3,288.51	0.01720
888	PE	PV	30 μM	299	328	KPAPPQGSRPESTVSFNPyEPTGGPWGPDR	y19: Phospho (+79.97)	99.70%	6.87	36.6	14.4	4	822.89	3,287.52	0.01196
889	PE	PV	30 μM	299	328	KPAPPQGSRPESTVSFNPyEPTGGPWGPDR	y19: Phospho (+79.97)	99.70%	6.66	31.7	14.2	4	822.89	3,287.52	0.01123
890	PE	PV	30 μM	299	328	KPAPPQGSRPESTVSFNPyEPTGGPWGPDR	y19: Phospho (+79.97)	99.70%	4.72	23.5	10.2	3	1096.85	3,287.52	0.02033
891	PE	PV	30 μM	299	328	KPAPPQGSRPESTVSFNPyEPTGGPWGPDR	y19: Phospho (+79.97)	99.70%	4.99	22.2	11.2	3	1096.85	3,287.52	0.01411
892	PE	PV	30 μM	299	328	KPAPPQGSRPESTVSFNPyEPTGGPWGPDR	y19: Phospho (+79.97)	99.70%	5.13	23.1	8.39	3	1096.85	3,287.52	0.02033
893	PE	PV	30 μM	299	328	KPAPPQGSRPESTVSFNPyEPTGGPWGPDR	y19: Phospho (+79.97)	99.70%	7.73	38.3	13.1	4	822.89	3,287.52	0.01245
894	PE	PV	30 μM	299	328	KPAPPQGSRPESTVSFNPyEPTGGPWGPDR	y19: Phospho (+79.97)	99.70%	5.42	33.8	13.5	3	1096.85	3,287.52	0.01521
895	PE	PV	30 μM	299	328	KPAPPQGSRPESTVSFNPyEPTGGPWGPDR	y19: Phospho (+79.97)	99.70%	4.99	21.6	7.22	3	1096.85	3,287.52	0.01521
896	PE	PV	30 μM	299	328	KPAPPQGSRPESTVSFNPyEPTGGPWGPDR	y19: Phospho (+79.97)	99.70%	3.97	24.8	0	5	658.51	3,287.50	-0.00356
897	PE	PV	30 μM	299	328	KPAPPQGSRPESTVSFNPyEPTGGPWGPDR	y19: Phospho (+79.97)	99.70%	5.56	35.5	10.7	4	822.89	3,287.51	0.00756
898	PE	PV	30 μM	299	328	KPAPPQGSRPESTVSFNPyEPTGGPWGPDR	y19: Phospho (+79.97)	99.70%	5.72	33.2	10.3	4	822.88	3,287.51	0.00415
899	PE	PV	30 μM	299	328	KPAPPQGSRPESTVSFNPyEPTGGPWGPDR	y19: Phospho (+79.97)	99.70%	3.6	21.5	11.6	3	1096.84	3,287.50	-0.00017
900	PE	PV	30 μM	299	328	KPAPPqGSRPESTVSFNPyEPTGGPWGPDR	q6: Deamidated (+0.98), y19: Phospho (+79.97)	99.70%	3.45	22.5	10.9	4	823.13	3,288.49	0.00621
901	PE	PV	30 μM	299	328	KPAPPqGSRPESTVSFNPyEPTGGPWGPDR	q6: Deamidated (+0.98), y19: Phospho (+79.97)	99.70%	4.29	17.7	7.7	3	1097.17	3,288.47	-0.01446
902	PE	PV	30 μM	299	328	KPAPPqGSRPESTVSFNPyEPTGGPWGPDR	q6: Deamidated (+0.98), y19: Phospho (+79.97)	99.70%	3.91	24.1	12	3	1097.17	3,288.49	0.00385
903	PE	PV	30 μM	299	328	KPAPPqGSRPESTVSFNPyEPTGGPWGPDR	q6: Deamidated (+0.98), y19: Phospho (+79.97)	99.70%	4.64	34.7	12.9	4	823.13	3,288.50	0.00719
904	PE	PV	30 μM	299	328	KPAPPQGSRPESTVSFNPyEPTGGPWGPDR	y19: Phospho (+79.97)	99.70%	6.57	34.9	12.4	4	822.88	3,287.51	0.00439
905	PE	PV	30 μM	299	328	KPAPPqGSRPESTVSFNPyEPTGGPWGPDR	q6: Deamidated (+0.98), y19: Phospho (+79.97)	99.70%	4.91	15.2	11.9	3	1097.17	3,288.49	0.00641
906	PE	PV	30 μM	299	328	KPAPPQGSRPESTVSFNPyEPTGGPWGPDR	y19: Phospho (+79.97)	99.70%	5.39	33.3	13.4	4	822.88	3,287.51	0.00097
907	PE	PV	30 μM	299	328	KPAPPqGSRPESTVSFNPyEPTGGPWGPDR	q6: Deamidated (+0.98), y19: Phospho (+79.97)	99.70%	4.84	36.6	13.6	4	823.13	3,288.49	0.00280

908	PE	PV	30 µM	299	328	KPAPPQGSRPESTVSFNPyEPTGGPWGPDR	y19: Phospho (+79.97)	99.70%	4.95	28.1	9.57	3	1096.85	3,287.53	0.02949
909	PE	PV	30 µM	299	328	KPAPPqGSRPESTVSFNPyEPTGGPWGPDR	q6: Deamidated (+0.98), y19: Phospho (+79.97)	99.70%	5.61	37.6	12.3	4	823.13	3,288.50	0.01329
910	PE	PV	30 µM	299	328	KPAPPQGSRPESTVSFNPyEPTGGPWGPDR	y19: Phospho (+79.97)	99.70%	5.93	33.1	11.9	4	822.88	3,287.51	0.00561
911	PE	PV	30 µM	299	328	KPAPPQGSRPESTVSFNPyEPTGGPWGPDR	y19: Phospho (+79.97)	99.70%	5.81	22.9	10.6	3	1096.84	3,287.51	0.00642
912	PE	PV	30 µM	299	328	KPAPPqGSRPESTVSFNPyEPTGGPWGPDR	q6: Deamidated (+0.98), y19: Phospho (+79.97)	99.70%	6.15	38.2	12.3	4	823.13	3,288.49	0.00377
913	PE	PV	30 µM	299	328	KPAPPQGSRPESTVSFNPyEPTGGPWGPDR	y19: Phospho (+79.97)	99.70%	6.5	31.4	10.6	4	822.88	3,287.50	-0.00391
914	PE	PV	30 µM	299	328	KPAPPqGSRPESTVSFNPyEPTGGPWGPDR	q6: Deamidated (+0.98), y19: Phospho (+79.97)	99.70%	5.8	38.5	14.2	4	823.13	3,288.49	0.00255
915	PE	PV	30 µM	299	328	KPAPPqGSRPESTVSFNPyEPTGGPWGPDR	q6: Deamidated (+0.98), y19: Phospho (+79.97)	99.70%	5.85	38.2	13.1	4	823.13	3,288.49	0.00109
916	PE	PV	30 µM	299	328	KPAPPQGSRPESTVSFNPyEPTGGPWGPDR	y19: Phospho (+79.97)	99.70%	5.58	30.7	10.6	3	1096.85	3,287.53	0.02290
917	PE	PV	30 µM	299	328	KPAPPQGSRPESTVSFNPyEPTGGPWGPDR	y19: Phospho (+79.97)	99.70%	6.74	32	11.4	3	1096.84	3,287.51	0.00825
918	PE	PV	30 µM	299	328	KPAPPQGSRPESTVSFNPyEPTGGPWGPDR	y19: Phospho (+79.97)	99.70%	5.8	19.9	13.7	3	1096.85	3,287.52	0.01301
919	PE	PV	30 µM	299	328	KPAPPqGSRPESTVSFNPyEPTGGPWGPDR	q6: Deamidated (+0.98), y19: Phospho (+79.97)	99.70%	6.31	19.7	10.8	3	1097.17	3,288.49	0.00568
920	PE	PV	30 µM	299	328	KPAPPQGSRPESTVSFNPyEPTGGPWGPDR	y19: Phospho (+79.97)	99.70%	2.56	20.3	6.34	3	1096.85	3,287.51	0.01081
921	PE	PV	30 µM	299	328	KPAPPQGSRPESTVSFNPyEPTGGPWGPDR	y19: Phospho (+79.97)	99.70%	3.7	28.3	14	3	1096.85	3,287.52	0.01118
922	PE	PV	30 µM	299	328	KPAPPQGSRPESTVSFNPyEPTGGPWGPDR	y19: Phospho (+79.97)	99.70%	4.81	35.7	15.3	4	822.89	3,287.52	0.01342
923	PE	PV	30 µM	299	328	KPAPPQGSRPESTVSFNPyEPTGGPWGPDR	y19: Phospho (+79.97)	99.70%	4.27	49.1	14.6	4	822.89	3,287.51	0.01049
924	PE	PV	30 µM	299	328	KPAPPQGSRPESTVsFNPYEPTGGPWGPDR	s15: Phospho (+79.97)	93.00%	0	0	3.02	4	823.15	3,288.57	1.06900
925	PE	PV	30 µM	299	328	KPAPPqGSRPESTVSFNPyEPTGGPWGPDR	q6: Deamidated (+0.98), y19: Phospho (+79.97)	99.70%	0	0	7.54	4	823.40	3,289.56	1.06700
926	PE	PV	30 µM	299	328	KPAPPQGSRPESTVSFNPyEPTGGPWGPDR	y19: Phospho (+79.97)	99.70%	3.79	12.6	4.23	4	822.90	3,287.56	0.05273
927	PE	PV	30 µM	299	328	KPAPPQGSRPESTVSFNPyEPTGGPWGPDR	y19: Phospho (+79.97)	99.70%	3.84	11.1	5.05	4	822.90	3,287.56	0.05273
928	PE	PV	30 µM	299	328	KPAPPQGSRPESTVSFNPyEPTGGPWGPDR	y19: Phospho (+79.97)	99.70%	3.74	18	6.57	4	822.90	3,287.57	0.06176
929	PE	PV	30 µM	299	328	KPAPPQGSRPESTVSFNPyEPTGGPWGPDR	y19: Phospho (+79.97)	99.70%	3.49	14	6.01	4	822.90	3,287.55	0.04785
930	PE	PV	30 µM	299	328	KPAPPQGSRPESTVSFNPyEPTGGPWGPDR	y19: Phospho (+79.97)	99.70%	3.7	14.6	6.19	4	822.90	3,287.55	0.04760
931	PE	PV	30 µM	299	328	KPAPPQGSRPESTVSFNPyEPTGGPWGPDR	y19: Phospho (+79.97)	99.70%	2.64	23.5	6.37	4	822.90	3,287.56	0.05224
932	PE	PV	30 µM	299	328	KPAPPQGSRPESTVSFNPyEPTGGPWGPDR	y19: Phospho (+79.97)	99.70%	2.43	20	6.96	3	1096.86	3,287.56	0.05293
933	PE	PV	30 µM	299	328	KPAPPQGsRPESTVSFNPYEPTGGPWGPDR	s8: Phospho (+79.97)	99.70%	2.76	13.1	7.92	3	1096.86	3,287.56	0.05805
934	PE	PV	30 µM	299	328	KPAPPQGSRPESTVSFNPyEPTGGPWGPDR	y19: Phospho (+79.97)	99.70%	4.25	25.9	8.66	4	822.90	3,287.57	0.06225
935	PE	PV	30 µM	299	328	KPAPPqGSRPESTVSFNPyEPTGGPWGPDR	q6: Deamidated (+0.98), y19: Phospho (+79.97)	99.70%	3.18	16	9.04	3	1097.19	3,288.55	0.05805
936	PE	PV	30 µM	299	328	KPAPPQGSRPESTVSFNPyEPTGGPWGPDR	y19: Phospho (+79.97)	99.70%	3.86	26	8.89	4	822.90	3,287.57	0.06225
937	PE	PV	30 µM	299	328	KPAPPQGSRPESTVSFNPyEPTGGPWGPDR	y19: Phospho (+79.97)	99.70%	3.83	26	9.1	4	822.90	3,287.56	0.05835
938	PE	PV	30 µM	299	328	KPAPPQGSRPESTVSFNPyEPTGGPWGPDR	y19: Phospho (+79.97)	99.70%	3.85	12.3	9.16	3	1096.86	3,287.56	0.05696
939	PE	PV	30 µM	299	328	KPAPPQGSRPESTVSFNPyEPTGGPWGPDR	y19: Phospho (+79.97)	99.70%	3.1	16.8	9.39	4	822.90	3,287.55	0.05053
940	PE	PV	30 µM	299	328	KPAPPQGSRPESTVSFnPyEPTGGPWGPDR	n17: Deamidated (+0.98), y19: Phospho (+79.97)	99.70%	0	35.3	15	4	823.15	3,288.55	0.06505
941	PE	PV	30 µM	299	328	KPAPPQGSRPESTVSFNPyEPTGGPWGPDR	y19: Phospho (+79.97)	99.70%	3.37	0	9.62	3	1096.86	3,287.56	0.05329
942	PE	PV	30 µM	299	328	KPAPPQGSRPESTVSFNPyEPTGGPWGPDR	y19: Phospho (+79.97)	99.70%	3.65	27.4	12.3	3	1096.86	3,287.55	0.05036
943	PE	PV	30 µM	299	328	KPAPPQGSRPESTVSFNPyEPTGGPWGPDR	y19: Phospho (+79.97)	99.70%	3.43	29.7	10.4	4	822.89	3,287.55	0.04272
944	PE	PV	30 µM	299	328	KPAPPQGSRPESTVSFNPyEPTGGPWGPDR	y19: Phospho (+79.97)	99.70%	3.22	25.2	12.6	4	822.90	3,287.55	0.04858

945	PE	PV	30 μM	299	328	KPAPPQGSRPESTVSFNPyEPTGGPWGPDR	y19: Phospho (+79.97)	99.70%	3.72	28.2	11.6	4	822.89	3,287.55	0.04589
946	PE	PV	30 μM	299	328	KPAPPQGSRPESTVSFNPyEPTGGPWGPDR	y19: Phospho (+79.97)	99.70%	3.83	22.9	11.8	4	822.89	3,287.55	0.04589
947	PE	PV	30 μM	299	328	KPAPPQGSRPESTVSFNPyEPTGGPWGPDR	y19: Phospho (+79.97)	99.70%	3.48	22	11.3	3	1096.86	3,287.55	0.04743
948	PE	PV	30 μM	299	328	KPAPPQGSRPESTVSFNPyEPTGGPWGPDR	y19: Phospho (+79.97)	99.70%	4	25.2	12.4	4	822.89	3,287.55	0.04565
949	PE	PV	30 μM	299	328	KPAPPQGSRPESTVSFNPyEPTGGPWGPDR	y19: Phospho (+79.97)	99.70%	3.87	25.6	11.8	4	822.90	3,287.55	0.04760
950	PE	PV	30 μM	299	328	KPAPPQGSRPESTVSFNPyEPTGGPWGPDR	y19: Phospho (+79.97)	99.70%	4.13	13.4	10.3	3	1096.86	3,287.56	0.05403
951	PE	PV	30 μM	299	328	KPAPPQGSRPESTVSFNPyEPTGGPWGPDR	y19: Phospho (+79.97)	99.70%	3.91	11.6	12.7	3	1096.86	3,287.55	0.04853
952	PE	PV	30 μM	299	328	KPAPPQGSRPESTVSFNPyEPTGGPWGPDR	y19: Phospho (+79.97)	99.70%	3.93	32.6	12.4	4	822.90	3,287.55	0.04711
953	PE	PV	30 μM	299	328	KPAPPQGSRPESTVSFNPyEPTGGPWGPDR	y19: Phospho (+79.97)	99.70%	3.87	22.1	10.2	3	1096.86	3,287.55	0.04450
954	PE	PV	30 μM	299	328	KPAPPQGSRPESTVSFNPyEPTGGPWGPDR	y19: Phospho (+79.97)	99.70%	3.63	27.3	11.7	4	822.90	3,287.55	0.04907
955	PE	PV	30 μM	299	328	KPAPPQGSRPESTVsFNPYEPTGGPWGPDR	s15: Phospho (+79.97)	99.70%	4.25	13.7	10.2	3	1096.86	3,287.55	0.04780
956	PE	PV	30 μM	299	328	KPAPPQGSRPESTVSFNPyEPTGGPWGPDR	y19: Phospho (+79.97)	99.70%	4	25.8	11.9	4	822.90	3,287.55	0.04736
957	PE	PV	30 μM	299	328	KPAPPQGSRPESTVSFNPyEPTGGPWGPDR	y19: Phospho (+79.97)	99.70%	4.11	26.7	11.8	4	822.90	3,287.55	0.04785
958	PE	PV	30 μM	299	328	KPAPPQGSRPESTVSFNPyEPTGGPWGPDR	y19: Phospho (+79.97)	99.70%	4.31	15.4	11.3	3	1096.86	3,287.55	0.04743
959	PE	PV	30 μM	299	328	KPAPPQGSRPESTVSFNPyEPTGGPWGPDR	y19: Phospho (+79.97)	99.70%	4.39	17.2	11.9	3	1096.86	3,287.55	0.04780
960	PE	PV	30 μM	299	328	KPAPPQGSRPESTVSFNPyEPTGGPWGPDR	y19: Phospho (+79.97)	99.70%	2.6	25.4	5.02	4	822.88	3,287.50	-0.00196
961	PE	PV	100 μM	299	328	KPAPPQGSRPESTVSFNPyEPTGGPWGPDR	y19: Phospho (+79.97)	99.20%	2.74	30.6	0	5	658.51	3,287.52	0.01140
962	PE	PV	100 μM	299	328	KPAPPQGSRPESTVSFNPyEPTGGPWGPDR	y19: Phospho (+79.97)	99.70%	5.17	36.5	11.7	4	822.89	3,287.51	0.00683
963	PE	PV	100 μM	299	328	KPAPPQGSRPESTVSFNPyEPTGGPWGPDR	y19: Phospho (+79.97)	99.70%	4.51	39.7	11.7	4	822.88	3,287.50	0.00024
964	PE	PV	100 μM	299	328	KPAPPQGSRPESTVSFNPyEPTGGPWGPDR	y19: Phospho (+79.97)	99.70%	4.65	15.9	10.7	3	1096.85	3,287.52	0.01191
965	PE	PV	100 μM	299	328	KPAPPQGSRPESTVSFNPyEPTGGPWGPDR	y19: Phospho (+79.97)	99.70%	4.58	16.5	12	3	1096.85	3,287.52	0.01301
966	PE	PV	100 μM	299	328	KPAPPQGSRPESTVSFnPyEPTGGPWGPDR	n17: Deamidated (+0.98), y19: Phospho (+79.97)	99.70%	2.93	19.7	4.23	3	1097.18	3,288.51	0.01996
967	PE	PV	100 μM	299	328	KPAPPqGSRPESTVSFNPyEPTGGPWGPDR	q6: Deamidated (+0.98), y19: Phospho (+79.97)	99.70%	2.73	31.3	7.92	3	1097.18	3,288.51	0.02655
968	PE	PV	100 μM	299	328	KPAPPQGSRPESTVSFNPyEPTGGPWGPDR	y19: Phospho (+79.97)	99.70%	0	0	8.51	4	823.39	3,289.52	2.01400
969	PE	PV	100 μM	299	328	KPAPPQGSRPESTVSFNPyEPTGGPWGPDR	y19: Phospho (+79.97)	99.70%	3.41	24.8	8.77	3	1096.85	3,287.53	0.02107
970	PE	PV	100 μM	299	328	KPAPPQGSRPESTVSFNPyEPTGGPWGPDR	y19: Phospho (+79.97)	99.70%	5.79	17.2	9.82	4	822.89	3,287.51	0.00781
971	PE	PV	100 μM	299	328	KPAPPQGSRPESTVSFNPyEPTGGPWGPDR	y19: Phospho (+79.97)	99.70%	5.68	28	11.4	4	822.89	3,287.52	0.01538
972	PE	PV	100 μM	299	328	KPAPPQGSRPESTVSFNPyEPTGGPWGPDR	y19: Phospho (+79.97)	99.70%	7.02	30	12	4	822.89	3,287.52	0.01269
973	PE	PV	100 μM	299	328	KPAPPQGSRPESTVSFNPyEPTGGPWGPDR	y19: Phospho (+79.97)	99.70%	6.91	36.8	12.9	4	822.89	3,287.52	0.01611
974	PE	PV	100 μM	299	328	KPAPPQGSRPESTVSFNPyEPTGGPWGPDR	y19: Phospho (+79.97)	99.70%	6.23	38.5	13.2	4	822.89	3,287.52	0.01538
975	PE	PV	100 μM	299	328	KPAPPQGSRPESTVSFnPyEPTGGPWGPDR	n17: Deamidated (+0.98), y19: Phospho (+79.97)	99.70%	3.61	21.3	9.28	4	823.14	3,288.51	0.02575
976	PE	PV	100 μM	299	328	KPAPPqGSRPESTVSFNPyEPTGGPWGPDR	q6: Deamidated (+0.98), y19: Phospho (+79.97)	99.70%	3.96	24	10.9	3	1097.18	3,288.50	0.01557
977	PE	PV	100 μM	299	328	KPAPPqGSRPESTVSFNPyEPTGGPWGPDR	q6: Deamidated (+0.98), y19: Phospho (+79.97)	99.70%	5.23	40.5	11.7	4	823.13	3,288.51	0.02233
978	PE	PV	100 μM	299	328	KPAPPqGSRPESTVSFNPyEPTGGPWGPDR	q6: Deamidated (+0.98), y19: Phospho (+79.97)	99.70%	5.12	40.1	14.3	4	823.14	3,288.51	0.02355
979	PE	PV	100 μM	299	328	KPAPPQGSRPESTVSFNPyEPTGGPWGPDR	y19: Phospho (+79.97)	99.70%	4.68	10.4	7.49	3	1096.85	3,287.53	0.02546
980	PE	PV	100 μM	299	328	KPAPPqGSRPESTVSFNPyEPTGGPWGPDR	q6: Deamidated (+0.98), y19: Phospho (+79.97)	99.70%	5.37	38.9	11.7	4	823.14	3,288.51	0.02404
981	PE	PV	100 μM	299	328	KPAPPQGSRPESTVSFnPyEPTGGPWGPDR	n17: Deamidated (+0.98), y19: Phospho (+79.97)	99.70%	3.86	28.3	8.35	3	1097.18	3,288.51	0.02033

982	PE	PV	100 µM	299	328	KPAPPqGSRPESTVSFNPyEPTGGPWGPDR	q6: Deamidated (+0.98), y19: Phospho (+79.97)	99.70%	5.06	44.4	10.7	4	823.13	3,288.50	0.01500
983	PE	PV	100 µM	299	328	KPAPPQGSRPESTVSFNPyEPTGGPWGPDR	y19: Phospho (+79.97)	99.70%	6.3	36.9	13	4	822.89	3,287.52	0.01391
984	PE	PV	100 µM	299	328	KPAPPQGSRPESTVSFnPyEPTGGPWGPDR	n17: Deamidated (+0.98), y19: Phospho (+79.97)	99.70%	5.36	30.6	14.8	4	823.13	3,288.50	0.01598
985	PE	PV	100 µM	299	328	KPAPPQGSRPESTVSFNPyEPTGGPWGPDR	y19: Phospho (+79.97)	99.70%	6.41	29.5	13.6	4	822.89	3,287.51	0.00805
986	PE	PV	100 µM	299	328	KPAPPQGSRPESTVSFNPyEPTGGPWGPDR	y19: Phospho (+79.97)	99.70%	4.88	16.4	12	3	1096.85	3,287.53	0.02363
987	PE	PV	100 µM	299	328	KPAPPQGSRPESTVSFNPyEPTGGPWGPDR	y19: Phospho (+79.97)	99.70%	5.32	31.4	16.4	3	1096.85	3,287.52	0.01521
988	PE	PV	100 µM	299	328	KPAPPQGSRPESTVSFNPyEPTGGPWGPDR	y19: Phospho (+79.97)	99.70%	6.44	28.3	10.4	3	1096.85	3,287.53	0.02107
989	PE	PV	100 µM	299	328	KPAPPQGSRPESTVSFNPyEPTGGPWGPDR	y19: Phospho (+79.97)	99.70%	6.28	27.2	12.3	3	1096.85	3,287.52	0.01594
990	PE	PV	100 µM	299	328	KPAPPQGSRPESTVSFnPyEPTGGPWGPDR	n17: Deamidated (+0.98), y19: Phospho (+79.97)	99.70%	2.92	0	2.54	3	1097.18	3,288.51	0.02069
991	PE	PV	100 µM	299	328	KPAPPQGSRPESTVSFNPyEPTGGPWGPDR	y19: Phospho (+79.97)	99.70%	2.99	17.8	0	5	658.51	3,287.50	-0.00173
992	PE	PV	100 µM	299	328	KPAPPqGSRPESTVSFNPyEPTGGPWGPDR	q6: Deamidated (+0.98), y19: Phospho (+79.97)	99.70%	2.89	13.2	6.28	3	1097.17	3,288.50	0.00861
993	PE	PV	100 µM	299	328	KPAPPQGSRPESTVSFNPyEPTGGPWGPDR	y19: Phospho (+79.97)	99.70%	4.28	30.6	0	5	658.51	3,287.51	0.00285
994	PE	PV	100 µM	299	328	KPAPPQGSRPESTVSFNPyEPTGGPWGPDR	y19: Phospho (+79.97)	99.70%	5.48	29.5	9.52	4	822.89	3,287.51	0.00708
995	PE	PV	100 µM	299	328	KPAPPQGSRPESTVSFNPyEPTGGPWGPDR	y19: Phospho (+79.97)	99.70%	5.53	38.6	12.4	4	822.89	3,287.51	0.00805
996	PE	PV	100 µM	299	328	KPAPPQGSRPESTVSFNPyEPTGGPWGPDR	y19: Phospho (+79.97)	99.70%	6.14	30.5	12	4	822.88	3,287.51	0.00537
997	PE	PV	100 µM	299	328	KPAPPQGSRPESTVSFNPyEPTGGPWGPDR	y19: Phospho (+79.97)	99.70%	6.63	31.3	12.2	4	822.89	3,287.51	0.00854
998	PE	PV	100 µM	299	328	KPAPPQGSRPESTVSFnPyEPTGGPWGPDR	n17: Deamidated (+0.98), y19: Phospho (+79.97)	99.70%	4	33.8	14.6	4	823.13	3,288.51	0.02208
999	PE	PV	100 µM	299	328	KPAPPqGSRPESTVSFNPyEPTGGPWGPDR	q6: Deamidated (+0.98), y19: Phospho (+79.97)	99.70%	5.07	38.4	14.1	4	823.13	3,288.49	0.00328
1000	PE	PV	100 µM	299	328	KPAPPQGSRPESTVSFnPyEPTGGPWGPDR	n17: Deamidated (+0.98), y19: Phospho (+79.97)	99.70%	5.24	24.9	11.5	4	823.13	3,288.50	0.01647
1001	PE	PV	100 µM	299	328	KPAPPqGSRPESTVSFNPyEPTGGPWGPDR	q6: Deamidated (+0.98), y19: Phospho (+79.97)	99.70%	5.25	35.8	14.9	4	823.13	3,288.49	0.00255
1002	PE	PV	100 µM	299	328	KPAPPqGSRPESTVSFNPyEPTGGPWGPDR	q6: Deamidated (+0.98), y19: Phospho (+79.97)	99.70%	4.39	46.3	13.9	4	823.13	3,288.50	0.00719
1003	PE	PV	100 µM	299	328	KPAPPQGSRPESTVSFNPyEPTGGPWGPDR	y19: Phospho (+79.97)	99.70%	5	29.2	14.8	3	1096.84	3,287.50	0.00019
1004	PE	PV	100 µM	299	328	KPAPPqGSRPESTVSFNPyEPTGGPWGPDR	q6: Deamidated (+0.98), y19: Phospho (+79.97)	99.70%	5.73	36	12.1	4	823.13	3,288.49	0.00084
1005	PE	PV	100 µM	299	328	KPAPPQGSRPESTVSFNPyEPTGGPWGPDR	y19: Phospho (+79.97)	99.70%	4.68	27.3	8.92	3	1096.85	3,287.53	0.02290
1006	PE	PV	100 µM	299	328	KPAPPqGSRPESTVSFNPyEPTGGPWGPDR	q6: Deamidated (+0.98), y19: Phospho (+79.97)	99.70%	5.06	23.4	12.9	3	1097.17	3,288.50	0.01483
1007	PE	PV	100 µM	299	328	KPAPPqGSRPESTVSFNPyEPTGGPWGPDR	q6: Deamidated (+0.98), y19: Phospho (+79.97)	99.70%	5.47	25.6	11.4	3	1097.18	3,288.51	0.02216
1008	PE	PV	100 µM	299	328	KPAPPqGSRPESTVSFNPyEPTGGPWGPDR	q6: Deamidated (+0.98), y19: Phospho (+79.97)	99.70%	5.61	29	11.3	3	1097.18	3,288.51	0.02252
1009	PE	PV	100 µM	299	328	KPAPPQGSRPESTVSFNPyEPTGGPWGPDR	y19: Phospho (+79.97)	99.70%	6.27	15.4	11.6	3	1096.85	3,287.52	0.01850
1010	PE	PV	100 µM	299	328	KPAPPQGSRPESTVSFNPyEPTGGPWGPDR	y19: Phospho (+79.97)	99.70%	6	22.5	11.6	4	822.88	3,287.50	-0.00245
1011	PE	PV	100 µM	299	328	KPAPPQGSRPESTVSFNPyEPTGGPWGPDR	y19: Phospho (+79.97)	99.70%	6.25	28.8	10	4	822.88	3,287.50	-0.00147
1012	PE	PV	100 µM	299	328	KPAPPQGSRPESTVSFNPyEPTGGPWGPDR	y19: Phospho (+79.97)	99.70%	6.1	24.4	12.8	3	1096.85	3,287.51	0.01081
1013	PE	PV	100 µM	299	328	KPAPPQGSRPESTVSFNPyEPTGGPWGPDR	y19: Phospho (+79.97)	99.70%	6.81	36.7	15.1	4	822.88	3,287.51	0.00390

1014	PE	PV	100 µM	299	328	KPAPPQGSRPESTVSFNPyEPTGGPWGPDR	y19: Phospho (+79.97)	99.70%	5.97	19.6	16.2	3	1096.84	3,287.51	0.00605
1015	PE	PV	100 µM	299	328	KPAPPQGSRPESTVSFNPyEPTGGPWGPDR	y19: Phospho (+79.97)	99.70%	6.19	25.1	12.2	3	1096.85	3,287.53	0.02473
1016	PE	PV	100 µM	299	328	KPAPPQGSRPESTVSFNPyEPTGGPWGPDR	y19: Phospho (+79.97)	99.70%	6.18	28.6	12.6	3	1096.85	3,287.53	0.02509
1017	PE	PV	100 µM	299	328	KPAPPqGSRPESTVSFNPyEPTGGPWGPDR	q6: Deamidated (+0.98), y19: Phospho (+79.97)	99.70%	5.85	36	14.4	3	1097.18	3,288.51	0.01703
1018	PE	PV	100 µM	299	328	KPAPPQGSRPESTVSFNPyEPTGGPWGPDR	y19: Phospho (+79.97)	99.70%	4.73	34.4	15.6	4	822.89	3,287.51	0.00854
1019	PE	PV	100 µM	299	328	KPAPPQGSRPESTVSFNPyEPTGGPWGPDR	y19: Phospho (+79.97)	99.70%	5.34	36	16.6	3	1096.85	3,287.51	0.00935
1020	PE	PV	100 µM	299	328	KPAPPqGSRPESTVSFNPyEPTGGPWGPDR	q6: Deamidated (+0.98), y19: Phospho (+79.97)	98.00%	0	0	4.8	3	1097.53	3,289.56	1.06900
1021	PE	PV	100 µM	299	328	KPAPPQGSRPESTVSFNPyEPTGGPWGPDR	y19: Phospho (+79.97)	99.70%	4.08	14	6.04	4	822.89	3,287.55	0.04589
1022	PE	PV	100 µM	299	328	KPAPPQGSRPESTVSFNPyEPTGGPWGPDR	y19: Phospho (+79.97)	99.70%	3.98	14.1	6.3	4	822.90	3,287.55	0.04785
1023	PE	PV	100 µM	299	328	KPAPPQGSRPESTVSFNPyEPTGGPWGPDR	y19: Phospho (+79.97)	99.70%	4.31	11.7	7.74	3	1096.86	3,287.56	0.05146
1024	PE	PV	100 µM	299	328	KPAPPQGSRPESTVSFNPyEPTGGPWGPDR	y19: Phospho (+79.97)	99.70%	3.38	22.1	8.57	4	822.90	3,287.56	0.05981
1025	PE	PV	100 µM	299	328	KPAPPQGSRPESTVSFNPyEPTGGPWGPDR	y19: Phospho (+79.97)	99.70%	3.14	10.5	8.54	3	1096.86	3,287.56	0.05146
1026	PE	PV	100 µM	299	328	KPAPPQGSRPESTVSFNPyEPTGGPWGPDR	y19: Phospho (+79.97)	99.70%	4.39	25.1	8.48	3	1096.86	3,287.56	0.05146
1027	PE	PV	100 µM	299	328	KPAPPQGSRPESTVSFNPyEPTGGPWGPDR	y19: Phospho (+79.97)	99.70%	4.01	22.1	9.74	4	822.90	3,287.57	0.06372
1028	PE	PV	100 µM	299	328	KPAPPQGSRPESTVSFNPyEPTGGPWGPDR	y19: Phospho (+79.97)	99.70%	3.82	21.7	9.08	4	822.89	3,287.55	0.04516
1029	PE	PV	100 µM	299	328	KPAPPQGSRPESTVSFNPyEPTGGPWGPDR	y19: Phospho (+79.97)	99.70%	3.71	26	9.8	4	822.90	3,287.55	0.04711
1030	PE	PV	100 µM	299	328	KPAPPQGSRPESTVSFNPyEPTGGPWGPDR	y19: Phospho (+79.97)	99.70%	4.2	20	9.1	3	1096.86	3,287.55	0.04487
1031	PE	PV	100 µM	299	328	KPAPPQGSRPESTVSFNPyEPTGGPWGPDR	y19: Phospho (+79.97)	99.70%	3.49	16.3	11.1	3	1096.86	3,287.56	0.05732
1032	PE	PV	100 µM	299	328	KPAPPQGSRPESTVSFNPyEPTGGPWGPDR	y19: Phospho (+79.97)	99.70%	3.73	14.3	11.3	3	1096.86	3,287.55	0.04304
1033	PE	PV	100 µM	299	328	KPAPPQGSRPEsTVSFnPYEPTGGPWGPDR	s12: Phospho (+79.97), n17: Deamidated (+0.98)	99.70%	3.42	16.8	12.3	3	1097.19	3,288.54	0.05255
1034	PE	PV	100 µM	299	328	KPAPPQGSRPESTVSFNPyEPTGGPWGPDR	y19: Phospho (+79.97)	99.70%	3.59	17.3	12.5	3	1096.86	3,287.54	0.04084
1035	PE	PV	100 µM	299	328	KPAPPQGSRPESTVSFNPyEPTGGPWGPDR	y19: Phospho (+79.97)	99.70%	4.02	33.4	14	4	822.89	3,287.55	0.04296
1036	PE	PV	100 µM	299	328	KPAPPQGSRPESTVSFNPyEPTGGPWGPDR	y19: Phospho (+79.97)	99.70%	3.57	22.3	10.7	4	822.89	3,287.55	0.04174
1037	PE	PV	100 µM	299	328	KPAPPQGSRPESTVSFNPyEPTGGPWGPDR	y19: Phospho (+79.97)	99.70%	4.36	30.1	13.6	4	822.90	3,287.55	0.04711
1038	PE	PV	100 µM	299	328	KPAPPQGSRPESTVSFNPyEPTGGPWGPDR	y19: Phospho (+79.97)	99.70%	3.6	15.3	11	3	1096.86	3,287.55	0.04487
1039	PE	PV	100 µM	299	328	KPAPPQGSRPESTVSFNPyEPTGGPWGPDR	y19: Phospho (+79.97)	99.70%	4.02	26.9	13	4	822.89	3,287.55	0.04272
1040	PE	PV	100 µM	299	328	KPAPPQGSRPESTVSFNPyEPTGGPWGPDR	y19: Phospho (+79.97)	99.70%	3.88	24.8	12.4	4	822.89	3,287.55	0.04370
1041	PE	PV	100 µM	299	328	KPAPPQGSRPESTVSFNPyEPTGGPWGPDR	y19: Phospho (+79.97)	99.70%	3.82	18.9	12.9	3	1096.86	3,287.55	0.04597
1042	PE	PV	100 µM	299	328	KPAPPQGSRPESTVSFNPyEPTGGPWGPDR	y19: Phospho (+79.97)	99.70%	3.59	18.7	9.8	3	1096.86	3,287.55	0.04341
1043	PE	PV	100 µM	299	328	KPAPPQGSRPESTVSFNPyEPTGGPWGPDR	y19: Phospho (+79.97)	99.70%	4.15	23.1	11.6	3	1096.86	3,287.55	0.04890
1044	PE	PV	100 µM	299	328	KPAPPQGSRPESTVSFNPyEPTGGPWGPDR	y19: Phospho (+79.97)	99.70%	3.95	36	12	4	822.89	3,287.55	0.04345
1045	PE	PV	100 µM	299	328	KPAPPQGSRPESTVSFNPyEPTGGPWGPDR	y19: Phospho (+79.97)	99.70%	3.97	24.1	11.8	4	822.89	3,287.55	0.04565
1046	PE	PV	100 µM	299	328	KPAPPQGSRPESTVSFNPyEPTGGPWGPDR	y19: Phospho (+79.97)	99.70%	3.88	29.8	12.2	4	822.89	3,287.55	0.04126
1047	PE	PV	100 µM	299	328	KPAPPQGSRPESTVSFNPyEPTGGPWGPDR	y19: Phospho (+79.97)	99.70%	4.07	25.5	11.7	4	822.89	3,287.55	0.04199
1048	PE	PV	100 µM	299	328	KPAPPQGSRPESTVSFNPyEPTGGPWGPDR	y19: Phospho (+79.97)	99.70%	4.12	29	12.7	4	822.89	3,287.55	0.04223
1049	PE	PV	100 µM	299	328	KPAPPQGSRPESTVSFNPyEPTGGPWGPDR	y19: Phospho (+79.97)	99.70%	4.07	19	10.1	3	1096.86	3,287.54	0.03938
1050	PE	PV	100 µM	299	328	KPAPPQGSRPESTVSFNPyEPTGGPWGPDR	y19: Phospho (+79.97)	99.70%	3.91	25	13.5	4	822.89	3,287.54	0.04052
1051	PE	PV	100 µM	299	328	KPAPPQGSRPESTVSFNPyEPTGGPWGPDR	y19: Phospho (+79.97)	99.70%	4.31	17.2	12.1	3	1096.86	3,287.55	0.04377
1052	PE	PV	100 µM	299	328	KPAPPqGsRPESTVSFNPYEPTGGPWGPDR	q6: Deamidated (+0.98), s8: Phospho (+79.97)	99.50%	1.15	10.1	1.55	4	823.14	3,288.51	0.02526
1053	PE	PV	100 µM	299	328	KPAPPQGSRPESTVSFNPyEPTGGPWGPDR	y19: Phospho (+79.97)	99.70%	1.35	16	0.96	3	1096.84	3,287.50	-0.00457
1054	PE	PV	100 µM	299	328	KPAPPQGSRPESTVSFNPyEPTGGPWGPDR	y19: Phospho (+79.97)	99.70%	2.14	0	2.85	4	822.88	3,287.49	-0.01246

1055	PE	PV	100 μM	299	328	KPAPPQGSRPESTVSFNPyEPTGGPWGPDR	y19: Phospho (+79.97)	99.70%	4.27	24.5	6.42	4	822.89	3,287.51	0.00708
1056	PE	PV	100 μM	299	328	KPAPPQGSRPESTVSFNPyEPTGGPWGPDR	y19: Phospho (+79.97)	99.70%	4.42	21.1	8.82	4	822.88	3,287.50	0.00048
1057	IP	NT	0	299	332	KPAPPQGSRPESTVSFNPyEPTGGPWGPDRGLQR	y19: Phospho (+79.97)	99.70%	5.55	40.2	3.36	4	936.45	3,741.77	0.00105
1058	IP	NT	0	299	332	KPAPPQGSRPESTVSFNPyEPTGGPWGPDRGLQR	y19: Phospho (+79.97)	99.70%	5.06	21.6	2.48	4	936.45	3,741.77	0.00520
1059	IP	NT	0	299	332	KPAPPQGSRPESTVSFnPyEPTGGPWGPDRGLQR	n17: Deamidated (+0.98), y19: Phospho (+79.97)	99.70%	5.96	41.2	2.62	4	936.70	3,742.77	0.01826
1060	IP	NT	0	299	332	KPAPPQGSRPESTVSFNPyEPTGGPWGPDRGLQR	y19: Phospho (+79.97)	99.70%	5.02	24.7	3.57	4	936.45	3,741.77	0.00350
1061	IP	Hg	50 μM	299	332	KPAPPQGSRPESTVSFNPyEPTGGPWGPDRGLQR	y19: Phospho (+79.97)	98.70%	3.58	17.4	1.92	4	936.45	3,741.79	0.01692
1062	IP	Hg	50 μM	299	332	KPAPPQGSRPESTVSFNPyEPTGGPWGPDRGLqR	y19: Phospho (+79.97), q33: Deamidated (+0.98)	99.60%	0	0	2.57	4	937.20	3,744.78	2.02600
1063	IP	Hg	50 μM	299	332	KPAPPQGSRPESTVSFNPyEPTGGPWGPDRGLQR	y19: Phospho (+79.97)	99.70%	4.49	15.5	4.21	4	936.45	3,741.78	0.00569
1064	IP	Hg	50 μM	299	332	KPAPPQGSRPESTVSFNPyEPTGGPWGPDRGLQR	y19: Phospho (+79.97)	99.70%	5.44	33	2.41	4	936.45	3,741.77	0.00179
1065	IP	Hg	50 μM	299	332	KPAPPQGSRPESTVSFNPyEPTGGPWGPDRGLQR	y19: Phospho (+79.97)	99.70%	5.25	28.9	3.08	4	936.45	3,741.78	0.00765
1066	IP	Hg	50 μM	299	332	KPAPPQGSRPESTVSFNPyEPTGGPWGPDRGLQR	y19: Phospho (+79.97)	99.70%	4.94	26.6	2.89	4	936.45	3,741.77	0.00276
1067	IP	PV	0	299	332	KPAPPQGSRPESTVSFNPYEPtGGPWGPDRGLQR	t22: Phospho (+79.97)	99.20%	5.57	28.6	1.85	4	936.45	3,741.77	0.00374
1068	IP	PV	0	299	332	KPAPPQGSRPESTVSFNPyEPTGGPWGPDRGLQR	y19: Phospho (+79.97)	99.60%	4.75	21.7	1.96	4	936.45	3,741.78	0.00569
1069	IP	PV	0	299	332	KPAPPqGSRPESTVSFnPyEPTGGPWGPDRGLQR	q6: Deamidated (+0.98), n17: Deamidated (+0.98), y19: Phospho (+79.97)	99.70%	4.48	37	2.01	4	936.95	3,743.77	0.02985
1070	IP	PV	0	299	332	KPAPPQGSRPESTVSFNPyEPTGGPWGPDRGLQR	y19: Phospho (+79.97)	99.70%	5.6	26.2	3.31	4	936.45	3,741.77	0.00301
1071	PE	Ig	2 min	299	332	KPAPPQGSRPESTVSFNPyEPTGGPWGPDRGLQR	y19: Phospho (+79.97)	99.60%	3.84	11.6	2.28	4	936.45	3,741.76	-0.00920
1072	PE	Ig	2 min	299	332	KPAPPQGSRPESTVSFNPyEPTGGPWGPDRGLQR	y19: Phospho (+79.97)	99.70%	3.91	30.4	3.01	4	936.45	3,741.76	-0.01164
1073	IP	PV	0	333	355	EALPmDTEVYESPyADPEEIRPK	m5: Oxidation (+15.99), y14: Phospho (+79.97)	99.00%	3.82	45.7	3.52	3	925.74	2,774.21	0.00578
1074	IP	PV	0	333	355	EALPmDTEVyESPYADPEEIRPK	m5: Oxidation (+15.99), y10: Phospho (+79.97)	99.30%	3.81	25.6	1.85	3	925.74	2,774.21	0.00395
1075	IP	PV	0	333	355	EALPmDTEVYESPyADPEEIRPK	m5: Oxidation (+15.99), y14: Phospho (+79.97)	99.70%	3.93	36.9	3.72	3	925.74	2,774.21	0.00193
1076	IP	PV	0	333	355	EALPmDTEVYESPyADPEEIRPK	m5: Oxidation (+15.99), y14: Phospho (+79.97)	99.70%	0	0	4.74	3	926.08	2,775.21	1.01000
1077	IP	PV	0	333	355	EALPmDTEVYESPyADPEEIRPK	m5: Oxidation (+15.99), y14: Phospho (+79.97)	99.70%	3.32	7.43	5.25	3	925.74	2,774.21	0.00358
1078	IP	PV	0	333	355	EALPmDTEVyESPYADPEEIRPK	m5: Oxidation (+15.99), y10: Phospho (+79.97)	99.70%	4.01	9.85	4.15	3	925.74	2,774.21	0.00230
1079	IP	PV	0	333	355	EALPmDTEVyESPYADPEEIRPK	m5: Oxidation (+15.99), y10: Phospho (+79.97)	99.70%	4.27	37.8	5.1	3	925.74	2,774.21	0.00431
1080	PE	Ig	NT	333	355	EALPMDTEVYESPyADPEEIRPK	y14: Phospho (+79.97)	99.70%	2.6	37.5	11.5	3	920.43	2,758.26	0.05074
1081	PE	Ig	1 min	333	355	EALPMDTEVYESPyADPEEIRPK	y14: Phospho (+79.97)	97.40%	0.49	12.3	0	3	920.41	2,758.22	0.01101
1082	PE	Ig	1 min	333	355	EALPMDTEVYESPyADPEEIRPK	y14: Phospho (+79.97)	99.70%	2.81	36.8	9	3	920.43	2,758.25	0.04561
1083	PE	Ig	1 min	333	355	EALPMDTEVYESPyADPEEIRPK	y14: Phospho (+79.97)	99.70%	2.61	26.7	9.82	3	920.43	2,758.26	0.05001
1084	PE	Ig	1 min	333	355	EALPMDTEVYESPyADPEEIRPK	y14: Phospho (+79.97)	99.70%	3.41	43.1	13.3	3	920.42	2,758.25	0.04177
1085	PE	Ig	2 min	333	355	EALPMDTEVYESPyADPEEIRPK	y14: Phospho (+79.97)	99.70%	2.28	20.2	5.21	3	920.42	2,758.23	0.02456
1086	PE	Ig	2 min	333	355	EALPmDTEVYESPyADPEEIRPK	m5: Oxidation (+15.99), y14: Phospho (+79.97)	99.70%	1.53	17.2	0	3	925.75	2,774.22	0.01402
1087	PE	Ig	2 min	333	355	EALPMDTEVYESPyADPEEIRPK	y14: Phospho (+79.97)	99.70%	2.58	47.7	0	3	920.41	2,758.22	0.00973

1088	PE	Ig	2 min	333	355	EALPMDTEVyESPYADPEEIRPK	y10: Phospho (+79.97)	99.70%	1.78	30	0	3	920.41	2,758.21	0.00606
1089	PE	Ig	2 min	333	355	EALPMDTEVYESPyADPEEIRPK	y14: Phospho (+79.97)	99.70%	2.57	39.4	9.02	3	920.43	2,758.25	0.04525
1090	PE	Ig	2 min	333	355	EALPMDTEVYESPyADPEEIRPK	y14: Phospho (+79.97)	99.70%	3.02	37.5	9.7	3	920.43	2,758.26	0.04873
1091	PE	Ig	3 min	333	355	EALPMDTEVyESPYADPEEIRPK	y10: Phospho (+79.97)	99.50%	0.74	12	0	3	920.41	2,758.22	0.00918
1092	PE	Ig	3 min	333	355	EALPmDTEVYESPyADPEEIRPK	m5: Oxidation (+15.99), y14: Phospho (+79.97)	99.70%	1.75	29.2	0	3	925.74	2,774.21	0.00779
1093	PE	Ig	3 min	333	355	EALPMDTEVYESPyADPEEIRPK	y14: Phospho (+79.97)	99.70%	0	0	8.66	3	921.09	2,760.25	2.04000
1094	PE	Ig	3 min	333	355	EALPMDTEVYESPyADPEEIRPK	y14: Phospho (+79.97)	99.70%	2.74	19.1	10.2	3	920.42	2,758.24	0.03206
1095	PE	Ig	3 min	333	355	EALPMDTEVYESPyADPEEIRPK	y14: Phospho (+79.97)	99.70%	3.06	16.1	12.7	3	920.42	2,758.24	0.03316
1096	PE	Ig	5 min	333	355	EALPMDTEVYESPyADPEEIRPK	y14: Phospho (+79.97)	99.70%	2.87	21.9	8.24	3	920.41	2,758.21	0.00075
1097	PE	Ig	5 min	333	355	EALPMDTEVYEsPYADPEEIRPK	s12: Phospho (+79.97)	86.30%	0.67	0	0	3	920.41	2,758.22	0.00844
1098	PE	Ig	5 min	333	355	EALPMDtEVYESPYADPEEIRPK	t7: Phospho (+79.97)	99.70%	0.38	21.1	0	2	1380.12	2,758.22	0.00956
1099	PE	Ig	5 min	333	355	EALPmDTEVYEsPYADPEEIRPK	m5: Oxidation (+15.99), s12: Phospho (+79.97)	99.70%	1.24	37.4	0	3	925.75	2,774.21	0.01072
1100	PE	Ig	5 min	333	355	EALPMDTEVYESPyADPEEIRPK	y14: Phospho (+79.97)	99.70%	2.4	37.5	0	3	920.42	2,758.23	0.01650
1101	PE	Ig	5 min	333	355	EALPMDTEVyESPYADPEEIRPK	y10: Phospho (+79.97)	99.70%	2.54	57.9	0	3	920.41	2,758.21	0.00533
1102	PE	Ig	5 min	333	355	EALPmDTEVYESPyADPEEIRPK	m5: Oxidation (+15.99), y14: Phospho (+79.97)	99.70%	2.87	42.3	0	3	925.74	2,774.21	0.00541
1103	PE	Ig	15 min	333	355	EALPMDTEVYESPyADPEEIRPK	y14: Phospho (+79.97)	99.10%	1.18	17.6	0	3	920.41	2,758.22	0.01064
1104	PE	Ig	15 min	333	355	EALPmDTEVYESPyADPEEIRPK	m5: Oxidation (+15.99), y14: Phospho (+79.97)	99.70%	2.4	51	0	3	925.74	2,774.21	0.00541
1105	PE	Ig	15 min	333	355	EALPMDTEVYESPyADPEEIRPK	y14: Phospho (+79.97)	99.70%	2.8	17.7	8.28	3	920.43	2,758.26	0.04799
1106	PE	Hg	0	333	355	EALPmDTEVYESPyADPEEIRPK	m5: Oxidation (+15.99), y14: Phospho (+79.97)	98.40%	2.68	0	0.7	3	925.74	2,774.21	0.00816
1107	PE	Hg	0	333	355	EALPMDTEVYESPyADPEEIRPK	y14: Phospho (+79.97)	99.70%	0	40.4	3	3	920.41	2,758.21	0.00149
1108	PE	Hg	0	333	355	EALPMDTEVyESPYADPEEIRPK	y10: Phospho (+79.97)	99.70%	1.07	16.5	2.22	3	920.41	2,758.21	0.00295
1109	PE	Hg	0	333	355	EALPmDTEVYESPyADPEEIRPK	m5: Oxidation (+15.99), y14: Phospho (+79.97)	99.70%	1.4	12.8	4.96	3	925.75	2,774.22	0.01402
1110	PE	Hg	0	333	355	EALPmDTEVYESPyADPEEIRPK	m5: Oxidation (+15.99), y14: Phospho (+79.97)	99.70%	1.58	17	4.89	3	925.74	2,774.21	0.00889
1111	PE	Hg	0	333	355	EALPMDTEVYESPyADPEEIRPK	y14: Phospho (+79.97)	99.70%	0	19.6	5.92	3	920.42	2,758.23	0.01742
1112	PE	Hg	1 µM	333	355	EALPmDTEVYESPyADPEEIRPK	m5: Oxidation (+15.99), y14: Phospho (+79.97)	99.00%	0.99	11.1	0	3	925.74	2,774.20	-0.00851
1113	PE	Hg	1 µM	333	355	EALPMDTEVYESPyADPEEIRPK	y14: Phospho (+79.97)	99.60%	1.99	11.7	2.34	3	920.43	2,758.26	0.04799
1114	PE	Hg	1 µM	333	355	EALPMDTEVYESPyADPEEIRPK	y14: Phospho (+79.97)	99.70%	2.53	29.5	7.74	3	920.42	2,758.24	0.03609
1115	PE	Hg	5 µM	333	355	EALPmDTEVYESPyADPEEIRPK	m5: Oxidation (+15.99), y14: Phospho (+79.97)	88.80%	0.65	0	0.85	3	925.74	2,774.21	0.00834
1116	PE	Hg	10 µM	333	355	EALPmDTEVYEsPYADPEEIRPK	m5: Oxidation (+15.99), s12: Phospho (+79.97)	99.70%	1.09	0	2.35	3	925.74	2,774.21	0.00688
1117	PE	Hg	10 µM	333	355	EALPmDTEVYESPyADPEEIRPK	m5: Oxidation (+15.99), y14: Phospho (+79.97)	99.70%	1.44	26.5	5.44	3	925.74	2,774.20	-0.00118
1118	PE	Hg	10 µM	333	355	EALPmDTEVYESPyADPEEIRPK	m5: Oxidation (+15.99), y14: Phospho (+79.97)	99.70%	0	19.9	1.33	3	925.75	2,774.22	0.01383
1119	PE	Hg	20 µM	333	355	EALPMDTEVyESPYADPEEIRPK	y10: Phospho (+79.97)	96.10%	0.74	0	0	3	920.41	2,758.20	-0.00694
1120	PE	Hg	20 µM	333	355	EALPMDTEVYESPyADPEEIRPK	y14: Phospho (+79.97)	99.70%	1.31	0	2.19	3	920.41	2,758.22	0.00808

1121	PE	Hg	20 µM	333	355	EALPmDTEVYESPyADPEEIRPK	m5: Oxidation (+15.99), y14: Phospho (+79.97)	99.70%	1.22	10.3	3.18	3	925.75	2,774.21	0.01109
1122	PE	Hg	50 µM	333	355	EALPMDTEVYESPyADPEEIRPK	y14: Phospho (+79.97)	99.70%	2.76	33.9	8.07	3	920.42	2,758.23	0.01815
1123	PE	Hg	50 µM	333	355	EALPMDTEVYESPyADPEEIRPK	y14: Phospho (+79.97)	99.70%	3.37	34.2	9.3	3	920.41	2,758.22	0.01430
1124	PE	Hg	50 µM	333	355	EALPmDTEVYESPyADPEEIRPK	m5: Oxidation (+15.99), y14: Phospho (+79.97)	99.70%	1.43	11.3	2.28	4	694.56	2,774.19	-0.01041
1125	PE	Hg	50 µM	333	355	EALPmDTEVYESPyADPEEIRPK	m5: Oxidation (+15.99), y14: Phospho (+79.97)	99.70%	1.43	21.3	3.15	3	925.74	2,774.20	-0.00100
1126	PE	Hg	50 µM	333	355	EALPmDTEVYESPyADPEEIRPK	m5: Oxidation (+15.99), y14: Phospho (+79.97)	99.70%	2.47	39.5	9.49	3	925.74	2,774.20	-0.00136
1127	PE	Hg	50 µM	333	355	EALPMDTEVYESPyADPEEIRPK	y14: Phospho (+79.97)	99.70%	2.62	46.2	8.29	3	920.41	2,758.20	-0.00584
1128	PE	Hg	50 µM	333	355	EALPmDTEVYESPyADPEEIRPK	m5: Oxidation (+15.99), y14: Phospho (+79.97)	99.70%	1.16	10.9	2.96	3	925.74	2,774.21	0.00834
1129	PE	Hg	50 µM	333	355	EALPMDTEVYESPyADPEEIRPK	y14: Phospho (+79.97)	99.70%	2.17	49.9	5.35	3	920.41	2,758.22	0.00918
1130	PE	Hg	50 µM	333	355	EALPMDtEVYESPyADPEEIRPK	t7: Phospho (+79.97), y14: Phospho (+79.97)	99.70%	0	0	8.55	3	947.75	2,840.22	2.04100
1131	PE	Hg	50 µM	333	355	eALPmDTEVYESPyADPEEIRPK	n-term: Dehydrated (-18.01), m5: Oxidation (+15.99), y14: Phospho (+79.97)	99.70%	0	0	10.3	3	920.09	2,757.25	1.05200
1132	PE	Hg	50 µM	333	355	EALPMDTEVYESPyADPEEIRPK	y14: Phospho (+79.97)	99.70%	2.79	21.9	8.14	3	920.43	2,758.26	0.05038
1133	PE	Hg	50 µM	333	355	EALPMDTEVYESPyADPEEIRPK	y14: Phospho (+79.97)	99.70%	2.91	38.7	9.89	3	920.43	2,758.26	0.04726
1134	PE	Hg	50 µM	333	355	EALPMDTEVYESPyADPEEIRPK	y14: Phospho (+79.97)	99.70%	2.86	32.9	9.6	3	920.43	2,758.26	0.05111
1135	PE	Hg	50 µM	333	355	EALPMDTEVyESPyADPEEIRPK	y10: Phospho (+79.97), y14: Phospho (+79.97)	99.70%	3.27	41.1	9.1	3	947.08	2,838.21	0.03961
1136	PE	Hg	50 µM	333	355	EALPMDTEVyESPyADPEEIRPK	y10: Phospho (+79.97), y14: Phospho (+79.97)	99.70%	2.79	12.9	10.5	3	947.08	2,838.21	0.03686
1137	PE	Hg	50 µM	333	355	EALPMDTEVyESPyADPEEIRPK	y10: Phospho (+79.97), y14: Phospho (+79.97)	99.70%	3.38	53.3	13	3	947.07	2,838.20	0.02734
1138	PE	Hg	50 µM	333	355	EALPMDTEVYESPyADPEEIRPK	y14: Phospho (+79.97)	99.70%	2.49	36.9	5.77	3	920.41	2,758.21	0.00039
1139	PE	Hg	100 µM	333	355	EALPmDTEVYESPyADPEEIRPK	m5: Oxidation (+15.99), y14: Phospho (+79.97)	90.90%	0	0	0.68	3	926.08	2,775.21	1.00500
1140	PE	Hg	100 µM	333	355	EALPMDTEVYESPyADPEEIRPK	y14: Phospho (+79.97)	91.40%	2.66	0	0	2	1380.11	2,758.21	0.00541
1141	PE	Hg	100 µM	333	355	EALPMDTEVYESPyADPEEIRPK	y14: Phospho (+79.97)	99.70%	0	0	3.48	3	920.75	2,759.22	1.00700
1142	PE	Hg	100 µM	333	355	EALPMDTEVYESPyADPEEIRPK	y14: Phospho (+79.97)	99.70%	2.47	16.7	1.8	3	920.41	2,758.21	-0.00236
1143	PE	Hg	100 µM	333	355	EALPmDTEVYESPyADPEEIRPK	m5: Oxidation (+15.99), y14: Phospho (+79.97)	99.70%	2.19	15.6	2.82	3	925.74	2,774.19	-0.01400
1144	PE	Hg	100 µM	333	355	EALPmDTEVYESPyADPEEIRPK	m5: Oxidation (+15.99), y14: Phospho (+79.97)	99.70%	2.96	16.6	3.77	3	925.75	2,774.22	0.01621
1145	PE	Hg	100 µM	333	355	EALPmDTEVYESPyADPEEIRPK	m5: Oxidation (+15.99), y14: Phospho (+79.97)	99.70%	4.15	30.2	3.1	3	925.74	2,774.21	0.00926
1146	PE	Hg	100 µM	333	355	EALPMDTEVYESPyADPEEIRPK	y14: Phospho (+79.97)	99.70%	2.83	35.5	4.68	3	920.41	2,758.21	0.00588
1147	PE	Hg	100 µM	333	355	EALPMDTEVYESPyADPEEIRPK	y14: Phospho (+79.97)	99.70%	1.93	21	3.33	3	920.41	2,758.21	0.00094
1148	PE	Hg	100 µM	333	355	EALPMDTEVyESPyADPEEIRPK	y10: Phospho (+79.97), y14: Phospho (+79.97)	99.60%	0	28.1	7.8	3	947.06	2,838.17	-0.00763

1149	PE	Hg	100 µM	333	355	EALPMDTEVyESPyADPEEIRPK	y10: Phospho (+79.97), y14: Phospho (+79.97)	99.60%	0	18.7	8.34	3	947.06	2,838.17	-0.00250
1150	PE	Hg	100 µM	333	355	EALPMDTEVYESPyADPEEIRPK	y14: Phospho (+79.97)	99.70%	0	39.3	9.96	3	920.41	2,758.22	0.01028
1151	PE	Hg	100 µM	333	355	EALPMDTEVYESPyADPEEIRPK	y14: Phospho (+79.97)	99.70%	0	35.8	11.1	3	920.41	2,758.22	0.01394
1152	PE	Hg	100 µM	333	355	EALPMDTEVYESPyADPEEIRPK	y14: Phospho (+79.97)	99.70%	0	34.3	9.57	3	920.41	2,758.21	0.00112
1153	PE	Hg	100 µM	333	355	EALPMDTEVYESPyADPEEIRPK	y14: Phospho (+79.97)	99.70%	0	50.9	10.5	3	920.41	2,758.22	0.01430
1154	PE	Hg	100 µM	333	355	EALPMDTEVyESPYADPEEIRPK	y10: Phospho (+79.97)	99.70%	0	40.3	11.5	3	920.41	2,758.22	0.00753
1155	PE	Hg	100 µM	333	355	EALPMDTEVYESPyADPEEIRPK	y14: Phospho (+79.97)	99.70%	1.47	16.4	2.32	3	920.41	2,758.20	-0.00584
1156	PE	Hg	100 µM	333	355	EALPmDTEVYESPyADPEEIRPK	m5: Oxidation (+15.99), y14: Phospho (+79.97)	99.70%	1.49	27.9	9.08	3	925.74	2,774.20	-0.00173
1157	PE	Hg	100 µM	333	355	EALPmDTEVYESPyADPEEIRPK	m5: Oxidation (+15.99), y14: Phospho (+79.97)	99.70%	2.44	38.4	10.5	3	925.74	2,774.20	-0.00136
1158	PE	Hg	100 µM	333	355	EALPMDTEVYESPyADPEEIRPK	y14: Phospho (+79.97)	99.70%	3.82	39.6	7.44	3	920.42	2,758.23	0.01797
1159	PE	Hg	100 µM	333	355	EALPmDTEVYESPyADPEEIRPK	m5: Oxidation (+15.99), y14: Phospho (+79.97)	99.70%	1.94	39.2	7.03	3	925.75	2,774.21	0.01090
1160	PE	Hg	100 µM	333	355	EALPMDTEVYESPyADPEEIRPK	y14: Phospho (+79.97)	99.70%	2.62	46.8	5.38	3	920.41	2,758.22	0.01064
1161	PE	Hg	100 µM	333	355	EALPMDtEVYESPyADPEEIRPK	t7: Phospho (+79.97), y14: Phospho (+79.97)	99.70%	0	0	7.6	3	947.75	2,840.23	2.05100
1162	PE	Hg	100 µM	333	355	EALPMDTEVYESPyADPEEIRPK	y14: Phospho (+79.97)	99.70%	2.25	27	7.12	3	920.42	2,758.24	0.03463
1163	PE	Hg	100 µM	333	355	EALPMDTEVYESPyADPEEIRPK	y14: Phospho (+79.97)	99.70%	2.37	25.1	8.7	3	920.42	2,758.25	0.04140
1164	PE	Hg	100 µM	333	355	EALPMDTEVyESPyADPEEIRPK	y10: Phospho (+79.97), y14: Phospho (+79.97)	99.70%	2.35	21.9	8.77	3	947.08	2,838.21	0.03705
1165	PE	Hg	100 µM	333	355	EALPMDTEVYESPyADPEEIRPK	y14: Phospho (+79.97)	99.70%	2.86	37.2	11	3	920.42	2,758.25	0.04287
1166	PE	Hg	100 µM	333	355	EALPMDTEVYESPyADPEEIRPK	y14: Phospho (+79.97)	99.70%	2.69	34.3	11.6	3	920.42	2,758.25	0.03884
1167	PE	Hg	100 µM	333	355	EALPMDTEVyESPyADPEEIRPK	y10: Phospho (+79.97), y14: Phospho (+79.97)	99.70%	2.42	27.4	11.4	3	947.08	2,838.21	0.03796
1168	PE	Hg	100 µM	333	355	EALPMDTEVyESPyADPEEIRPK	y10: Phospho (+79.97), y14: Phospho (+79.97)	99.70%	2.64	22.9	11.7	3	947.08	2,838.21	0.03137
1169	PE	Hg	100 µM	333	355	EALPMDTEVYESPyADPEEIRPK	y14: Phospho (+79.97)	99.70%	1.26	18.1	4.21	3	920.41	2,758.21	-0.00035
1170	PE	PV	0	333	355	EALPMDTEVYESPyADPEEIRPK	y14: Phospho (+79.97)	99.70%	1.21	0	2.64	3	920.41	2,758.21	0.00442
1171	PE	PV	0	333	355	EALPMDTEVYESPyADPEEIRPK	y14: Phospho (+79.97)	99.70%	0	19.6	5.92	3	920.42	2,758.23	0.01742
1172	PE	PV	1 µM	333	355	EALPmDTEVYESPyADPEEIRPK	m5: Oxidation (+15.99), y14: Phospho (+79.97)	99.70%	2.61	28.5	7.96	3	925.74	2,774.20	-0.00466
1173	PE	PV	3 µM	333	355	EALPmDTEVYESPyADPEEIRPK	m5: Oxidation (+15.99), y14: Phospho (+79.97)	99.70%	0.74	0	1.29	3	925.74	2,774.20	-0.00741
1174	PE	PV	10 µM	333	355	EALPMDTEVYESPyADPEEIRPK	y14: Phospho (+79.97)	99.70%	1.67	0	1.82	3	920.41	2,758.20	-0.00456
1175	PE	PV	10 µM	333	355	EALPMDtEVYESPYADPEEIRPK	t7: Phospho (+79.97)	99.70%	2.59	19.4	6.82	3	920.42	2,758.23	0.01742
1176	PE	PV	10 µM	333	355	EALPMDTEVyESPyADPEEIRPK	y10: Phospho (+79.97), y14: Phospho (+79.97)	99.70%	3.31	27.2	8.31	3	947.06	2,838.16	-0.01221
1177	PE	PV	10 µM	333	355	EALPMDTEVYESPyADPEEIRPK	y14: Phospho (+79.97)	99.70%	4.5	32.3	7.74	3	920.41	2,758.22	0.00716
1178	PE	PV	10 µM	333	355	eALPmDtEVYESPYADPEEIRPK	n-term: Dehydrated (-18.01), m5: Oxidation (+15.99), t7: Phospho (+79.97)	99.70%	0	0	12.9	3	920.08	2,757.21	1.01400
1179	PE	PV	10 µM	333	355	EALPmDTEVYESPyADPEEIRPK	m5: Oxidation (+15.99), y14: Phospho (+79.97)	99.70%	2.83	27.6	7.16	3	925.74	2,774.21	0.00578

1180	PE	PV	10 μM	333	355	EALPMDTEVYESPyADPEEIRPK	y14: Phospho (+79.97)	99.70%	4.51	38.8	9.92	3	920.41	2,758.22	0.00790
1181	PE	PV	10 μM	333	355	EALPMDTEVYESPyADPEEIRPK	y14: Phospho (+79.97)	99.70%	3.27	22.3	8.85	3	920.41	2,758.21	-0.00218
1182	PE	PV	10 μM	333	355	EALPMDTEVyESPyADPEEIRPK	y10: Phospho (+79.97), y14: Phospho (+79.97)	99.70%	3.34	29.3	9.92	3	947.07	2,838.19	0.01233
1183	PE	PV	10 μM	333	355	EALPMDTEVyESPyADPEEIRPK	y10: Phospho (+79.97), y14: Phospho (+79.97)	99.70%	3.86	26	10.4	3	947.07	2,838.18	0.00061
1184	PE	PV	10 μM	333	355	EALPMDTEVyESPYADPEEIRPK	y10: Phospho (+79.97)	99.70%	3.65	66.8	10.8	3	920.41	2,758.21	0.00570
1185	PE	PV	10 μM	333	355	EALPMDTEVyESPYADPEEIRPK	y10: Phospho (+79.97)	99.70%	4.32	65.3	10.5	3	920.41	2,758.22	0.01009
1186	PE	PV	10 μM	333	355	EALPMDTEVYESPyADPEEIRPK	y14: Phospho (+79.97)	99.70%	2.07	41.2	7.22	3	920.41	2,758.22	0.01028
1187	PE	PV	10 μM	333	355	EALPMDTEVyESPYADPEEIRPK	y10: Phospho (+79.97)	99.70%	2.05	39.1	9.6	3	920.41	2,758.22	0.00735
1188	PE	PV	10 μM	333	355	EALPmDTEVYESPyADPEEIRPK	m5: Oxidation (+15.99), y14: Phospho (+79.97)	99.70%	2.67	50	8.33	3	925.74	2,774.20	0.00010
1189	PE	PV	10 μM	333	355	EALPmDTEVYESPyADPEEIRPK	m5: Oxidation (+15.99), y14: Phospho (+79.97)	99.70%	2.08	42.3	6.49	3	925.74	2,774.21	0.00816
1190	PE	PV	10 μM	333	355	EALPMDTEVyESPYADPEEIRPK	y10: Phospho (+79.97)	99.70%	2.89	40.8	10.5	3	920.41	2,758.22	0.01430
1191	PE	PV	10 μM	333	355	EALPMDTEVyESPYADPEEIRPK	y10: Phospho (+79.97)	99.70%	3.23	57.2	8.92	3	920.41	2,758.22	0.00790
1192	PE	PV	10 μM	333	355	EALPMDTEVYESPyADPEEIRPK	y14: Phospho (+79.97)	99.70%	4.2	41.8	9.11	3	920.41	2,758.22	0.01412
1193	PE	PV	10 μM	333	355	EALPmDTEVYESPyADPEEIRPK	m5: Oxidation (+15.99), y14: Phospho (+79.97)	83.10%	0.87	0	1.62	3	925.75	2,774.22	0.01310
1194	PE	PV	10 μM	333	355	EALPMDTEVYESPyADPEEIRPK	y14: Phospho (+79.97)	99.70%	2.52	45.3	7.2	3	920.41	2,758.21	0.00497
1195	PE	PV	10 μM	333	355	EALPmDTEVYESPyADPEEIRPK	m5: Oxidation (+15.99), y14: Phospho (+79.97)	95.70%	0	0	2.72	3	926.42	2,776.24	2.03600
1196	PE	PV	10 μM	333	355	EALPMDTEVYESPyADPEEIRPK	y14: Phospho (+79.97)	99.70%	2.45	27.9	7.8	3	920.43	2,758.26	0.04836
1197	PE	PV	10 μM	333	355	EALPMDTEVYESPyADPEEIRPK	y14: Phospho (+79.97)	99.70%	2.65	10.9	6.42	3	920.42	2,758.25	0.03811
1198	PE	PV	10 μM	333	355	EALPMDTEVYESPyADPEEIRPK	y14: Phospho (+79.97)	99.70%	2.75	32.2	8.21	3	920.43	2,758.26	0.04928
1199	PE	PV	10 μM	333	355	EALPMDTEVYESPyADPEEIRPK	y14: Phospho (+79.97)	99.70%	2.91	34.9	9.74	3	920.43	2,758.26	0.04818
1200	PE	PV	10 μM	333	355	EALPMDTEVYESPyADPEEIRPK	y14: Phospho (+79.97)	99.70%	2.67	29	9.44	3	920.43	2,758.25	0.04543
1201	PE	PV	10 μM	333	355	EALPMDtEVYESPYADPEEIRPK	t7: Phospho (+79.97)	99.70%	0	0	10.4	3	921.09	2,760.25	2.03700
1202	PE	PV	10 μM	333	355	EALPMDTEVYESPyADPEEIRPK	y14: Phospho (+79.97)	99.70%	2.57	37.5	10.7	3	920.43	2,758.26	0.04671
1203	PE	PV	30 μM	333	355	EALPMDTEVYESPyADPEEIRPK	y14: Phospho (+79.97)	99.70%	2.11	25.4	1.38	3	920.41	2,758.21	-0.00035
1204	PE	PV	30 μM	333	355	EALPmDTEVYEsPYADPEEIRPK	m5: Oxidation (+15.99), s12: Phospho (+79.97)	99.70%	2.29	26.4	2.89	3	925.74	2,774.21	0.00267
1205	PE	PV	30 μM	333	355	EALPmDTEVYESPyADPEEIRPK	m5: Oxidation (+15.99), y14: Phospho (+79.97)	99.70%	2.32	19.1	3.89	3	925.74	2,774.21	0.00743
1206	PE	PV	30 μM	333	355	EALPmDTEVyESPYADPEEIRPK	m5: Oxidation (+15.99), y10: Phospho (+79.97)	99.70%	1.37	18.9	3.89	3	925.74	2,774.21	0.00212
1207	PE	PV	30 μM	333	355	EALPMDTEVYESPyADPEEIRPK	y14: Phospho (+79.97)	99.70%	1.89	27.6	6.21	3	920.41	2,758.22	0.00790
1208	PE	PV	30 μM	333	355	EALPMDTEVYESPyADPEEIRPK	y14: Phospho (+79.97)	99.70%	3.19	52.6	8.52	3	920.41	2,758.21	0.00204
1209	PE	PV	30 μM	333	355	EALPMDTEVyESPYADPEEIRPK	y10: Phospho (+79.97)	99.70%	2.46	27.8	6.85	3	920.41	2,758.22	0.01357
1210	PE	PV	30 μM	333	355	EALPMDTEVYESPyADPEEIRPK	y14: Phospho (+79.97)	99.70%	3.9	36.5	7.54	3	920.42	2,758.23	0.01687
1211	PE	PV	30 μM	333	355	EALPMDTEVYESPyADPEEIRPK	y14: Phospho (+79.97)	99.70%	4.06	52.9	9.89	3	920.41	2,758.22	0.01192
1212	PE	PV	30 μM	333	355	EALPMDTEVyESPyADPEEIRPK	y10: Phospho (+79.97), y14: Phospho (+79.97)	99.70%	3.98	30	7.85	3	947.07	2,838.18	0.00574
1213	PE	PV	30 μM	333	355	EALPMDTEVYESPyADPEEIRPK	y14: Phospho (+79.97)	99.70%	3.27	43.1	9.96	3	920.42	2,758.22	0.01613

1214	PE	PV	30 μM	333	355	EALPmDTEVYESPyADPEEIRPK	m5: Oxidation (+15.99), y14: Phospho (+79.97)	99.70%	2.88	34	7.05	3	925.74	2,774.21	0.00505
1215	PE	PV	30 μM	333	355	EALPmDTEVyESPyADPEEIRPK	m5: Oxidation (+15.99), y10: Phospho (+79.97), y14: Phospho (+79.97)	99.70%	3.16	43.1	9.01	3	952.40	2,854.18	0.01058
1216	PE	PV	30 μM	333	355	EALPMDTEVyESPyADPEEIRPK	y10: Phospho (+79.97), y14: Phospho (+79.97)	99.70%	2.78	33.8	9.68	3	947.07	2,838.18	0.00317
1217	PE	PV	30 μM	333	355	EALPMDTEVyESPyADPEEIRPK	y10: Phospho (+79.97), y14: Phospho (+79.97)	99.70%	3.82	28	8.19	3	947.07	2,838.18	0.00262
1218	PE	PV	30 μM	333	355	EALPmDTEVyESPyADPEEIRPK	m5: Oxidation (+15.99), y10: Phospho (+79.97), y14: Phospho (+79.97)	99.70%	3.09	42.3	10.1	3	952.40	2,854.18	0.01314
1219	PE	PV	30 μM	333	355	EALPmDTEVyESPYADPEEIRPK	m5: Oxidation (+15.99), y10: Phospho (+79.97)	99.70%	3.3	39.2	7.6	3	925.74	2,774.21	0.00724
1220	PE	PV	30 μM	333	355	EALPmDTEVYESPyADPEEIRPK	m5: Oxidation (+15.99), y14: Phospho (+79.97)	99.70%	3.08	36	7.47	3	925.74	2,774.21	0.00834
1221	PE	PV	30 μM	333	355	EALPMDTEVyESPYADPEEIRPK	y10: Phospho (+79.97)	99.70%	3.68	43.5	10.3	3	920.41	2,758.22	0.01064
1222	PE	PV	30 μM	333	355	EALPMDTEVyESPYADPEEIRPK	y10: Phospho (+79.97)	99.70%	4.09	50.9	10.7	3	920.41	2,758.22	0.00863
1223	PE	PV	30 μM	333	355	EALPMDTEVyESPYADPEEIRPK	y10: Phospho (+79.97)	99.70%	3.7	53.9	10.5	3	920.41	2,758.22	0.00753
1224	PE	PV	30 μM	333	355	EALPmDTEVYEsPYADPEEIRPK	m5: Oxidation (+15.99), s12: Phospho (+79.97)	94.90%	0.25	0	1.38	2	1388.12	2,774.22	0.01293
1225	PE	PV	30 μM	333	355	EALPMDTEVYESPyADPEEIRPK	y14: Phospho (+79.97)	99.70%	1.3	14.2	2.64	3	920.41	2,758.21	-0.00126
1226	PE	PV	30 μM	333	355	EALPmDTEVYESPyADPEEIRPK	m5: Oxidation (+15.99), y14: Phospho (+79.97)	99.70%	1.27	16.6	5.19	3	925.75	2,774.21	0.01036
1227	PE	PV	30 μM	333	355	EALPmDTEVYEsPYADPEEIRPK	m5: Oxidation (+15.99), s12: Phospho (+79.97)	99.70%	1.32	22.8	2.37	3	925.74	2,774.20	-0.00851
1228	PE	PV	30 μM	333	355	EALPMDtEVYESPYADPEEIRPK	t7: Phospho (+79.97)	99.70%	2.96	58.4	14.3	3	920.41	2,758.20	-0.00474
1229	PE	PV	30 μM	333	355	EALPmDTEVYEsPYADPEEIRPK	m5: Oxidation (+15.99), s12: Phospho (+79.97)	99.70%	3.44	47.6	10.1	3	925.74	2,774.20	-0.00265
1230	PE	PV	30 μM	333	355	EALPMDTEVYESPyADPEEIRPK	y14: Phospho (+79.97)	99.70%	3.94	50.9	7.21	3	920.41	2,758.22	0.00881
1231	PE	PV	30 μM	333	355	EALPMDTEVYESPyADPEEIRPK	y14: Phospho (+79.97)	99.70%	2.9	16.4	5.64	3	920.41	2,758.20	-0.00399
1232	PE	PV	30 μM	333	355	eALPMDTEVYESPyADPEEIRPK	n-term: Dehydrated (-18.01), y14: Phospho (+79.97)	99.70%	0	0	9.7	3	914.41	2,740.19	-0.00480
1233	PE	PV	30 μM	333	355	EALPMDTEVyESPyADPEEIRPK	y10: Phospho (+79.97), y14: Phospho (+79.97)	99.70%	3.3	30.7	8.92	3	947.06	2,838.16	-0.01056
1234	PE	PV	30 μM	333	355	EALPMDTEVyESPYADPEEIRPK	y10: Phospho (+79.97)	99.70%	3.24	43.6	10.1	3	920.41	2,758.21	0.00313
1235	PE	PV	30 μM	333	355	EALPMDTEVyESPyADPEEIRPK	y10: Phospho (+79.97), y14: Phospho (+79.97)	99.70%	4.09	36	11.5	3	947.06	2,838.17	-0.00305
1236	PE	PV	30 μM	333	355	EALPmDTEVYEsPYADPEEIRPK	m5: Oxidation (+15.99), s12: Phospho (+79.97)	99.70%	3.09	40.1	6.6	3	925.74	2,774.20	-0.00100
1237	PE	PV	30 μM	333	355	EALPMDtEVYESPYADPEEIRPK	t7: Phospho (+79.97)	99.70%	3.56	53.9	12.3	3	920.41	2,758.21	0.00222
1238	PE	PV	30 μM	333	355	EALPMDTEVYESPyADPEEIRPK	y14: Phospho (+79.97)	99.70%	4.23	30	8.02	3	920.41	2,758.22	0.00643
1239	PE	PV	30 μM	333	355	EALPMDtEVYESPYADPEEIRPK	t7: Phospho (+79.97)	99.70%	3.3	50.6	12.5	3	920.41	2,758.22	0.01229
1240	PE	PV	30 μM	333	355	EALPmDTEVYESPyADPEEIRPK	m5: Oxidation (+15.99), y14: Phospho (+79.97)	99.70%	3.86	50.5	12.7	3	925.75	2,774.21	0.01054

1241	PE	PV	30 μM	333	355	EALPMDTEVYESPyADPEEIRPK	y14: Phospho (+79.97)	99.70%	3.69	40.9	9.37	3	920.41	2,758.22	0.00808
1242	PE	PV	30 μM	333	355	EALPmDTEVYEsPYADPEEIRPK	m5: Oxidation (+15.99), s12: Phospho (+79.97)	99.70%	0	0	4.37	3	926.09	2,775.24	1.04100
1243	PE	PV	30 μM	333	355	EALPmDTEVYESPyADPEEIRPK	m5: Oxidation (+15.99), y14: Phospho (+79.97)	99.70%	0	0	5.66	3	926.42	2,776.24	2.03700
1244	PE	PV	30 μM	333	355	EALPmDTEVYEsPYADPEEIRPK	m5: Oxidation (+15.99), s12: Phospho (+79.97)	99.70%	0	0	8.04	3	926.09	2,775.24	1.03600
1245	PE	PV	30 μM	333	355	EALPMDTEVYESPyADPEEIRPK	y14: Phospho (+79.97)	99.70%	2.97	16.6	7.49	3	920.43	2,758.25	0.04470
1246	PE	PV	30 μM	333	355	EALPMDTEVYESPyADPEEIRPK	y14: Phospho (+79.97)	99.70%	1.36	22	5.48	2	1380.13	2,758.25	0.03861
1247	PE	PV	30 μM	333	355	EALPMDTEVYESPyADPEEIRPK	y14: Phospho (+79.97)	99.70%	3.04	35.9	8	3	920.42	2,758.25	0.04232
1248	PE	PV	30 μM	333	355	EALPMDTEVYESPyADPEEIRPK	y14: Phospho (+79.97)	99.70%	2.98	34.8	8.52	3	920.42	2,758.25	0.03902
1249	PE	PV	30 μM	333	355	EALPMDTEVYEsPYADPEEIRPK	s12: Phospho (+79.97)	99.70%	1.86	10.7	8.22	3	920.42	2,758.24	0.02767
1250	PE	PV	30 μM	333	355	EALPMDTEVYEsPYADPEEIRPK	s12: Phospho (+79.97)	99.70%	2.28	34.3	8.68	3	920.43	2,758.26	0.04946
1251	PE	PV	30 μM	333	355	EALPMDTEVYESPyADPEEIRPK	y14: Phospho (+79.97)	99.70%	2.77	28.7	10.3	3	920.42	2,758.25	0.04287
1252	PE	PV	30 μM	333	355	EALPMDTEVYESPyADPEEIRPK	y14: Phospho (+79.97)	99.70%	2.87	46.8	10.9	3	920.43	2,758.25	0.04488
1253	PE	PV	30 μM	333	355	EALPmDTEVyESPYADPEEIRPK	m5: Oxidation (+15.99), y10: Phospho (+79.97)	99.70%	2.79	28	8.12	3	925.75	2,774.24	0.03544
1254	PE	PV	30 μM	333	355	EALPMDTEVYESPyADPEEIRPK	y14: Phospho (+79.97)	99.70%	0	20.5	10.8	3	920.43	2,758.26	0.05367
1255	PE	PV	30 μM	333	355	EALPMDTEVYEsPYADPEEIRPK	s12: Phospho (+79.97)	99.70%	2.79	25.6	12.4	3	920.42	2,758.25	0.03774
1256	PE	PV	30 μM	333	355	EALPMDtEVYESPYADPEEIRPK	t7: Phospho (+79.97)	99.70%	2.76	33.4	14.2	3	920.42	2,758.25	0.03994
1257	PE	PV	30 μM	333	355	EALPMDtEVYESPYADPEEIRPK	t7: Phospho (+79.97)	99.70%	2.78	43.8	12.1	3	920.43	2,758.26	0.04671
1258	PE	PV	30 μM	333	355	EALPMDTEVyESPYADPEEIRPK	y10: Phospho (+79.97)	99.70%	2.96	41.1	13.9	3	920.42	2,758.25	0.03884
1259	PE	PV	30 μM	333	355	EALPMDTEVyESPYADPEEIRPK	y10: Phospho (+79.97)	99.70%	3.13	56.9	12.2	3	920.42	2,758.25	0.03628
1260	PE	PV	30 μM	333	355	EALPMDTEVyESPYADPEEIRPK	y10: Phospho (+79.97)	99.70%	3.16	54.3	11	3	920.42	2,758.25	0.03774
1261	PE	PV	30 μM	333	355	EALPMDtEVYESPYADPEEIRPK	t7: Phospho (+79.97)	99.70%	0	0	11.5	3	921.09	2,760.25	2.04200
1262	PE	PV	30 μM	333	355	EALPMDtEVYESPYADPEEIRPK	t7: Phospho (+79.97)	99.70%	0	0	12	3	921.09	2,760.25	2.04200
1263	PE	PV	100 μM	333	355	EALPMDTEVYESPyADPEEIRPK	y14: Phospho (+79.97)	95.20%	1.51	0	1.05	3	920.41	2,758.21	0.00020
1264	PE	PV	100 μM	333	355	EALPmDTEVYESPyADPEEIRPK	m5: Oxidation (+15.99), y14: Phospho (+79.97)	96.40%	1.95	0	1.19	3	925.74	2,774.21	0.00578
1265	PE	PV	100 μM	333	355	EALPMDTEVYEsPYADPEEIRPK	s12: Phospho (+79.97)	99.70%	2.49	36.3	2.62	3	920.41	2,758.20	-0.00456
1266	PE	PV	100 μM	333	355	EALPMDTEVYESPyADPEEIRPK	y14: Phospho (+79.97)	99.70%	2.19	27.4	5.24	3	920.41	2,758.21	0.00460
1267	PE	PV	100 μM	333	355	EALPmDTEVyESPyADPEEIRPK	m5: Oxidation (+15.99), y10: Phospho (+79.97), y14: Phospho (+79.97)	99.70%	1.71	21.3	5.11	3	952.40	2,854.17	0.00215
1268	PE	PV	100 μM	333	355	EALPmDTEVYESPyADPEEIRPK	m5: Oxidation (+15.99), y14: Phospho (+79.97)	99.70%	3.07	28.5	6.1	3	925.74	2,774.21	0.00431
1269	PE	PV	100 μM	333	355	EALPMDTEVyESPyADPEEIRPK	y10: Phospho (+79.97), y14: Phospho (+79.97)	99.70%	2.11	30.1	8.24	3	947.06	2,838.17	-0.00800
1270	PE	PV	100 μM	333	355	EALPMDTEVyESPYADPEEIRPK	y10: Phospho (+79.97)	99.70%	2.15	53.4	10	3	920.41	2,758.21	0.00149
1271	PE	PV	100 μM	333	355	eALPMDTEVYEsPYADPEEIRPK	n-term: Dehydrated (-18.01), s12: Phospho (+79.97)	99.70%	0	0	6.92	3	915.08	2,742.21	2.01300
1272	PE	PV	100 μM	333	355	EALPMDTEVYESPyADPEEIRPK	y14: Phospho (+79.97)	99.70%	3.68	34.4	7.72	3	920.41	2,758.22	0.01211
1273	PE	PV	100 μM	333	355	EALPMDTEVyESPyADPEEIRPK	y10: Phospho (+79.97), y14: Phospho (+79.97)	99.70%	2.76	39.7	5.92	3	947.07	2,838.18	0.00793
1274	PE	PV	100 μM	333	355	EALPMDTEVYESPyADPEEIRPK	y14: Phospho (+79.97)	99.70%	4.04	28	8.15	3	920.41	2,758.22	0.00808

1275	PE	PV	100 µM	333	355	EALPmDTEVyESPyADPEEIRPK	m5: Oxidation (+15.99), y10: Phospho (+79.97), y14: Phospho (+79.97)	99.70%	2.56	25.3	9.01	3	952.39	2,854.16	-0.00902
1276	PE	PV	100 µM	333	355	EALPmDTEVYESPyADPEEIRPK	m5: Oxidation (+15.99), y14: Phospho (+79.97)	99.70%	3.44	29.4	7.82	3	925.75	2,774.21	0.01072
1277	PE	PV	100 µM	333	355	EALPMDTEVYESPyADPEEIRPK	y14: Phospho (+79.97)	99.70%	3	42.7	11	3	920.42	2,758.22	0.01540
1278	PE	PV	100 µM	333	355	EALPmDTEVYESPyADPEEIRPK	m5: Oxidation (+15.99), y14: Phospho (+79.97)	99.70%	2.86	17.2	8.96	3	925.75	2,774.21	0.01072
1279	PE	PV	100 µM	333	355	EALPmDTEVyESPyADPEEIRPK	m5: Oxidation (+15.99), y10: Phospho (+79.97), y14: Phospho (+79.97)	99.70%	3.89	49.4	9.34	3	952.40	2,854.18	0.00856
1280	PE	PV	100 µM	333	355	EALPmDTEVyESPyADPEEIRPK	m5: Oxidation (+15.99), y10: Phospho (+79.97), y14: Phospho (+79.97)	99.70%	3.51	21.1	10	3	952.40	2,854.18	0.01113
1281	PE	PV	100 µM	333	355	EALPmDTEVyESPyADPEEIRPK	m5: Oxidation (+15.99), y10: Phospho (+79.97), y14: Phospho (+79.97)	99.70%	2.75	27	10.6	3	952.40	2,854.18	0.00527
1282	PE	PV	100 µM	333	355	EALPMDTEVyESPyADPEEIRPK	y10: Phospho (+79.97), y14: Phospho (+79.97)	99.70%	4.07	29.4	12.2	3	947.07	2,838.18	0.00683
1283	PE	PV	100 µM	333	355	EALPMDTEVyESPyADPEEIRPK	y10: Phospho (+79.97), y14: Phospho (+79.97)	99.70%	4.48	31.4	12.4	3	947.07	2,838.18	0.00390
1284	PE	PV	100 µM	333	355	EALPmDTEVyESPYADPEEIRPK	m5: Oxidation (+15.99), y10: Phospho (+79.97)	99.70%	3.3	31.6	8.4	3	925.74	2,774.21	0.00944
1285	PE	PV	100 µM	333	355	EALPMDTEVyESPYADPEEIRPK	y10: Phospho (+79.97)	99.70%	3.23	57.1	9.59	3	920.42	2,758.23	0.01723
1286	PE	PV	100 µM	333	355	EALPMDTEVyESPYADPEEIRPK	y10: Phospho (+79.97)	99.70%	4.1	41.1	11.4	3	920.41	2,758.22	0.00918
1287	PE	PV	100 µM	333	355	EALPMDTEVyESPYADPEEIRPK	y10: Phospho (+79.97)	99.70%	4.18	58.6	11.9	3	920.41	2,758.22	0.00936
1288	PE	PV	100 µM	333	355	EALPmDTEVyESPYADPEEIRPK	m5: Oxidation (+15.99), y10: Phospho (+79.97)	99.70%	3.95	64.9	11.2	3	925.74	2,774.21	0.00889
1289	PE	PV	100 µM	333	355	EALPmDTEVyESPYADPEEIRPK	m5: Oxidation (+15.99), y10: Phospho (+79.97)	99.70%	4.19	52.2	9.41	3	925.75	2,774.21	0.01054
1290	PE	PV	100 µM	333	355	EALPMDTEVyESPYADPEEIRPK	y10: Phospho (+79.97)	99.70%	4.49	65	10.4	3	920.41	2,758.22	0.00826
1291	PE	PV	100 µM	333	355	EALPMDTEVYESPyADPEEIRPK	y14: Phospho (+79.97)	99.70%	1.72	28.1	2.54	3	920.41	2,758.21	-0.00071
1292	PE	PV	100 µM	333	355	EALPmDTEVYESPyADPEEIRPK	m5: Oxidation (+15.99), y14: Phospho (+79.97)	99.70%	1.92	37.7	6.55	3	925.74	2,774.20	-0.00576
1293	PE	PV	100 µM	333	355	EALPmDtEVYESPYADPEEIRPK	m5: Oxidation (+15.99), t7: Phospho (+79.97)	99.70%	3.08	47.4	11.3	3	925.74	2,774.20	-0.00759
1294	PE	PV	100 µM	333	355	eALPMDTEVYESPyADPEEIRPK	n-term: Dehydrated (-18.01), y14: Phospho (+79.97)	99.30%	0	0	4.55	3	914.40	2,740.19	-0.00846
1295	PE	PV	100 µM	333	355	EALPmDTEVYEsPyADPEEIRPK	m5: Oxidation (+15.99), s12: Phospho (+79.97), y14: Phospho (+79.97)	99.70%	0	20.7	7.96	3	952.41	2,854.20	0.02815
1296	PE	PV	100 µM	333	355	EALPMDTEVYESPyADPEEIRPK	y14: Phospho (+79.97)	99.70%	1.78	18.2	5.38	3	920.41	2,758.20	-0.01206
1297	PE	PV	100 µM	333	355	EALPmDTEVyESPyADPEEIRPK	m5: Oxidation (+15.99), y10: Phospho (+79.97), y14: Phospho (+79.97)	99.70%	3.58	39.1	6.66	3	952.40	2,854.17	0.00325

1298	PE	PV	100 µM	333	355	EALPMDTEVyESPyADPEEIRPK	y10: Phospho (+79.97), y14: Phospho (+79.97)	99.70%	3.49	29.1	8.68	3	947.06	2,838.16	-0.01697
1299	PE	PV	100 µM	333	355	EALPmDTEVyESPyADPEEIRPK	m5: Oxidation (+15.99), y10: Phospho (+79.97), y14: Phospho (+79.97)	99.70%	3.22	45.8	7.49	3	952.40	2,854.17	-0.00352
1300	PE	PV	100 µM	333	355	EALPMDTEVyESPYADPEEIRPK	y10: Phospho (+79.97)	99.70%	2.98	45.2	8.23	3	920.41	2,758.21	0.00130
1301	PE	PV	100 µM	333	355	EALPMDTEVyESPyADPEEIRPK	y10: Phospho (+79.97), y14: Phospho (+79.97)	99.70%	3.82	36	7.7	3	947.06	2,838.17	-0.00580
1302	PE	PV	100 µM	333	355	EALPMDTEVyESPyADPEEIRPK	y10: Phospho (+79.97), y14: Phospho (+79.97)	99.70%	2.95	29.6	8	3	947.06	2,838.17	-0.00269
1303	PE	PV	100 µM	333	355	EALPMDTEVYESPyADPEEIRPK	y14: Phospho (+79.97)	99.70%	3.76	38.4	9.31	3	920.41	2,758.20	-0.00511
1304	PE	PV	100 µM	333	355	EALPmDTEVYESPyADPEEIRPK	m5: Oxidation (+15.99), y14: Phospho (+79.97)	99.70%	2.9	27.4	9.06	3	925.74	2,774.21	0.00212
1305	PE	PV	100 µM	333	355	EALPmDTEVYESPyADPEEIRPK	m5: Oxidation (+15.99), y14: Phospho (+79.97)	99.70%	3.28	33.7	9.44	3	925.74	2,774.21	0.00285
1306	PE	PV	100 µM	333	355	EALPMDTEVYEsPYADPEEIRPK	s12: Phospho (+79.97)	99.70%	2.66	38.1	9.41	3	920.41	2,758.21	-0.00071
1307	PE	PV	100 µM	333	355	EALPMDtEVYESPYADPEEIRPK	t7: Phospho (+79.97)	99.70%	3.96	48.2	9.57	3	920.41	2,758.21	-0.00163
1308	PE	PV	100 µM	333	355	EALPmDTEVyESPYADPEEIRPK	m5: Oxidation (+15.99), y10: Phospho (+79.97)	99.70%	3.95	56.8	9.41	3	925.74	2,774.20	0.00010
1309	PE	PV	100 µM	333	355	EALPmDTEVyESPYADPEEIRPK	m5: Oxidation (+15.99), y10: Phospho (+79.97)	99.70%	4.05	56.8	10.3	3	925.74	2,774.20	0.00120
1310	PE	PV	100 µM	333	355	EALPmDtEVYESPYADPEEIRPK	m5: Oxidation (+15.99), t7: Phospho (+79.97)	86.60%	0.99	0	0	3	925.74	2,774.21	0.00798
1311	PE	PV	100 µM	333	355	EALPMDTEVYEsPYADPEEIRPK	s12: Phospho (+79.97)	99.70%	2.67	33.2	13.1	3	920.41	2,758.22	0.00973
1312	PE	PV	100 µM	333	355	EALPmDTEVYESPyADPEEIRPK	m5: Oxidation (+15.99), y14: Phospho (+79.97)	99.70%	1.26	18.5	3.6	3	925.74	2,774.20	-0.00228
1313	PE	PV	100 µM	333	355	EALPMDTEVYESPyADPEEIRPK	y14: Phospho (+79.97)	99.70%	4.02	39.4	9.39	3	920.41	2,758.22	0.00716
1314	PE	PV	100 µM	333	355	EALPmDTEVYEsPYADPEEIRPK	m5: Oxidation (+15.99), s12: Phospho (+79.97)	99.70%	3.93	60.8	9.74	3	925.74	2,774.21	0.00596
1315	PE	PV	100 µM	333	355	EALPmDTEVyESPyADPEEIRPK	m5: Oxidation (+15.99), y10: Phospho (+79.97), y14: Phospho (+79.97)	90.90%	2.14	0	2.43	3	952.41	2,854.21	0.04482
1316	PE	PV	100 µM	333	355	EALPmDTEVyESPyADPEEIRPK	m5: Oxidation (+15.99), y10: Phospho (+79.97), y14: Phospho (+79.97)	97.50%	1.87	0	2.72	3	952.41	2,854.21	0.03749
1317	PE	PV	100 µM	333	355	EALPmDTEVYEsPyADPEEIRPK	m5: Oxidation (+15.99), s12: Phospho (+79.97), y14: Phospho (+79.97)	99.70%	0	0	4.18	3	952.75	2,855.22	1.04600
1318	PE	PV	100 µM	333	355	EALPmDtEVYEsPYADPEEIRPK	m5: Oxidation (+15.99), t7: Phospho (+79.97), s12: Phospho (+79.97)	99.70%	1.89	0	5.34	3	952.41	2,854.21	0.03768
1319	PE	PV	100 µM	333	355	EALPmDtEVYEsPYADPEEIRPK	m5: Oxidation (+15.99), t7: Phospho (+79.97), s12: Phospho (+79.97)	99.70%	1.93	11.7	3.89	3	952.41	2,854.22	0.04738

1320	PE	PV	100 µM	333	355	EALPmDTEVYESPyADPEEIRPK	m5: Oxidation (+15.99), y14: Phospho (+79.97)	99.70%	0	0	6.77	3	926.42	2,776.24	2.03300
1321	PE	PV	100 µM	333	355	EALPmDTEVYESPyADPEEIRPK	m5: Oxidation (+15.99), y14: Phospho (+79.97)	99.70%	0	0	7.14	3	926.09	2,775.24	1.03400
1322	PE	PV	100 µM	333	355	EALPmDTEVYESPyADPEEIRPK	m5: Oxidation (+15.99), y14: Phospho (+79.97)	99.70%	0	0	7	3	926.09	2,775.24	1.03600
1323	PE	PV	100 µM	333	355	EALPmDTEVYEsPYADPEEIRPK	m5: Oxidation (+15.99), s12: Phospho (+79.97)	99.70%	0	0	7.74	3	926.09	2,775.24	1.04100
1324	PE	PV	100 µM	333	355	EALPMDTEVYEsPYADPEEIRPK	s12: Phospho (+79.97)	99.70%	2.27	14.3	6.74	3	920.42	2,758.25	0.04250
1325	PE	PV	100 µM	333	355	EALPmDTEVyESPyADPEEIRPK	m5: Oxidation (+15.99), y10: Phospho (+79.97), y14: Phospho (+79.97)	99.70%	2.7	20.2	5.96	3	952.41	2,854.22	0.04573
1326	PE	PV	100 µM	333	355	EALPmDTEVYESPyADPEEIRPK	m5: Oxidation (+15.99), y14: Phospho (+79.97)	99.70%	2.86	17.6	5.89	3	925.75	2,774.23	0.02867
1327	PE	PV	100 µM	333	355	EALPmDTEVyESPyADPEEIRPK	m5: Oxidation (+15.99), y10: Phospho (+79.97), y14: Phospho (+79.97)	99.70%	2.58	16.6	7.28	3	952.41	2,854.22	0.04720
1328	PE	PV	100 µM	333	355	EALPmDTEVYESPyADPEEIRPK	m5: Oxidation (+15.99), y14: Phospho (+79.97)	99.70%	0	0	10.2	3	926.09	2,775.24	1.03900
1329	PE	PV	100 µM	333	355	EALPmDTEVyESPYADPEEIRPK	m5: Oxidation (+15.99), y10: Phospho (+79.97)	99.70%	3.68	38	7.96	3	925.75	2,774.24	0.03819
1330	PE	PV	100 µM	333	355	EALPmDTEVyESPYADPEEIRPK	m5: Oxidation (+15.99), y10: Phospho (+79.97)	99.70%	3.91	35.5	8.13	3	925.75	2,774.24	0.03929
1331	PE	PV	100 µM	333	355	EALPmDTEVYESPyADPEEIRPK	m5: Oxidation (+15.99), y14: Phospho (+79.97)	99.70%	3.89	24	8.21	3	925.75	2,774.24	0.03691
1332	PE	PV	100 µM	333	355	EALPmDTEVYESPyADPEEIRPK	m5: Oxidation (+15.99), y14: Phospho (+79.97)	99.70%	3.96	29.9	8.25	3	925.75	2,774.24	0.03489
1333	PE	PV	100 µM	333	355	EALPmDTEVYESPyADPEEIRPK	m5: Oxidation (+15.99), y14: Phospho (+79.97)	99.70%	3.06	24.7	8.66	3	925.75	2,774.24	0.03361
1334	PE	PV	100 µM	333	355	EALPMDTEVyESPyADPEEIRPK	y10: Phospho (+79.97), y14: Phospho (+79.97)	99.70%	2.78	13.2	9.1	3	947.08	2,838.21	0.03796
1335	PE	PV	100 µM	333	355	EALPMDTEVyESPyADPEEIRPK	y10: Phospho (+79.97), y14: Phospho (+79.97)	99.70%	2.41	12.5	10.8	3	947.08	2,838.22	0.04126
1336	PE	PV	100 µM	333	355	EALPMDTEVYESPyADPEEIRPK	y14: Phospho (+79.97)	99.70%	2.52	18	10.4	3	920.42	2,758.23	0.02144
1337	PE	PV	100 µM	333	355	EALPMDTEVyESPyADPEEIRPK	y10: Phospho (+79.97), y14: Phospho (+79.97)	99.70%	2.54	13.1	10	3	947.08	2,838.21	0.03393
1338	PE	PV	100 µM	333	355	EALPMDtEVYEsPYADPEEIRPK	t7: Phospho (+79.97), s12: Phospho (+79.97)	99.70%	2.77	10.7	10.9	3	947.08	2,838.21	0.03924
1339	PE	PV	100 µM	333	355	EALPmDTEVyESPYADPEEIRPK	m5: Oxidation (+15.99), y10: Phospho (+79.97)	99.70%	2.48	28	9.47	3	925.75	2,774.24	0.03214
1340	PE	PV	100 µM	333	355	EALPMDTEVyESPyADPEEIRPK	y10: Phospho (+79.97), y14: Phospho (+79.97)	99.70%	2.89	13.4	10.1	3	947.08	2,838.21	0.03851
1341	PE	PV	100 µM	333	355	EALPMDTEVyESPyADPEEIRPK	y10: Phospho (+79.97), y14: Phospho (+79.97)	99.70%	2.95	12.3	11.3	3	947.08	2,838.21	0.03851

1342	PE	PV	100 μM	333	355	EALPMDTEVyESPyADPEEIRPK	y10: Phospho (+79.97), y14: Phospho (+79.97)	99.70%	3.06	20.8	11.3	3	947.08	2,838.21	0.03924
1343	PE	PV	100 μM	333	355	EALPMDtEVYESPYADPEEIRPK	t7: Phospho (+79.97)	99.70%	3.03	44.4	11.9	3	920.42	2,758.25	0.04177
1344	PE	PV	100 μM	333	355	EALPMDTEVyESPyADPEEIRPK	y10: Phospho (+79.97), y14: Phospho (+79.97)	99.70%	3.22	28.1	11.4	3	947.08	2,838.21	0.03760
1345	PE	PV	100 μM	333	355	EALPMDTEVyESPYADPEEIRPK	y10: Phospho (+79.97)	99.70%	3.07	51.2	11.4	3	920.42	2,758.24	0.03390
1346	PE	PV	100 μM	333	355	EALPMDTEVyESPYADPEEIRPK	y10: Phospho (+79.97)	99.70%	3.17	56.8	10.8	3	920.42	2,758.24	0.03536
1347	PE	PV	100 μM	333	355	EALPmDTEVYESPyADPEEIRPK	m5: Oxidation (+15.99), y14: Phospho (+79.97)	99.70%	3.87	28.6	10.4	3	925.75	2,774.24	0.03453
1348	PE	PV	100 μM	333	355	EALPMDTEVyESPYADPEEIRPK	y10: Phospho (+79.97)	99.70%	2.96	44.5	12.7	3	920.42	2,758.24	0.03536
1349	PE	PV	100 μM	333	355	EALPMDTEVyESPYADPEEIRPK	y10: Phospho (+79.97)	99.70%	3.44	56.9	13.1	3	920.42	2,758.25	0.04195
1350	PE	PV	100 μM	333	355	EALPMDTEVyESPYADPEEIRPK	y10: Phospho (+79.97)	99.70%	3.35	44.5	12.4	3	920.42	2,758.25	0.03829
1351	PE	PV	100 μM	333	355	EALPMDTEVyESPYADPEEIRPK	y10: Phospho (+79.97)	99.70%	3.44	57.3	12.2	3	920.42	2,758.24	0.03408
1352	PE	PV	100 μM	333	355	EALPmDTEVyESPYADPEEIRPK	m5: Oxidation (+15.99), y10: Phospho (+79.97)	99.70%	3.45	40.6	10.1	3	925.75	2,774.24	0.03581
1353	PE	PV	100 μM	333	355	EALPmDTEVyESPYADPEEIRPK	m5: Oxidation (+15.99), y10: Phospho (+79.97)	99.70%	3.57	56.6	9.4	3	925.75	2,774.24	0.03654
1354	PE	PV	100 μM	333	355	EALPMDTEVYESPyADPEEIRPK	y14: Phospho (+79.97)	99.70%	2.67	41.1	4	3	920.41	2,758.21	0.00588
1355	PE	PV	100 μM	333	355	EALPmDTEVyESPYADPEEIRPK	m5: Oxidation (+15.99), y10: Phospho (+79.97)	99.70%	2.48	33.2	7.72	3	925.74	2,774.20	-0.00576
1356	PE	Hg	100 μM	356	361	EVyLDR	y3: Phospho (+79.97)	90.00%	1.85	28	1.68	2	437.69	873.363	-0.00039
1357	PE	Hg	100 μM	356	361	EVyLDR	y3: Phospho (+79.97)	90.00%	1.59	23.4	2.04	2	437.70	873.377	0.01316
1358	PE	Hg	100 μM	493	503	NVLLVTQHyAK	y9: Phospho (+79.97)	99.70%	2.97	25.2	2.51	2	683.35	1,364.69	0.00427
1359	IP	PV	0	512	521	ALRADENYyK	y9: Phospho (+79.97)	99.60%	2.18	20.8	2.66	2	661.79	1,321.57	0.00172
1360	PE	Ig	NT	512	521	ALRADENyYK	y8: Phospho (+79.97)	83.10%	1.25	40.3	0	2	661.79	1,321.57	0.00220
1361	PE	Ig	NT	512	521	ALRADENyyK	y8: Phospho (+79.97), y9: Phospho (+79.97)	97.80%	0.87	12.5	0	2	701.78	1,401.54	0.00218
1362	PE	Ig	1 min	512	521	ALRADENyYK	y8: Phospho (+79.97)	95.90%	2.83	30.1	0	2	661.79	1,321.57	0.00159
1363	PE	Ig	1 min	512	521	ALRADENYyK	y9: Phospho (+79.97)	92.30%	0.65	18	0	2	661.79	1,321.57	0.00233
1364	PE	Ig	1 min	512	521	ALRADENYyK	y9: Phospho (+79.97)	97.90%	1.03	12.9	0	2	661.79	1,321.57	0.00135
1365	PE	Ig	1 min	512	521	ALRADENyYK	y8: Phospho (+79.97)	99.70%	1.66	16.2	0	3	441.53	1,321.58	0.00509
1366	PE	Ig	1 min	512	521	ALRADENyyK	y8: Phospho (+79.97), y9: Phospho (+79.97)	99.70%	1.07	29.8	0	2	701.78	1,401.54	0.00133
1367	PE	Ig	2 min	512	521	ALRADENyYK	y8: Phospho (+79.97)	99.60%	1.76	42.9	0	2	661.79	1,321.57	0.00220
1368	PE	Ig	2 min	512	521	ALRADENyyK	y8: Phospho (+79.97), y9: Phospho (+79.97)	99.70%	1.51	27.8	0	2	701.78	1,401.54	0.00157
1369	PE	Ig	2 min	512	521	ALRADENyYK	y8: Phospho (+79.97)	99.70%	2	18.5	0	3	441.53	1,321.57	0.00317
1370	PE	Ig	3 min	512	521	ALRADENyYK	y8: Phospho (+79.97)	95.40%	2.13	11.3	0	3	441.53	1,321.57	0.00060
1371	PE	Ig	3 min	512	521	ALRADENYyK	y9: Phospho (+79.97)	97.80%	0.7	20.9	0	2	661.79	1,321.57	-0.00048
1372	PE	Ig	3 min	512	521	ALRADENyyK	y8: Phospho (+79.97), y9: Phospho (+79.97)	99.70%	1.28	33.3	0	2	701.78	1,401.54	0.00035
1373	PE	Ig	3 min	512	521	ALRADENyYK	y8: Phospho (+79.97)	99.70%	2.33	44.6	0	2	661.79	1,321.57	0.00111
1374	PE	Ig	5 min	512	521	ALRADENyYK	y8: Phospho (+79.97)	98.50%	1.13	12.5	0	3	441.53	1,321.57	-0.00123
1375	PE	Ig	5 min	512	521	ALRADENyyK	y8: Phospho (+79.97), y9: Phospho (+79.97)	88.60%	0.5	0	0	2	701.78	1,401.54	0.00572

1376	PE	Ig	5 min	512	521	ALRADENyYK	y8: Phospho (+79.97)	97.40%	1.46	38.6	0	2	661.79	1,321.57	0.00062
1377	PE	Ig	5 min	512	521	ALRADENyYK	y8: Phospho (+79.97)	99.70%	2.22	16.9	0	3	441.53	1,321.57	0.00408
1378	PE	Ig	15 min	512	521	ALRADENyYK	y8: Phospho (+79.97)	97.00%	0.7	13.4	0	2	661.79	1,321.57	0.00123
1379	PE	Ig	15 min	512	521	ALRADENyYK	y8: Phospho (+79.97)	99.60%	2.33	43.2	0	2	661.79	1,321.57	0.00220
1380	PE	Ig	15 min	512	521	ALRADENyyK	y8: Phospho (+79.97), y9: Phospho (+79.97)	99.70%	2.11	32.4	0	2	701.78	1,401.54	0.00182
1381	PE	Hg	0	512	521	ALRADENYyK	y9: Phospho (+79.97)	98.40%	1.86	0	2.24	2	661.79	1,321.58	0.00477
1382	PE	Hg	0	512	521	ALRADENyYK	y8: Phospho (+79.97)	99.70%	1.57	30.1	3.48	2	661.79	1,321.57	-0.00439
1383	PE	Hg	0	512	521	ALRADENyyK	y8: Phospho (+79.97), y9: Phospho (+79.97)	81.70%	0.49	0	0	2	701.78	1,401.54	0.00450
1384	PE	Hg	1 μM	512	521	ALRADENyYK	y8: Phospho (+79.97)	99.70%	1.17	14.2	2.4	3	441.53	1,321.58	0.00500
1385	PE	Hg	1 μM	512	521	ALRADENyyK	y8: Phospho (+79.97), y9: Phospho (+79.97)	99.70%	1.47	30.6	2.07	2	701.78	1,401.54	0.00267
1386	PE	Hg	5 μM	512	521	ALRADENYyK	y9: Phospho (+79.97)	99.70%	2.96	19.2	4.27	2	661.79	1,321.57	0.00233
1387	PE	Hg	5 μM	512	521	ALRADENyYK	y8: Phospho (+79.97)	95.60%	0.91	17.3	0	2	661.79	1,321.57	-0.00036
1388	PE	Hg	5 μM	512	521	ALRADENyyK	y8: Phospho (+79.97), y9: Phospho (+79.97)	99.70%	1.81	41.4	7.66	2	701.78	1,401.54	0.00145
1389	PE	Hg	10 μM	512	521	ALRADENYyK	y9: Phospho (+79.97)	99.00%	1.72	0	2.54	2	661.80	1,321.58	0.00733
1390	PE	Hg	10 μM	512	521	ALRADENyYK	y8: Phospho (+79.97)	99.70%	2.56	30.8	2.12	2	661.79	1,321.57	0.00281
1391	PE	Hg	10 μM	512	521	ALRADENYyK	y9: Phospho (+79.97)	99.70%	0.67	18.4	0	2	661.79	1,321.56	-0.00707
1392	PE	Hg	10 μM	512	521	ALRADENYyK	y9: Phospho (+79.97)	96.10%	0.5	11.7	0	2	661.79	1,321.57	0.00281
1393	PE	Hg	10 μM	512	521	ALRADENyYK	y8: Phospho (+79.97)	98.70%	0.69	13.5	0.27	2	661.80	1,321.58	0.00587
1394	PE	Hg	10 μM	512	521	ALRADENyYK	y8: Phospho (+79.97)	99.70%	1.51	11.2	3.15	3	441.53	1,321.58	0.00665
1395	PE	Hg	10 μM	512	521	ALRADENyyK	y8: Phospho (+79.97), y9: Phospho (+79.97)	99.70%	1.15	18.2	2.8	2	701.78	1,401.54	0.00352
1396	PE	Hg	20 μM	512	521	ALRADENyYK	y8: Phospho (+79.97)	99.70%	1.14	0	1.06	3	441.53	1,321.57	0.00097
1397	PE	Hg	20 μM	512	521	ALRADENyYK	y8: Phospho (+79.97)	99.70%	1.39	12.4	1.22	3	441.53	1,321.57	0.00308
1398	PE	Hg	20 μM	512	521	ALRADENyYK	y8: Phospho (+79.97)	99.70%	1.53	26.7	1.92	2	661.79	1,321.57	0.00306
1399	PE	Hg	20 μM	512	521	ALRADENyyK	y8: Phospho (+79.97), y9: Phospho (+79.97)	99.70%	1.29	25	2.14	2	701.78	1,401.54	0.00084
1400	PE	Hg	50 μM	512	521	ALRADENyYK	y8: Phospho (+79.97)	99.70%	2.95	14.6	0.82	3	441.53	1,321.57	0.00198
1401	PE	Hg	50 μM	512	521	ALRADENyYK	y8: Phospho (+79.97)	99.70%	2.7	25.1	2.74	2	661.79	1,321.57	0.00294
1402	PE	Hg	50 μM	512	521	ALRADENyYK	y8: Phospho (+79.97)	99.70%	1.32	24.8	2.06	2	661.79	1,321.57	0.00001
1403	PE	Hg	50 μM	512	521	ALRADENyYK	y8: Phospho (+79.97)	99.70%	1.4	13.5	1.3	3	441.53	1,321.57	-0.00040
1404	PE	Hg	50 μM	512	521	ALRADENyYK	y8: Phospho (+79.97)	99.70%	1.64	38.1	4.74	2	661.79	1,321.57	0.00428
1405	PE	Hg	50 μM	512	521	ALRADENyyK	y8: Phospho (+79.97), y9: Phospho (+79.97)	99.70%	1.27	31.4	2.8	2	701.78	1,401.54	0.00780
1406	PE	Hg	50 μM	512	521	ALRADENyYK	y8: Phospho (+79.97)	99.70%	1.65	25.3	4.57	3	441.53	1,321.58	0.00610
1407	PE	Hg	100 μM	512	521	ALRADENYyK	y9: Phospho (+79.97)	99.70%	2.57	21	1.92	2	661.80	1,321.58	0.00867
1408	PE	Hg	100 μM	512	521	ALRADENYyK	y9: Phospho (+79.97)	99.70%	2.83	28.5	4.19	2	661.79	1,321.57	0.00123
1409	PE	Hg	100 μM	512	521	ALRADENyYK	y8: Phospho (+79.97)	99.70%	2.19	34.4	5.04	2	661.79	1,321.57	-0.00317
1410	PE	Hg	100 μM	512	521	ALRADENyYK	y8: Phospho (+79.97)	99.70%	2.79	20.1	5.39	3	441.53	1,321.57	-0.00324
1411	PE	Hg	100 μM	512	521	ALRADENyYK	y8: Phospho (+79.97)	99.70%	1.72	48.4	7.38	2	661.79	1,321.57	0.00196
1412	PE	Hg	100 μM	512	521	ALRADENyyK	y8: Phospho (+79.97), y9: Phospho (+79.97)	99.70%	1.29	29.3	4.51	2	701.78	1,401.54	0.00499

1413	PE	PV	0	512	521	ALRADENyYK	y8: Phospho (+79.97)	99.70%	2.08	16.6	2.55	2	661.79	1,321.58	0.00477
1414	PE	PV	0	512	521	ALRADENyyK	y8: Phospho (+79.97), y9: Phospho (+79.97)	99.70%	1.09	25.3	1.16	2	701.78	1,401.54	0.00499
1415	PE	PV	0	512	521	ALRADENyYK	y8: Phospho (+79.97)	99.70%	1.64	47.2	4.66	2	661.79	1,321.57	0.00257
1416	PE	PV		512	521	ALRADENyYK	y8: Phospho (+79.97)	99.70%	1.77	19.5	3.05	3	441.53	1,321.57	-0.00077
1417	PE	PV	1 µM	512	521	ALRADENyyK	y8: Phospho (+79.97), y9: Phospho (+79.97)	99.70%	1.19	22.7	2.89	2	701.78	1,401.54	0.00352
1418	PE	PV	3 µM	512	521	ALRADENyYK	y8: Phospho (+79.97)	99.70%	1.27	17.2	1.25	3	441.53	1,321.57	-0.00205
1419	PE	PV	3 µM	512	521	ALRADENyyK	y8: Phospho (+79.97), y9: Phospho (+79.97)	99.70%	1.45	18.6	1.62	2	701.78	1,401.54	0.00304
1420	PE	PV	10 µM	512	521	ALRADENyYK	y8: Phospho (+79.97)	99.70%	2.74	23.4	0.82	3	441.53	1,321.57	0.00298
1421	PE	PV	10 µM	512	521	ALRADENYyK	y9: Phospho (+79.97)	99.70%	2.03	0	3.47	2	661.79	1,321.57	0.00159
1422	PE	PV	10 µM	512	521	ALRADENyYK	y8: Phospho (+79.97)	99.70%	3.09	29.1	2.19	2	661.79	1,321.57	0.00269
1423	PE	PV	10 µM	512	521	ALRADENyYK	y8: Phospho (+79.97)	99.70%	2.34	31.1	5.02	2	661.79	1,321.57	0.00135
1424	PE	PV	10 µM	512	521	ALRADENyYK	y8: Phospho (+79.97)	99.70%	2.71	31.5	8.12	2	661.79	1,321.57	0.00172
1425	PE	PV	10 µM	512	521	ALRADENyYK	y8: Phospho (+79.97)	88.10%	1.27	20.2	0	2	661.79	1,321.57	0.00147
1426	PE	PV	10 µM	512	521	ALRADENyyK	y8: Phospho (+79.97), y9: Phospho (+79.97)	98.70%	0.94	11.9	0	3	468.19	1,401.54	-0.00018
1427	PE	PV	10 µM	512	521	ALRADENYyK	y9: Phospho (+79.97)	99.70%	1.35	31.5	2.51	2	661.79	1,321.57	-0.00121
1428	PE	PV	10 µM	512	521	ALRADENyYK	y8: Phospho (+79.97)	99.70%	2.89	37.3	9.66	2	661.79	1,321.57	-0.00390
1429	PE	PV	10 µM	512	521	ALRADENyYK	y8: Phospho (+79.97)	99.70%	3.02	17.3	5.09	3	441.53	1,321.57	-0.00306
1430	PE	PV	10 µM	512	521	ALRADENyyK	y8: Phospho (+79.97), y9: Phospho (+79.97)	99.70%	2.33	25.3	4.03	2	701.78	1,401.54	0.00328
1431	PE	PV	10 µM	512	521	ALRADENyYK	y8: Phospho (+79.97)	99.70%	2.81	26	5.04	2	661.79	1,321.57	0.00111
1432	PE	PV	10 µM	512	521	ALRADENyYK	y8: Phospho (+79.97)	99.70%	2.48	31	5.51	2	661.79	1,321.57	0.00135
1433	PE	PV	10 µM	512	521	ALRADENyyK	y8: Phospho (+79.97), y9: Phospho (+79.97)	99.70%	2.7	33.1	6.48	2	701.78	1,401.54	-0.00087
1434	PE	PV	10 µM	512	521	ALRADENyYK	y8: Phospho (+79.97)	99.70%	1.55	37.5	4.8	2	661.79	1,321.57	0.00208
1435	PE	PV	10 µM	512	521	ALRADENYyK	y9: Phospho (+79.97)	99.70%	1.67	40.5	5.15	2	661.79	1,321.57	0.00184
1436	PE	PV	10 µM	512	521	ALRADENyyK	y8: Phospho (+79.97), y9: Phospho (+79.97)	99.70%	1.87	35.4	5.66	2	701.78	1,401.54	0.00218
1437	PE	PV	10 µM	512	521	ALRADENyYK	y8: Phospho (+79.97)	99.70%	3.13	37.4	9.54	2	661.79	1,321.57	0.00245
1438	PE	PV	10 µM	512	521	ALRADENyYK	y8: Phospho (+79.97)	99.70%	2	22.4	3.14	3	441.53	1,321.57	0.00390
1439	PE	PV	10 µM	512	521	ALRADENyYK	y8: Phospho (+79.97)	99.70%	2.64	28.6	6.16	3	441.53	1,321.58	0.00738
1440	PE	PV	10 µM	512	521	ALRADENyYK	y8: Phospho (+79.97)	99.70%	2.29	23.3	6.04	2	661.79	1,321.57	0.00037
1441	PE	PV	30 µM	512	521	ALRADENyYK	y8: Phospho (+79.97)	99.40%	2.43	22.8	0.44	3	441.53	1,321.57	0.00363
1442	PE	PV	30 µM	512	521	ALRADENYyK	y9: Phospho (+79.97)	99.50%	2.4	19.5	0.85	2	661.79	1,321.57	0.00294
1443	PE	PV	30 µM	512	521	ALRADENyYK	y8: Phospho (+79.97)	99.70%	2.33	12.4	1.28	2	661.79	1,321.57	0.00013
1444	PE	PV	30 µM	512	521	ALRADENyYK	y8: Phospho (+79.97)	99.70%	2.17	0	1.32	3	441.53	1,321.57	0.00189
1445	PE	PV	30 µM	512	521	ALRADENYyK	y9: Phospho (+79.97)	99.70%	2	12.7	2.62	2	661.80	1,321.58	0.00709
1446	PE	PV	30 µM	512	521	ALRADENyYK	y8: Phospho (+79.97)	99.70%	2.2	22.2	2.51	2	661.79	1,321.57	0.00257
1447	PE	PV	30 µM	512	521	ALRADENYyK	y9: Phospho (+79.97)	99.70%	2.37	15.3	2.92	2	661.79	1,321.57	0.00001
1448	PE	PV	30 µM	512	521	ALRADENyYK	y8: Phospho (+79.97)	99.70%	3.06	13.3	1.66	3	441.53	1,321.57	-0.00040
1449	PE	PV	30 µM	512	521	ALRADENyYK	y8: Phospho (+79.97)	99.00%	1.97	19.5	2.06	2	661.79	1,321.57	0.00269

1450	PE	PV	30 µM	512	521	ALRADENyyK	y8: Phospho (+79.97), y9: Phospho (+79.97)	99.60%	1.52	13.8	1.4	3	468.19	1,401.54	-0.00018
1451	PE	PV	30 µM	512	521	ALRADENYyK	y9: Phospho (+79.97)	99.70%	1.6	24.1	2.72	3	441.53	1,321.57	0.00060
1452	PE	PV	30 µM	512	521	ALRADENYyK	y9: Phospho (+79.97)	99.70%	3.01	29	5	2	661.79	1,321.57	-0.00109
1453	PE	PV	30 µM	512	521	ALRADENyYK	y8: Phospho (+79.97)	99.70%	2.4	29.4	5.44	2	661.79	1,321.57	-0.00036
1454	PE	PV	30 µM	512	521	ALRADENYyK	y9: Phospho (+79.97)	99.70%	3.08	29.5	6.21	2	661.79	1,321.57	-0.00158
1455	PE	PV	30 µM	512	521	ALRADENyYK	y8: Phospho (+79.97)	99.70%	2.75	27.6	7.8	2	661.79	1,321.57	0.00404
1456	PE	PV	30 µM	512	521	ALRADENyyK	y8: Phospho (+79.97), y9: Phospho (+79.97)	99.70%	2.97	26.7	5.4	2	701.78	1,401.54	-0.00063
1457	PE	PV	30 µM	512	521	ALRADENyYK	y8: Phospho (+79.97)	99.70%	3.19	33.5	7.51	2	661.79	1,321.57	-0.00195
1458	PE	PV	30 µM	512	521	ALRADENyyK	y8: Phospho (+79.97), y9: Phospho (+79.97)	99.70%	3.29	32.4	10.7	2	701.77	1,401.53	-0.00368
1459	PE	PV	30 µM	512	521	ALRADENyyK	y8: Phospho (+79.97), y9: Phospho (+79.97)	99.70%	3.32	27.7	5.51	2	701.78	1,401.54	-0.00014
1460	PE	PV	30 µM	512	521	ALRADENyyK	y8: Phospho (+79.97), y9: Phospho (+79.97)	82.40%	0.96	10.1	0	3	468.19	1,401.54	0.00064
1461	PE	PV	30 µM	512	521	ALRADENyyK	y8: Phospho (+79.97), y9: Phospho (+79.97)	99.70%	1.19	16.4	2.6	2	701.78	1,401.54	0.00108
1462	PE	PV	30 µM	512	521	ALRADENyyK	y8: Phospho (+79.97), y9: Phospho (+79.97)	99.70%	1.34	17.9	1.33	3	468.19	1,401.54	-0.00055
1463	PE	PV	30 µM	512	521	ALRADENYyK	y9: Phospho (+79.97)	99.70%	1.41	30.7	3.92	2	661.79	1,321.57	0.00098
1464	PE	PV	30 µM	512	521	ALRADENyyK	y8: Phospho (+79.97), y9: Phospho (+79.97)	99.70%	1.8	23.4	6	2	701.78	1,401.54	0.00121
1465	PE	PV	30 µM	512	521	ALRADENyYK	y8: Phospho (+79.97)	99.70%	2.39	42.3	10.6	2	661.79	1,321.57	-0.00329
1466	PE	PV	30 µM	512	521	ALRADENYyK	y9: Phospho (+79.97)	99.70%	2.01	23.2	3.51	3	441.53	1,321.57	0.00033
1467	PE	PV	30 µM	512	521	ALRADENyYK	y8: Phospho (+79.97)	99.70%	2.88	21.6	5.48	3	441.53	1,321.57	-0.00233
1468	PE	PV	30 µM	512	521	ALRADENyyK	y8: Phospho (+79.97), y9: Phospho (+79.97)	99.70%	2.77	20.2	4.33	2	701.78	1,401.54	-0.00014
1469	PE	PV	30 µM	512	521	ALRADENyYK	y8: Phospho (+79.97)	99.70%	2.89	24.5	4.85	2	661.79	1,321.57	0.00123
1470	PE	PV	30 µM	512	521	ALRADENyYK	y8: Phospho (+79.97)	99.70%	3.31	31.4	4.82	2	661.79	1,321.57	0.00159
1471	PE	PV	30 µM	512	521	ALRADENyYK	y8: Phospho (+79.97)	99.70%	3.32	15.2	3.96	3	441.53	1,321.57	0.00125
1472	PE	PV	30 µM	512	521	ALRADENyyK	y8: Phospho (+79.97), y9: Phospho (+79.97)	99.70%	1.11	21.2	1.96	2	701.78	1,401.54	0.00377
1473	PE	PV	30 µM	512	521	ALRADENyYK	y8: Phospho (+79.97)	99.70%	1.57	30.8	5.68	2	661.79	1,321.57	0.00281
1474	PE	PV	30 µM	512	521	ALRADENyYK	y8: Phospho (+79.97)	99.70%	2.09	33.1	8.03	2	661.79	1,321.57	0.00330
1475	PE	PV	30 µM	512	521	ALRADENyyK	y8: Phospho (+79.97), y9: Phospho (+79.97)	99.70%	2.04	36	9.24	2	701.78	1,401.54	0.00645
1476	PE	PV	30 µM	512	521	ALRADENyYK	y8: Phospho (+79.97)	99.70%	2.2	51.8	7.68	2	661.79	1,321.57	0.00123
1477	PE	PV	30 µM	512	521	ALRADENYyK	y9: Phospho (+79.97)	99.70%	2.9	40.8	10.4	2	661.79	1,321.57	0.00294
1478	PE	PV	30 µM	512	521	ALRADENYyK	y9: Phospho (+79.97)	99.70%	2.31	12.7	3.19	3	441.53	1,321.57	0.00216
1479	PE	PV	30 µM	512	521	ALRADENyYK	y8: Phospho (+79.97)	99.70%	2.35	19.1	4.85	3	441.53	1,321.57	0.00436
1480	PE	PV	30 µM	512	521	ALRADENyyK	y8: Phospho (+79.97), y9: Phospho (+79.97)	99.70%	2.81	22.7	6.46	3	468.19	1,401.54	0.00256
1481	PE	PV	30 µM	512	521	ALRADENyyK	y8: Phospho (+79.97), y9: Phospho (+79.97)	99.70%	2.15	18.4	2.74	2	701.78	1,401.56	0.01842

1482	PE	PV	30 μM	512	521	ALRADENyYK	y8: Phospho (+79.97)	99.70%	1.97	20.5	3.19	2	661.80	1,321.59	0.01966
1483	PE	PV	30 μM	512	521	ALRADENyyK	y8: Phospho (+79.97), y9: Phospho (+79.97)	99.70%	2.14	35	5.42	2	701.79	1,401.56	0.02025
1484	PE	PV	30 μM	512	521	ALRADENYyK	y9: Phospho (+79.97)	99.70%	1.52	22.1	1.23	2	661.79	1,321.57	0.00086
1485	PE	PV	30 μM	512	521	ALRADENyYK	y8: Phospho (+79.97)	99.70%	1.68	37.3	6.72	2	661.79	1,321.57	0.00379
1486	PE	PV	30 μM	512	521	ALRADENyYK	y8: Phospho (+79.97)	99.70%	2.31	12.5	4.77	3	441.53	1,321.57	0.00427
1487	PE	PV	100 μM	512	521	ALRADENyYK	y8: Phospho (+79.97)	82.00%	1.73	14.8	0	2	661.79	1,321.57	0.00208
1488	PE	PV	100 μM	512	521	ALRADENYyK	y9: Phospho (+79.97)	98.70%	2.29	14.4	0	3	441.53	1,321.57	0.00207
1489	PE	PV	100 μM	512	521	ALRADENYyK	y9: Phospho (+79.97)	99.70%	2.82	0	0.92	3	441.53	1,321.57	0.00189
1490	PE	PV	100 μM	512	521	ALRADENYyK	y9: Phospho (+79.97)	99.70%	1.96	20.1	2.01	2	661.79	1,321.57	0.00233
1491	PE	PV	100 μM	512	521	ALRADENyYK	y8: Phospho (+79.97)	99.70%	2.39	15.9	1.49	3	441.53	1,321.57	0.00005
1492	PE	PV	100 μM	512	521	ALRADENyYK	y8: Phospho (+79.97)	99.70%	2.48	33.7	3.44	2	661.79	1,321.57	0.00074
1493	PE	PV	100 μM	512	521	ALRADENyYK	y8: Phospho (+79.97)	99.70%	2.63	30.1	5.89	2	661.79	1,321.57	0.00386
1494	PE	PV	100 μM	512	521	ALRADENyYK	y8: Phospho (+79.97)	99.70%	2.61	36.1	6.18	2	661.79	1,321.57	0.00326
1495	PE	PV	100 μM	512	521	ALRADENYyK	y9: Phospho (+79.97)	99.70%	1.94	20.1	4.66	3	441.53	1,321.57	0.00033
1496	PE	PV	100 μM	512	521	ALRADENYyK	y9: Phospho (+79.97)	99.70%	2.74	31.6	6.32	2	661.79	1,321.57	0.00050
1497	PE	PV	100 μM	512	521	ALRADENyYK	y8: Phospho (+79.97)	99.70%	2.86	36	9.96	2	661.79	1,321.57	0.00276
1498	PE	PV	100 μM	512	521	ALRADENyyK	y8: Phospho (+79.97), y9: Phospho (+79.97)	85.70%	0.54	0	0	2	701.78	1,401.54	-0.00002
1499	PE	PV	100 μM	512	521	ALRADENYyK	y9: Phospho (+79.97)	99.70%	1.08	20	1.19	2	661.79	1,321.57	-0.00024
1500	PE	PV	100 μM	512	521	ALRADENyyK	y8: Phospho (+79.97), y9: Phospho (+79.97)	99.70%	1.69	46	5.32	2	701.78	1,401.54	0.00023
1501	PE	PV	100 μM	512	521	ALRADENyyK	y8: Phospho (+79.97), y9: Phospho (+79.97)	99.70%	1.41	13.4	0	3	468.19	1,401.54	0.00110
1502	PE	PV	100 μM	512	521	ALRADENYyK	y9: Phospho (+79.97)	99.70%	1.96	42.5	5.48	2	661.79	1,321.57	-0.00341
1503	PE	PV	100 μM	512	521	ALRADENyYK	y8: Phospho (+79.97)	99.70%	2.77	20.6	5.02	3	441.53	1,321.57	-0.00352
1504	PE	PV	100 μM	512	521	ALRADENYyK	y9: Phospho (+79.97)	99.70%	2.7	14.7	4.31	3	441.53	1,321.57	0.00152
1505	PE	PV	100 μM	512	521	ALRADENyYK	y8: Phospho (+79.97)	99.70%	2.88	19.3	5.11	3	441.53	1,321.57	0.00152
1506	PE	PV	100 μM	512	521	ALRADENYyK	y9: Phospho (+79.97)	99.70%	3.1	31.6	5.44	2	661.79	1,321.57	0.00098
1507	PE	PV	100 μM	512	521	ALRADENyyK	y8: Phospho (+79.97), y9: Phospho (+79.97)	99.70%	3.1	28	7.05	2	701.78	1,401.54	0.00218
1508	PE	PV	100 μM	512	521	ALRADENyYK	y8: Phospho (+79.97)	99.10%	0	12.6	0.8	2	661.79	1,321.57	0.00440
1509	PE	PV	100 μM	512	521	ALRADENyyK	y8: Phospho (+79.97), y9: Phospho (+79.97)	99.70%	0.97	15.6	1.15	2	701.78	1,401.54	0.00413
1510	PE	PV	100 μM	512	521	ALRADENyYK	y8: Phospho (+79.97)	99.70%	1.47	26	2.51	2	661.79	1,321.57	0.00294
1511	PE	PV	100 μM	512	521	ALRADENYyK	y9: Phospho (+79.97)	99.70%	1.49	12.3	4.38	3	441.53	1,321.57	0.00280
1512	PE	PV	100 μM	512	521	ALRADENyyK	y8: Phospho (+79.97), y9: Phospho (+79.97)	99.70%	1.75	26.3	1.74	2	701.78	1,401.54	0.00462
1513	PE	PV	100 μM	512	521	ALRADENYyK	y9: Phospho (+79.97)	99.70%	2.15	43.7	6.18	2	661.79	1,321.57	-0.00109
1514	PE	PV	100 μM	512	521	ALRADENyyK	y8: Phospho (+79.97), y9: Phospho (+79.97)	99.70%	1.79	0	2.66	3	468.19	1,401.54	0.00421
1515	PE	PV	100 μM	512	521	ALRADENyyK	y8: Phospho (+79.97), y9: Phospho (+79.97)	99.70%	2.53	44.1	10.2	2	701.78	1,401.54	0.00377
1516	PE	PV	100 μM	512	521	ALRADENyYK	y8: Phospho (+79.97)	99.70%	3.32	35.2	13.9	2	661.79	1,321.58	0.00477

1517	PE	PV	100 µM	512	521	ALRADENyyK	y8: Phospho (+79.97), y9: Phospho (+79.97)	99.70%	1.95	17.5	2.89	2	701.78	1,401.55	0.01695
1518	PE	PV	100 µM	512	521	ALRADENyyK	y8: Phospho (+79.97), y9: Phospho (+79.97)	99.70%	1.78	18.5	4.38	2	701.79	1,401.56	0.02074
1519	PE	PV	100 µM	512	521	ALRADENyyK	y8: Phospho (+79.97), y9: Phospho (+79.97)	99.70%	2.07	28.9	4.47	2	701.79	1,401.56	0.01988
1520	PE	PV	100 µM	512	521	ALRADENYyK	y9: Phospho (+79.97)	99.70%	1.48	31.5	2.68	2	661.79	1,321.57	-0.00427
1521	PE	PV	100 µM	512	521	ALRADENYyK	y9: Phospho (+79.97)	99.70%	1.97	39.2	3.72	2	661.79	1,321.57	-0.00036
1522	PE	PV	100 µM	512	521	ALRADENyYK	y8: Phospho (+79.97)	99.70%	2.27	0	4.55	3	441.53	1,321.57	0.00088
1523	IP	PV	0	512	527	ALRADENYyKAQTHGK	y9: Phospho (+79.97)	99.30%	3.34	18.1	2.68	3	648.97	1,943.89	0.00273
1524	PE	PV	10 µM	512	527	ALRADENyyKAQTHGK	y8: Phospho (+79.97), y9: Phospho (+79.97)	99.70%	2.45	35.3	3.46	4	506.97	2,023.86	0.00049
1525	PE	PV	10 µM	512	527	ALRADENyyKAQTHGK	y8: Phospho (+79.97), y9: Phospho (+79.97)	99.70%	2.64	33.6	6.16	3	675.63	2,023.85	-0.00145
1526	PE	PV	30 µM	512	527	ALRADENyyKAQTHGK	y8: Phospho (+79.97), y9: Phospho (+79.97)	99.70%	2.66	21.7	4.37	3	675.63	2,023.85	-0.00181
1527	PE	PV	100 µM	512	527	ALRADENyyKAQTHGK	y8: Phospho (+79.97), y9: Phospho (+79.97)	99.70%	3.13	26.6	5.66	3	675.63	2,023.86	0.00002
1528	PE	Hg	100 µM	515	521	ADENyYK	y5: Phospho (+79.97)	98.60%	1.04	15.9	1.19	2	491.68	981.352	0.00368
1529	PE	PV	30 µM	515	521	ADENyYK	y5: Phospho (+79.97)	98.80%	1.02	27.1	0.51	2	491.68	981.349	0.00136
1530	PE	PV	30 µM	515	521	ADENyYK	y5: Phospho (+79.97)	98.80%	1.45	13.4	1.46	2	491.68	981.349	0.00087
1531	PE	PV	30 µM	515	521	ADENYyK	y6: Phospho (+79.97)	98.80%	1.5	22.1	3.82	2	491.68	981.349	0.00051
1532	PE	PV	100 µM	515	521	ADENYyK	y6: Phospho (+79.97)	98.30%	2.15	10.1	0.55	2	491.68	981.351	0.00270
1533	PE	PV	100 µM	515	521	ADENYyK	y6: Phospho (+79.97)	98.80%	1.58	44.8	3.24	2	491.68	981.348	0.00032
1534	PE	PV	100 µM	515	521	ADENYyK	y6: Phospho (+79.97)	98.80%	1.64	23.2	4.64	2	491.68	981.349	0.00105
1535	PE	PV	30 µM	515	527	ADENYyKAQTHGK	y6: Phospho (+79.97)	99.70%	2.09	20.1	5.11	3	535.56	1,603.67	-0.00160
1536	PE	PV	100 µM	515	527	ADENYyKAQTHGK	y6: Phospho (+79.97)	99.70%	3.01	38.7	5.43	3	535.56	1,603.66	-0.00416
1537	PE	Ig	NT	515	531	ADENyyKAQTHGKWPVK	y5: Phospho (+79.97), y6: Phospho (+79.97)	99.70%	2.73	47.1	0	3	732.32	2,193.93	0.00415
1538	PE	Ig	1 min	515	531	ADENyyKAQTHGKWPVK	y5: Phospho (+79.97), y6: Phospho (+79.97)	99.70%	3.15	39.3	0	3	732.32	2,193.94	0.00671
1539	PE	Ig	2 min	515	531	ADENyyKAQTHGKWPVK	y5: Phospho (+79.97), y6: Phospho (+79.97)	86.40%	1.69	0	0	4	549.49	2,193.94	0.00847
1540	PE	Ig	2 min	515	531	ADENyyKAQTHGKWPVK	y5: Phospho (+79.97), y6: Phospho (+79.97)	99.70%	2.88	52.4	0	3	732.32	2,193.93	0.00378
1541	PE	Ig	3 min	515	531	ADENyyKAQTHGKWPVK	y5: Phospho (+79.97), y6: Phospho (+79.97)	99.70%	3.38	35.9	0	3	732.32	2,193.93	0.00195
1542	PE	Ig	5 min	515	531	ADENyyKAQTHGKWPVK	y5: Phospho (+79.97), y6: Phospho (+79.97)	99.70%	1.02	28.7	0	3	732.32	2,193.93	0.00360
1543	PE	Ig	15 min	515	531	ADENyyKAQTHGKWPVK	y5: Phospho (+79.97), y6: Phospho (+79.97)	99.70%	1.87	30.3	0	3	732.32	2,193.93	0.00543
1544	PE	Hg	0	515	531	ADENyyKAQTHGKWPVK	y5: Phospho (+79.97), y6: Phospho (+79.97)	99.70%	1.51	37.5	2.59	3	732.32	2,193.93	-0.00043
1545	PE	Hg	1 µM	515	531	ADENyyKAQTHGKWPVK	y5: Phospho (+79.97), y6: Phospho (+79.97)	99.70%	1.82	48.4	1.74	3	732.32	2,193.93	0.00616

1546	PE	Hg	5 µM	515	531	ADENyyKAQTHGKWPVK	y5: Phospho (+79.97), y6: Phospho (+79.97)	99.70%	2.6	47	5.15	3	732.32	2,193.93	0.00525
1547	PE	Hg	10 µM	515	531	ADENyyKAQTHGKWPVK	y5: Phospho (+79.97), y6: Phospho (+79.97)	99.70%	1.91	27.8	0.96	3	732.32	2,193.93	0.00598
1548	PE	Hg	20 µM	515	531	ADENyYKAQtHGKWPVK	y5: Phospho (+79.97), t10: Phospho (+79.97)	99.70%	1.15	29.2	0.68	3	732.32	2,193.94	0.00635
1549	PE	Hg	50 µM	515	531	ADENyyKAQTHGKWPVK	y5: Phospho (+79.97), y6: Phospho (+79.97)	99.70%	1.56	18.6	0.8	3	732.32	2,193.94	0.00909
1550	PE	Hg	100 µM	515	531	ADENyyKAQTHGKWPVK	y5: Phospho (+79.97), y6: Phospho (+79.97)	99.70%	2.22	44.8	5.54	3	732.32	2,193.94	0.00690
1551	PE	PV	0	515	531	ADENyyKAQTHGKWPVK	y5: Phospho (+79.97), y6: Phospho (+79.97)	98.30%	1.68	0	0	4	549.49	2,193.93	0.00456
1552	PE	PV	1 µM	515	531	ADENyyKAQTHGKWPVK	y5: Phospho (+79.97), y6: Phospho (+79.97)	99.70%	2.88	32.5	7.8	3	732.32	2,193.93	0.00195
1553	PE	PV	3 µM	515	531	ADENyyKAQTHGKWPVK	y5: Phospho (+79.97), y6: Phospho (+79.97)	99.40%	1.33	12.4	0	4	549.49	2,193.94	0.00774
1554	PE	PV	3 µM	515	531	ADENyyKAQTHGKWPVK	y5: Phospho (+79.97), y6: Phospho (+79.97)	99.70%	1.51	19.8	1.15	3	732.32	2,193.93	0.00323
1555	PE	PV	10 µM	515	531	ADENyyKAQTHGKWPVK	y5: Phospho (+79.97), y6: Phospho (+79.97)	98.70%	1.02	24.2	0	3	732.32	2,193.93	0.00598
1556	PE	PV	10 µM	515	531	ADENyyKAQTHGKWPVK	y5: Phospho (+79.97), y6: Phospho (+79.97)	99.70%	1.93	38.2	6.39	3	732.32	2,193.93	0.00543
1557	PE	PV	10 µM	515	531	ADENYYKAQtHGKWPVK	t10: Phospho (+79.97)	99.70%	2.52	34.3	8.27	3	705.66	2,113.97	0.00393
1558	PE	PV	10 µM	515	531	ADENyyKAQTHGKWPVK	y5: Phospho (+79.97), y6: Phospho (+79.97)	99.70%	2.76	24.6	5.8	4	549.49	2,193.93	0.00554
1559	PE	PV	10 µM	515	531	ADENyyKAQTHGKWPVK	y5: Phospho (+79.97), y6: Phospho (+79.97)	99.70%	3.14	33.6	2.89	3	732.32	2,193.93	0.00250
1560	PE	PV	30 µM	515	531	ADENYyKAQtHGKWPVK	y6: Phospho (+79.97), t10: Phospho (+79.97)	97.90%	0	18.6	0.6	3	732.32	2,193.93	0.00140
1561	PE	PV	30 µM	515	531	ADENyyKAQTHGKWPVK	y5: Phospho (+79.97), y6: Phospho (+79.97)	99.70%	0	30.9	4.62	3	732.32	2,193.93	0.00030
1562	PE	PV	30 µM	515	531	ADENyyKAQTHGKWPVK	y5: Phospho (+79.97), y6: Phospho (+79.97)	82.10%	0.77	0	0	3	732.32	2,193.93	0.00030
1563	PE	PV	30 µM	515	531	ADENyyKAQTHGKWPVK	y5: Phospho (+79.97), y6: Phospho (+79.97)	99.10%	1.25	21.6	0	3	732.32	2,193.93	0.00580
1564	PE	PV	30 µM	515	531	ADENyyKAQTHGKWPVK	y5: Phospho (+79.97), y6: Phospho (+79.97)	99.70%	1.33	21.4	0	4	549.49	2,193.93	0.00505
1565	PE	PV	30 µM	515	531	ADENyyKAQTHGKWPVK	y5: Phospho (+79.97), y6: Phospho (+79.97)	99.70%	2.23	31.8	5.3	3	732.32	2,193.94	0.00909
1566	PE	PV	30 µM	515	531	ADENyyKAQTHGKWPVK	y5: Phospho (+79.97), y6: Phospho (+79.97)	99.70%	3.52	23.6	4.82	4	549.49	2,193.93	0.00554
1567	PE	PV	30 µM	515	531	ADENyyKAQTHGKWPVK	y5: Phospho (+79.97), y6: Phospho (+79.97)	99.70%	2.91	46.5	5.01	3	732.32	2,193.93	0.00378
1568	PE	PV	30 µM	515	531	ADENyyKAQTHGKWPVK	y5: Phospho (+79.97), y6: Phospho (+79.97)	99.70%	2.81	34.3	5.14	3	732.33	2,193.96	0.03491

1569	PE	PV	30 µM	515	531	ADENyyKAQTHGKWPVK	y5: Phospho (+79.97), y6: Phospho (+79.97)	99.70%	2.96	30.5	5.68	3	732.33	2,193.97	0.03802
1570	PE	PV	30 µM	515	531	ADENyyKAQTHGKWPVK	y5: Phospho (+79.97), y6: Phospho (+79.97)	99.70%	3	34.8	5.59	3	732.33	2,193.96	0.03052
1571	PE	PV	30 µM	515	531	ADENyyKAQTHGKWPVK	y5: Phospho (+79.97), y6: Phospho (+79.97)	99.70%	2.33	56.5	6.47	3	732.33	2,193.96	0.02704
1572	PE	PV	30 µM	515	531	ADENyyKAQTHGKWPVK	y5: Phospho (+79.97), y6: Phospho (+79.97)	98.20%	0.94	13.2	0	3	732.32	2,193.93	0.00269
1573	PE	PV	30 µM	515	531	ADENyYKAQtHGKWPVK	y5: Phospho (+79.97), t10: Phospho (+79.97)	99.60%	0.74	15.4	0	3	732.32	2,193.93	0.00378
1574	PE	PV	100 µM	515	531	ADENyyKAQTHGKWPVK	y5: Phospho (+79.97), y6: Phospho (+79.97)	99.70%	0	19.8	2.06	3	732.32	2,193.93	0.00232
1575	PE	PV	100 µM	515	531	ADENyYKAQtHGKWPVK	y5: Phospho (+79.97), t10: Phospho (+79.97)	99.70%	0.76	23	0	3	732.32	2,193.93	-0.00189
1576	PE	PV	100 µM	515	531	ADENyyKAQTHGKWPVK	y5: Phospho (+79.97), y6: Phospho (+79.97)	99.00%	1.03	26.1	0	3	732.32	2,193.94	0.00726
1577	PE	PV	100 µM	515	531	ADENyYKAQtHGKWPVK	y5: Phospho (+79.97), t10: Phospho (+79.97)	99.40%	1.03	19.6	0	3	732.32	2,193.93	0.00287
1578	PE	PV	100 µM	515	531	ADENYyKAQTHGKWPVK	y6: Phospho (+79.97)	99.70%	1.43	26.3	3.06	3	705.66	2,113.96	0.00246
1579	PE	PV	100 µM	515	531	ADENyyKAQTHGKWPVK	y5: Phospho (+79.97), y6: Phospho (+79.97)	99.70%	2.51	39	6.07	3	732.32	2,193.94	0.00671
1580	PE	PV	100 µM	515	531	ADENyyKAQTHGKWPVK	y5: Phospho (+79.97), y6: Phospho (+79.97)	99.70%	2.63	30	3.35	4	549.49	2,193.93	0.00481
1581	PE	PV	100 µM	515	531	ADENyyKAQTHGKWPVK	y5: Phospho (+79.97), y6: Phospho (+79.97)	99.70%	3.63	45.8	9.82	3	732.32	2,193.93	0.00525
1582	PE	PV	100 µM	515	531	ADENyyKAQTHGKWPVK	y5: Phospho (+79.97), y6: Phospho (+79.97)	99.70%	3.08	56.1	3.29	3	732.33	2,193.96	0.03564
1583	PE	PV	100 µM	515	531	ADENyyKAQTHGKWPVK	y5: Phospho (+79.97), y6: Phospho (+79.97)	99.70%	3.36	28.3	4.57	3	732.33	2,193.96	0.02869
1584	PE	PV	100 µM	515	531	ADENyyKAQTHGKWPVK	y5: Phospho (+79.97), y6: Phospho (+79.97)	99.70%	3.11	48.6	6.64	3	732.33	2,193.96	0.03418
1585	PE	PV	100 µM	515	531	ADENyyKAQTHGKWPVK	y5: Phospho (+79.97), y6: Phospho (+79.97)	99.40%	0.9	15	0	3	732.32	2,193.93	0.00104
1586	PE	PV	100 µM	515	531	ADENyYKAQTHGKWPVK	y5: Phospho (+79.97)	99.50%	0.81	10.5	0.64	3	705.66	2,113.96	0.00027
1587	PE	PV	100 µM	515	531	ADENYYKAQtHGKWPVK	t10: Phospho (+79.97)	99.70%	0.88	29	1.68	3	705.66	2,113.96	-0.00102
1588	PE	PV	100 µM	515	531	ADENyyKAQTHGKWPVK	y5: Phospho (+79.97), y6: Phospho (+79.97)	99.70%	2.1	16.3	3.31	3	732.32	2,193.93	-0.00024
1589	PE	Ig	NT	620	629	LRNYyYDVVN	y5: Phospho (+79.97)	98.00%	1.29	28.3	0	2	699.81	1,397.61	0.00531
1590	PE	Ig	1 min	620	629	LRNYyYDVVN	y5: Phospho (+79.97)	99.70%	1.14	12	3	2	699.81	1,397.60	0.00079
1591	PE	Ig	1 min	620	629	LRNYyYDVVN	y5: Phospho (+79.97)	99.70%	1.7	26.2	2.51	2	699.81	1,397.60	0.00140
1592	PE	Ig	1 min	620	629	LRNYyYDVVN	y5: Phospho (+79.97)	99.70%	2.43	48.1	0	2	699.81	1,397.61	0.00604
1593	PE	Ig	1 min	620	629	LRNYyYDVVN	y5: Phospho (+79.97)	99.70%	1.54	21.2	2.62	2	699.82	1,397.62	0.01825
1594	PE	Ig	2 min	620	629	LRNYYyDVVN	y6: Phospho (+79.97)	99.10%	1.31	24.4	2.29	2	699.81	1,397.61	0.00348
1595	PE	Ig	2 min	620	629	LRNYyYDVVN	y5: Phospho (+79.97)	99.60%	0.89	17.9	0.43	2	699.81	1,397.61	0.00494
1596	PE	Ig	2 min	620	629	LRNYyYDVVN	y5: Phospho (+79.97)	99.70%	1.71	11.9	2.89	2	699.81	1,397.60	0.00250

1597	PE	Ig	2 min	620	629	LRNYyYDVVN	y5: Phospho (+79.97)	99.70%	1.72	31.6	2.08	2	699.81	1,397.60	0.00286
1598	PE	Ig	2 min	620	629	LRNYyYDVVN	y5: Phospho (+79.97)	99.70%	2.17	35.5	4.02	2	699.81	1,397.60	0.00140
1599	PE	Ig	2 min	620	629	LRNYYyDVVN	y6: Phospho (+79.97)	97.60%	0.5	21.8	0	2	699.81	1,397.61	0.00775
1600	PE	Ig	2 min	620	629	LRNYyYDVVN	y5: Phospho (+79.97)	99.70%	2.24	47.2	0	2	699.81	1,397.61	0.00592
1601	PE	Ig	3 min	620	629	LRNYyYDVVN	y5: Phospho (+79.97)	98.60%	1.52	24.5	2.16	2	699.81	1,397.61	0.00567
1602	PE	Ig	3 min	620	629	LRNYyYDVVN	y5: Phospho (+79.97)	99.60%	1.31	15.9	2.12	2	699.81	1,397.60	0.00274
1603	PE	Ig	3 min	620	629	LRNYYyDVVN	y6: Phospho (+79.97)	99.70%	1.26	12.6	4	2	699.81	1,397.61	0.00409
1604	PE	Ig	3 min	620	629	LRNYyYDVVN	y5: Phospho (+79.97)	99.70%	2.21	31.9	3.44	2	699.81	1,397.60	0.00201
1605	PE	Ig	3 min	620	629	LRNYyYDVVN	y5: Phospho (+79.97)	99.70%	1.5	29.1	0	2	699.81	1,397.60	-0.00080
1606	PE	Ig	3 min	620	629	LRNYyYDVVN	y5: Phospho (+79.97)	99.70%	2.23	23.5	4.11	2	699.81	1,397.60	-0.00043
1607	PE	Ig	3 min	620	629	LRNyYYDVVN	y4: Phospho (+79.97)	85.20%	0.5	0	0	2	699.81	1,397.60	0.00335
1608	PE	Ig	3 min	620	629	LRNYyYDVVN	y5: Phospho (+79.97)	99.70%	2.22	44.6	0	2	699.81	1,397.61	0.00604
1609	PE	Ig	3 min	620	629	LRNYyYDVVN	y5: Phospho (+79.97)	99.70%	1.89	24.7	3.39	2	699.82	1,397.62	0.02044
1610	PE	Ig	5 min	620	629	LRNYyYDVVN	y5: Phospho (+79.97)	97.00%	1.39	10.4	0	2	699.81	1,397.60	0.00189
1611	PE	Ig	5 min	620	629	LRNYyYDVVN	y5: Phospho (+79.97)	99.70%	1.92	23.2	2.92	2	699.81	1,397.61	0.00396
1612	PE	Ig	5 min	620	629	LRNYyYDVVN	y5: Phospho (+79.97)	99.70%	1.84	15.2	3.47	2	699.81	1,397.60	0.00250
1613	PE	Ig	5 min	620	629	LRNYyYDVVN	y5: Phospho (+79.97)	99.70%	2.6	35.1	4.46	2	699.81	1,397.60	0.00274
1614	PE	Ig	5 min	620	629	LRNYyYDVVN	y5: Phospho (+79.97)	83.50%	0.5	0	0	2	699.81	1,397.60	0.00018
1615	PE	Ig	5 min	620	629	LRNYyYDVVN	y5: Phospho (+79.97)	94.60%	0.75	25.6	0	2	699.81	1,397.61	0.00531
1616	PE	Ig	5 min	620	629	LRNYyYDVVN	y5: Phospho (+79.97)	99.70%	2.39	34.7	4.01	2	699.81	1,397.60	0.00238
1617	PE	Ig	5 min	620	629	LRNYYyDVVN	y6: Phospho (+79.97)	99.70%	1.47	32.4	0	2	699.81	1,397.61	0.00689
1618	PE	Ig	5 min	620	629	LRNYyYDVVN	y5: Phospho (+79.97)	99.70%	2.67	44.7	0	2	699.81	1,397.61	0.00518
1619	PE	Ig	5 min	620	629	LRNyYYDVVN	y4: Phospho (+79.97)	99.70%	1.16	12	2.11	2	699.82	1,397.62	0.01788
1620	PE	Ig	5 min	620	629	LRNYyYDVVN	y5: Phospho (+79.97)	99.70%	1.58	16.3	3.1	2	699.82	1,397.62	0.01934
1621	PE	Ig	5 min	620	629	LRNYYyDVVN	y6: Phospho (+79.97)	98.90%	0.98	31.2	0	2	699.81	1,397.60	0.00042
1622	PE	Ig	15 min	620	629	LRNYyYDVVN	y5: Phospho (+79.97)	87.30%	1.3	0	2.68	2	699.81	1,397.60	0.00238
1623	PE	Ig	15 min	620	629	LRNYyYDVVN	y5: Phospho (+79.97)	99.40%	1.83	12.9	2.13	2	699.81	1,397.60	0.00311
1624	PE	Ig	15 min	620	629	LRNYyYDVVN	y5: Phospho (+79.97)	99.70%	1.73	29.1	0	2	699.81	1,397.60	-0.00116
1625	PE	Ig	15 min	620	629	LRNYyYDVVN	y5: Phospho (+79.97)	99.70%	1.34	24.5	3.42	2	699.81	1,397.60	0.00213
1626	PE	Ig	15 min	620	629	LRNYyYDVVN	y5: Phospho (+79.97)	99.70%	1.94	42.1	4.85	2	699.81	1,397.60	0.00030
1627	PE	Ig	15 min	620	629	LRNYyYDVVN	y5: Phospho (+79.97)	97.80%	1.46	25.8	0	2	699.81	1,397.61	0.00653
1628	PE	Ig	15 min	620	629	LRNYyYDVVN	y5: Phospho (+79.97)	99.70%	2.59	38.4	0	2	699.81	1,397.61	0.00616
1629	PE	Hg	0	620	629	LRNYyYDVVN	y5: Phospho (+79.97)	99.70%	1.31	17.3	0	2	699.81	1,397.60	0.00335
1630	PE	Hg	0	620	629	LRNYyYDVVN	y5: Phospho (+79.97)	99.70%	1.17	26.3	0.62	2	699.81	1,397.61	0.00677
1631	PE	Hg	1 µM	620	629	LRNYyYDVVN	y5: Phospho (+79.97)	99.70%	1.67	28.5	2.96	2	699.81	1,397.60	-0.00263
1632	PE	Hg	1 µM	620	629	LRNYYyDVVN	y6: Phospho (+79.97)	88.20%	0.63	15.3	0	2	699.81	1,397.61	0.00702
1633	PE	Hg	5 µM	620	629	LRNYyYDVVN	y5: Phospho (+79.97)	99.70%	1.54	31.8	0	2	699.81	1,397.60	-0.00104
1634	PE	Hg	5 µM	620	629	LRNYyYDVVN	y5: Phospho (+79.97)	99.70%	1.88	43.5	2.96	2	699.81	1,397.61	0.00738
1635	PE	Hg	10 µM	620	629	LRNYyYDVVN	y5: Phospho (+79.97)	99.70%	2.01	60.5	1.74	2	699.81	1,397.60	-0.00141
1636	PE	Hg	20 µM	620	629	LRNYYyDVVN	y6: Phospho (+79.97)	99.70%	1.51	39.4	2	2	699.81	1,397.61	0.00665
1637	PE	Hg	50 µM	620	629	LRNYyYDVVN	y5: Phospho (+79.97)	99.70%	1.6	43.2	4.3	2	699.81	1,397.61	0.00982
1638	PE	Hg	100 µM	620	629	LRNyYYDVVN	y4: Phospho (+79.97)	92.00%	1.8	12.1	0	2	699.81	1,397.61	0.00665
1639	PE	Hg	100 µM	620	629	LRNYyYDVVN	y5: Phospho (+79.97)	99.70%	1.2	19.1	1.55	2	699.81	1,397.61	0.00702
1640	PE	Hg	100 µM	620	629	LRNYyYDVVN	y5: Phospho (+79.97)	99.70%	1.49	29.1	1.8	2	699.81	1,397.61	0.00775
1641	PE	PV	0	620	629	LRNYYyDVVN	y6: Phospho (+79.97)	99.70%	1.56	73.5	4.27	2	699.81	1,397.61	0.00677

1642	PE	PV	1 µM	620	629	LRNYyYDVVN	y5: Phospho (+79.97)	99.70%	1.43	42.3	1.17	2	699.81	1,397.60	-0.00165
1643	PE	PV	1 µM	620	629	LRNYyYDVVN	y5: Phospho (+79.97)	99.70%	1.8	30.7	1.11	2	699.81	1,397.61	0.00738
1644	PE	PV	3 µM	620	629	LRNyYYDVVN	y4: Phospho (+79.97)	99.70%	1.16	21.9	0	2	699.81	1,397.60	-0.00165
1645	PE	PV	3 µM	620	629	LRNYyYDVVN	y5: Phospho (+79.97)	99.70%	1.8	42.2	3.32	2	699.81	1,397.61	0.00726
1646	PE	PV	10 µM	620	629	LRNYYyDVVN	y6: Phospho (+79.97)	98.80%	1.83	23.3	0	2	699.81	1,397.60	0.00299
1647	PE	PV	10 µM	620	629	LRNYyyDVVN	y5: Phospho (+79.97), y6: Phospho (+79.97)	90.10%	1.11	22.3	1.08	2	739.79	1,477.57	0.00187
1648	PE	PV	10 µM	620	629	LRNYyYDVVN	y5: Phospho (+79.97)	99.70%	1.96	38.1	2.57	2	699.81	1,397.60	0.00189
1649	PE	PV	10 µM	620	629	LRNYYyDVVN	y6: Phospho (+79.97)	99.50%	1.54	20.9	2.51	2	699.81	1,397.60	0.00323
1650	PE	PV	10 µM	620	629	LRNYyYDVVN	y5: Phospho (+79.97)	99.70%	1.16	20.2	3.33	2	699.81	1,397.61	0.00433
1651	PE	PV	10 µM	620	629	LRNYyYDVVN	y5: Phospho (+79.97)	99.70%	2.03	34.2	4.23	2	699.81	1,397.60	0.00164
1652	PE	PV	10 µM	620	629	LRNYyYDVVN	y5: Phospho (+79.97)	99.70%	2.15	35.5	4.72	2	699.81	1,397.60	0.00274
1653	PE	PV	10 µM	620	629	LRNYyYDVVN	y5: Phospho (+79.97)	99.70%	2.23	34.1	4.66	2	699.81	1,397.60	0.00177
1654	PE	PV	10 µM	620	629	LRNYyYDVVN	y5: Phospho (+79.97)	99.70%	1.41	31.5	1.11	2	699.81	1,397.60	0.00299
1655	PE	PV	10 µM	620	629	LRNYyYDVVN	y5: Phospho (+79.97)	99.70%	1.53	27.8	1.42	2	699.81	1,397.60	0.00055
1656	PE	PV	10 µM	620	629	LRNYYyDVVN	y6: Phospho (+79.97)	99.70%	1.63	36	0.89	2	699.81	1,397.60	0.00030
1657	PE	PV	10 µM	620	629	LRNYyYDVVN	y5: Phospho (+79.97)	99.40%	1.48	11.8	1.82	2	699.81	1,397.60	-0.00336
1658	PE	PV	10 µM	620	629	LRNYyYDVVN	y5: Phospho (+79.97)	99.70%	2.69	37.6	3.55	2	699.81	1,397.60	-0.00007
1659	PE	PV	10 µM	620	629	LRNYyYDVVN	y5: Phospho (+79.97)	99.70%	2.2	19.2	4	2	699.81	1,397.60	-0.00092
1660	PE	PV	10 µM	620	629	LRNYyYDVVN	y5: Phospho (+79.97)	99.70%	2.02	36	4.92	2	699.81	1,397.60	0.00103
1661	PE	PV	10 µM	620	629	LRNYyYDVVN	y5: Phospho (+79.97)	99.70%	2.62	39.6	3.85	2	699.81	1,397.60	0.00006
1662	PE	PV	10 µM	620	629	LRNYyYDVVN	y5: Phospho (+79.97)	99.70%	1.83	54.9	4.48	2	699.81	1,397.61	0.00640
1663	PE	PV	10 µM	620	629	LRNYYyDVVN	y6: Phospho (+79.97)	99.70%	1.6	43.9	2.66	2	699.81	1,397.61	0.00640
1664	PE	PV	10 µM	620	629	LRNYyYDVVN	y5: Phospho (+79.97)	99.70%	2.62	52.2	4.68	2	699.81	1,397.61	0.00628
1665	PE	PV	10 µM	620	629	LRNYyYDVVN	y5: Phospho (+79.97)	99.30%	1.86	34.3	2.32	2	699.82	1,397.63	0.02581
1666	PE	PV	10 µM	620	629	LRNYyyDVVN	y5: Phospho (+79.97), y6: Phospho (+79.97)	99.70%	0	13.7	2.22	2	739.80	1,477.59	0.02213
1667	PE	PV	10 µM	620	629	LRNYyYDVVN	y5: Phospho (+79.97)	99.70%	1.44	15.9	2.55	2	699.82	1,397.62	0.02056
1668	PE	PV	10 µM	620	629	LRNYyYDVVN	y5: Phospho (+79.97)	99.70%	1.46	22.5	2.89	2	699.82	1,397.62	0.02252
1669	PE	PV	10 µM	620	629	LRNYyYDVVN	y5: Phospho (+79.97)	99.70%	1.81	34.4	3.1	2	699.82	1,397.62	0.02203
1670	PE	PV	10 µM	620	629	LRNyYYDVVN	y4: Phospho (+79.97)	99.70%	1.25	27.2	3.04	2	699.82	1,397.62	0.02069
1671	PE	PV	10 µM	620	629	LRNYyYDVVN	y5: Phospho (+79.97)	99.70%	1.86	27.8	3.32	2	699.82	1,397.62	0.02154
1672	PE	PV	30 µM	620	629	LRNYyyDVVN	y5: Phospho (+79.97), y6: Phospho (+79.97)	99.20%	1.24	33	2.47	2	739.79	1,477.57	0.00333
1673	PE	PV	30 µM	620	629	LRNYyyDVVN	y5: Phospho (+79.97), y6: Phospho (+79.97)	99.30%	1.66	27.8	2.17	2	739.79	1,477.57	0.00162
1674	PE	PV	30 µM	620	629	LRNYyYDVVN	y5: Phospho (+79.97)	99.70%	1.49	32.2	2.96	2	699.81	1,397.60	0.00225
1675	PE	PV	30 µM	620	629	LRNYyYDVVN	y5: Phospho (+79.97)	99.70%	2.04	33.1	3.15	2	699.81	1,397.60	0.00189
1676	PE	PV	30 µM	620	629	LRNYyYDVVN	y5: Phospho (+79.97)	99.70%	1.99	34.5	4.48	2	699.81	1,397.61	0.00445
1677	PE	PV	30 µM	620	629	LRNYyYDVVN	y5: Phospho (+79.97)	99.70%	2.31	32.1	4.57	2	699.81	1,397.60	0.00323
1678	PE	PV	30 µM	620	629	LRNYYyDVVN	y6: Phospho (+79.97)	99.70%	1.93	28.2	1.31	2	699.81	1,397.60	-0.00104
1679	PE	PV	30 µM	620	629	LRNYyYDVVN	y5: Phospho (+79.97)	99.70%	2.56	43.2	3.62	2	699.81	1,397.60	0.00299
1680	PE	PV	30 µM	620	629	LRNYyYDVVN	y5: Phospho (+79.97)	99.70%	2.72	36	3.31	2	699.81	1,397.60	0.00103
1681	PE	PV	30 µM	620	629	LRNYyYDVVN	y5: Phospho (+79.97)	99.70%	2.3	31.9	3.96	2	699.81	1,397.60	-0.00092
1682	PE	PV	30 µM	620	629	LRNYyYDVVN	y5: Phospho (+79.97)	99.70%	1.8	25.4	4.21	2	699.81	1,397.60	-0.00007

1683	PE	PV	30 µM	620	629	LRNYyYDVVN	y5: Phospho (+79.97)	99.70%	2.87	39	4.72	2	699.81	1,397.60	-0.00007
1684	PE	PV	30 µM	620	629	LRNYyYDVVN	y5: Phospho (+79.97)	99.70%	1.9	35	2.05	2	699.81	1,397.61	0.00677
1685	PE	PV	30 µM	620	629	LRNYYyDVVN	y6: Phospho (+79.97)	99.70%	2.32	35.8	2.08	2	699.81	1,397.60	0.00067
1686	PE	PV	30 µM	620	629	LRNYyYDVVN	y5: Phospho (+79.97)	99.70%	2.48	38.4	3.36	2	699.81	1,397.61	0.00592
1687	PE	PV	30 µM	620	629	LRNYyYDVVN	y5: Phospho (+79.97)	99.70%	1.74	28.1	2.57	2	699.82	1,397.62	0.01971
1688	PE	PV	30 µM	620	629	LRNYyYDVVN	y5: Phospho (+79.97)	99.70%	1.66	42	2.68	2	699.82	1,397.62	0.01812
1689	PE	PV	30 µM	620	629	LRNYyYDVVN	y5: Phospho (+79.97)	99.70%	2.07	16	2.64	2	699.82	1,397.62	0.01666
1690	PE	PV	30 µM	620	629	LRNYyYDVVN	y5: Phospho (+79.97)	99.70%	1.77	29.5	3.28	2	699.82	1,397.62	0.02020
1691	PE	PV	30 µM	620	629	LRNYyYDVVN	y5: Phospho (+79.97)	99.70%	1.92	31.2	4.09	2	699.82	1,397.62	0.01934
1692	PE	PV	30 µM	620	629	LRNYyYDVVN	y5: Phospho (+79.97)	99.60%	1.45	23.9	0.77	2	699.81	1,397.60	0.00238
1693	PE	PV	100 µM	620	629	LRNYYyDVVN	y6: Phospho (+79.97)	95.60%	1.61	12.2	0	2	699.81	1,397.60	0.00055
1694	PE	PV	100 µM	620	629	LRNYyYDVVN	y5: Phospho (+79.97)	99.50%	0.68	14.2	1.26	2	699.81	1,397.60	0.00299
1695	PE	PV	100 µM	620	629	LRNYyYDVVN	y5: Phospho (+79.97)	99.70%	1.54	13	2.54	2	699.81	1,397.60	0.00164
1696	PE	PV	100 µM	620	629	LRNYyYDVVN	y5: Phospho (+79.97)	99.70%	2.15	32.3	4.28	2	699.81	1,397.61	0.00409
1697	PE	PV	100 µM	620	629	LRNYyYDVVN	y5: Phospho (+79.97)	99.70%	2.04	22.7	4.11	2	699.81	1,397.60	0.00152
1698	PE	PV	100 µM	620	629	LRNYYyDVVN	y6: Phospho (+79.97)	99.60%	1.22	10.7	1.14	2	699.81	1,397.60	-0.00153
1699	PE	PV	100 µM	620	629	LRNYyYDVVN	y5: Phospho (+79.97)	99.70%	2.01	36.3	2.8	2	699.81	1,397.61	0.00360
1700	PE	PV	100 µM	620	629	LRNYyYDVVN	y5: Phospho (+79.97)	99.70%	1.81	25.4	4.1	2	699.81	1,397.60	0.00030
1701	PE	PV	100 µM	620	629	LRNYyYDVVN	y5: Phospho (+79.97)	99.70%	2.5	34.8	4.68	2	699.81	1,397.60	-0.00043
1702	PE	PV	100 µM	620	629	LRNYyYDVVN	y5: Phospho (+79.97)	99.70%	2.78	30	5.37	2	699.81	1,397.60	-0.00043
1703	PE	PV	100 µM	620	629	LRNYyYDVVN	y5: Phospho (+79.97)	99.70%	1.59	29	0.82	2	699.81	1,397.61	0.00750
1704	PE	PV	100 µM	620	629	LRNYYyDVVN	y6: Phospho (+79.97)	99.70%	1.86	45.5	3.34	2	699.81	1,397.60	0.00018
1705	PE	PV	100 µM	620	629	LRNYyYDVVN	y5: Phospho (+79.97)	99.70%	2.65	46.7	4.28	2	699.81	1,397.61	0.00518
1706	PE	PV	100 µM	620	629	LRNYyYDVVN	y5: Phospho (+79.97)	99.70%	1.68	24.1	2.55	2	699.82	1,397.62	0.02117
1707	PE	PV	100 µM	620	629	LRNYyYDVVN	y5: Phospho (+79.97)	99.70%	1.82	19.1	2.96	2	699.82	1,397.62	0.01898
1708	PE	PV	100 µM	620	629	LRNYyYDVVN	y5: Phospho (+79.97)	99.70%	1.74	23.4	3.55	2	699.82	1,397.62	0.02008
1709	PE	PV	100 µM	620	629	LRNYyYDVVN	y5: Phospho (+79.97)	99.70%	1.31	34.5	0.72	2	699.81	1,397.60	-0.00141
1710	PE	Ig	5 min	622	629	NYyYDVVN	y3: Phospho (+79.97)	86.70%	0.98	0	0	2	565.22	1,128.42	0.00191
1711	PE	Ig	15 min	622	629	NYyYDVVN	y3: Phospho (+79.97)	98.80%	0.94	15.9	0	2	565.22	1,128.42	0.00203

[1]PE = phosphopeptide enrichment, experiments were performed as either titanium dioxide or anti-pY pull-downs; IP = anti-SYK immunoprecipitation.

[2]NT = no treatment; Ig = anti-Ig activating antibody; Hg = mercury; PV = pervanadate.

[3]Lower case letters represent modifed amino acid residues.
